# Supplementary material for: Contraceptive progestins with androgenic properties stimulate breast epithelial cell proliferation
Source: EMBO Mol Med. 2021 May 27;13(7):e14314. doi: 10.15252/emmm.202114314 (PMC8261488; doi:10.15252/emmm.202114314)
Supplement: Supplementary file 1 — Appendix [file EMMM-13-e14314-s003.pdf]

Contraceptive progestins with androgenic properties stimulate breast epithelial cell proliferation

APPENDIX.

Table of contents.

Appendix Figure Legend. Pages 2 –3
Appendix Figure S1 Page 4
Appendix Figure S2 Page 5
Appendix Figure S3 Page 6
Appendix Figure S4 Page 7
Appendix Table S1 Page 8
Appendix Table S2 Page 9
Appendix Table S3 Page 10
Appendix Table S4 Page 11

Appendix Fig S1- *Ex vivo* stimulation of murine mammary organoids with different PR agonists.

A Bar plot showing number of differentially expressed genes (adj. *P*.Val < 0.05) between ethanol- and R5020-treated mouse mammary organoids, n=3.

B GSEA showing negative enrichment of TGFβ signaling and mitotic spindle in mouse mammary organoids upon R5020 stimulation.

C GSEA showing enrichment of oxidative phosphorylation and interferon gamma response repair in mouse mammary organoids upon R5020 stimulation, total number of genes = 14,244. NES: Normalized Enrichment Score.

D Bar graphs showing *Rankl* and *Wnt4* transcript levels, relative to 36B4 mRNA expression in mammary organoids derived from C57Bl/6 females 1, 2, and 6 hr after stimulation with 20 nM R5020 (n=12), DSG (n=5), GSN (n=6), LNG (n=6), CMA (n=6), CPA (n=6), or DSP (n=6).

E Bar graphs showing relative *Rankl* and *Wnt4* transcript levels normalized to *36B4* mRNA expression in mammary organoids derived from NSG females 6 hr after stimulation with different progestins, technical replicates are shown.

F Bar graph showing relative *Rankl* and *Wnt4* transcript levels normalized to *36B4* expression in C57Bl6-derived mammary organoids treated for 6 hr with R5020 or LNG and bicalutamide at a concentration of either 10 μM or 100 μM.

Appendix Fig S2- Expression of PR signaling targets in HBECs.

A Bar plot showing number of differentially expressed genes (adj. *P*.Val < 0.05) between ethanol- and progesterone or R5020-treated human EpCAM+ cells , n=3.

B Representative RNAscope micrographs of sections from human breast samples stained with *RANKL* (red) *WNT4* (white) probes and counterstained with DAPI. Scale bars, 10 μM.

Appendix Fig S3- Expression of the AR target *KLK3* in HBECs stimulated with progestins *in vivo*.

A, B Bar plots showing relative *KLK3* transcript levels normalized to *36B4* expression in intraductally-engrafted HBECs derived from different patients upon 60-day-exposure to CTRL (n=8), different

androgenic (n=6), DSG (n=1), GSN (n=1), LNG (n=4), and antiandrogenic progestins (n=3); CMA (n=2) and DSP (n=1), shown as groups (A) or by individual compound (B).

Appendix Fig S4- Histological changes after long-term exposure to LNG.

A Whole slide micrographs showing xenografted HBECs derived from 3 different mammoplasties after the host mice were treated for 8 months with either control pellet (upper panels) or LNG pellet (lower panels). Scalebars, 1 mm.

Appendix Table S1: Patient characteristics (Figure 5A-C)

Appendix Table S2: Mammoplasties used for 60-day-treatment experiments with progestogens (Fig 5A-C) and enzalutamide as well as AR down modulation (Fig 7)

Appendix Table S3: Patient characteristics (Figure 5K-L)

Appendix Table S4: Mammoplasties used for 21-day-stimulation experiments (Figure 5K-L)

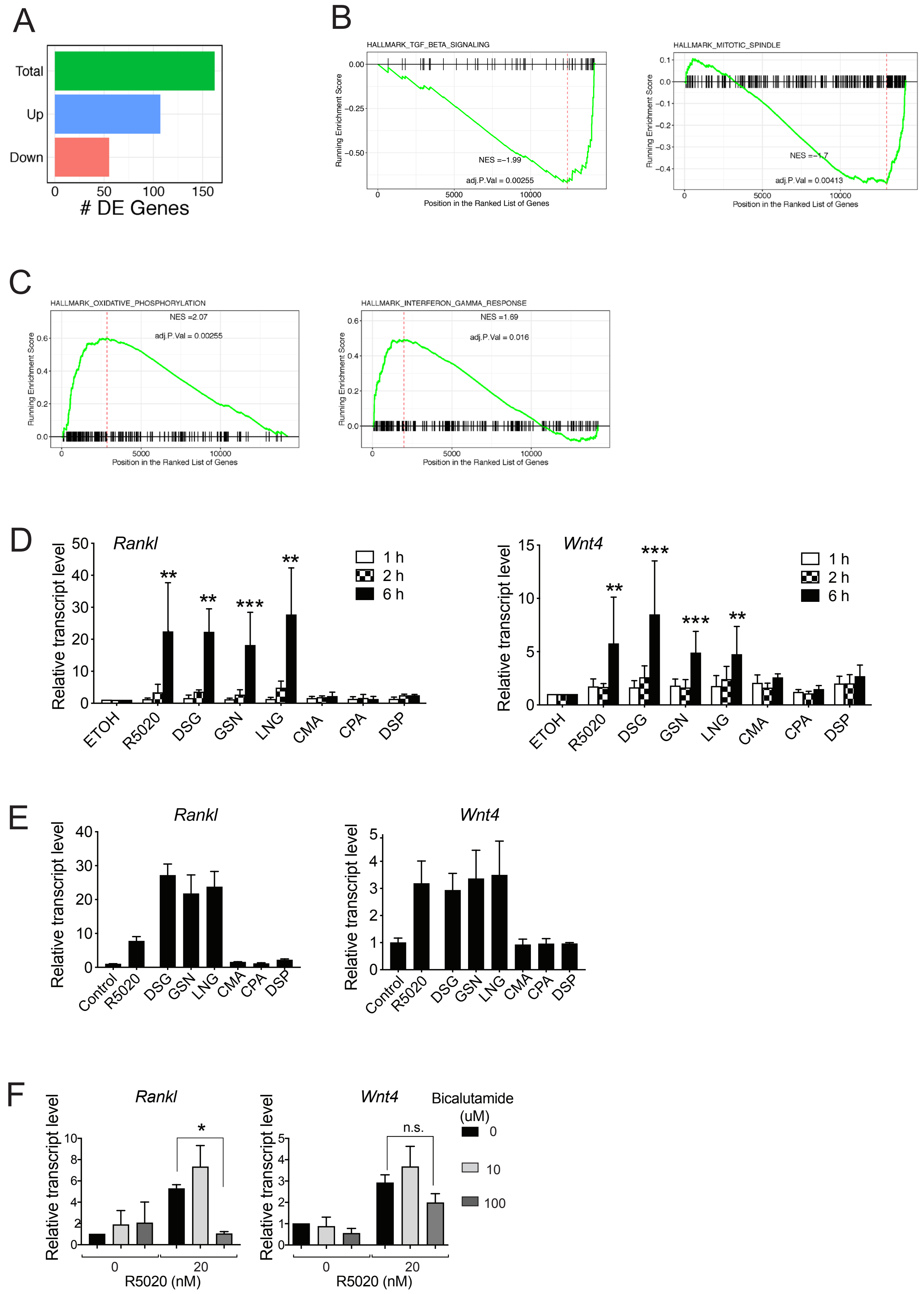
A
Total
Up
Down
0
50
100
150
DE Genes
B
HALLMARK_TGF_BETA_SIGNALING
Running Enrichment Score
NES =−1.99
adj.P.Val = 0.00255
Position in the Ranked List of Genes
HALLMARK_MITOTIC_SPINDLE
NES =−1.7
adj.P.Val = 0.00413
C
HALLMARK_OXIDATIVE_PHOSPHORYLATION
NES =2.07
adj.P.Val = 0.00255
HALLMARK_INTERFERON_GAMMA_RESPONSE
NES =1.69
adj.P.Val = 0.016
D
Rankl
Wnt4
Relative transcript level
1 h
2 h
6 h
ETOH
R5020
DSG
GSN
LNG
CMA
CPA
DSP
E
Control
F
Bicalutamide (uM)
0
10
100
n.s.
R5020 (nM)

Appendix Figure S2

A

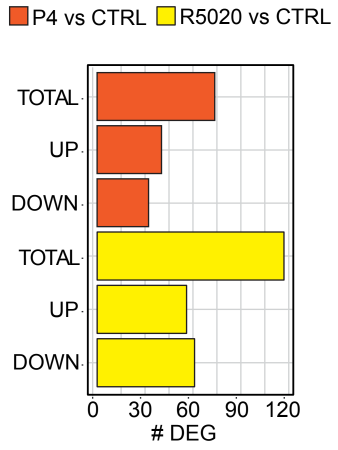

B

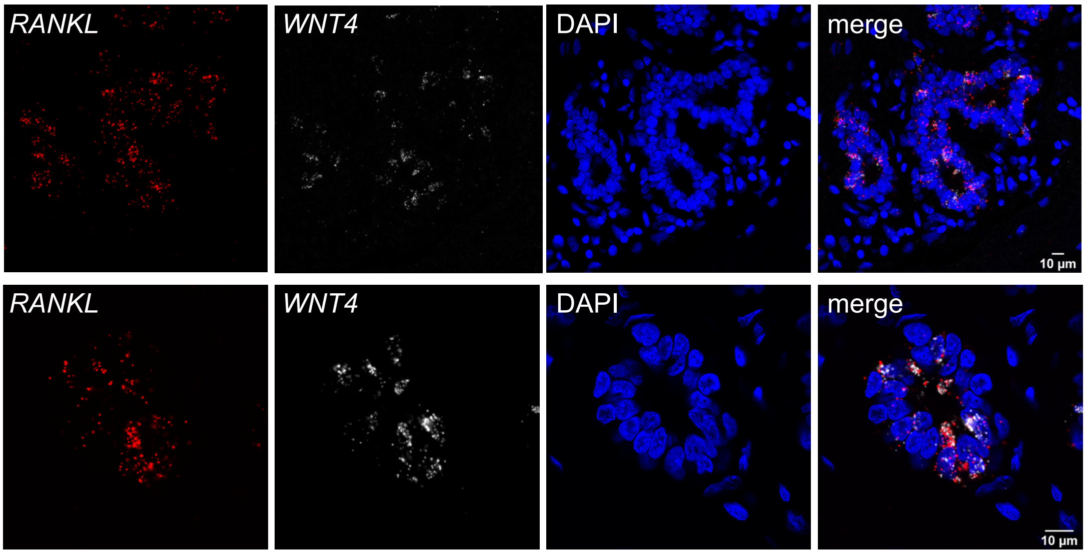

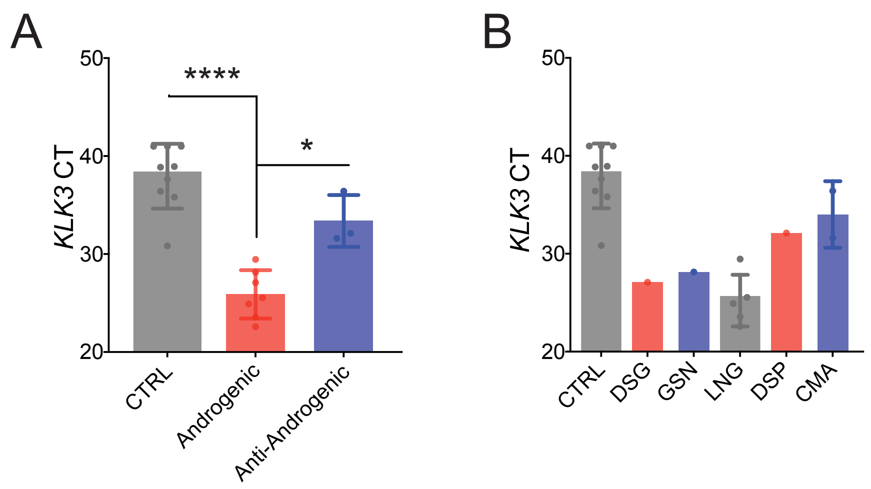
A
50
40
30
20
KLK3 CT
****
*
CTRL
Androgenic
Anti-Androgenic
B
50
40
30
20
KLK3 CT
CTRL
DSG
GSN
LNG
DSP
CMA

Vehicle

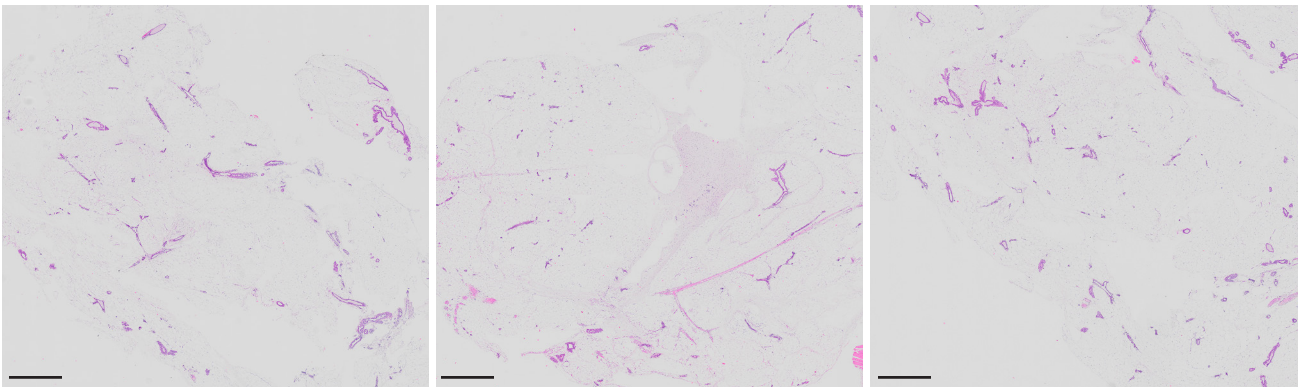

LNG

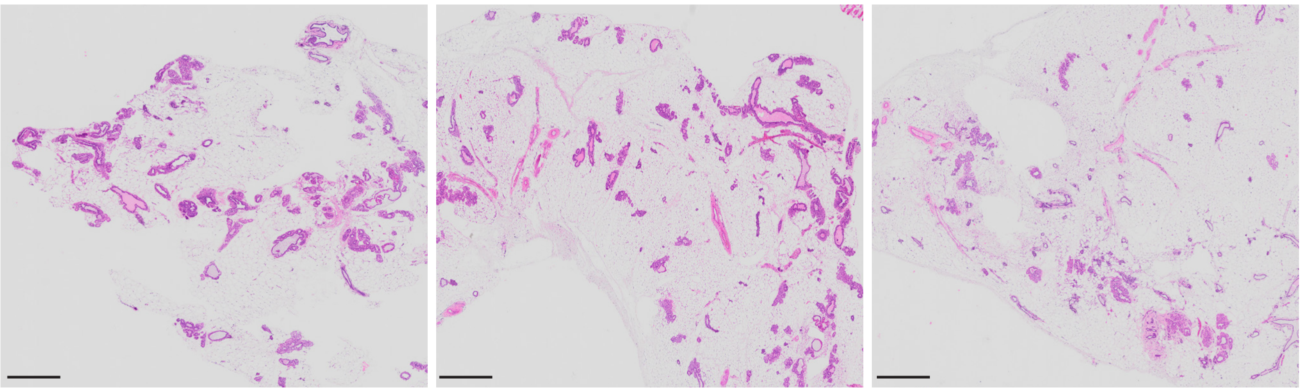

Appendix Table S1: Patient characteristics (Figure 5A-C)

ID	Age (y)	Ethnicity	Menopause	Hormonal contraception	Progestin	Plasma levels (ng/ml)
242	20	NA	NO	NO	NO	-
250	27	NA	NO	NO	NA	-
282	32	Caucasian	NO	NA	CMA	1,25
285	45	Caucasian	NO	NO	NO	-
287	50	African	NO	Stérilet	LNG	0,24
289	18	Caucasian	NO	Pill	LNG	0,56
290	28	NA	NO	NA	NO	-
291	45	Caucasian	NO	NO	NO	-
294	18	African	NO	NO	NO	-
295	18	NA	NO	NA	CMA	0,33
296	46	NA	NO	NA	NO	-
297	71	Caucasian	YES	NA	NO	-
303	46	Caucasian	NO	Stérilet	LNG	0,125
304	45	Caucasian	NO	Depo-Provera 150	MPA	2,855
306	26	Caucasian	NO	Pill	GSN	2,701
307	18	Caucasian	NO	NO	NO	-
308	22	NA	NO	NO	NO	
309	24	African	NO	NO	NO	-
310	19	Caucasian	NO	NO	NO	-
311	33	Caucasian	NO	Stérilet	LNG	0,308
313	47	Caucasian	NO	Pill	NO	-
318	22	Caucasian	NO	Microgynon	NomAC	0,109
319	NA	NA	NA	NA	NO	-
320	20	Caucasian	NO	NO	NO	-
321	49	Caucasian	YES	Pill	NO	-
323	32	Caucasian	NO	NO	NO	-
324	NA	NA	YES	NA	DSG	0,301
326	NA	NA	NA	NA	NO	-
327	45	American	YES	NO	NO	-
328	20	Caucasian	NO	Pill	LNG	1,422
329	49	NA	NA	NA	NomAC	0,100
330	18	Caucasian	NO	NO	NO	-
341	20	Caucasian	NO	NO	-	-
354	36	Caucasian	NO	Stérilet	LNG	-
362	29	Caucasian	NO	Depo-Provera 150	MPA	-
369	19	Caucasian	NO	NA	NA	-

IUD: intrauterine device

Appendix Table S2: Mammoplasties used for 60-day-stimulation experiments with progestogens (Fig 5A-C) as well as enzalutamide and AR down modulation (Fig 7)

Mammo plasty ID	# of mice	Treatment									
		CTRL	GSN	DSG	LNG	DSP	CMA	CPA	P4	ENZ	shAR
M242	4	X	X								
M250	2	X	X								
M282	8	X			X		X				
M285	10	X	X	X	X		X				
M287	18	X	X			X	X				
M289	5	X	X			X					
M290	4	X						X			
M291	6	X	X			X					
M294	13	X		X	X	X	X	X	X		
M295	9	X		X	X		X		X		
M296	7	X			X	X		X			
M297	7	X	X				X		X		
M303	5	X			X						
M306	6	X		X				X			
M307	6	X	X								
M311	7	X							X		
M313	6	X				X					
M318	4	X							X		
M319	5	X							X		
M320	4	X							X		
M321	16	X			X					X	
M323	4	X		X							
M324	4	X							X		
M326	4	X					X				
M327	12	X			X					X	
M328	4	X							X		
M329	12	X			X					X	
M330	4	X							X		
M341	12	X			X						X

Appendix Table S3: Patient characteristics (Figure 5K-L)

ID	Age (y)	Ethnicity	Menopause	Hormonal contraception	Progestin	Plasma levels (ng/ml)
304	45	Caucasian	NO	Depo-Provera 150	MPA	2,855
308	22	NA	NO	NO	NO	
309	24	African	NO	NO	NO	-
310	19	Caucasian	NO	NO	NO	-
311	33	Caucasian	NO	Stérilet	LNG	0,308
318	22	Caucasian	NO	Microgynon	NomAC	0,109
320	20	Caucasian	NO	NO	NO	-
321	49	Caucasian	YES	Pill	NO	-
341	20	Caucasian	NO	NO	-	-
354	36	Caucasian	NO	Stérilet	LNG	-
362	29	Caucasian	NO	Depo-Provera 150	MPA	-
369	19	Caucasian	NO	NA	NA	-

IUD: intrauterine device

Appendix Table S4: Mammoplasties used for 21-day-stimulation experiments (Figure 5K-L)

Mammoplasty ID	# of mice	Treatment							
		CTRL	P4	DSG	GSN	LNG	CMA	CPA	DSP
M304	3	X				X			
M308	3	X			X	X	X	X	
M309	3	X		X	X	X	X	X	
M310	3	X	X	X	X	X	X	X	X
M311	6	X	X	X	X				
M318	3	X	X	X	X	X	X	X	X
M320	3	X	X	X	X	X	X	X	X
M321	3	X			X	X			
M341	3	X	X	X	X	X	X	X	X
M354	6	X		X	X	X	X	X	X
M362	6	X	X	X	X	X	X	X	
M369	3		X	X	X	X	X		
