# Supplementary material for: Contraceptive progestins with androgenic properties stimulate breast epithelial cell proliferation
Source: EMBO Mol Med. 2021 May 27;13(7):e14314. doi: 10.15252/emmm.202114314 (PMC8261488; doi:10.15252/emmm.202114314)
Supplement: Supplementary file 3 — Source Data for Figure 3 [file EMMM-13-e14314-s004.pdf]

Table of contents:

- A_Pages 2-37: growth curves describing proliferative trends of untreated intraductally xenografted mammary glands per each patient included in our study. In red, polynomial regression fit. n = number of individuals xenografted mouse mammary gland per each PDX.

- B_Pages 38-92: instances of histological images stained with H&E showing primary human breast epithelial cells isolated from different donors.

- C_Pages 93-117: instances of immunofluorescence images of intraductally xenografted primary human breast epithelial cells stained with anti-E-cadherin (green – human specific) antibody and counterstained with DAPI (white).

- D_Page 118: representative immunofluorescence images of intraductal xenografts stained with anti-ER, PR or AR (red) and anti-E-cadherin (green – human specific) antibodies counterstained with DAPI (blue).

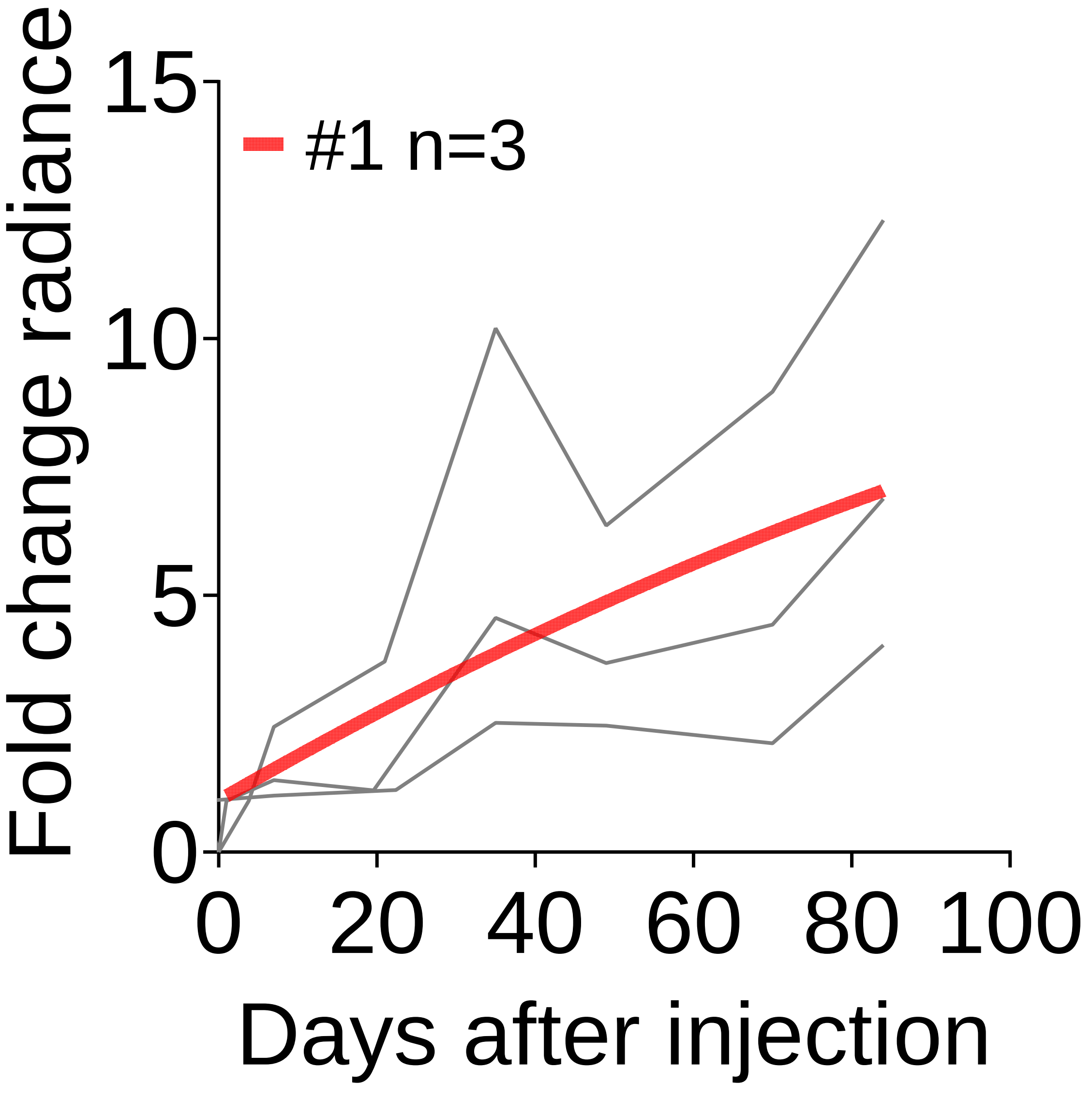

Fold change radiance
15
10
5
0
#1 n=3
0
20
40
60
80
100
Days after injection

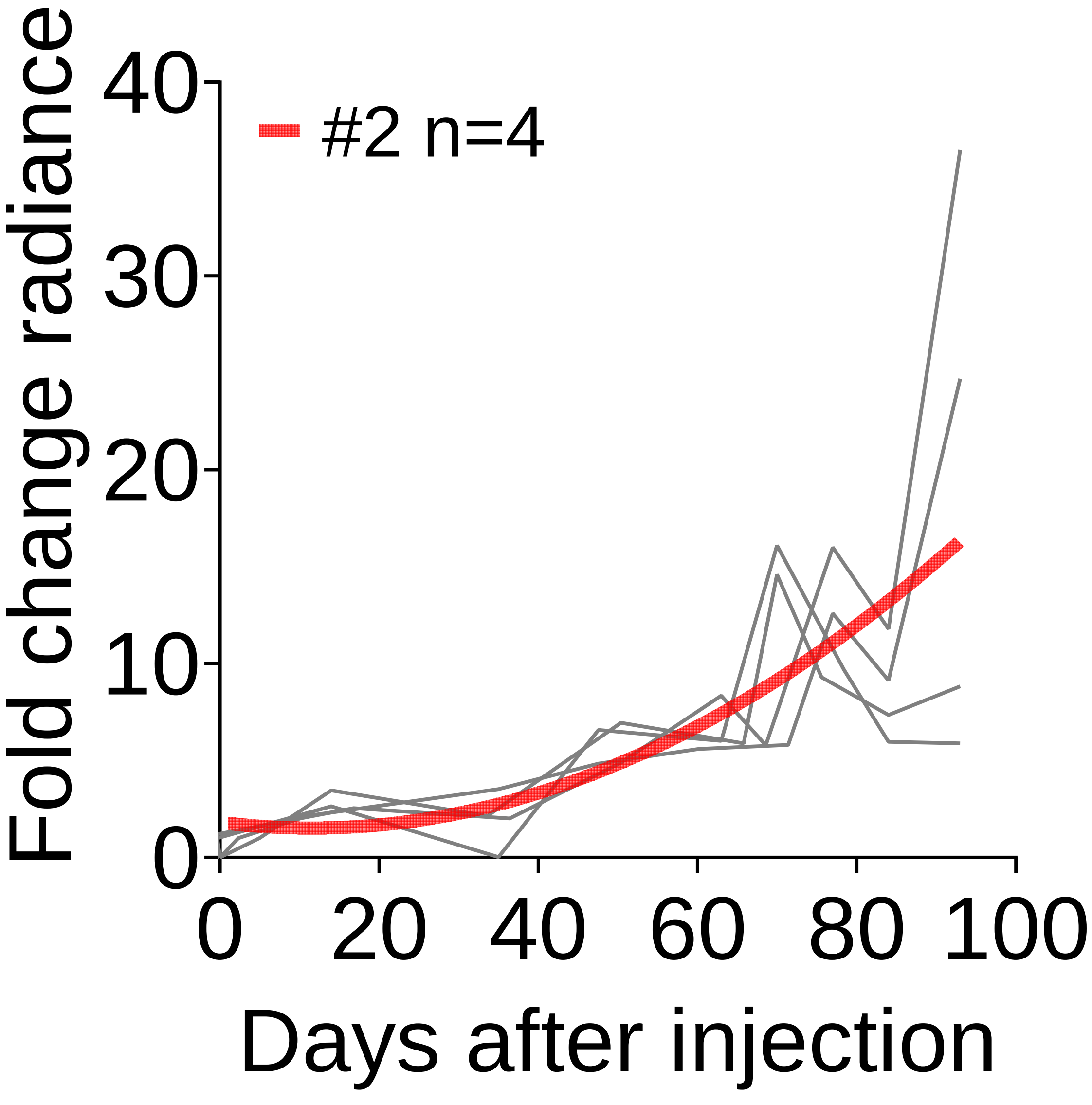

Fold change radiance
#2 n=4
0
10
20
30
40
0
20
40
60
80
100
Days after injection

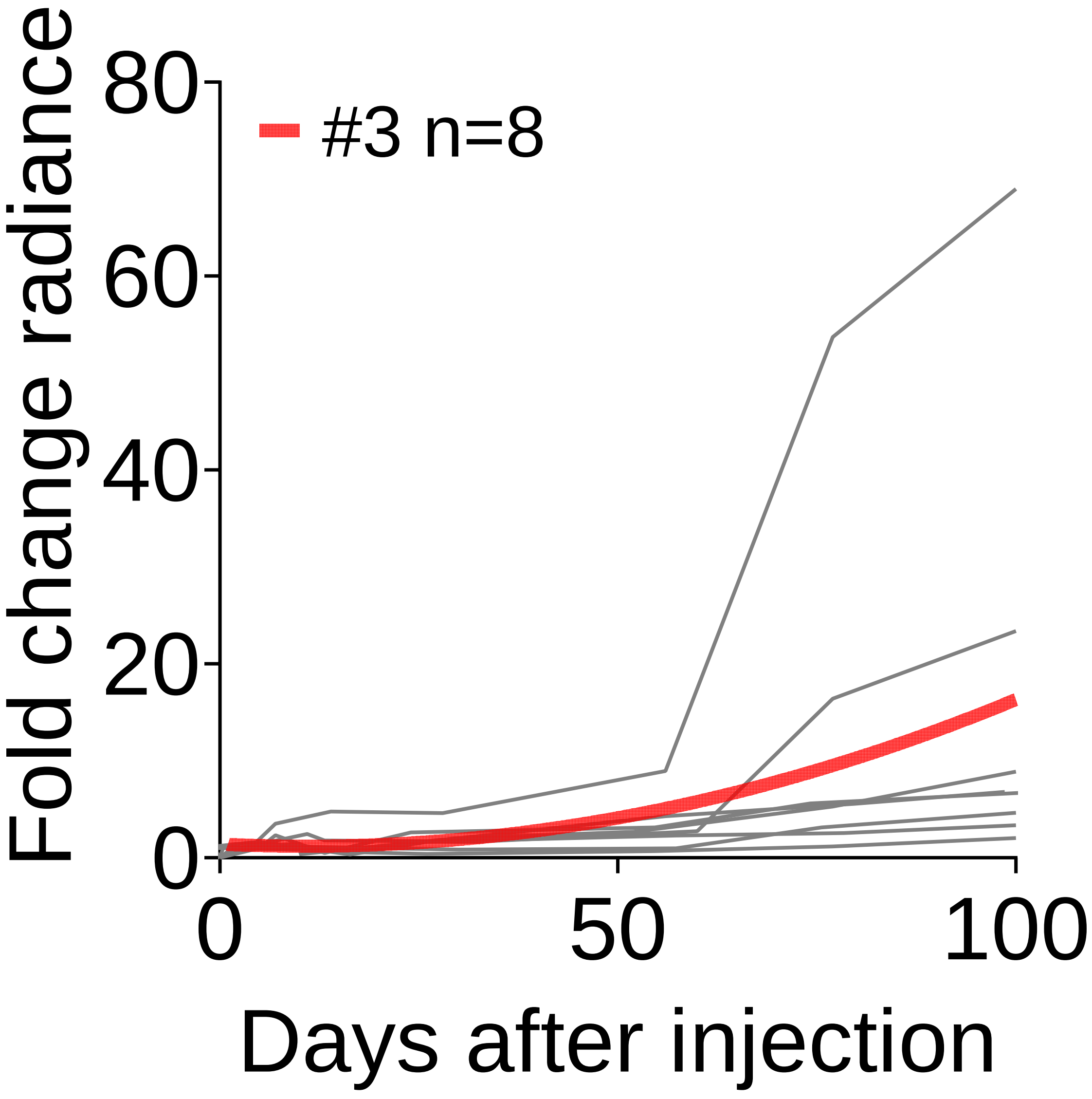

#3 n=8
Fold change radiance
0
20
40
60
80
0
50
100
Days after injection

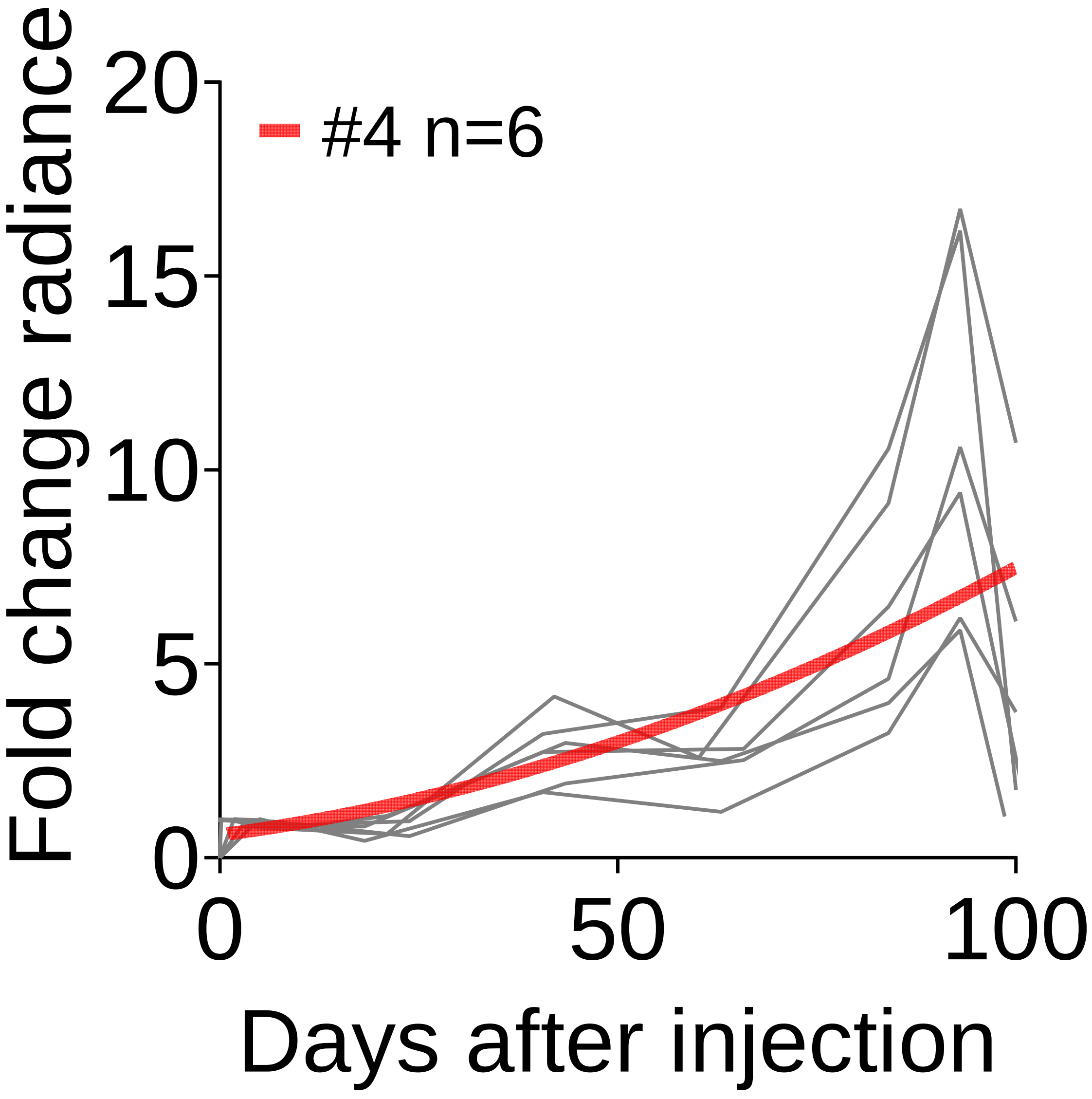

Fold change radiance
20
15
10
5
0
#4 n=6
0
50
100
Days after injection

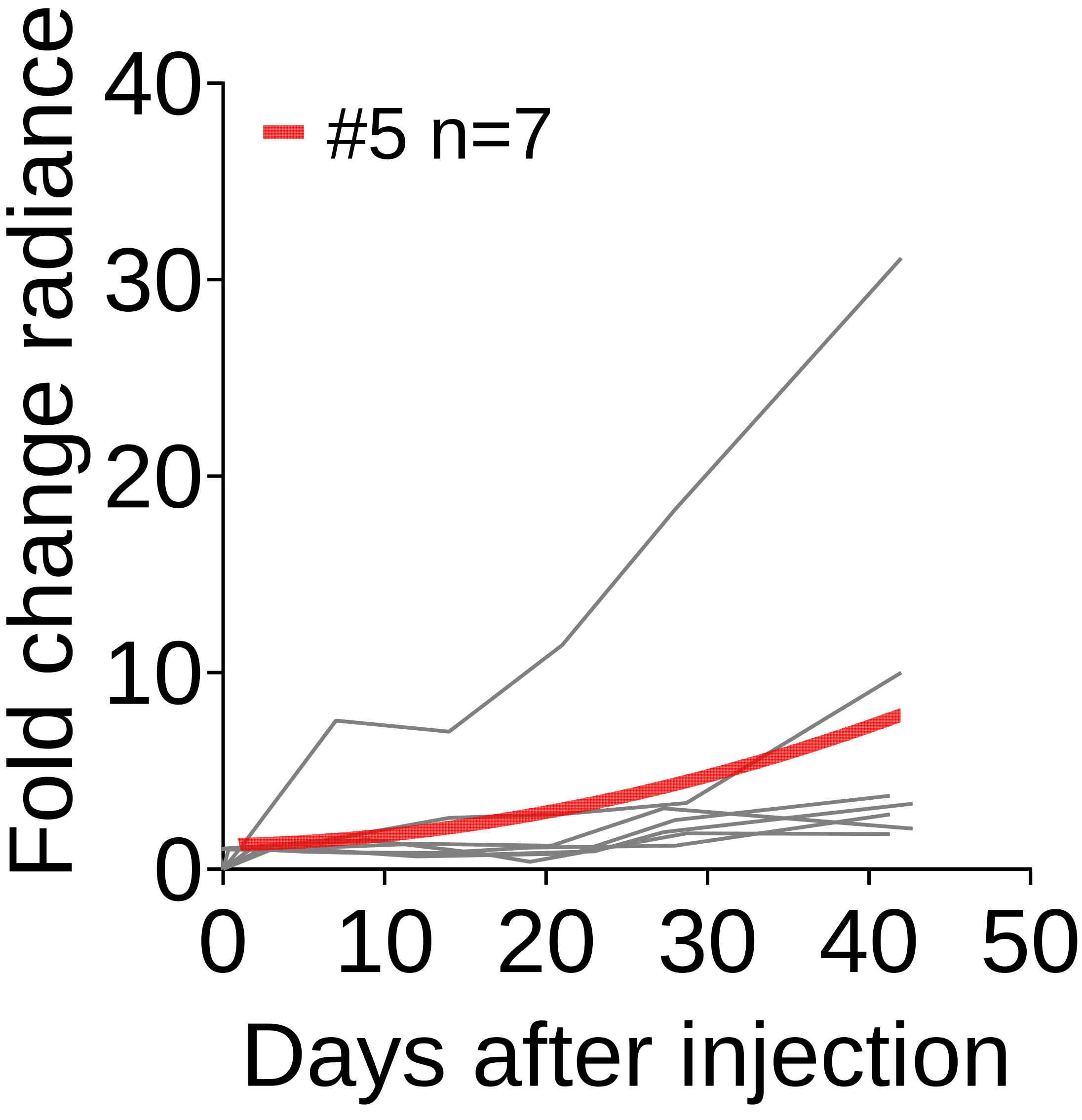
#5 n=7
Fold change radiance
0
10
20
30
40
0
10
20
30
40
50
Days after injection

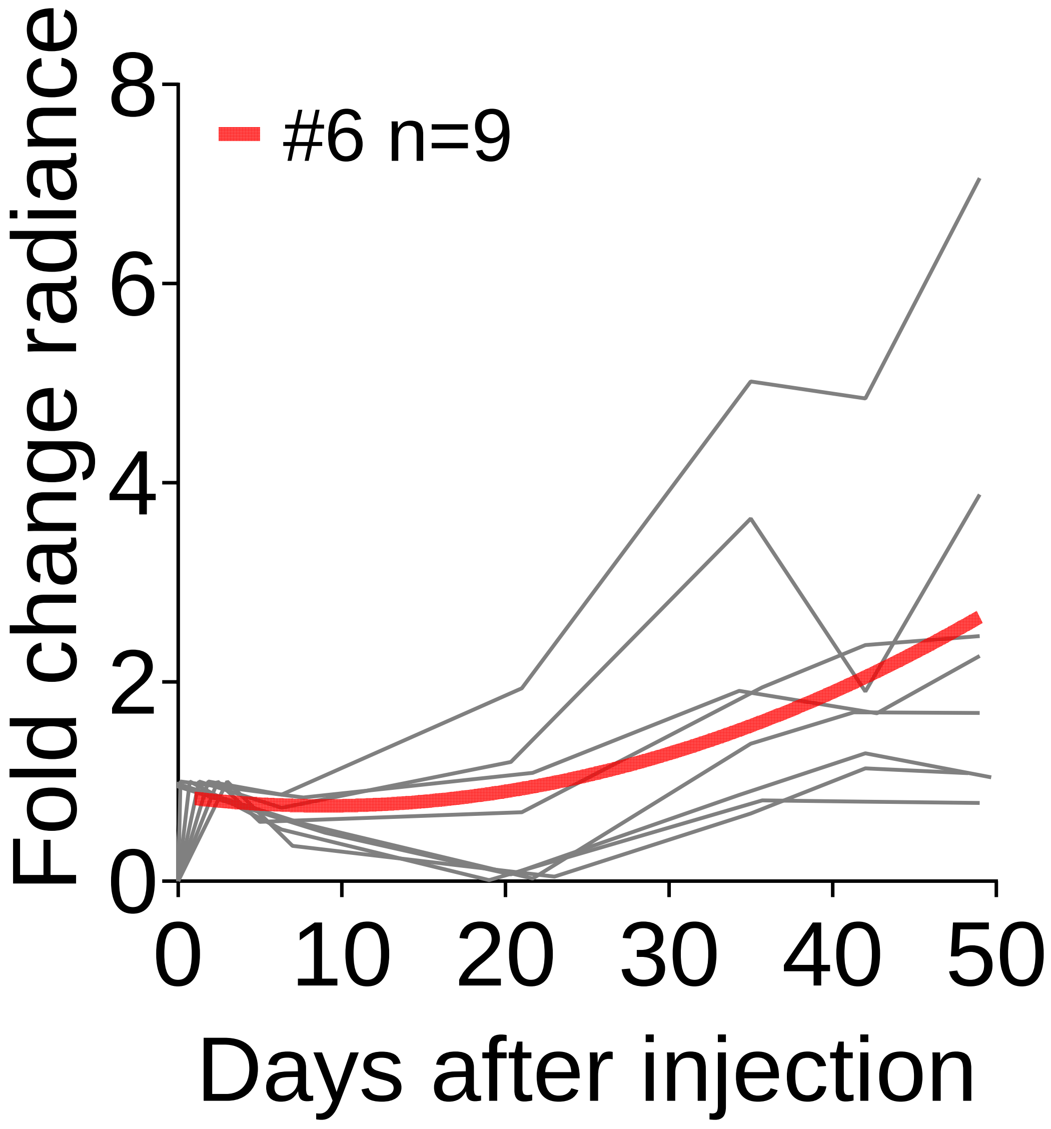

#6 n=9
Fold change radiance
8
6
4
2
0
0
10
20
30
40
50
Days after injection

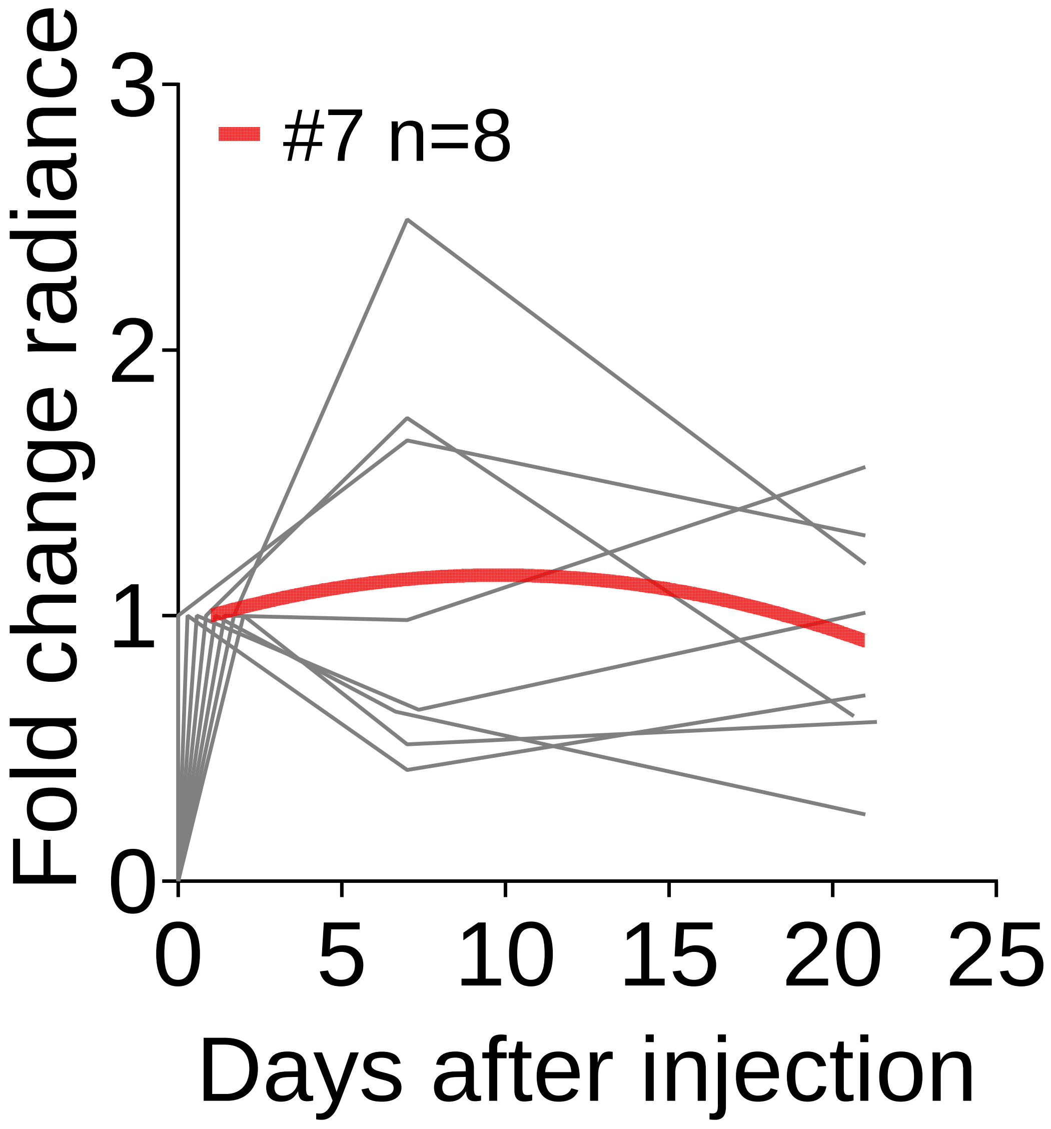

Fold change radiance
3
2
1
0
#7 n=8
0
5
10
15
20
25
Days after injection

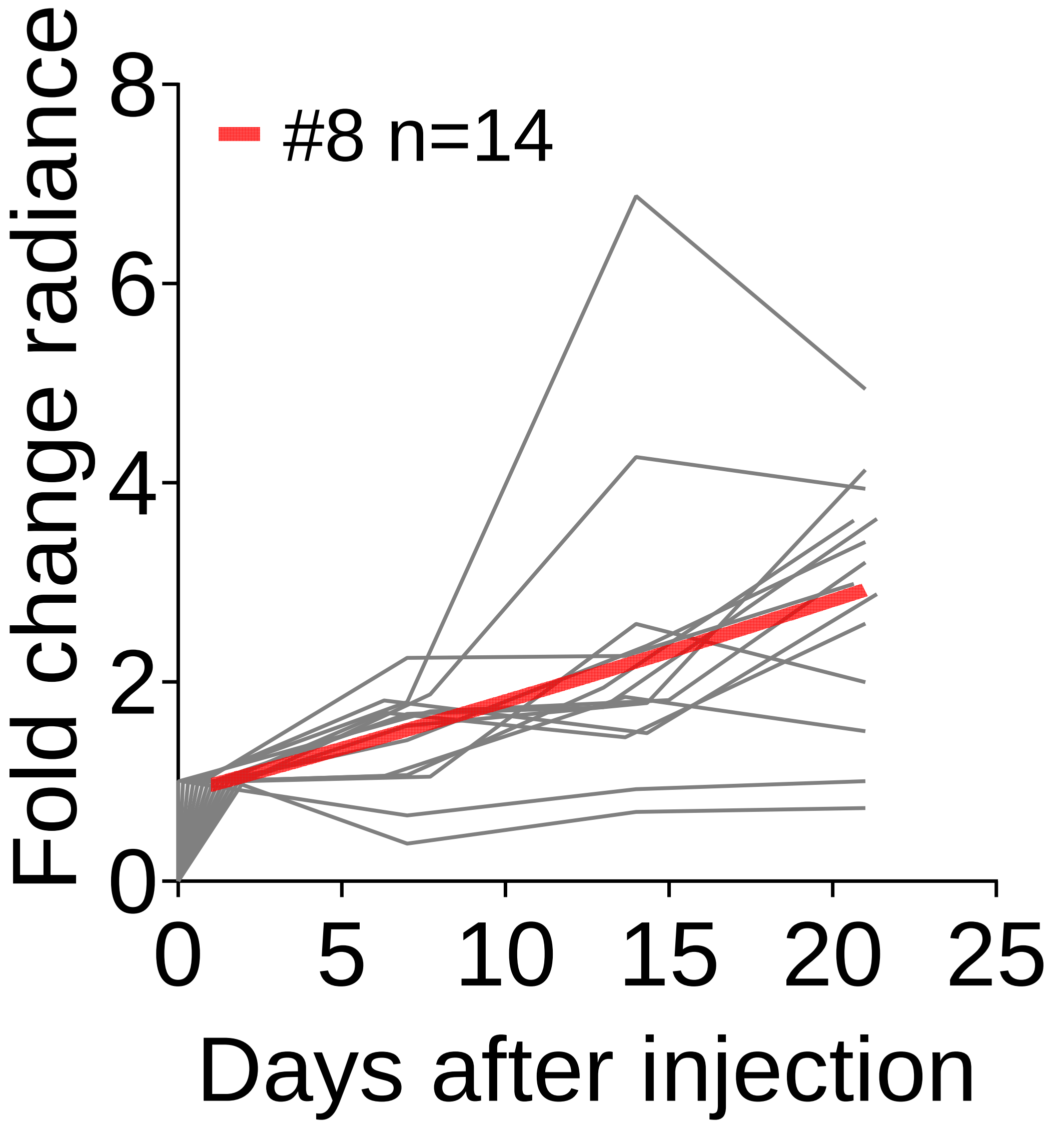

#8 n=14
Fold change radiance
0
2
4
6
8
0
5
10
15
20
25
Days after injection

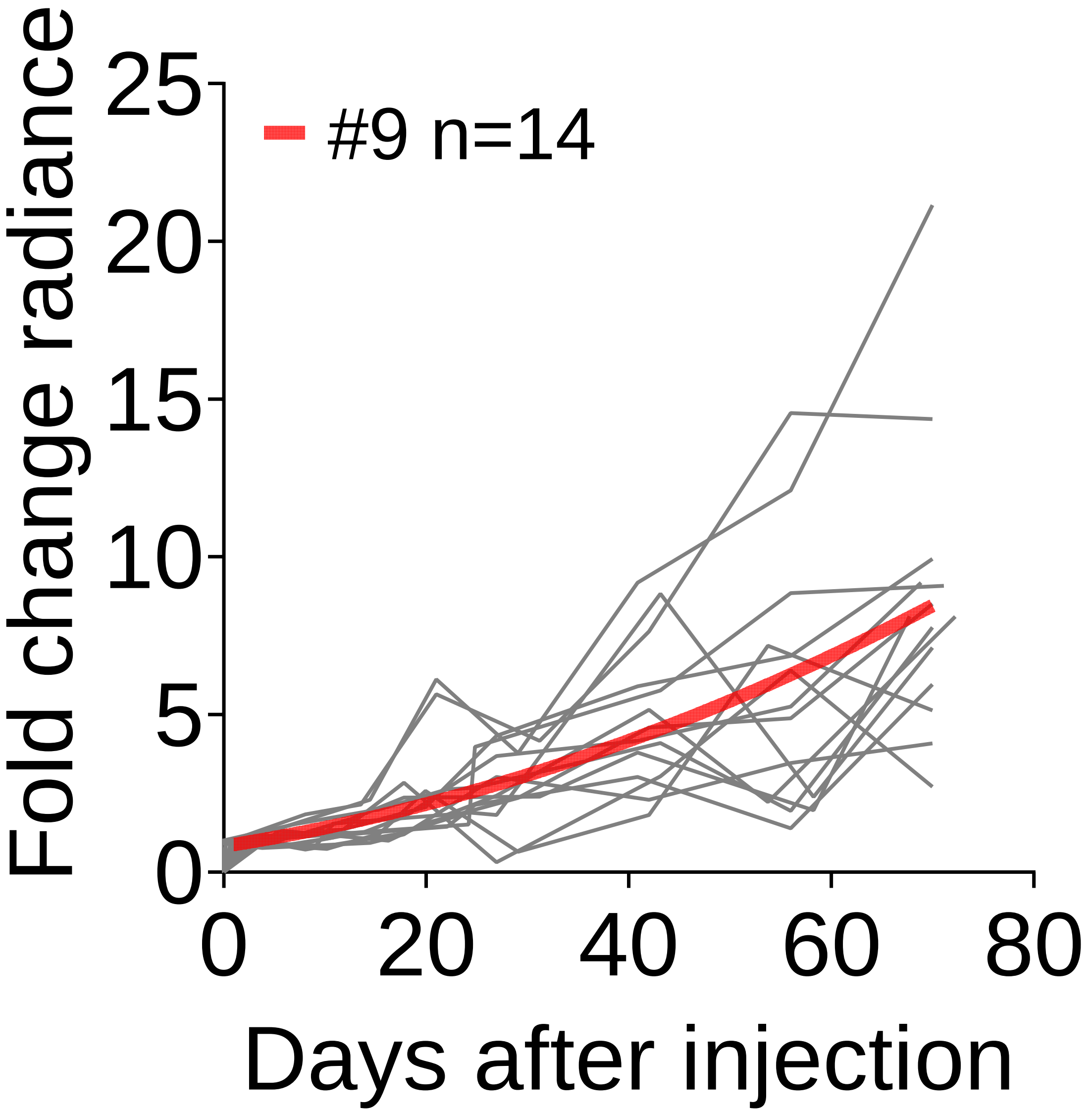

#9 n=14
Fold change radiance
0
5
10
15
20
25
0
20
40
60
80
Days after injection

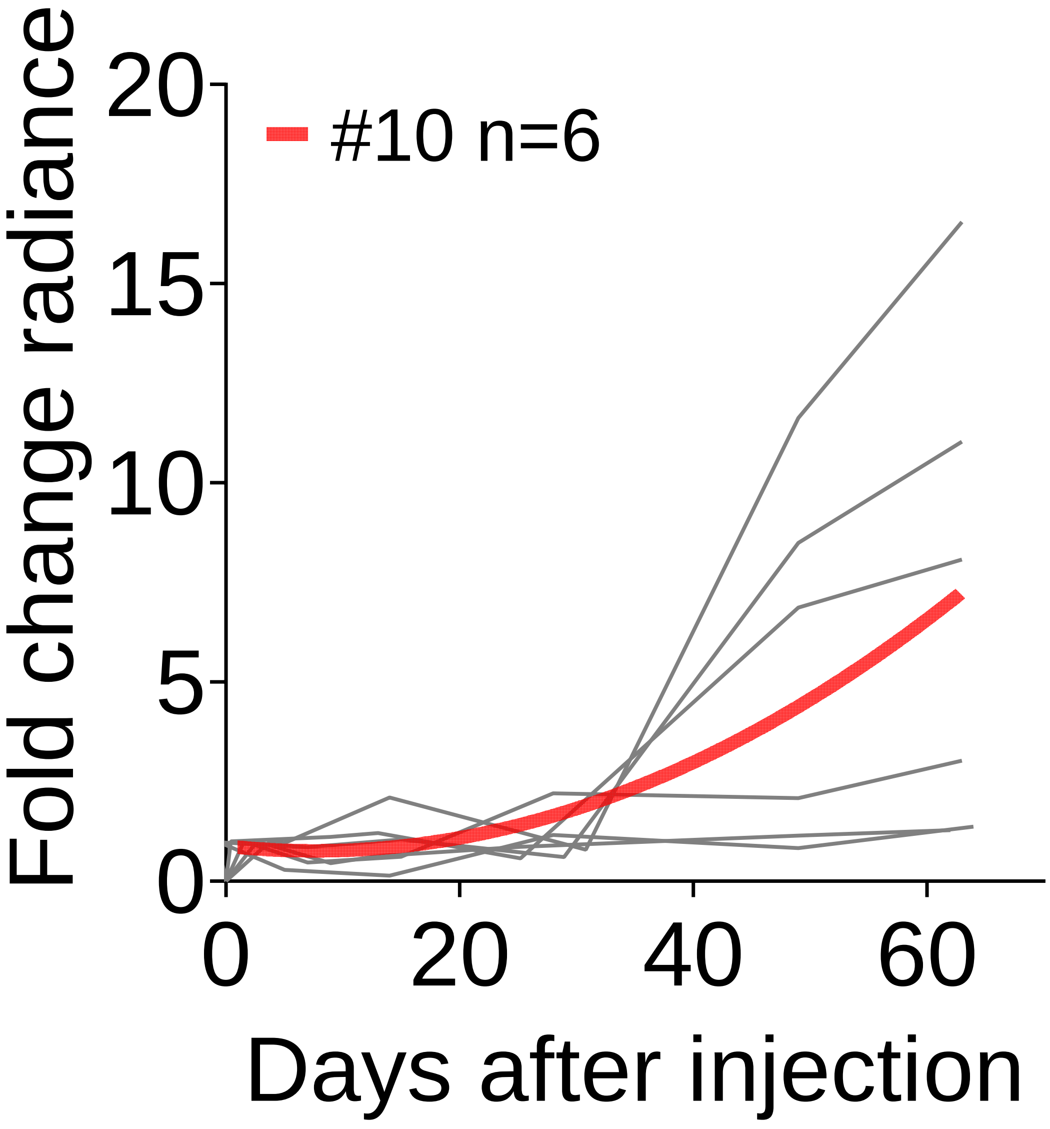

Fold change radiance
20
15
10
5
0
#10 n=6
0
20
40
60
Days after injection

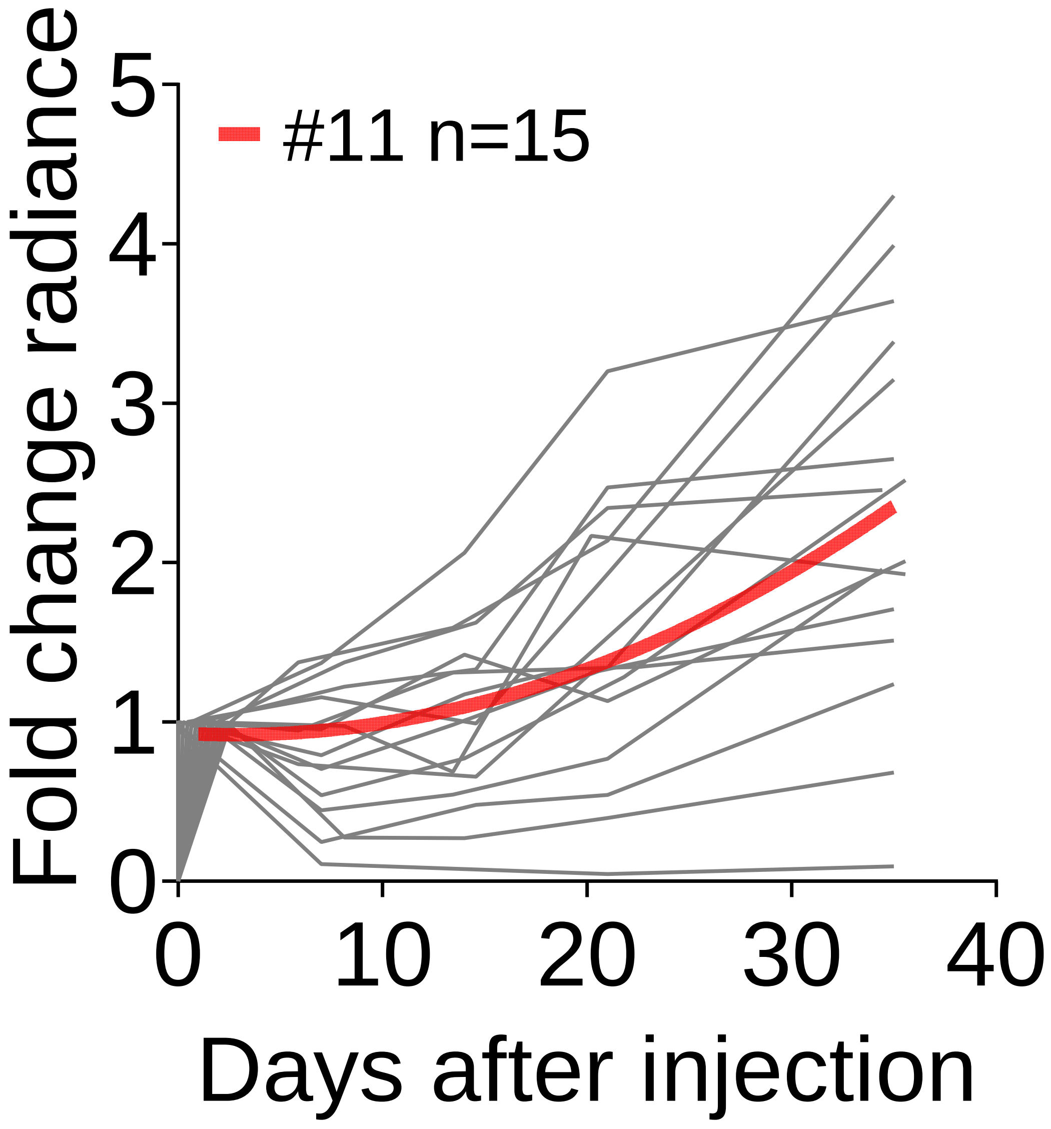

Fold change radiance
5
4
3
2
1
0
#11 n=15
0
10
20
30
40
Days after injection

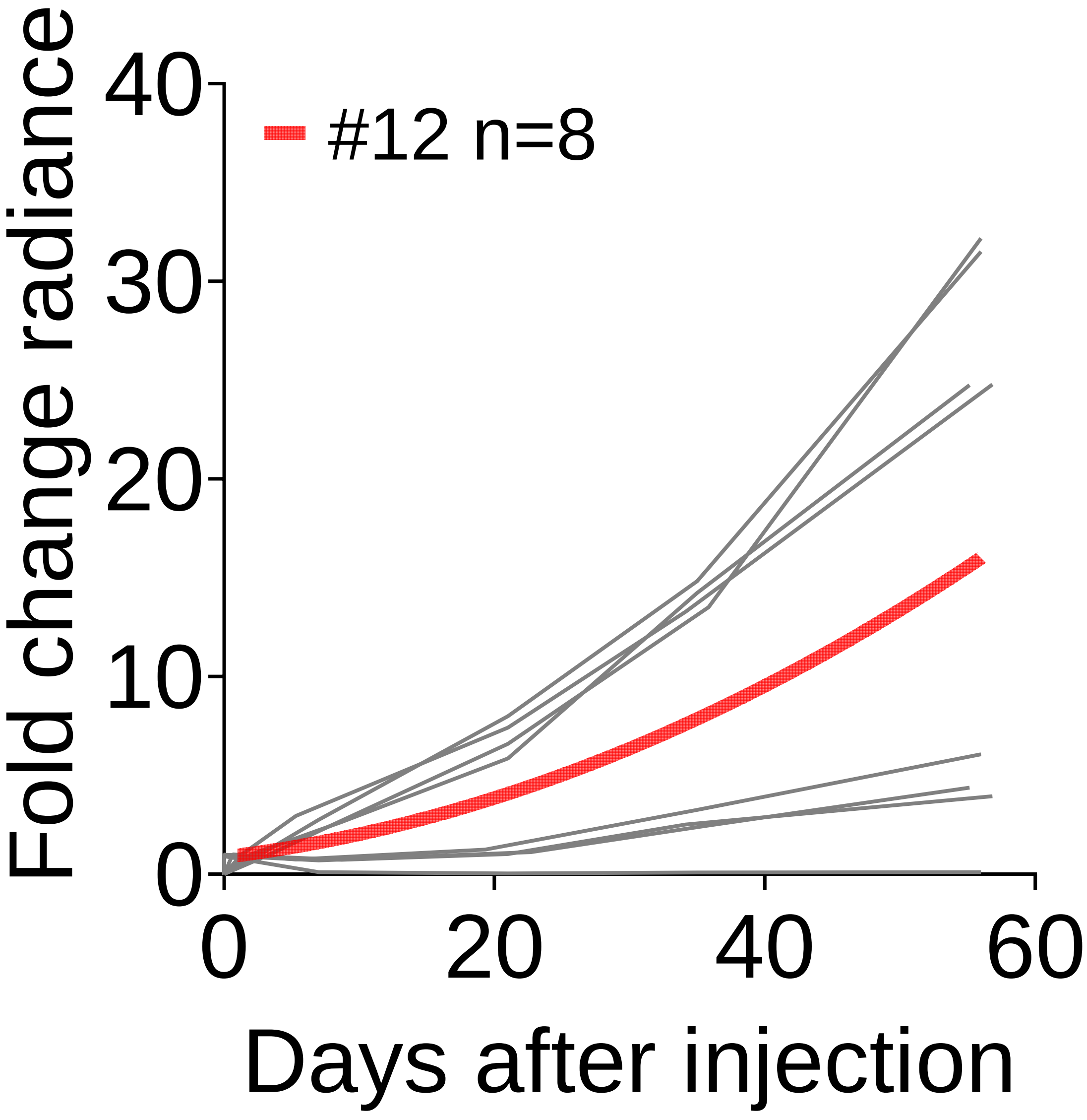

Fold change radiance
0
10
20
30
40
#12 n=8
0
20
40
60
Days after injection

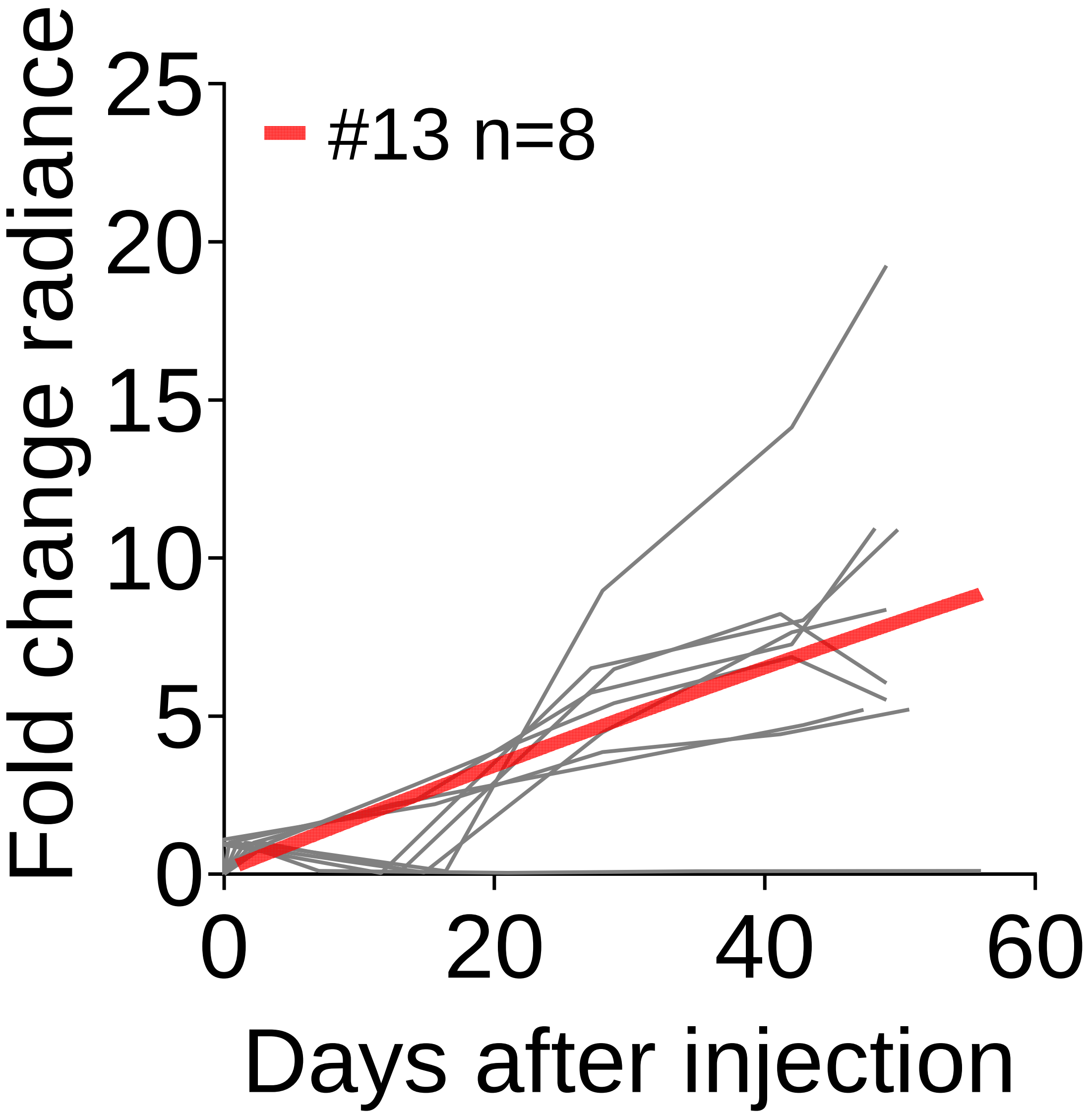

Fold change radiance
25
20
15
10
5
0
#13 n=8
0
20
40
60
Days after injection

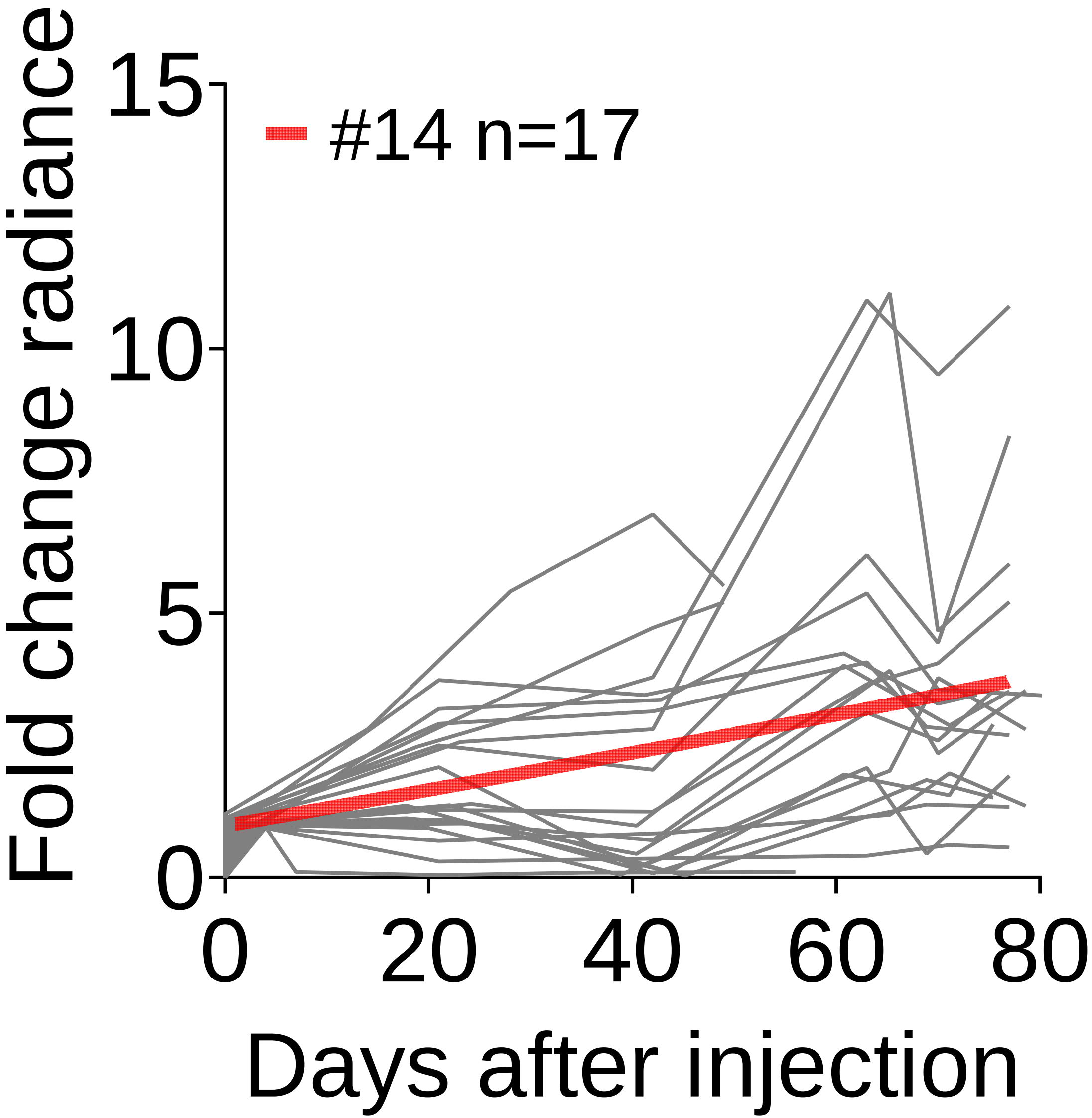
Fold change radiance
15
10
5
0
#14 n=17
0
20
40
60
80
Days after injection

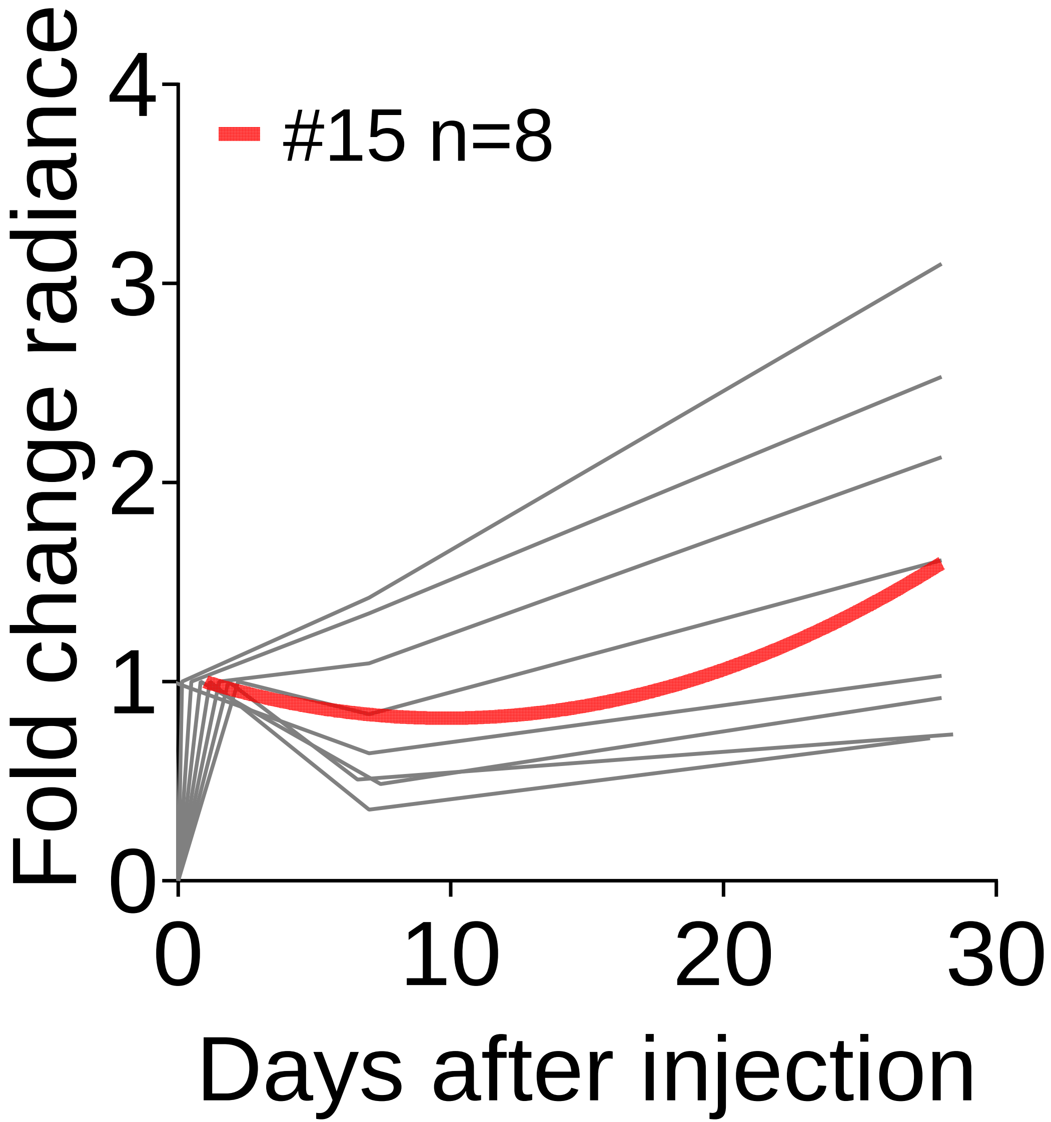
Fold change radiance
0
1
2
3
4
#15 n=8
0
10
20
30
Days after injection

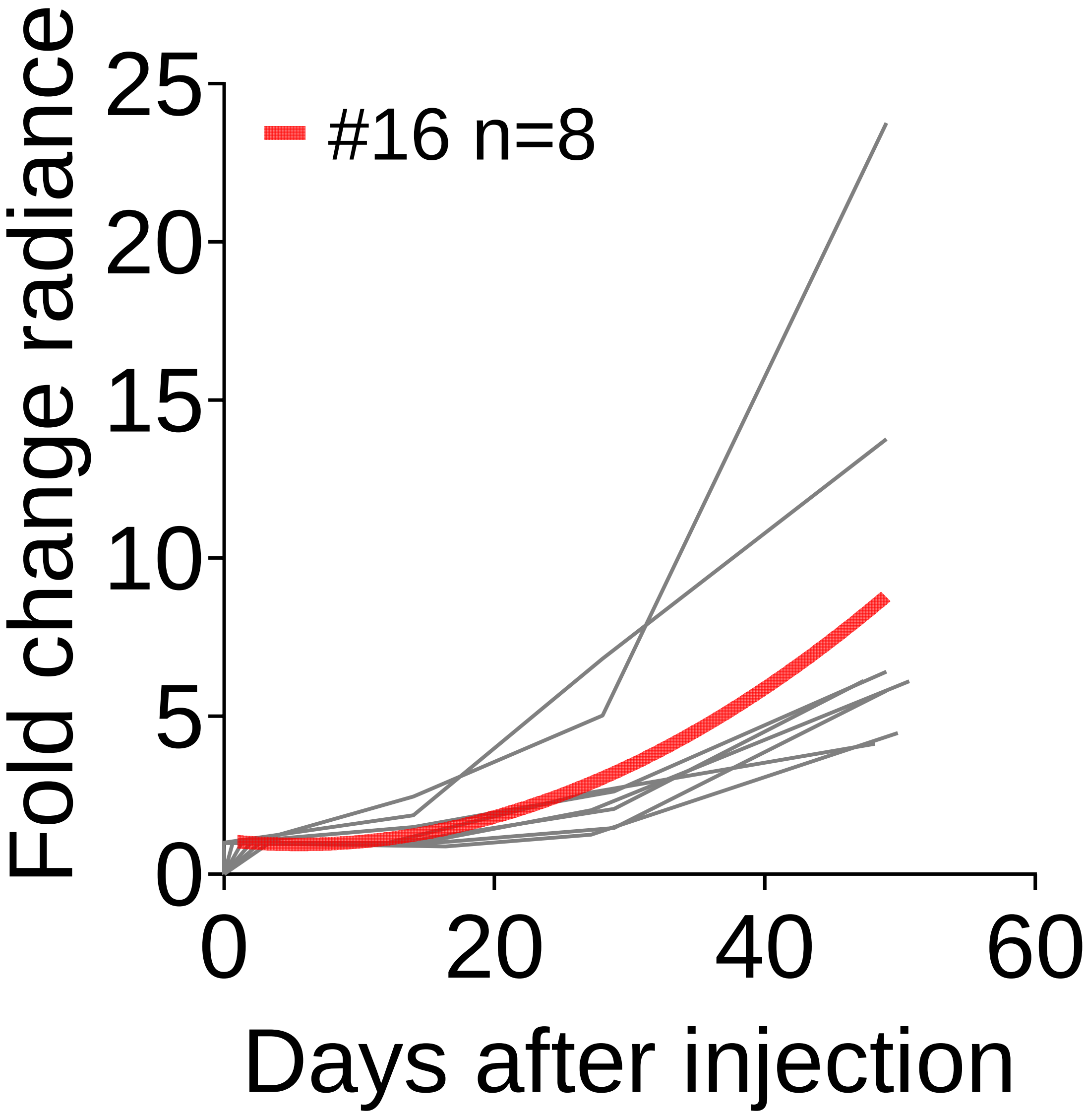

Fold change radiance
25
20
15
10
5
0
#16 n=8
0
20
40
60
Days after injection

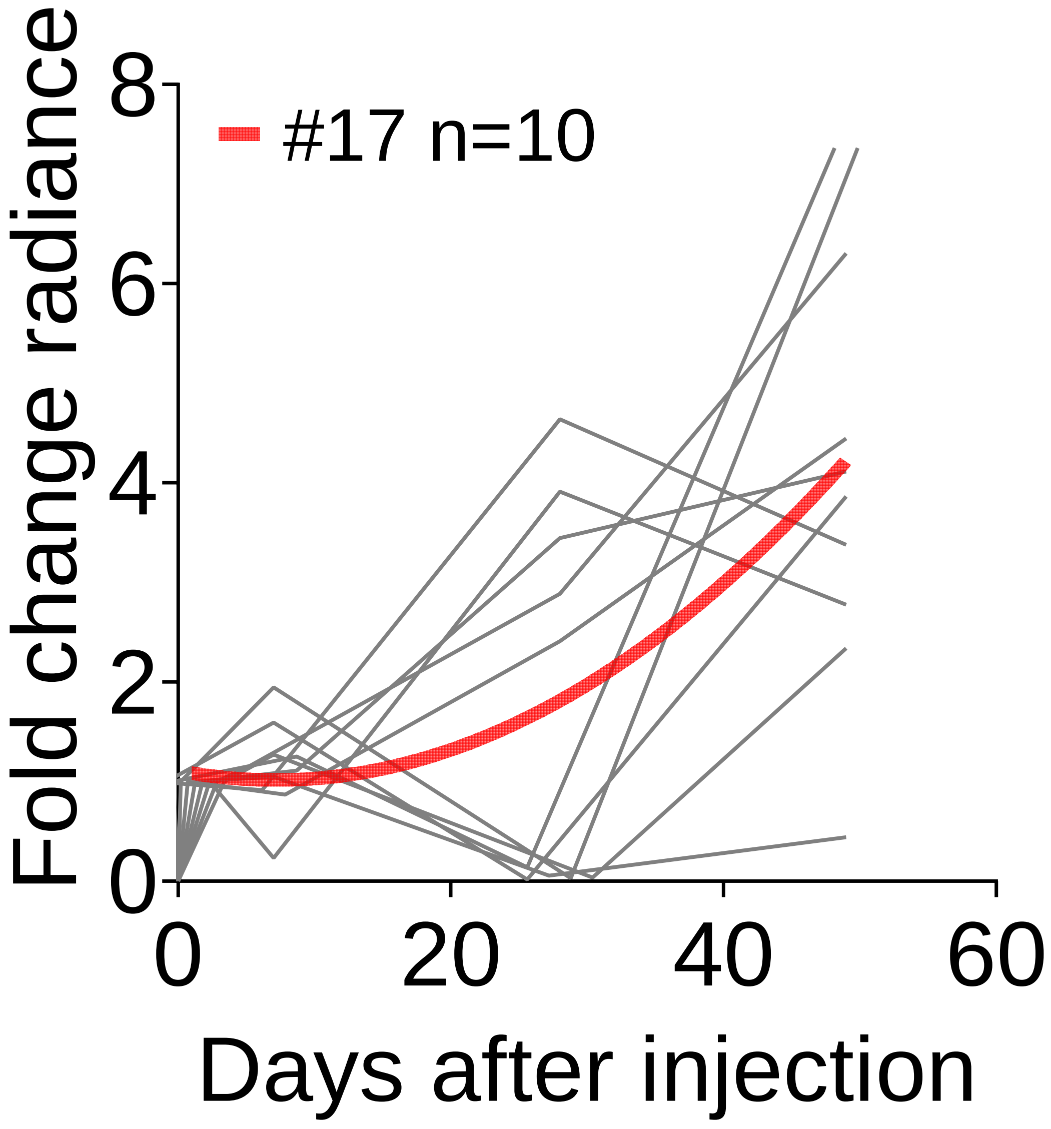
#17 n=10
Fold change radiance
Days after injection
0
2
4
6
8
0
20
40
60

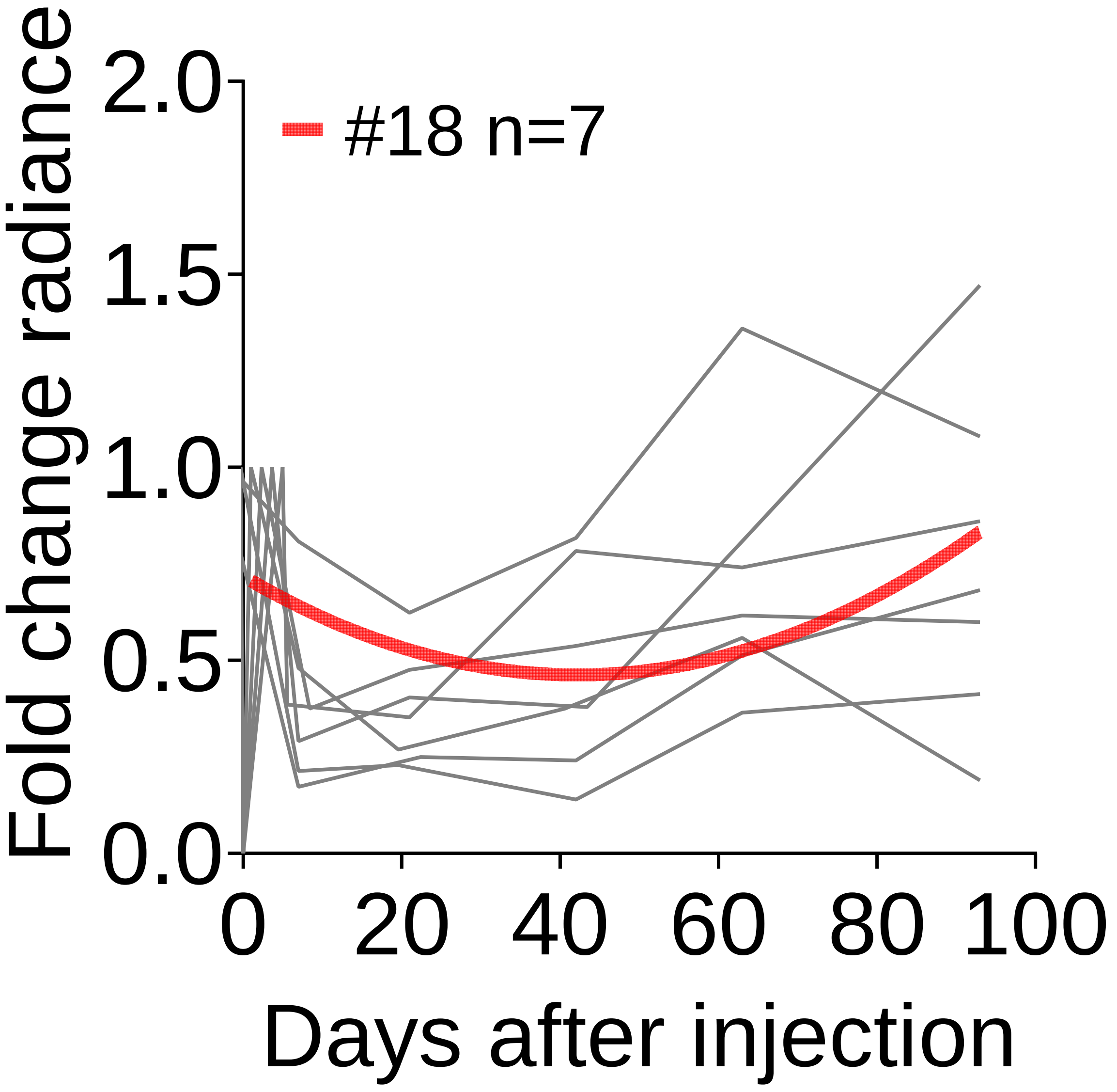

Fold change radiance
2.0
1.5
1.0
0.5
0.0
#18 n=7
0
20
40
60
80
100
Days after injection

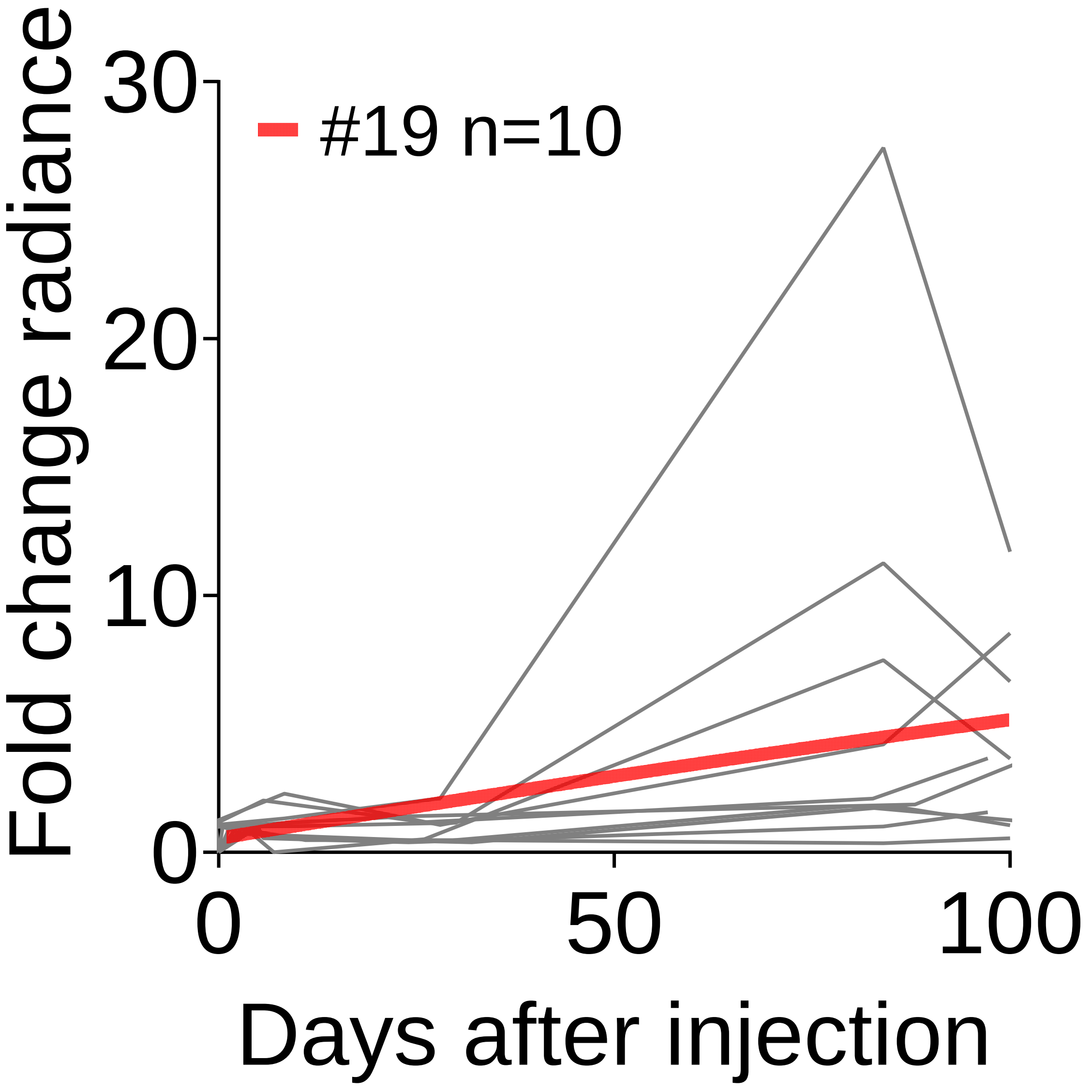

Fold change radiance
30
20
10
0
#19 n=10
0
50
100
Days after injection

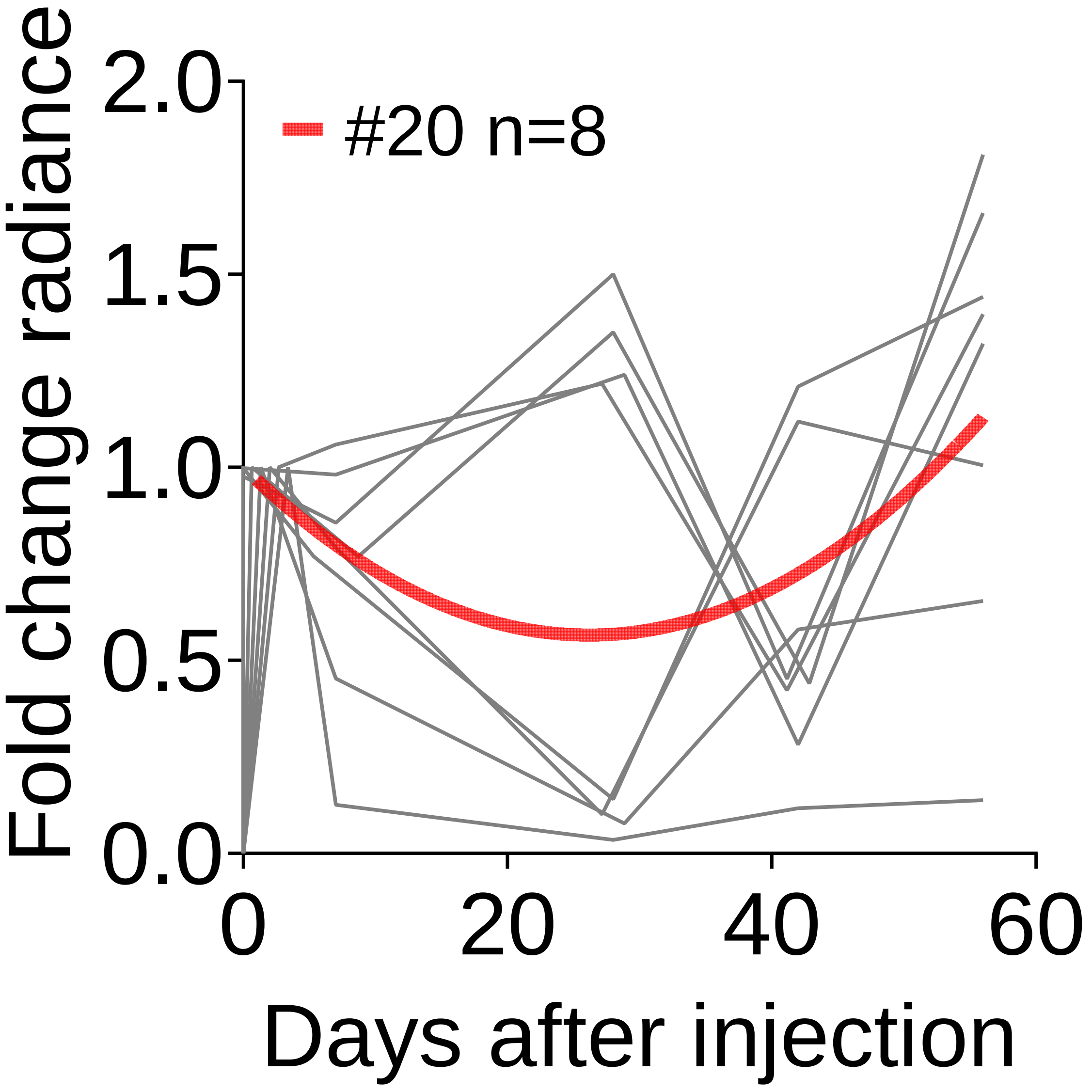

#20 n=8
Fold change radiance
2.0
1.5
1.0
0.5
0.0
0
20
40
60
Days after injection

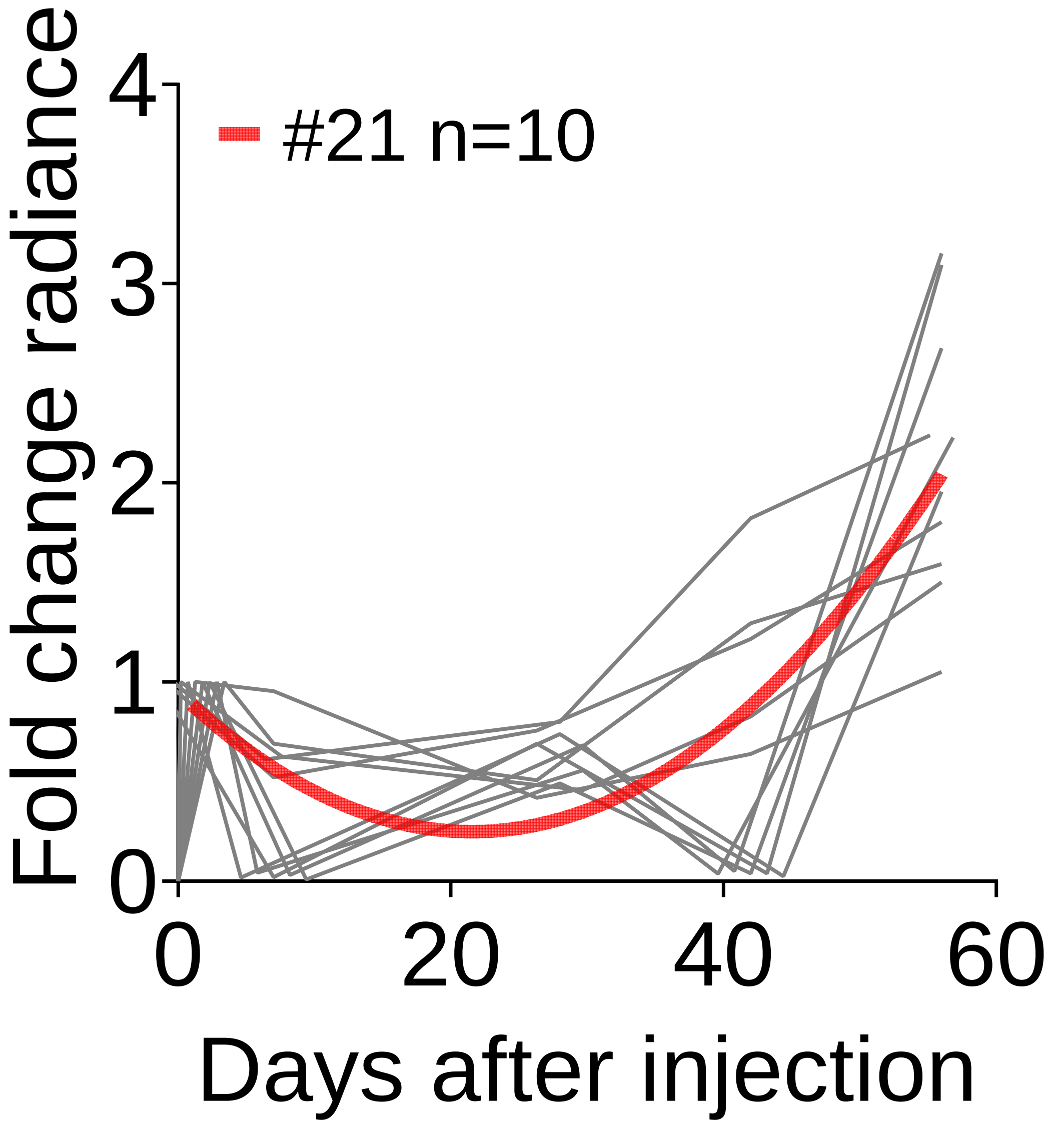

Fold change radiance
0
1
2
3
4
#21 n=10
0
20
40
60
Days after injection

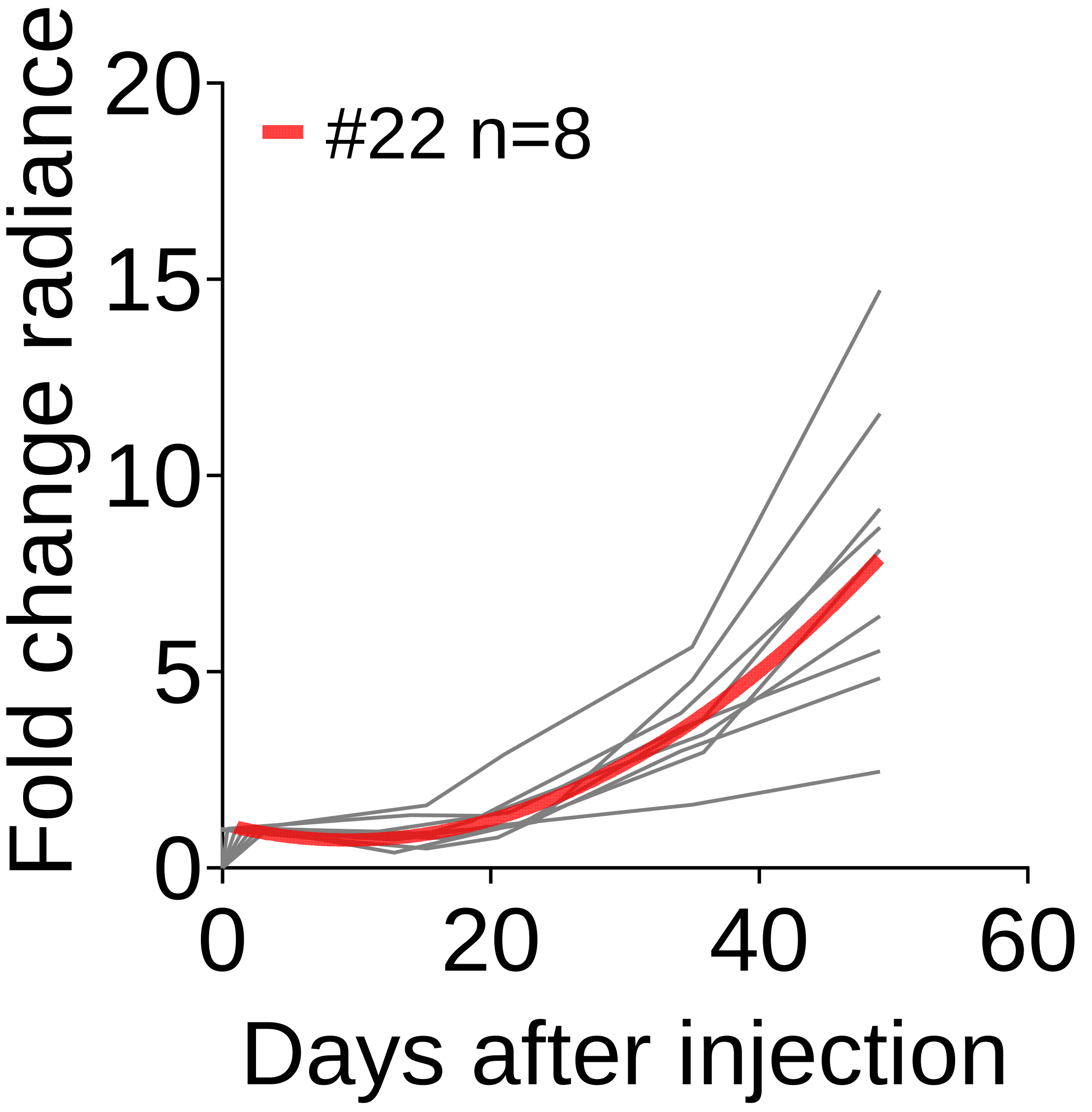

Fold change radiance
20
15
10
5
0
#22 n=8
0
20
40
60
Days after injection

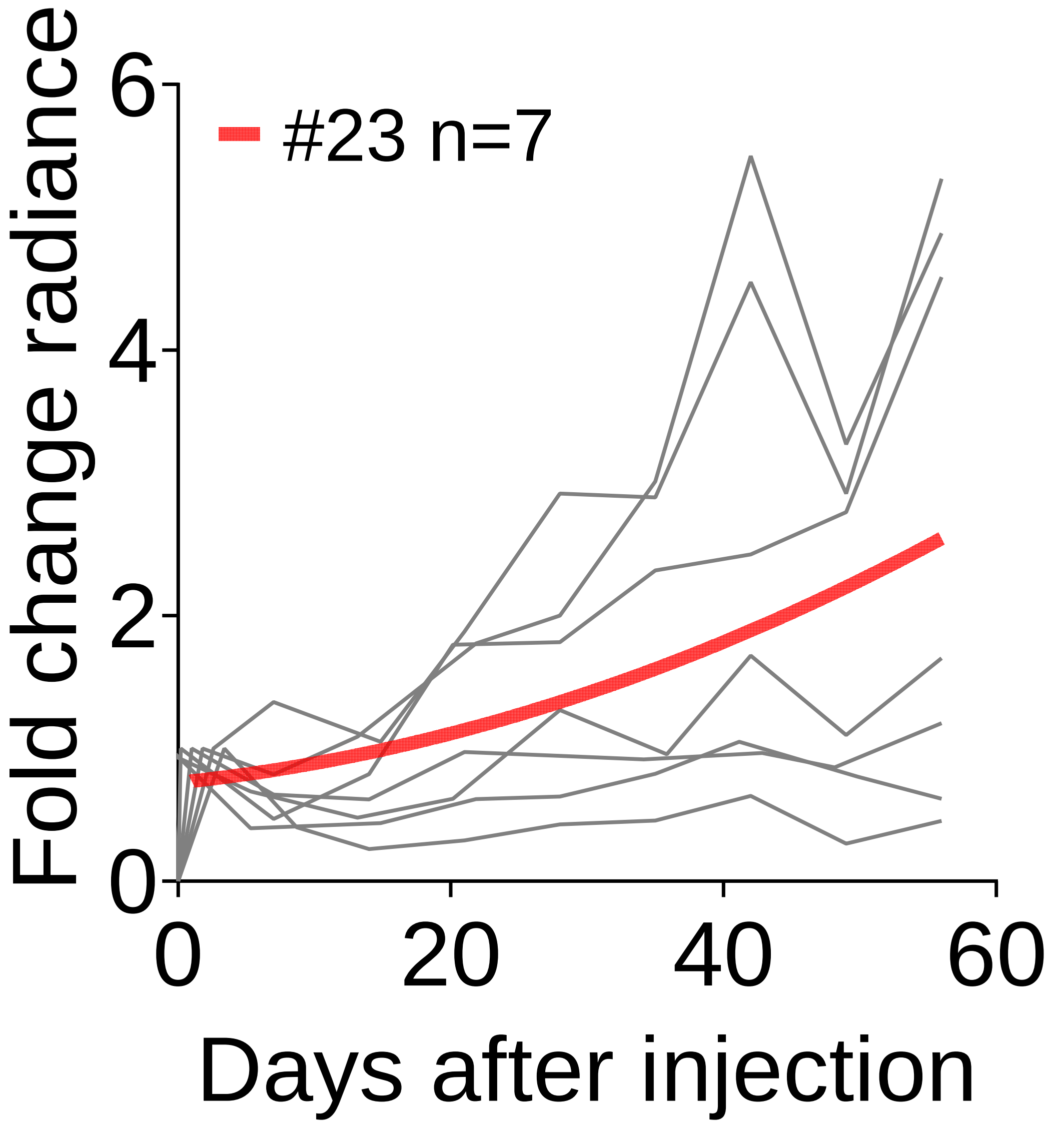
#23 n=7
Fold change radiance
0
2
4
6
0
20
40
60
Days after injection

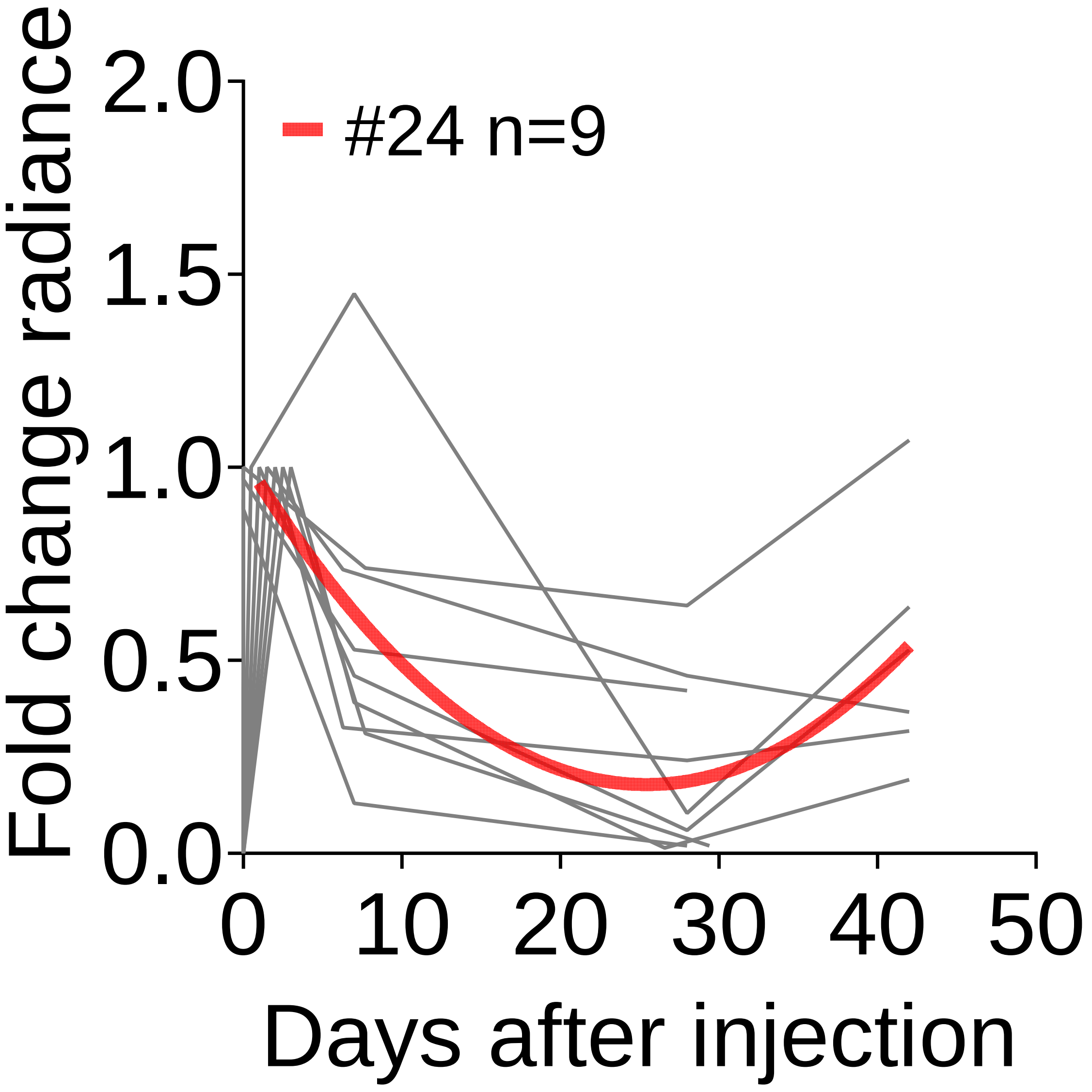

Fold change radiance
2.0
1.5
1.0
0.5
0.0
#24 n=9
0
10
20
30
40
50
Days after injection

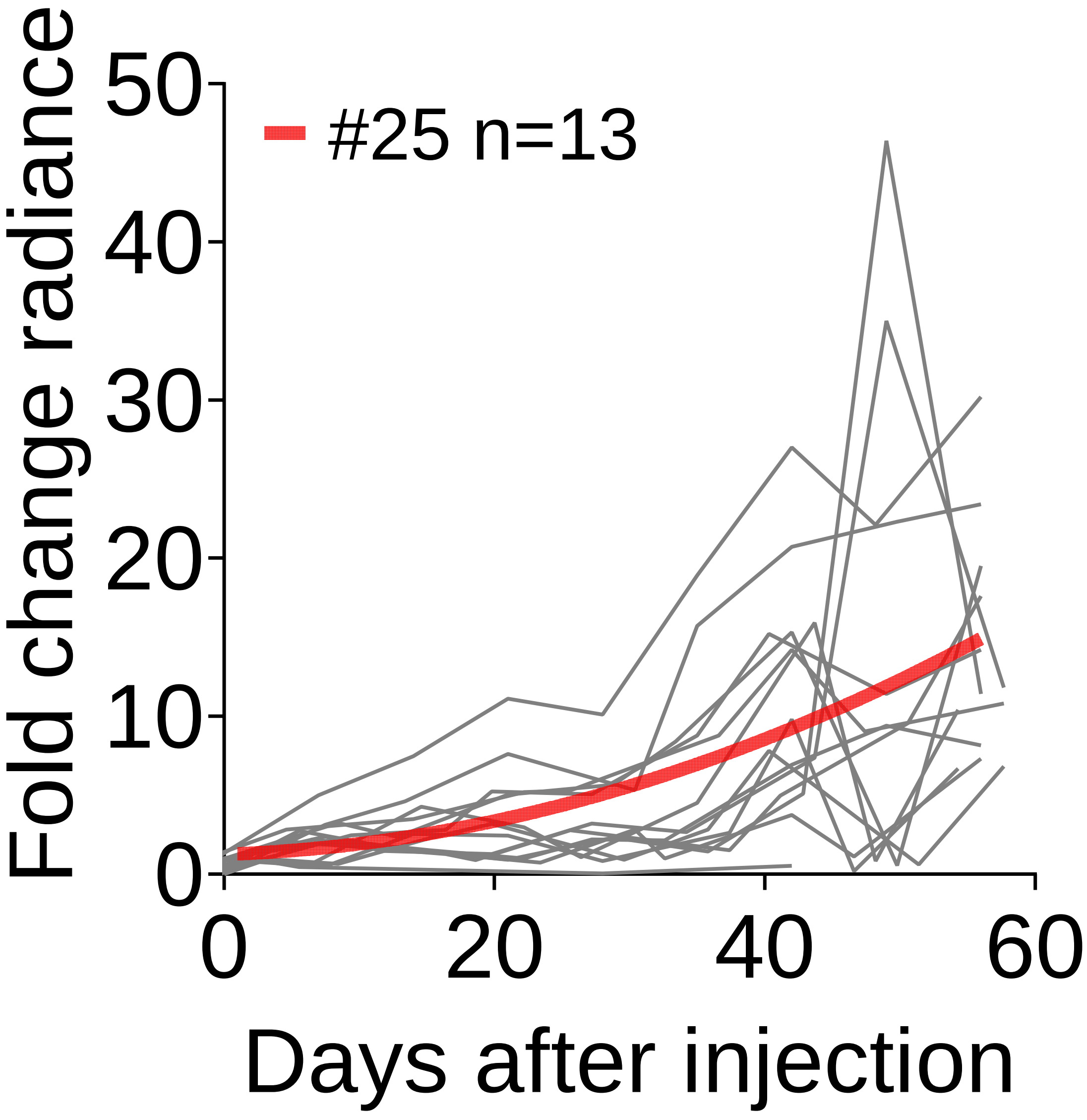

Fold change radiance
50
40
30
20
10
0
#25 n=13
0
20
40
60
Days after injection

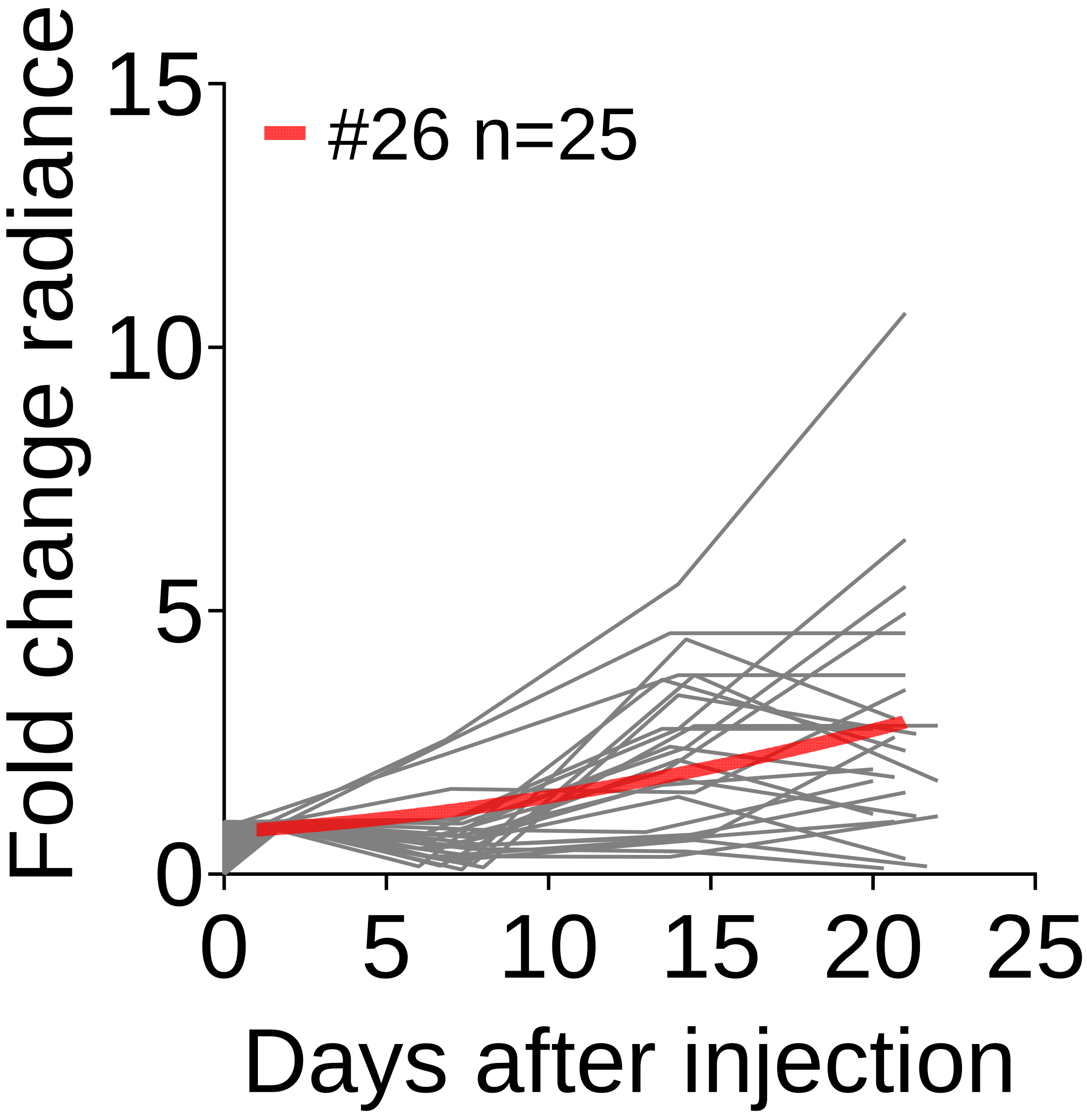

Fold change radiance
15
10
5
0
#26 n=25
0
5
10
15
20
25
Days after injection

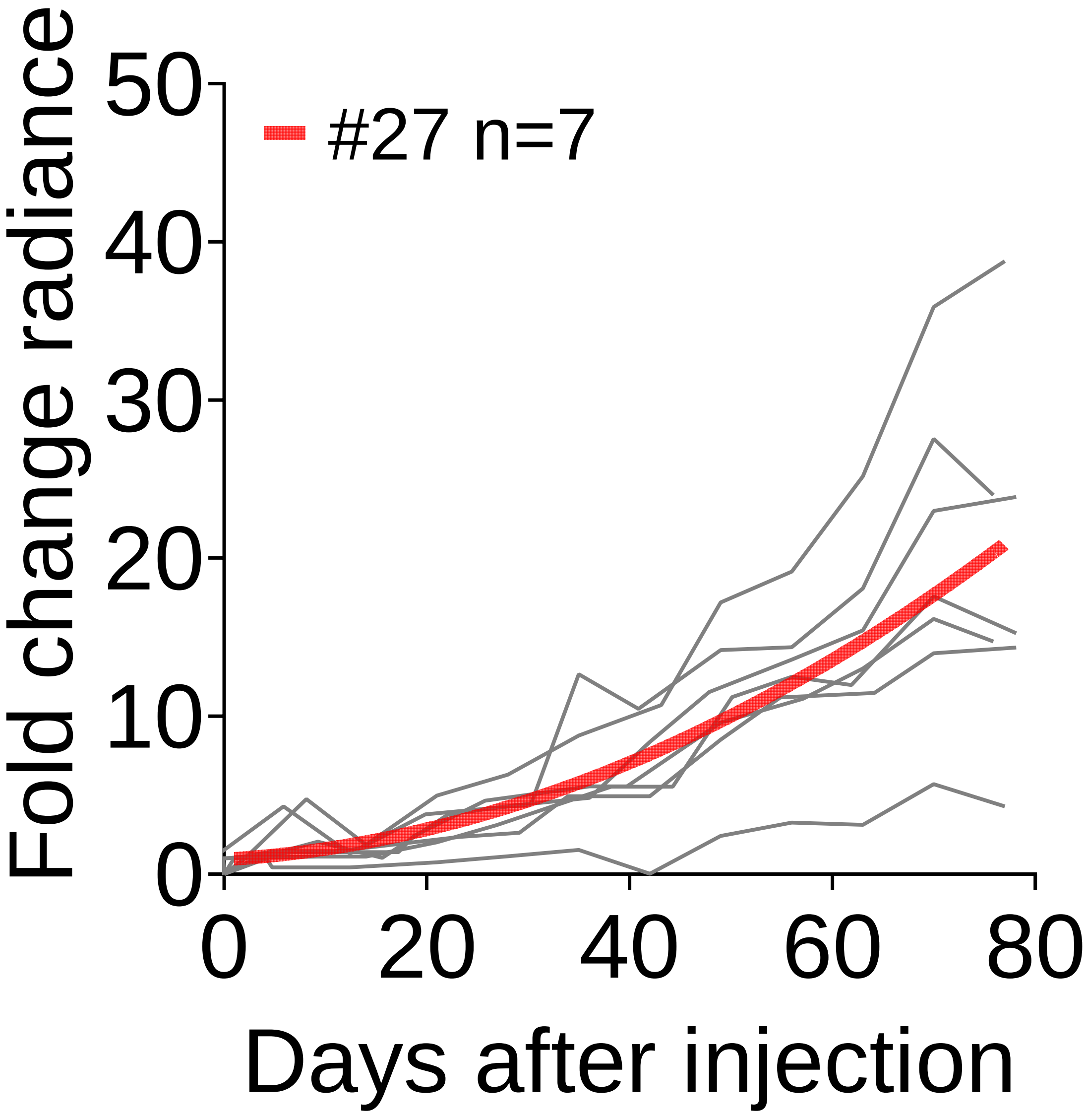

Fold change radiance
50
40
30
20
10
0
#27 n=7
0
20
40
60
80
Days after injection

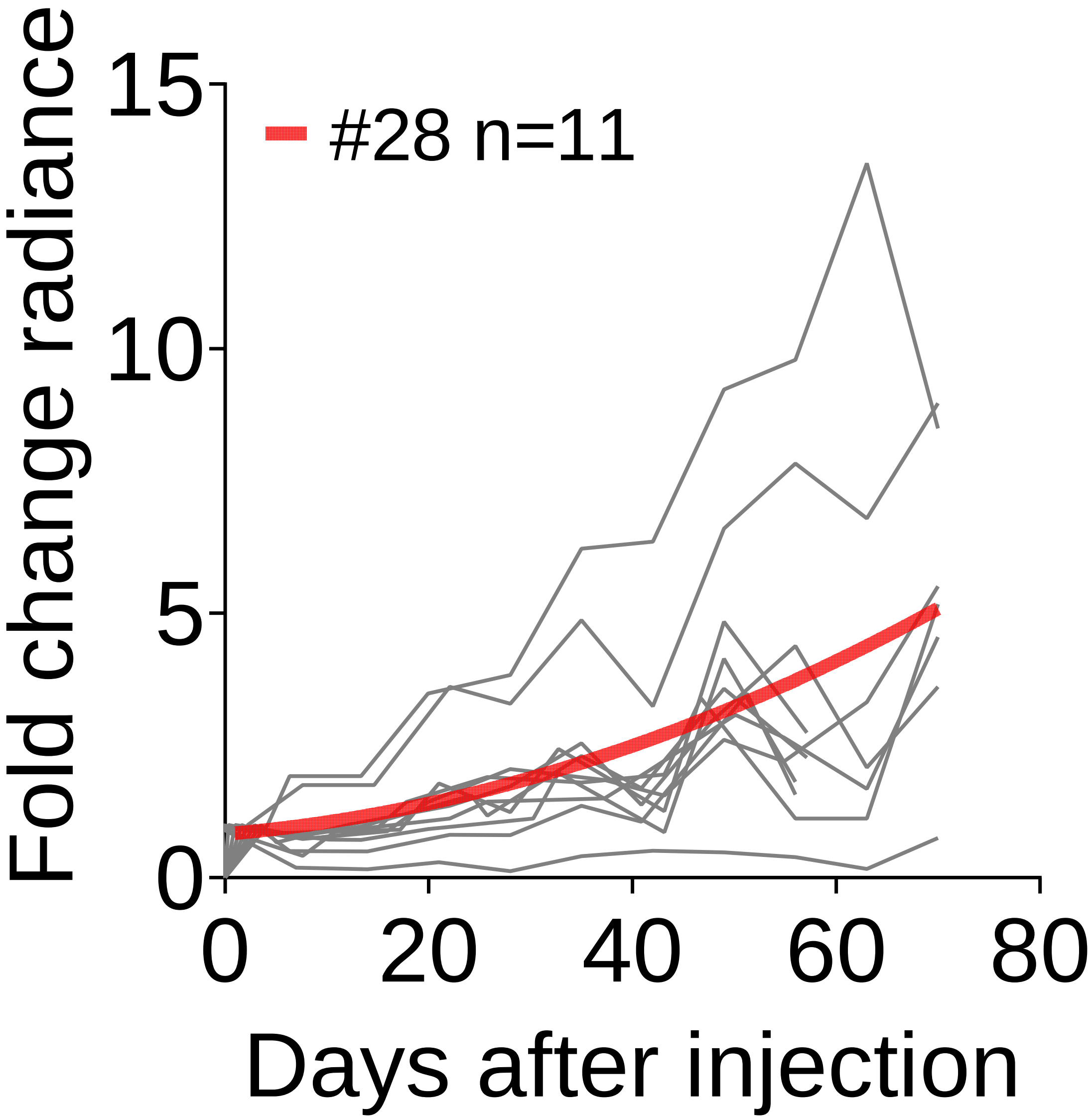

Fold change radiance
15
10
5
0
#28 n=11
0
20
40
60
80
Days after injection

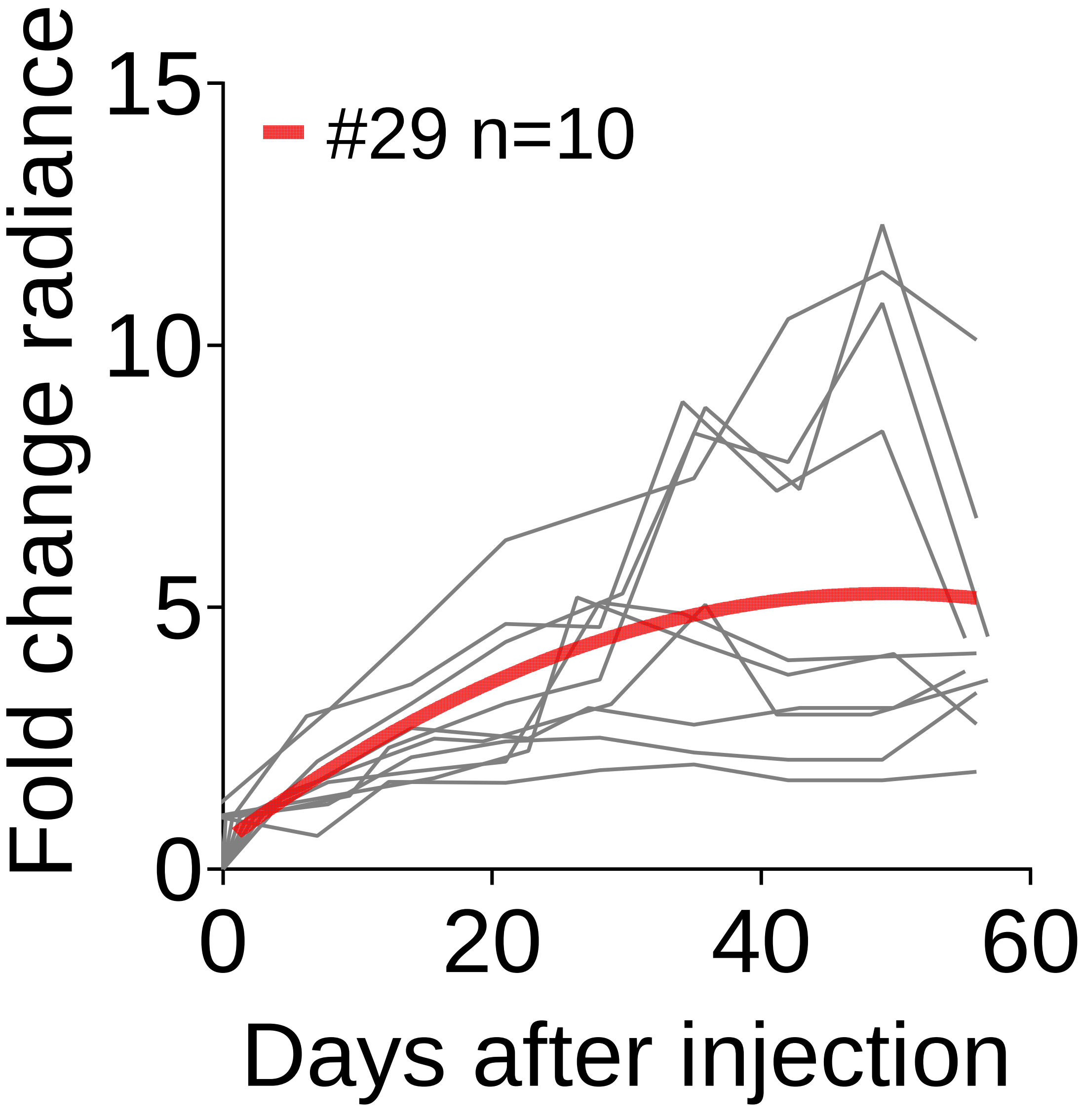
Fold change radiance
15
10
5
0
#29 n=10
0
20
40
60
Days after injection

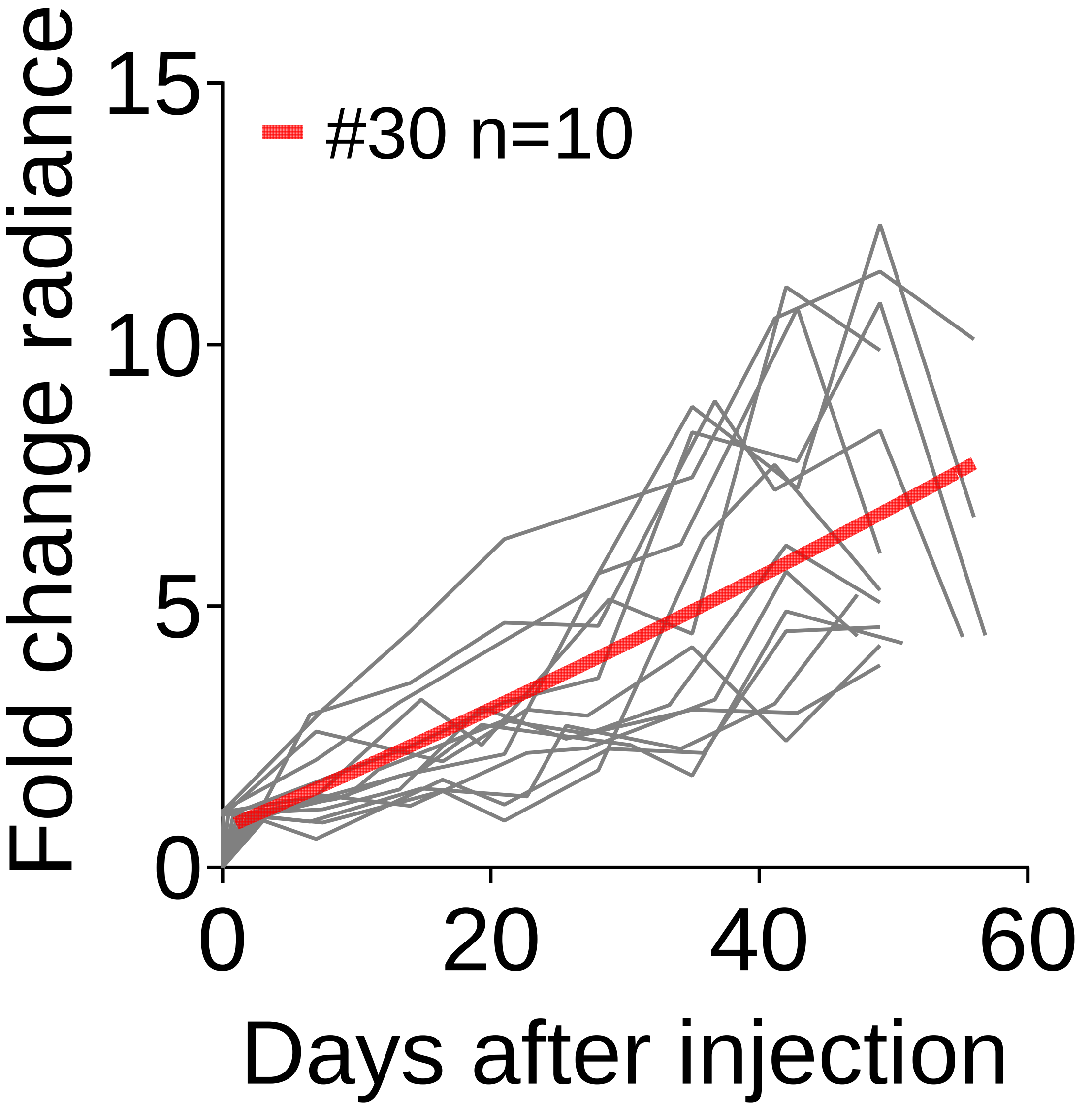
Fold change radiance
15
10
5
0
#30 n=10
0
20
40
60
Days after injection

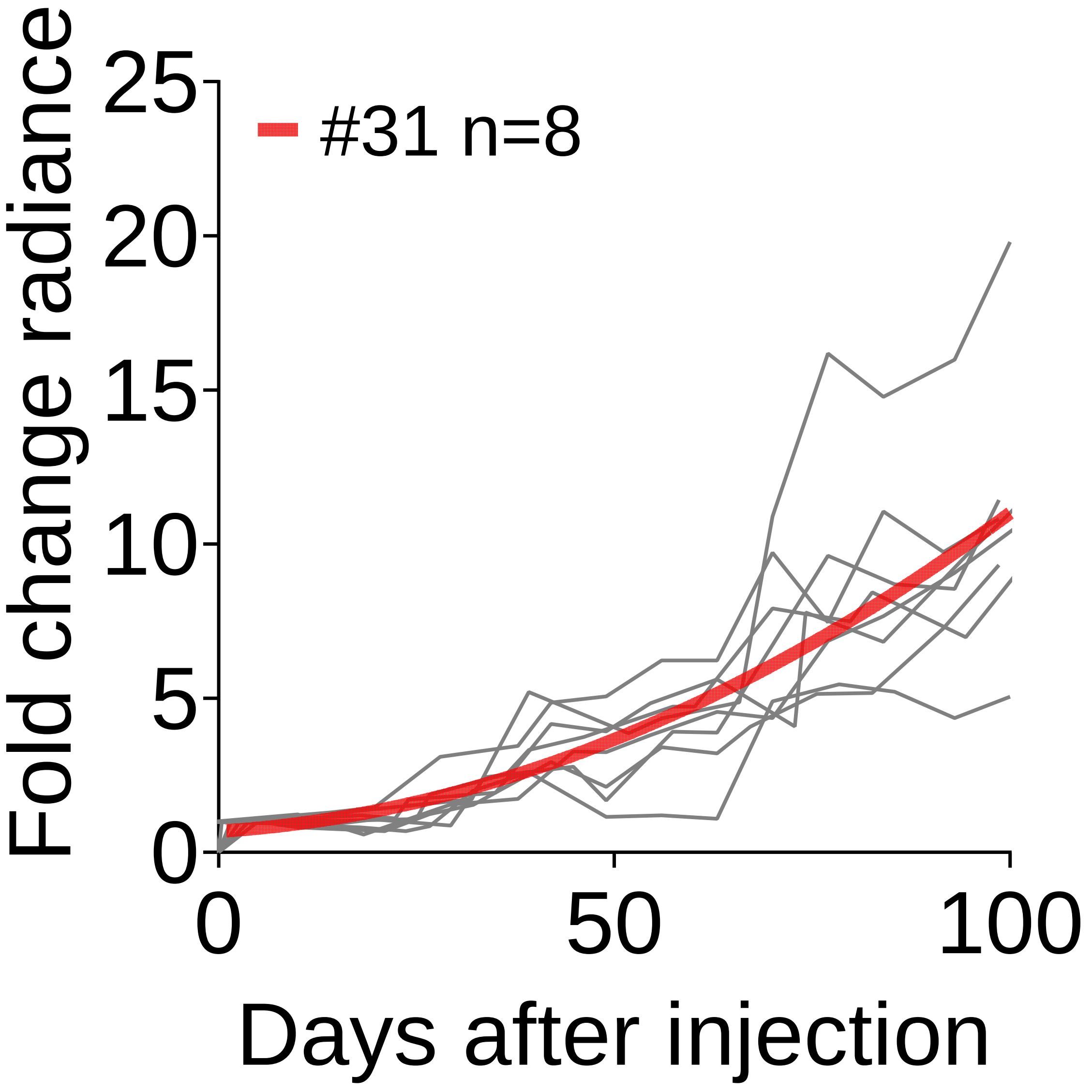

Fold change radiance
25
20
15
10
5
0
#31 n=8
0
50
100
Days after injection

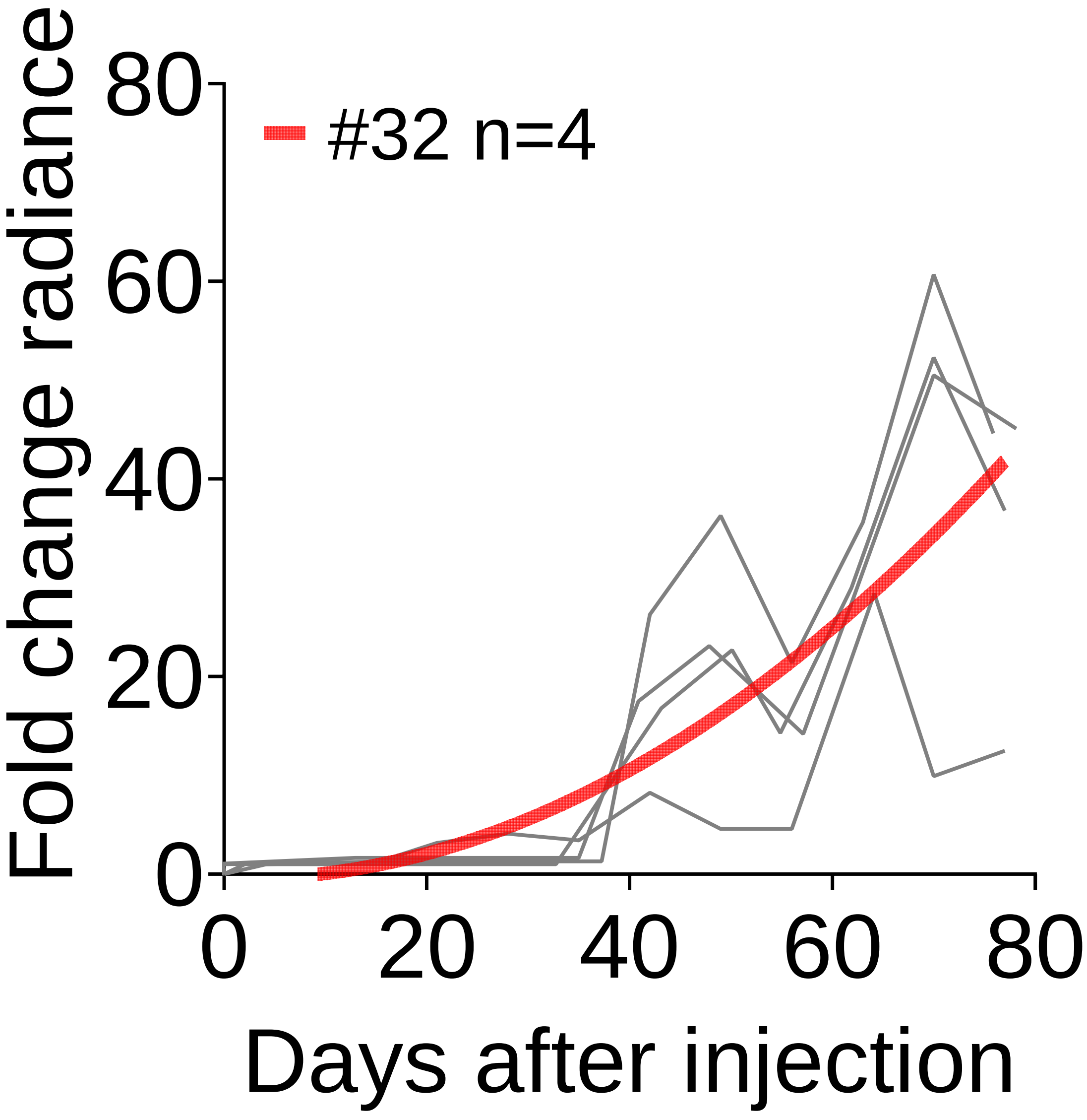
#32 n=4
Fold change radiance
0
20
40
60
80
Days after injection
0
20
40
60
80

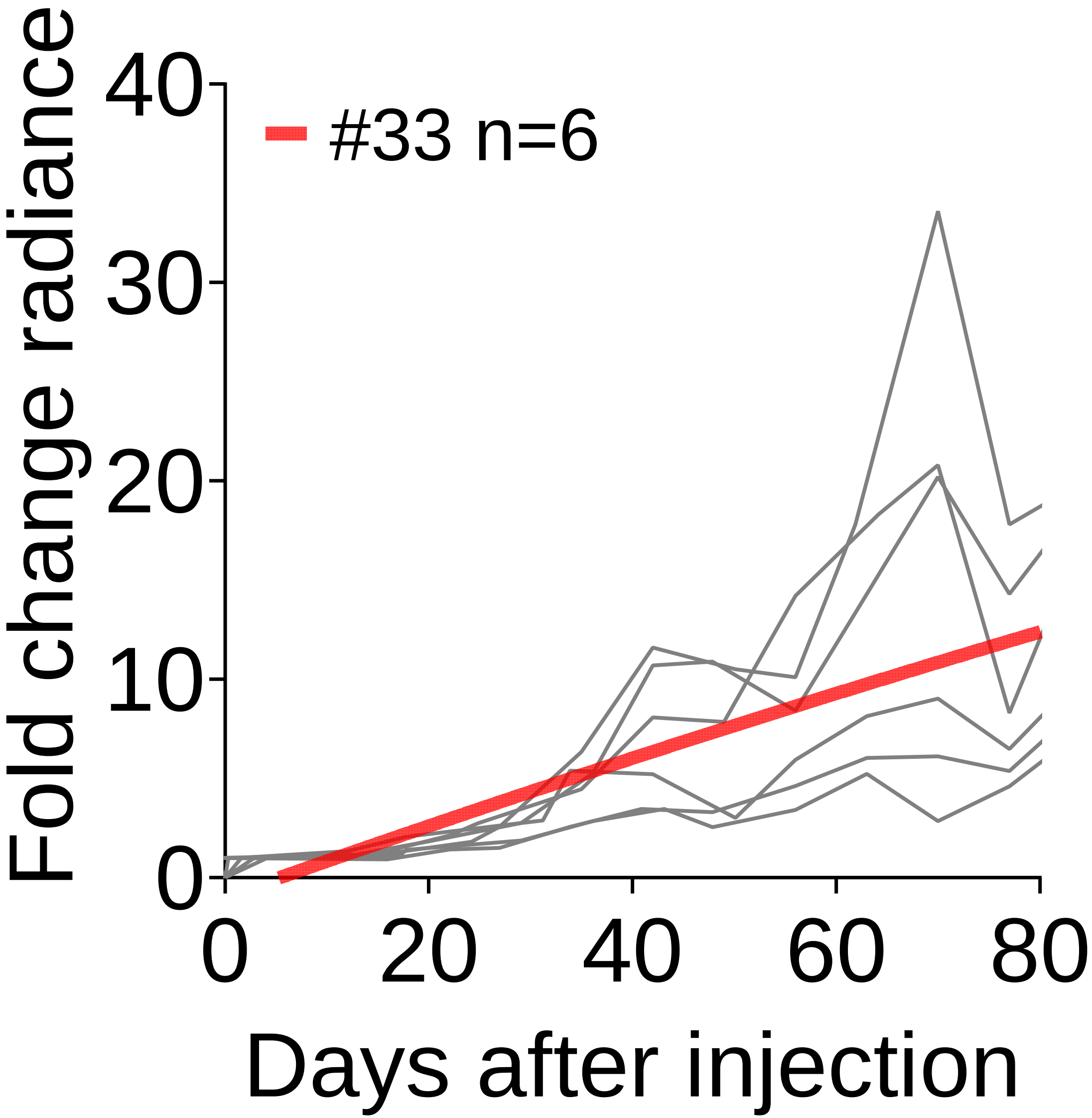

Fold change radiance
40
30
20
10
0
#33 n=6
0
20
40
60
80
Days after injection

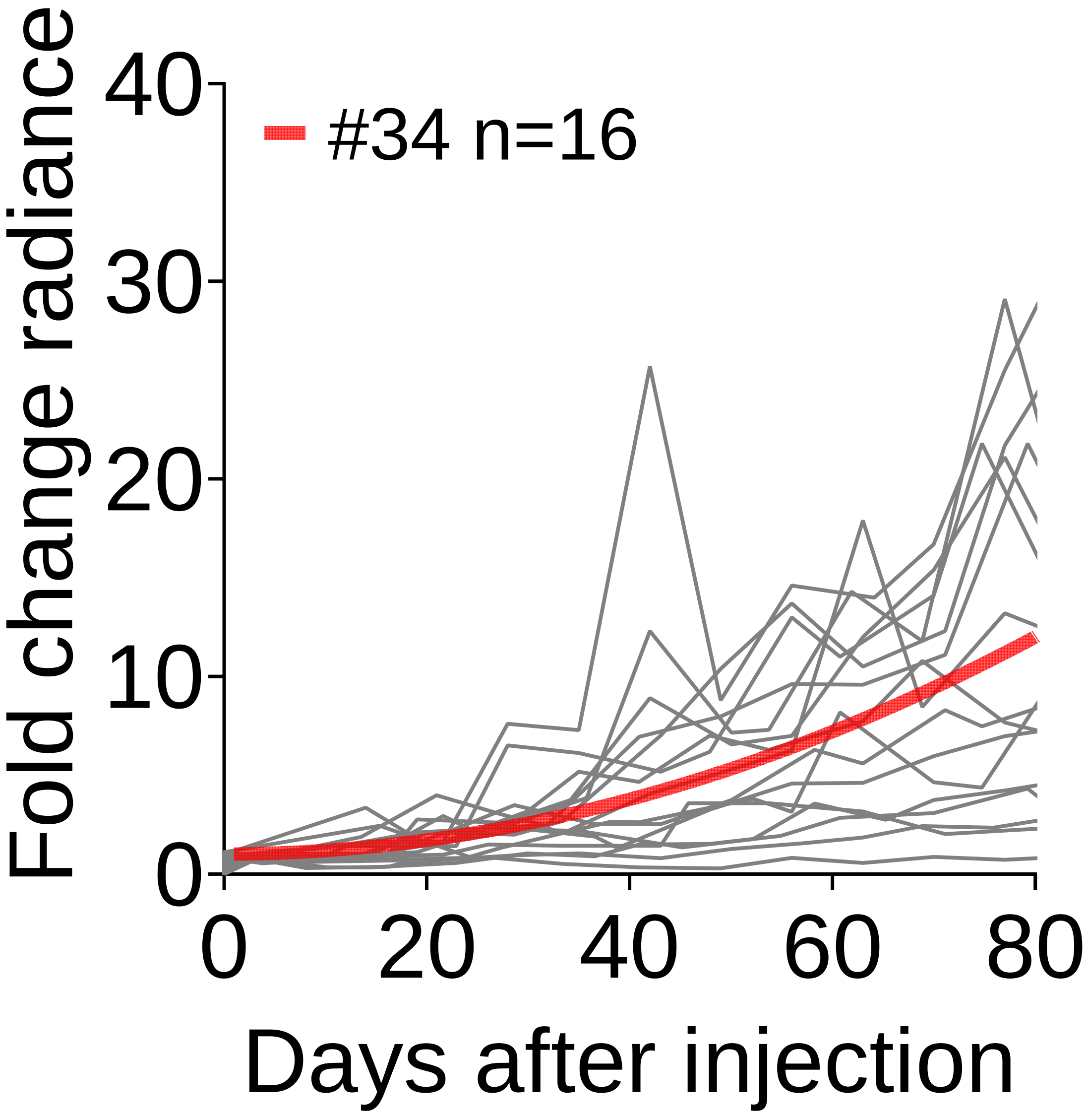

Fold change radiance
40
30
20
10
0
#34 n=16
0
20
40
60
80
Days after injection

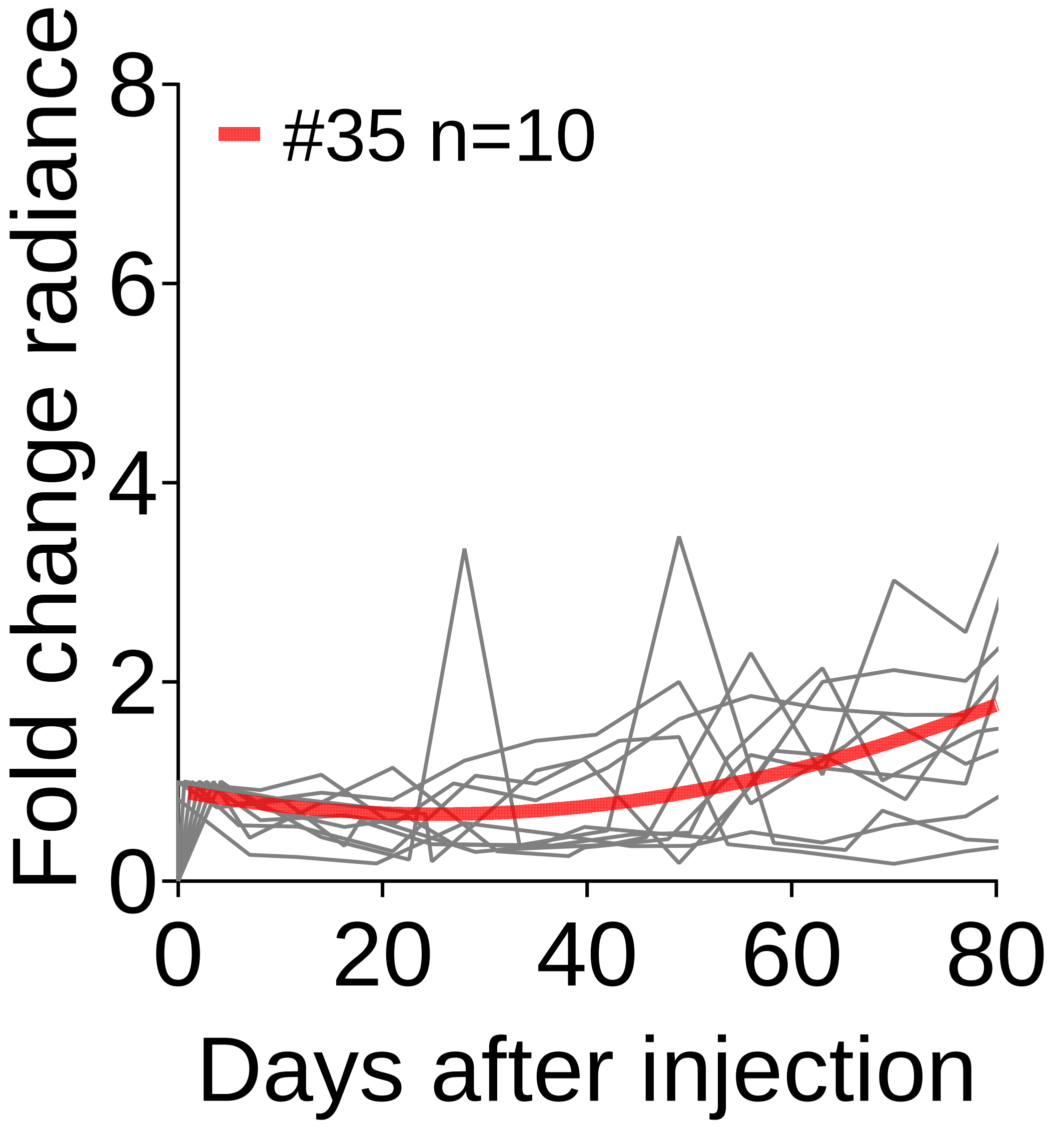

Fold change radiance
8
6
4
2
0
#35 n=10
0
20
40
60
80
Days after injection

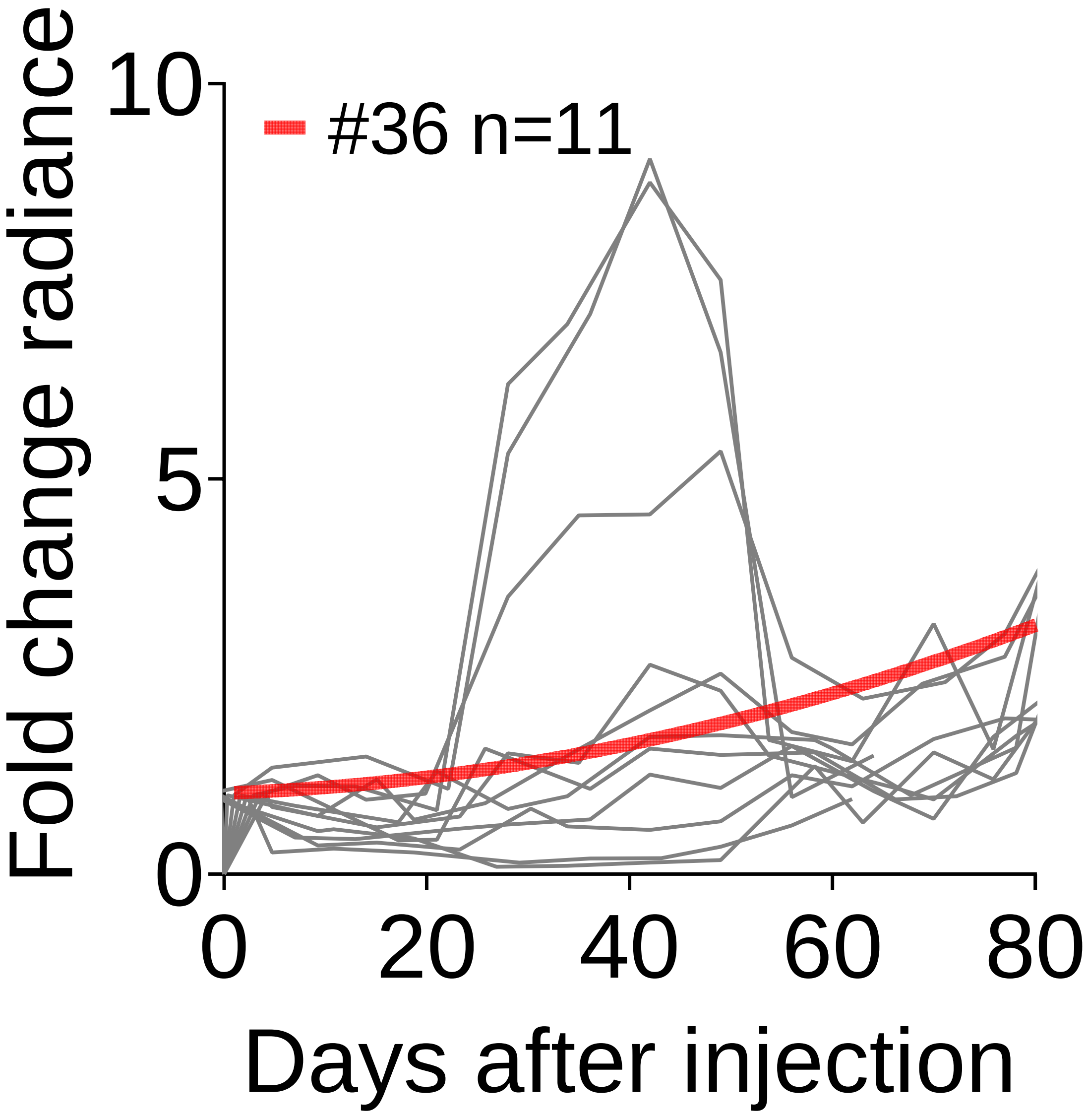

Fold change radiance
10
5
0
#36 n=11
0
20
40
60
80
Days after injection

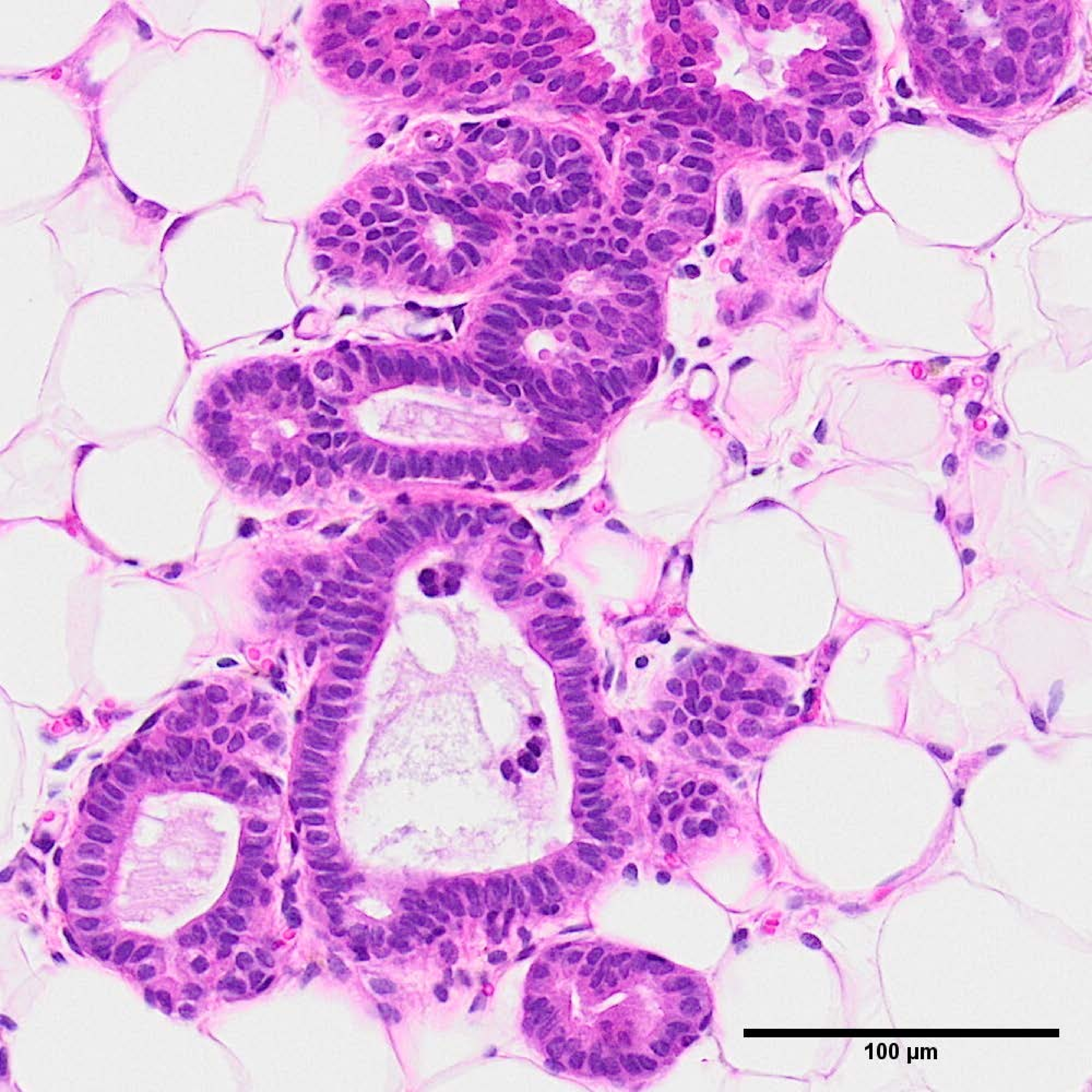
100 µm

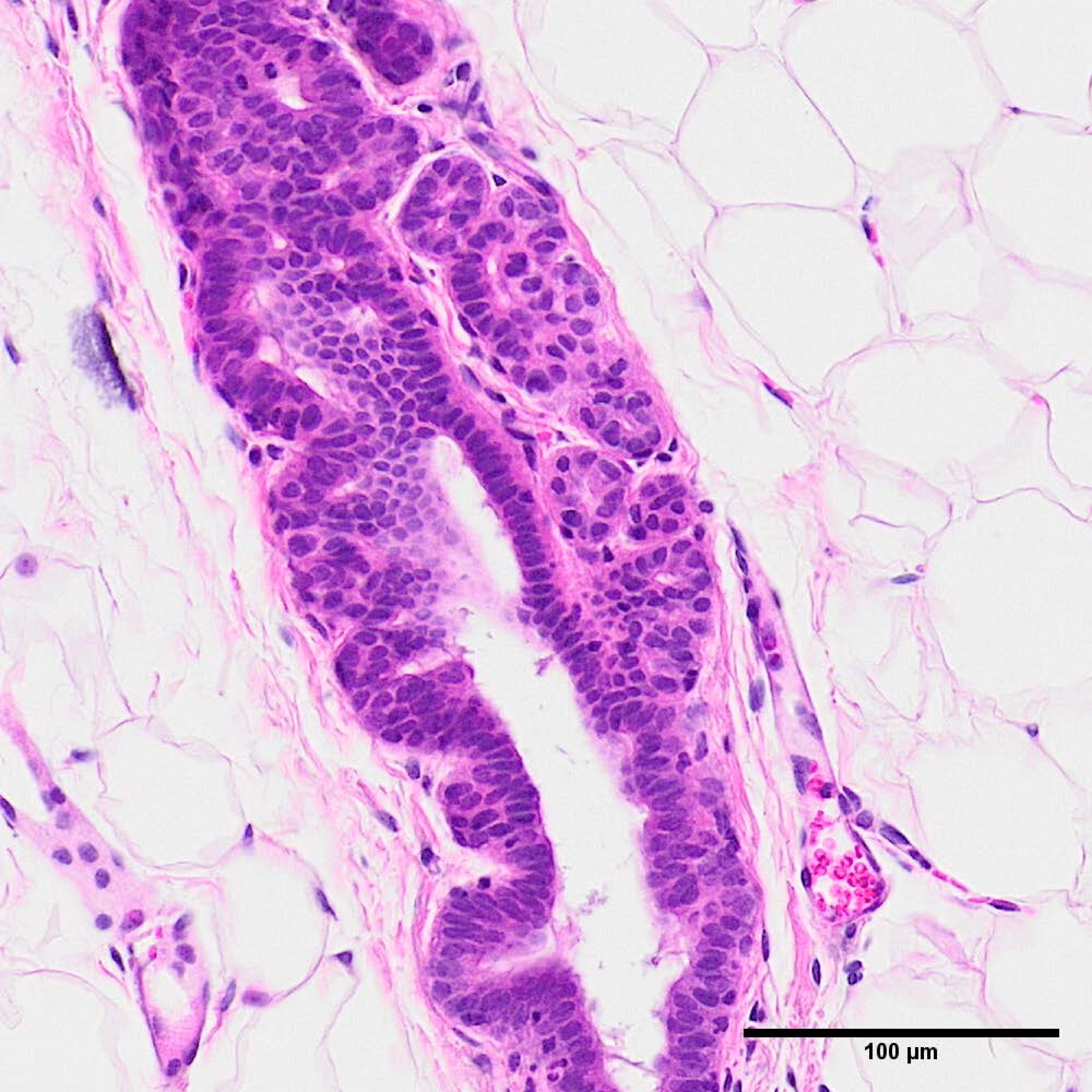
100 µm

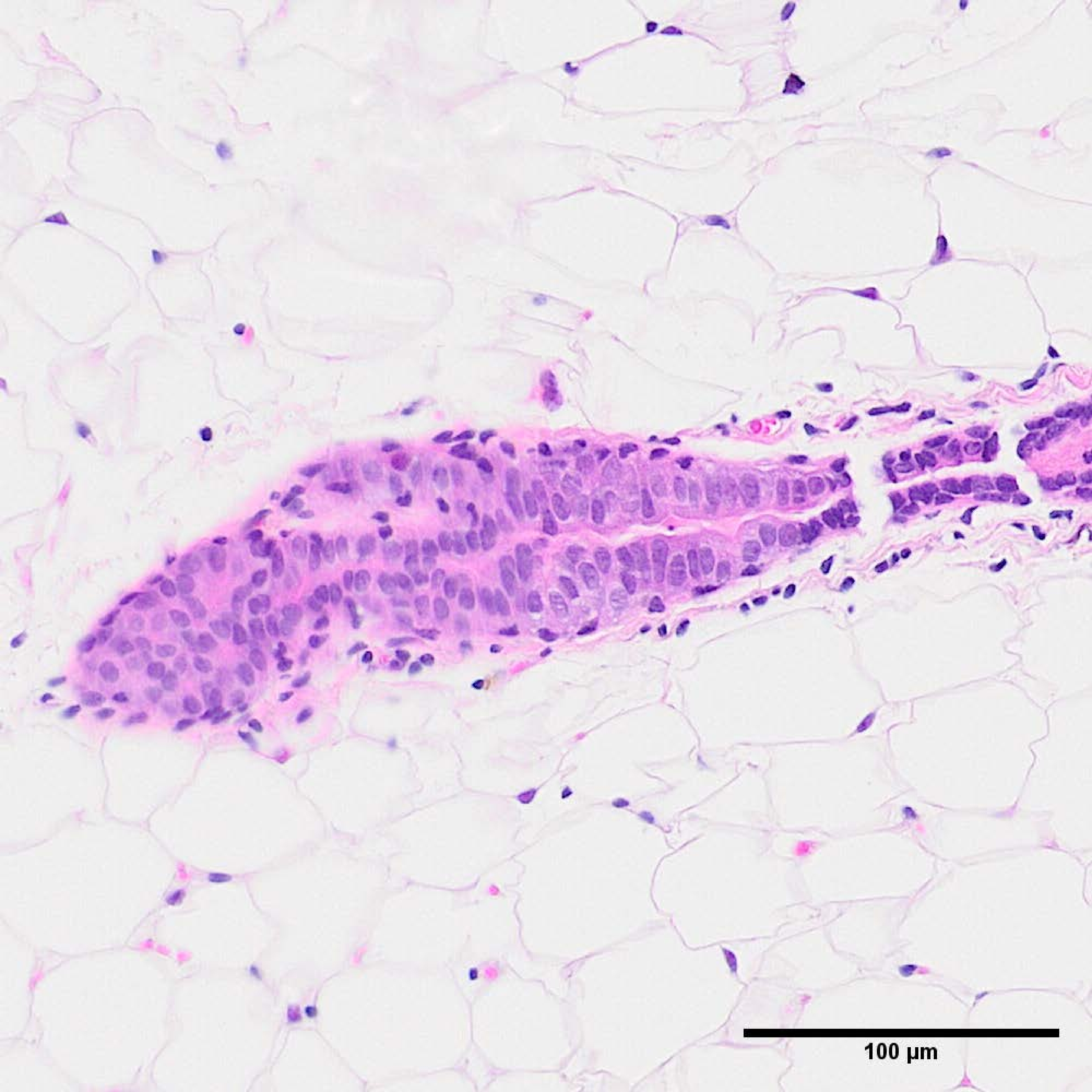
100 µm

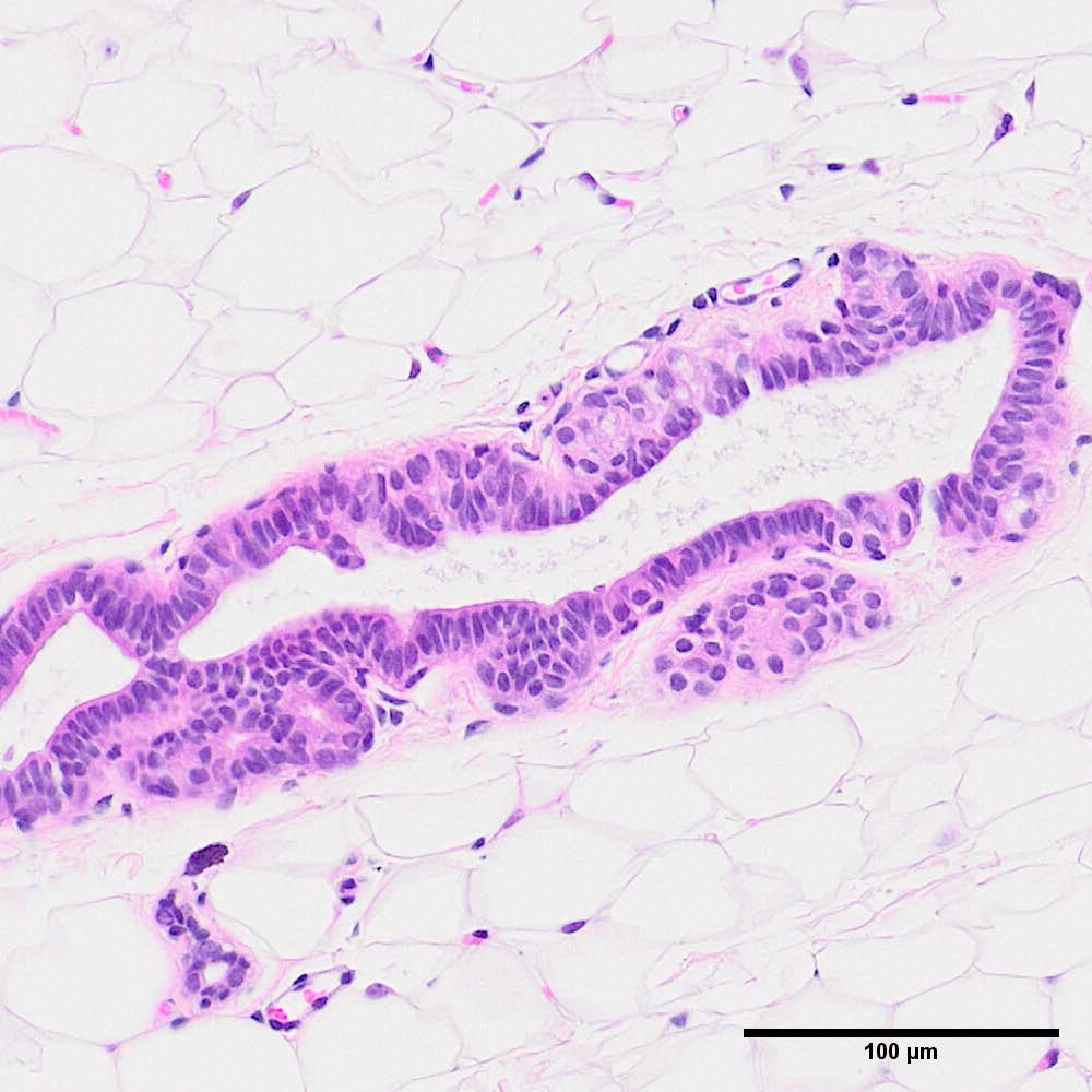
100 µm

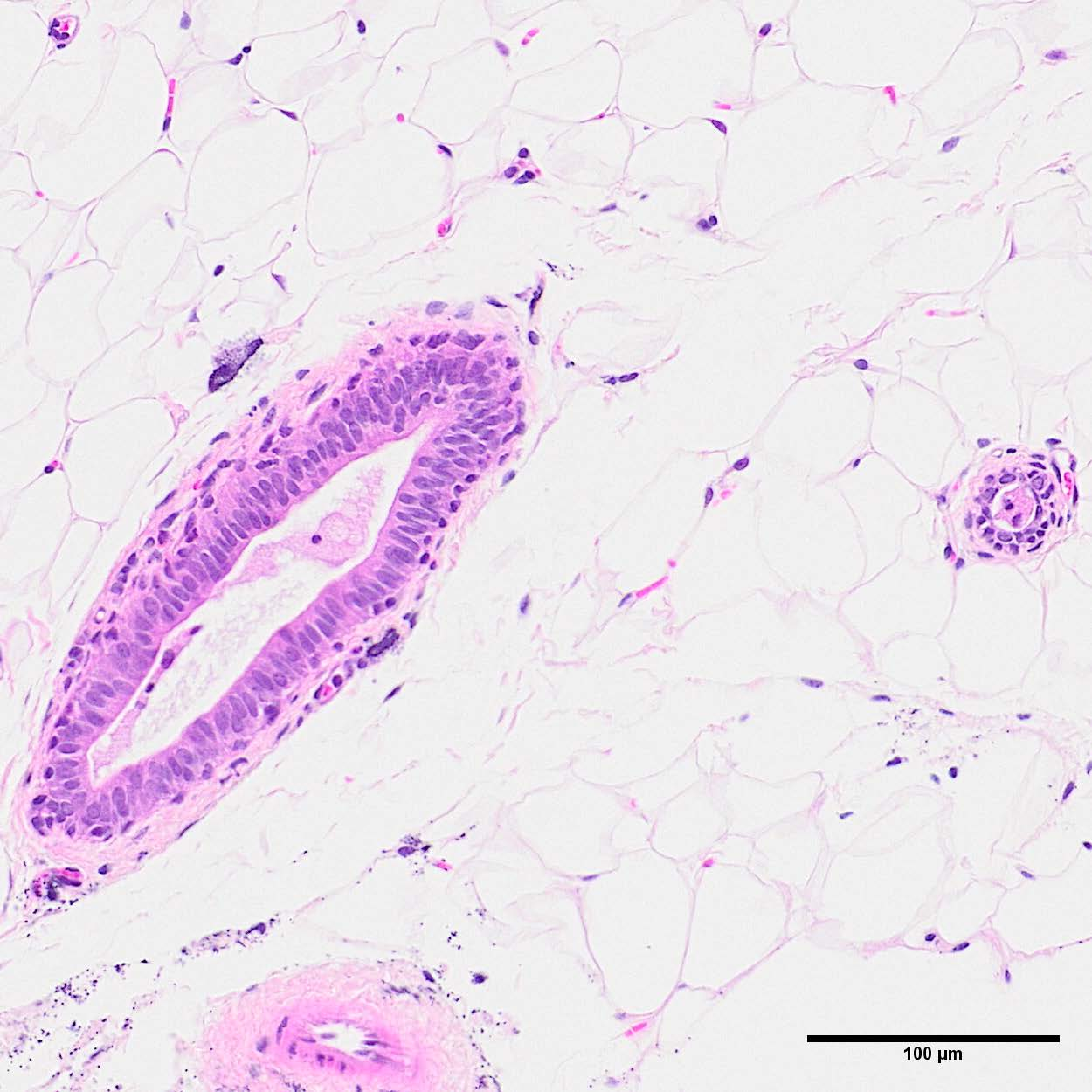
100 µm

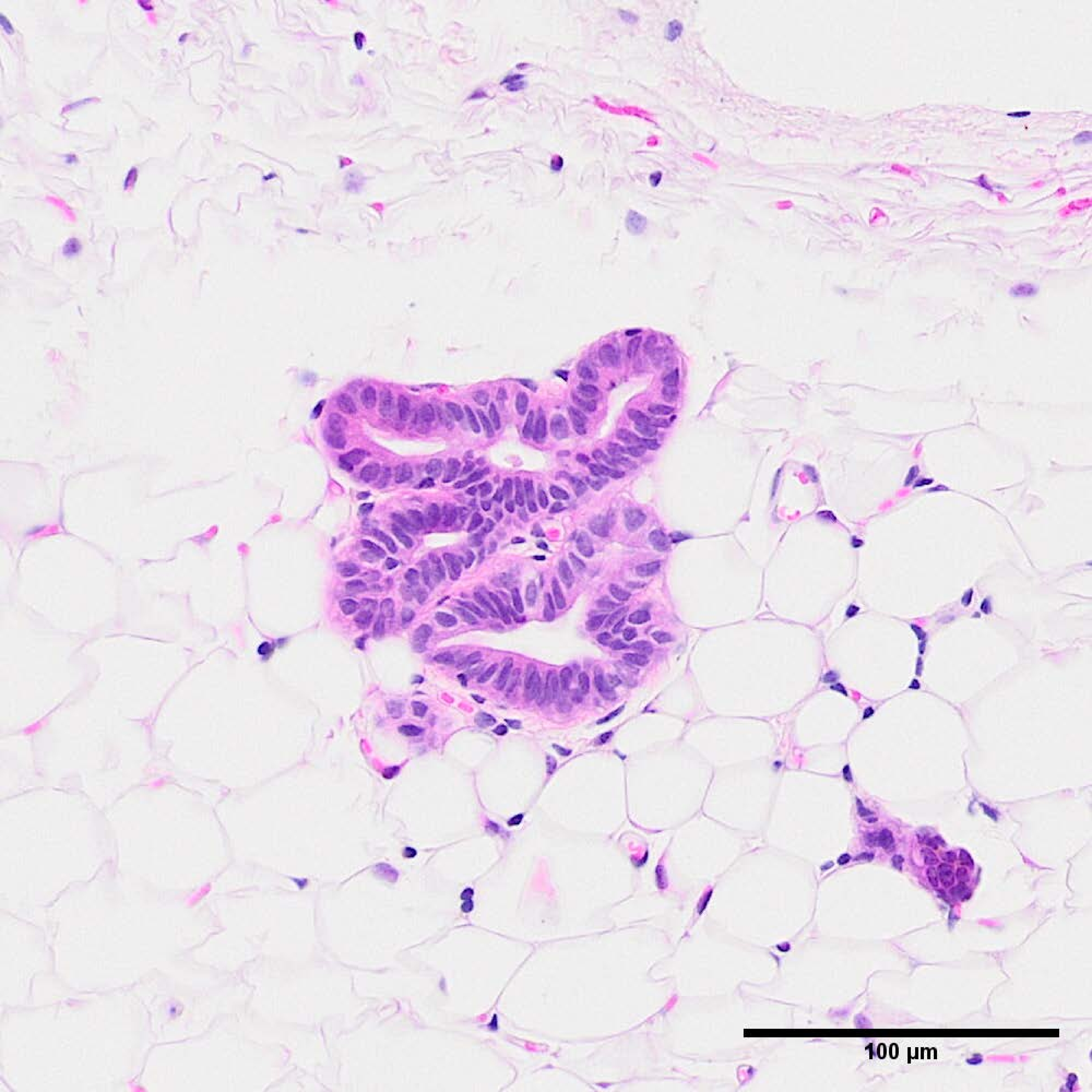
100 µm

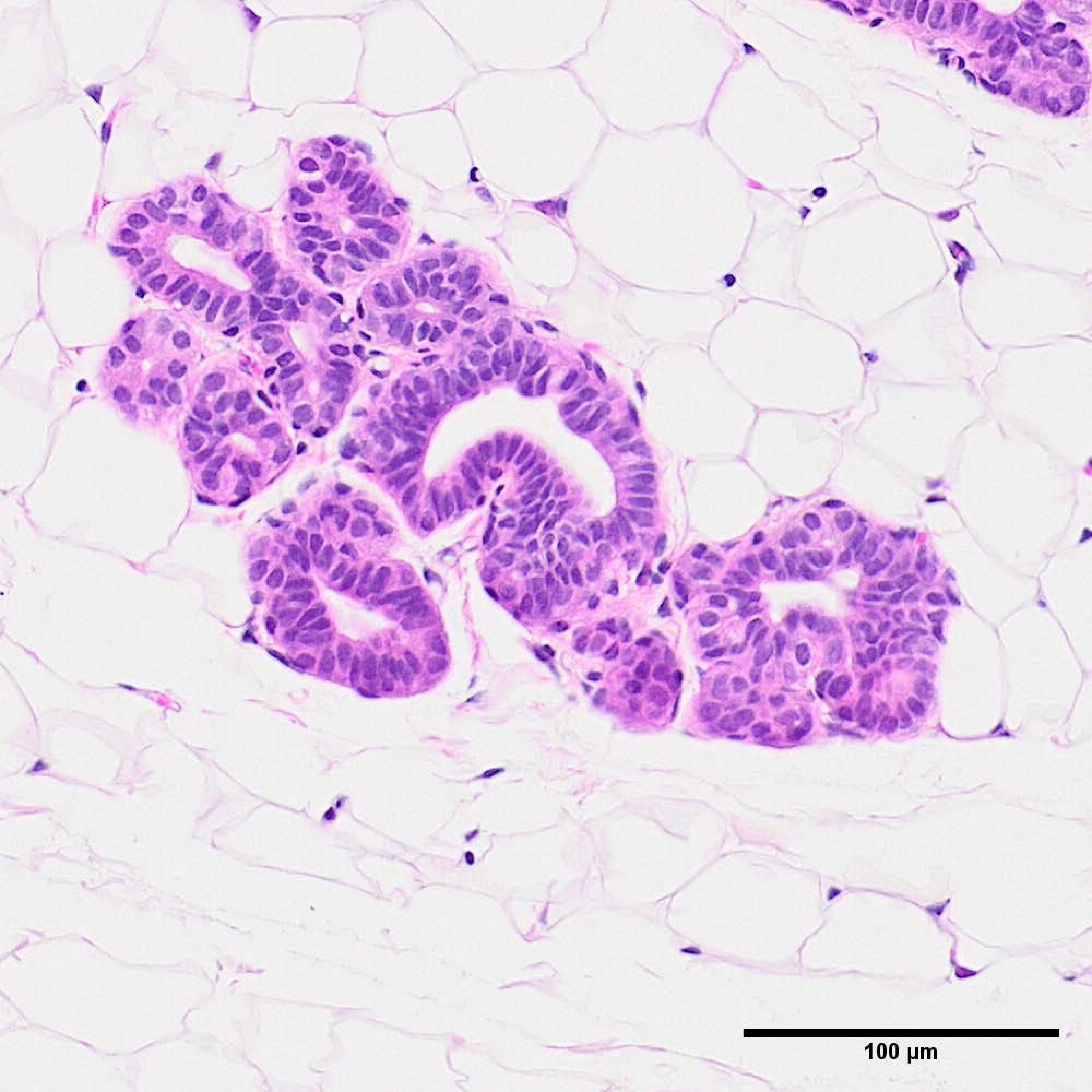
100 µm

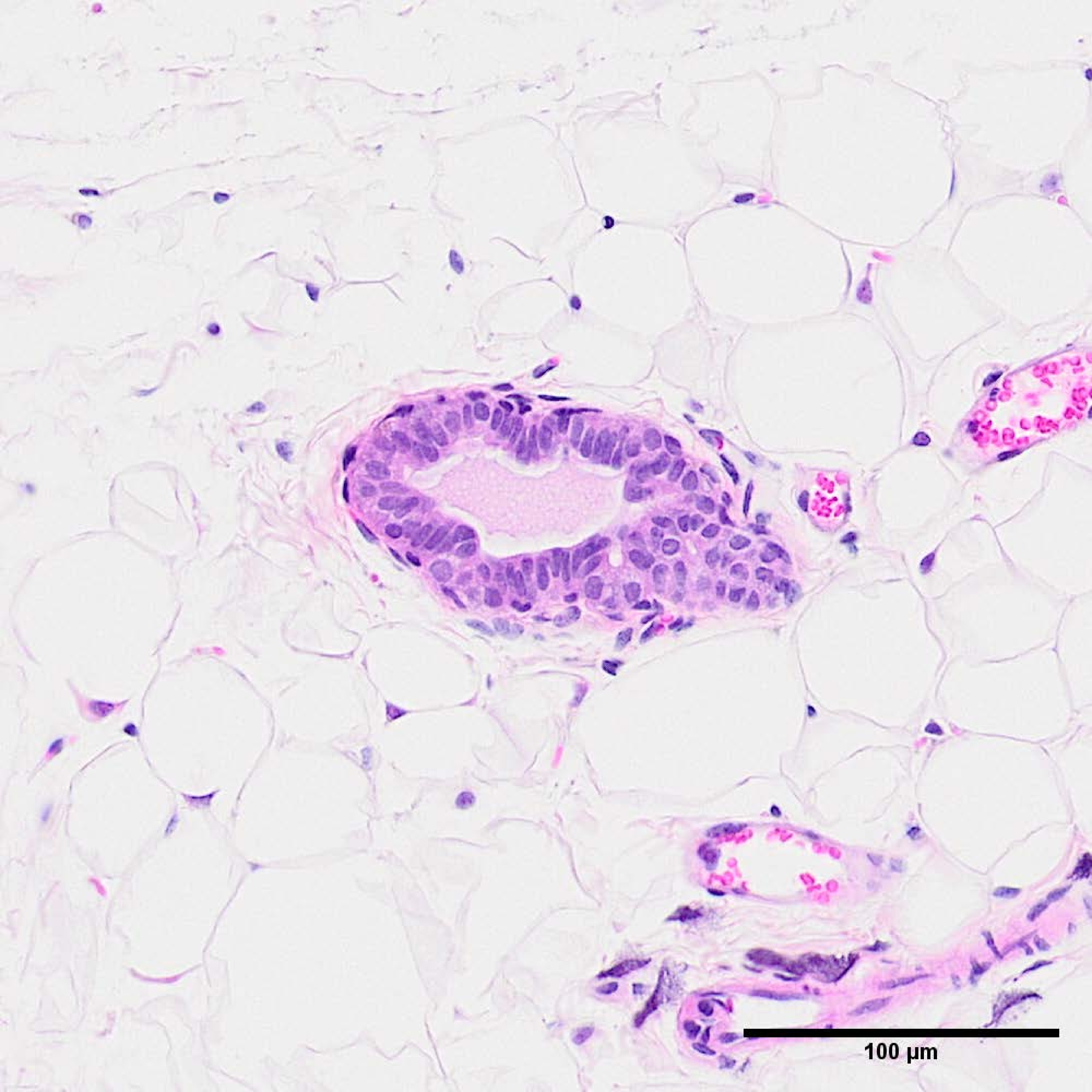
100 µm

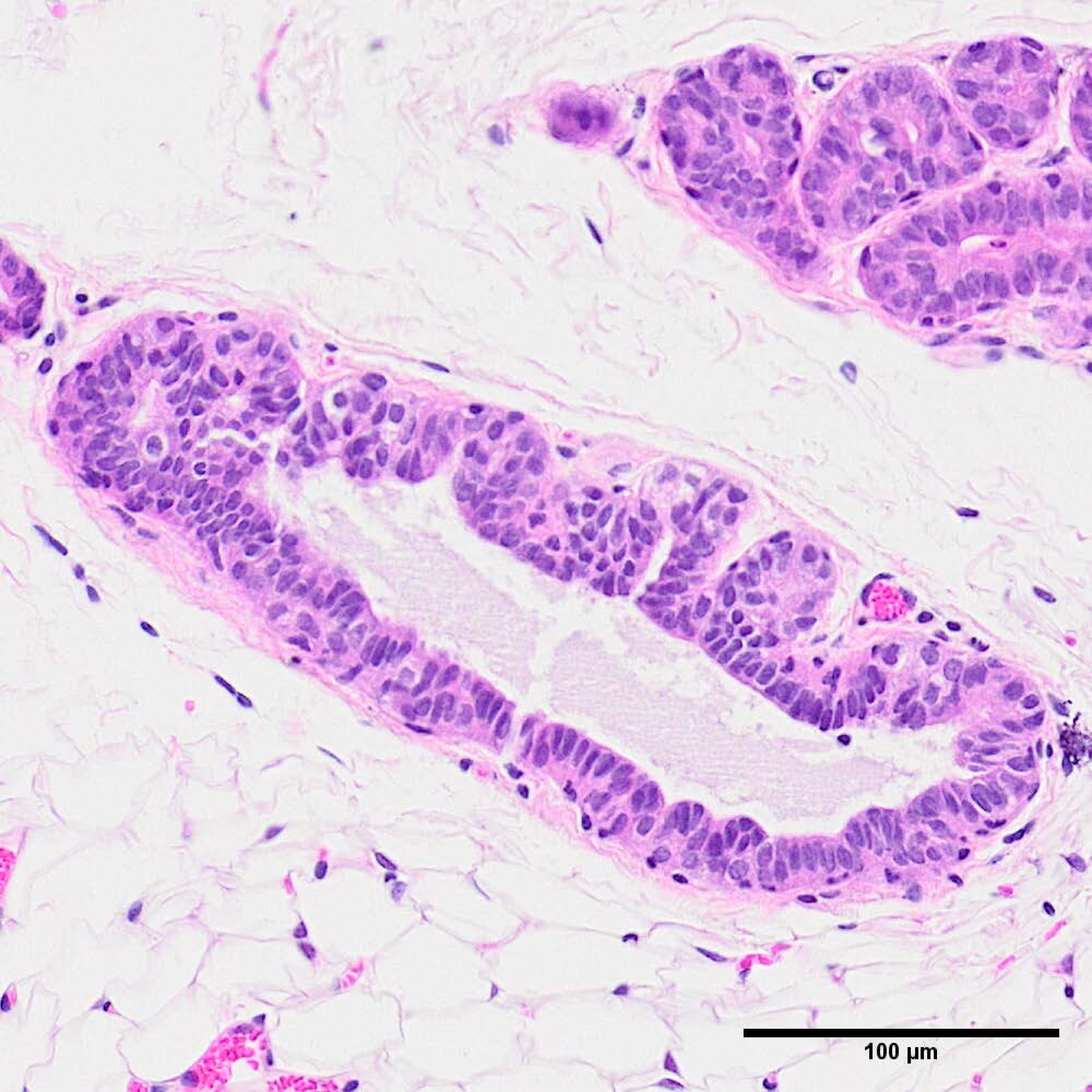
100 µm

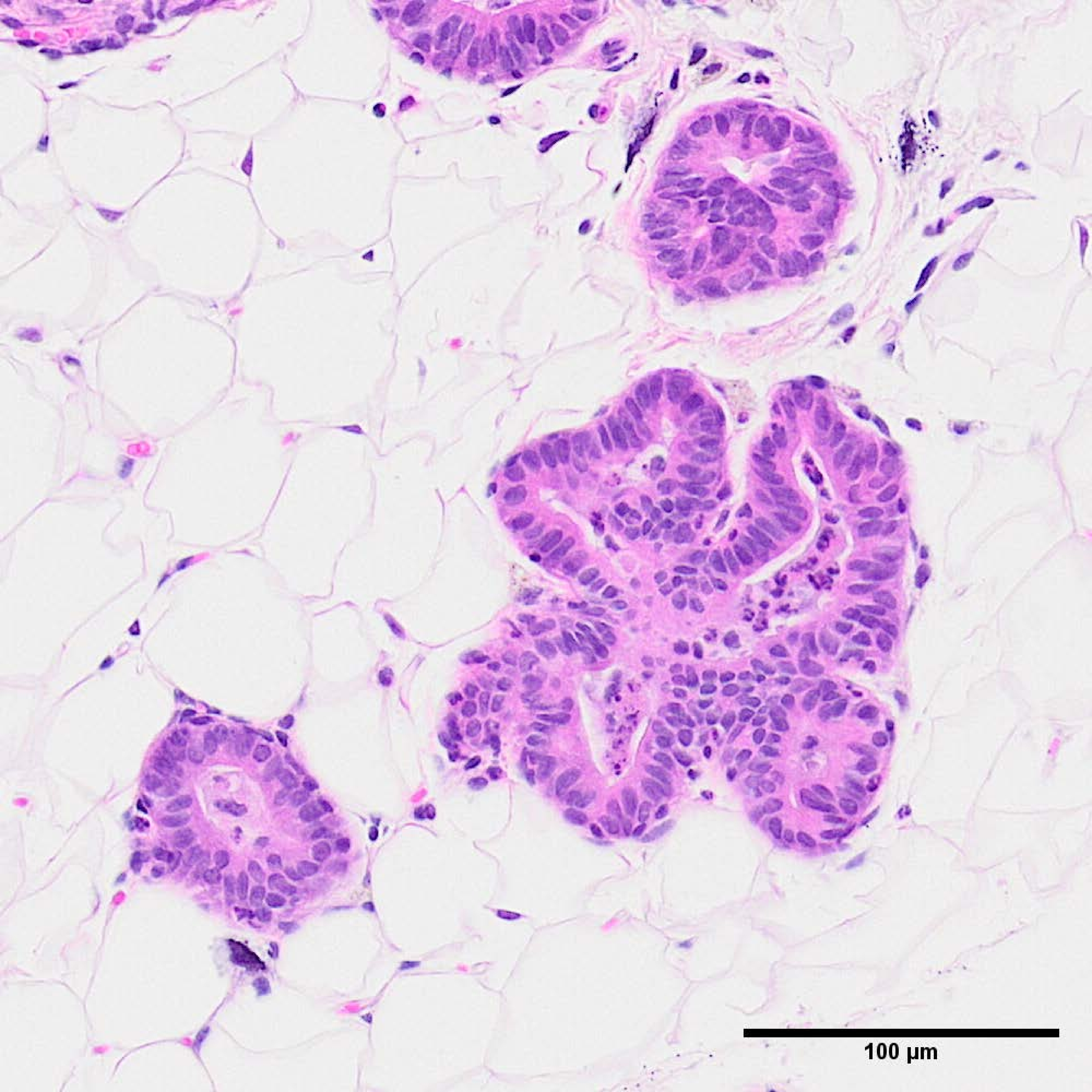
100 µm

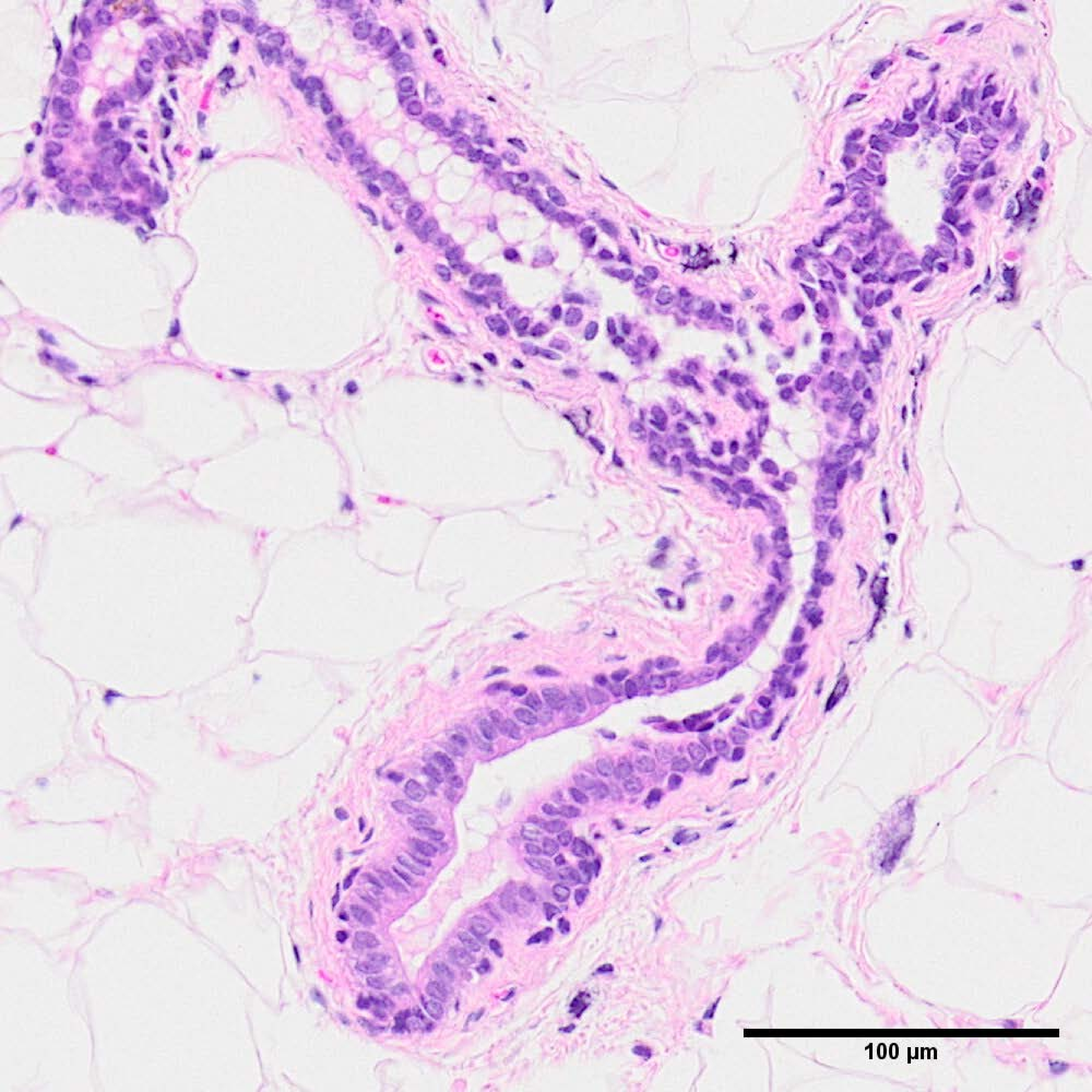
100 µm

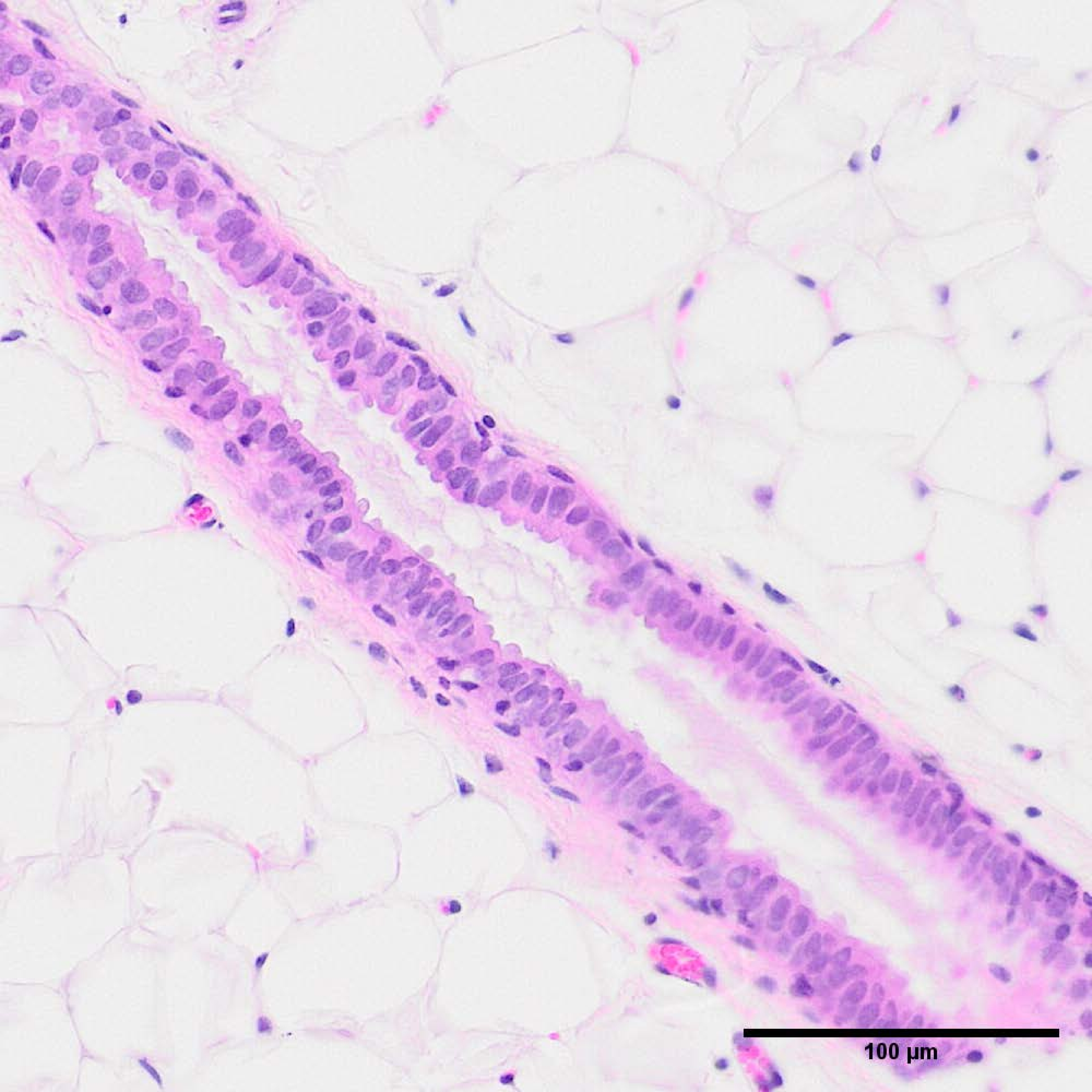
100 µm

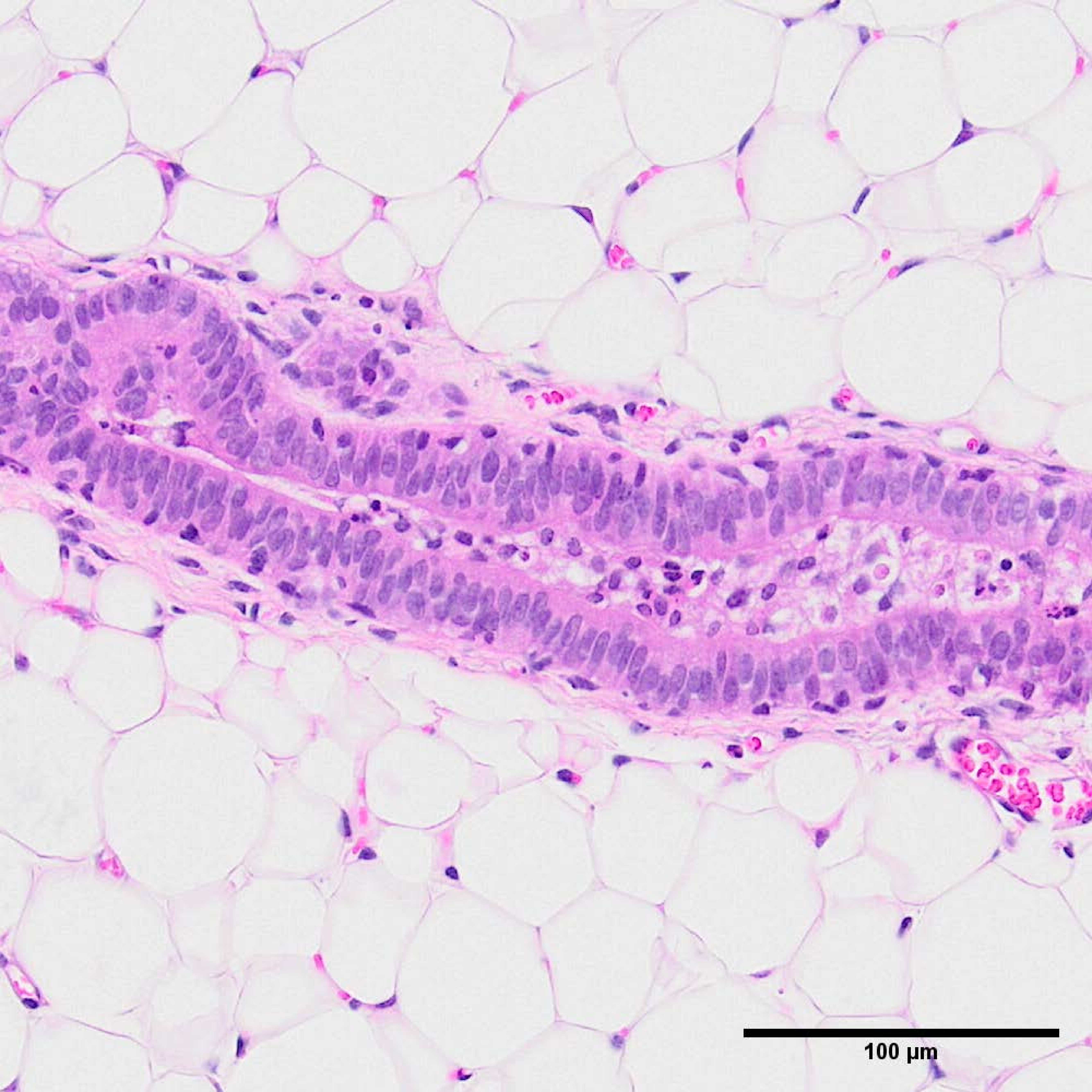
100 µm

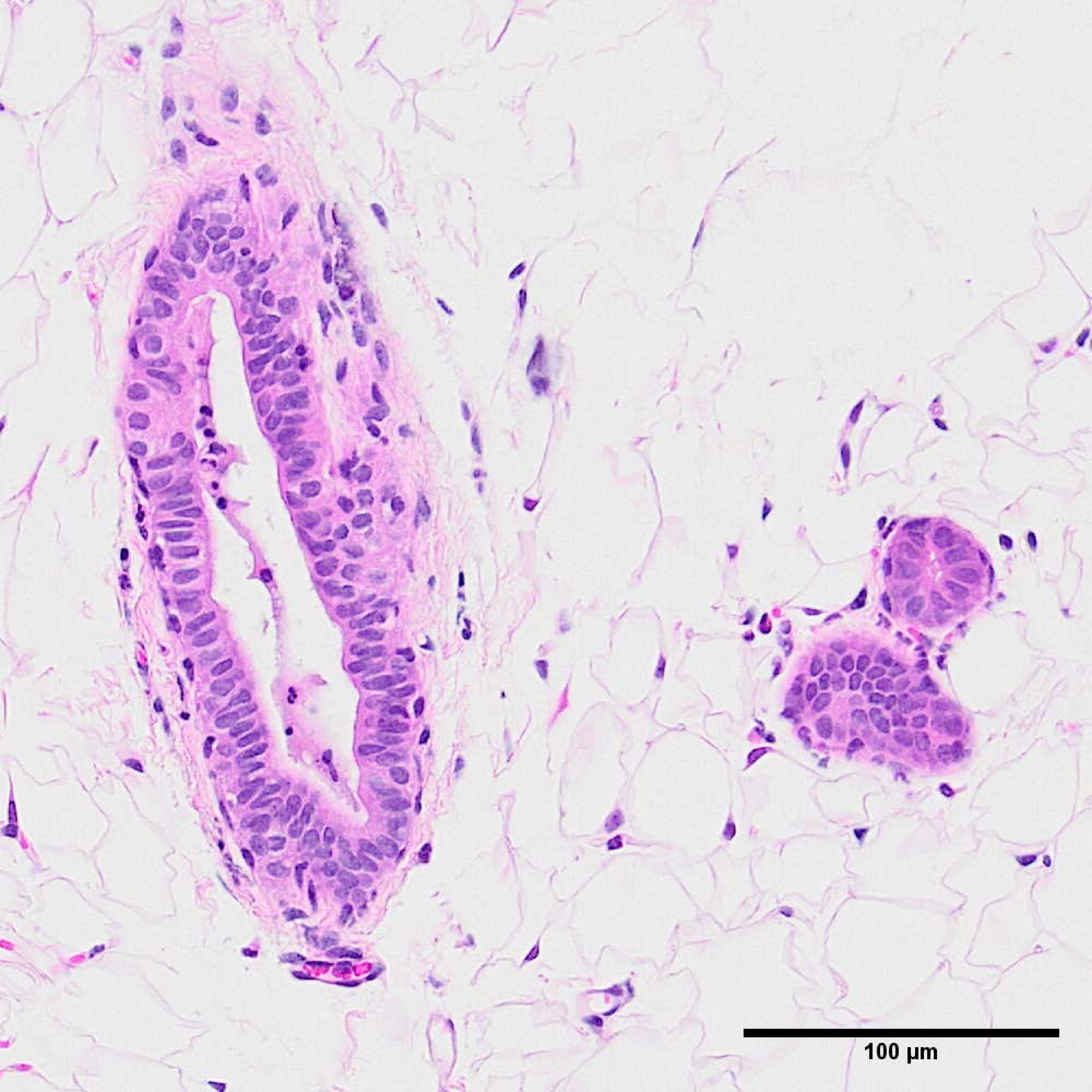
100 µm

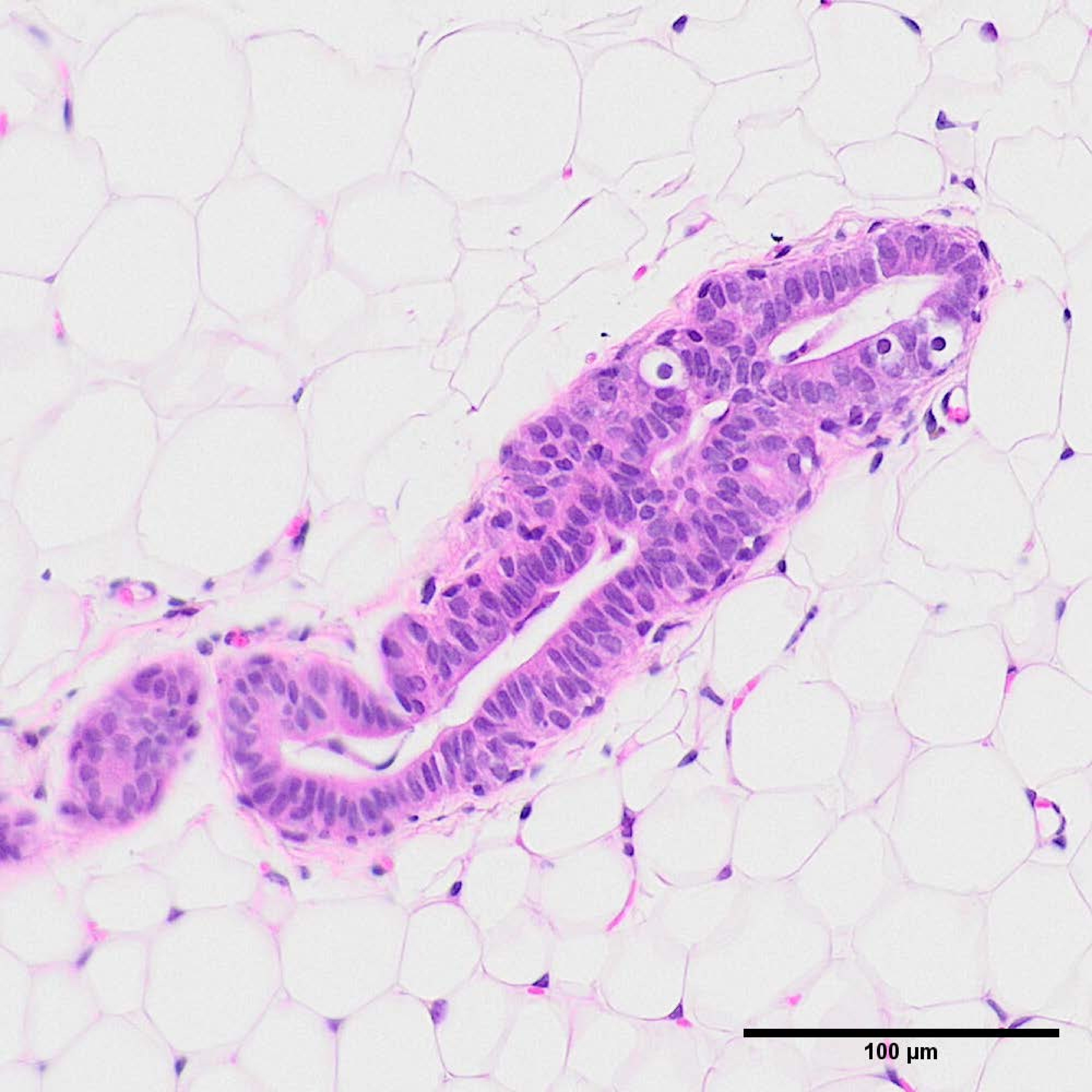
100 µm

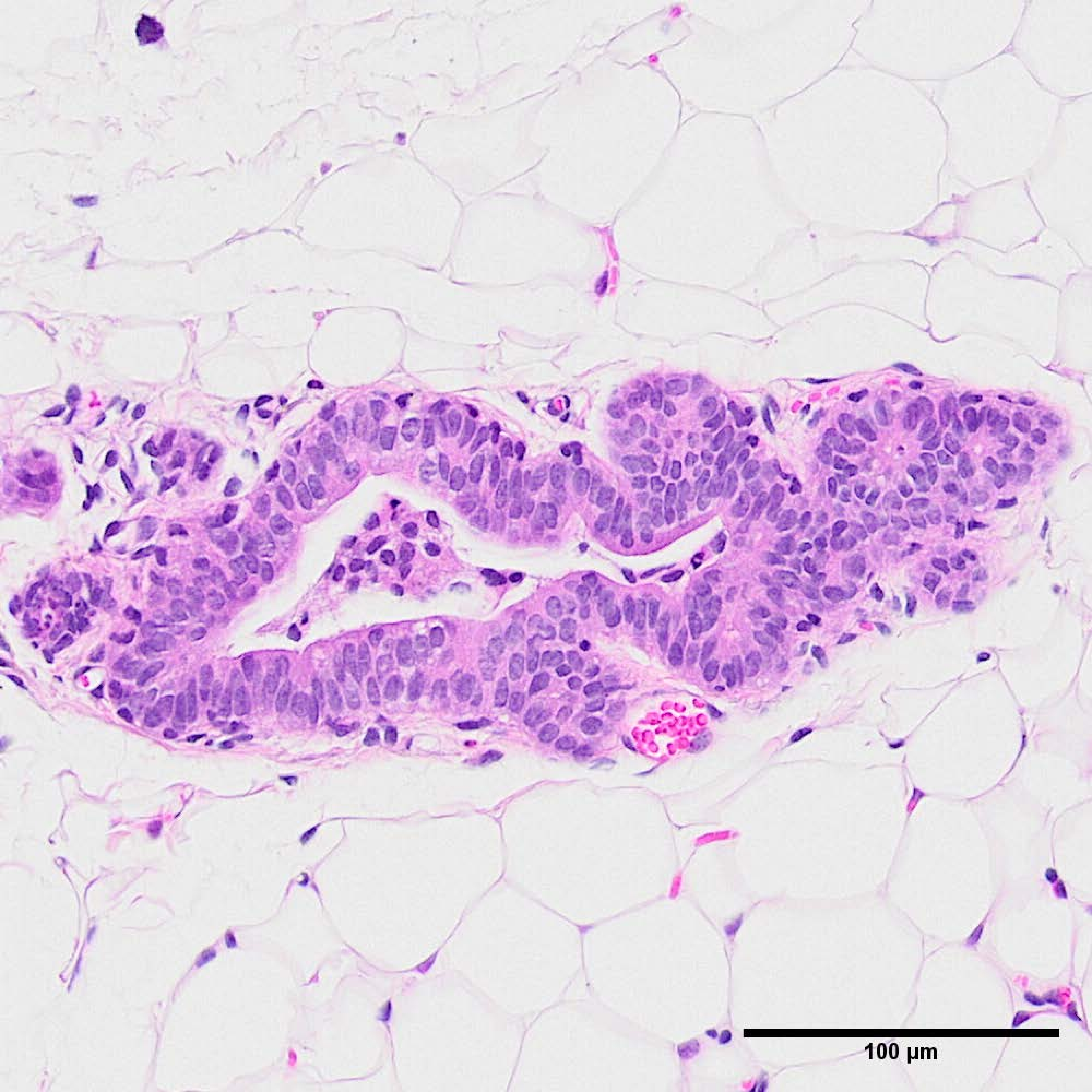
100 µm

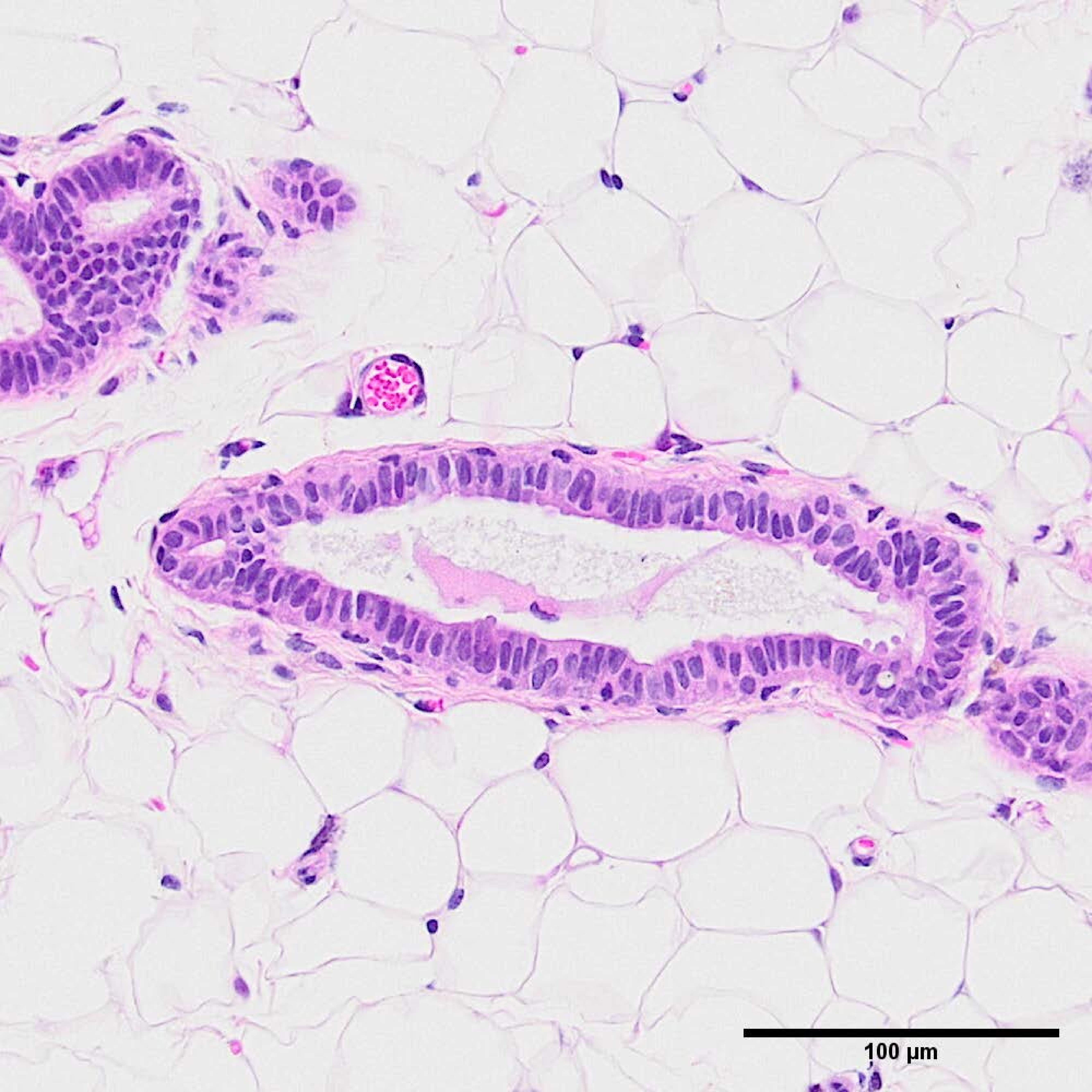
100 µm

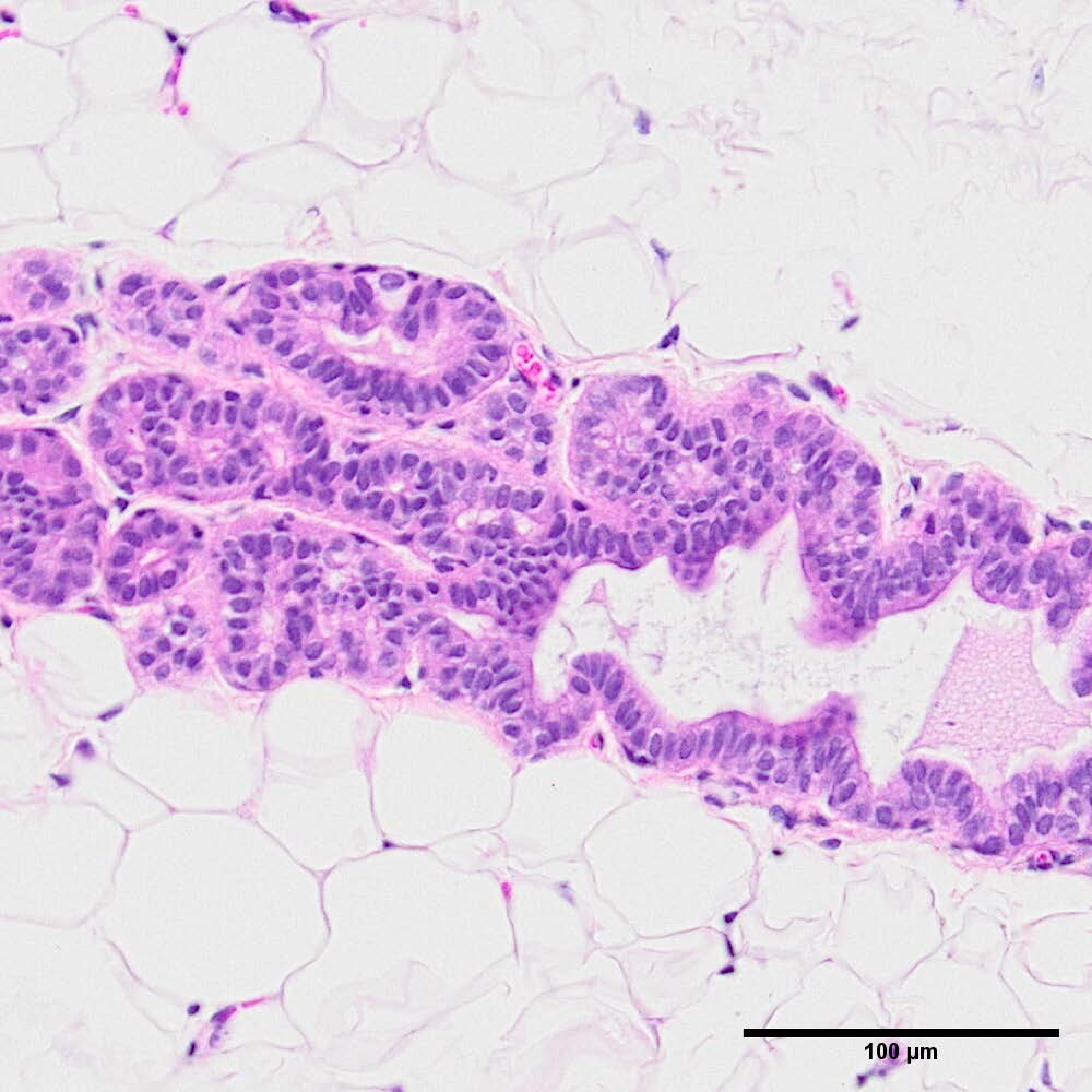
100 µm

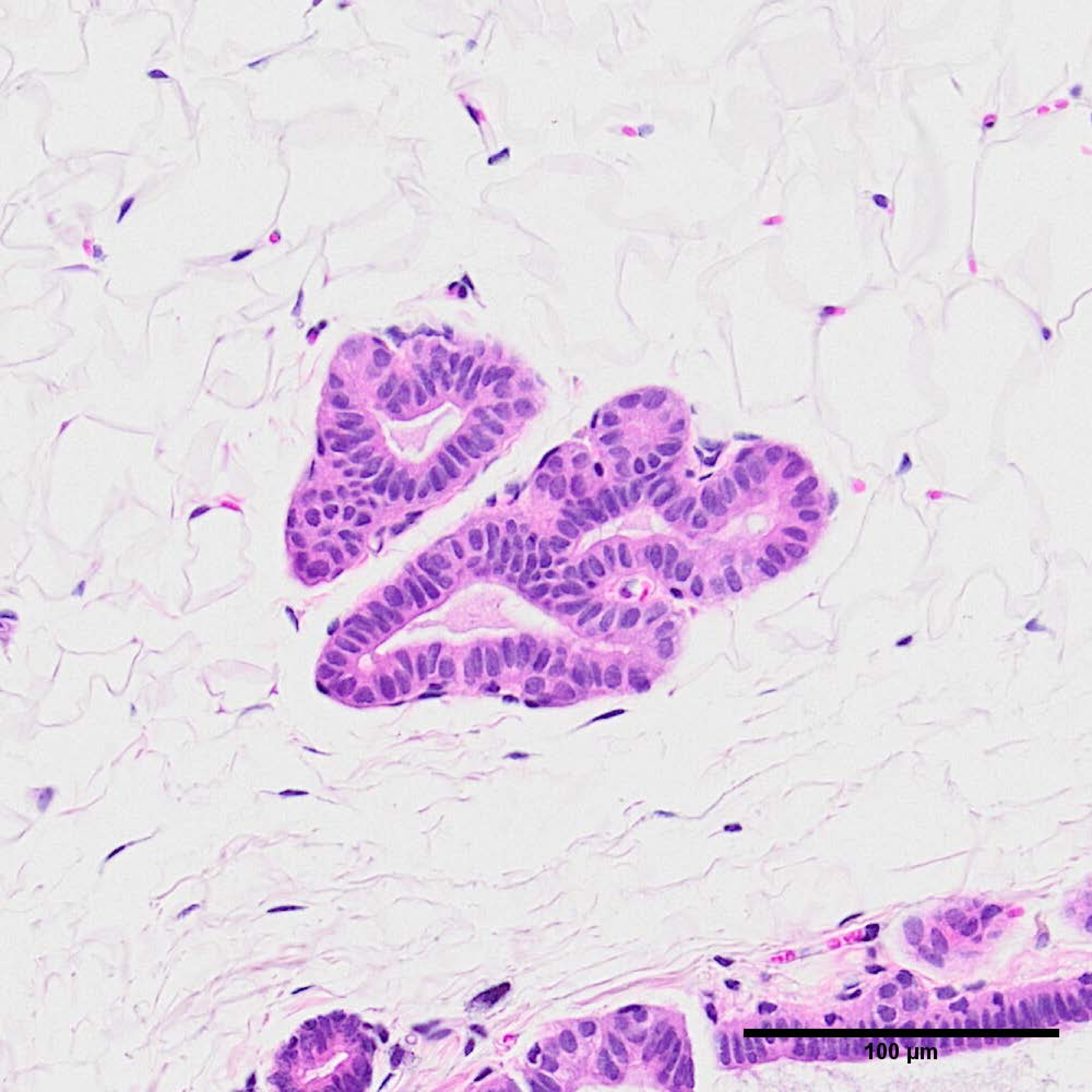
100 µm

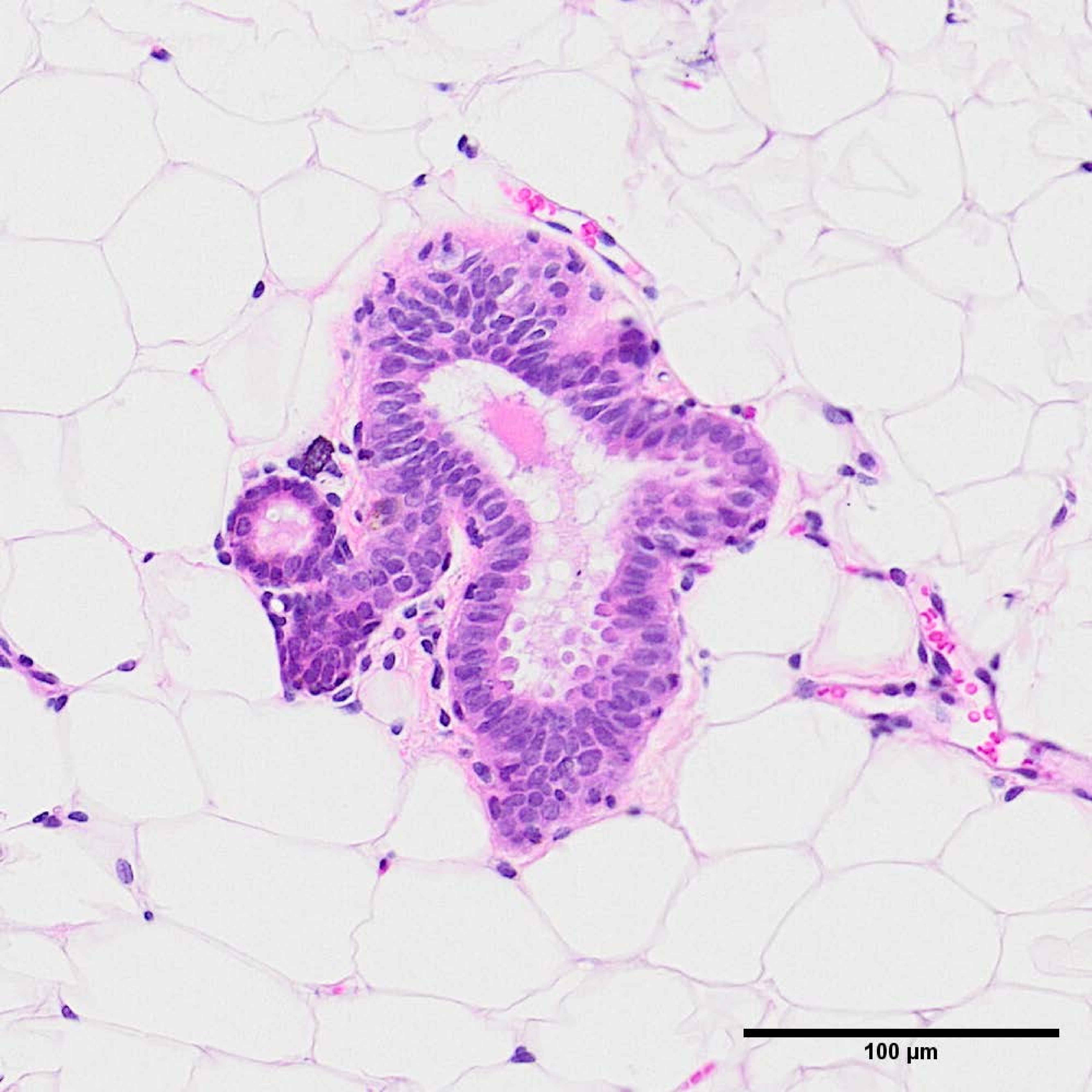
100 µm

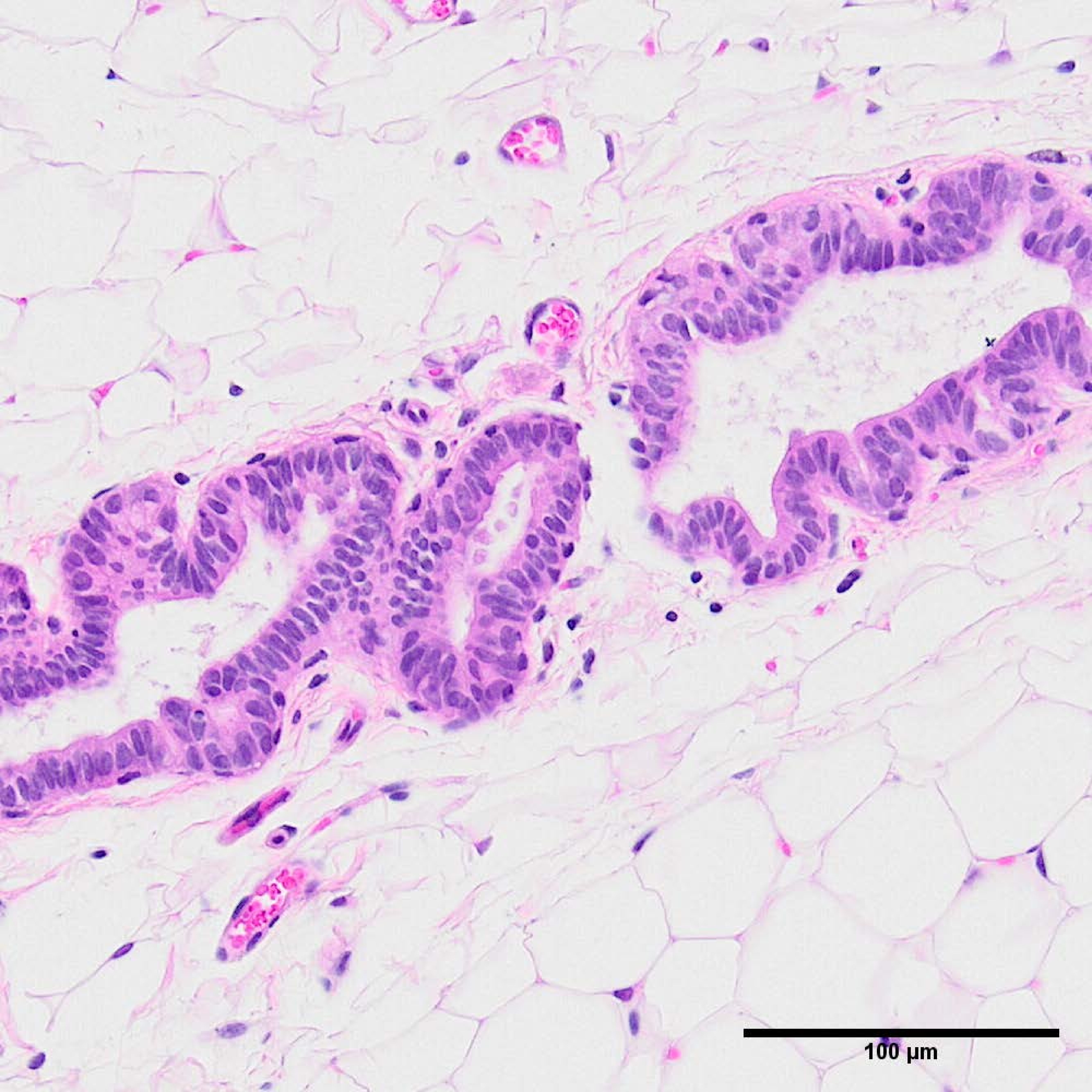
100 µm

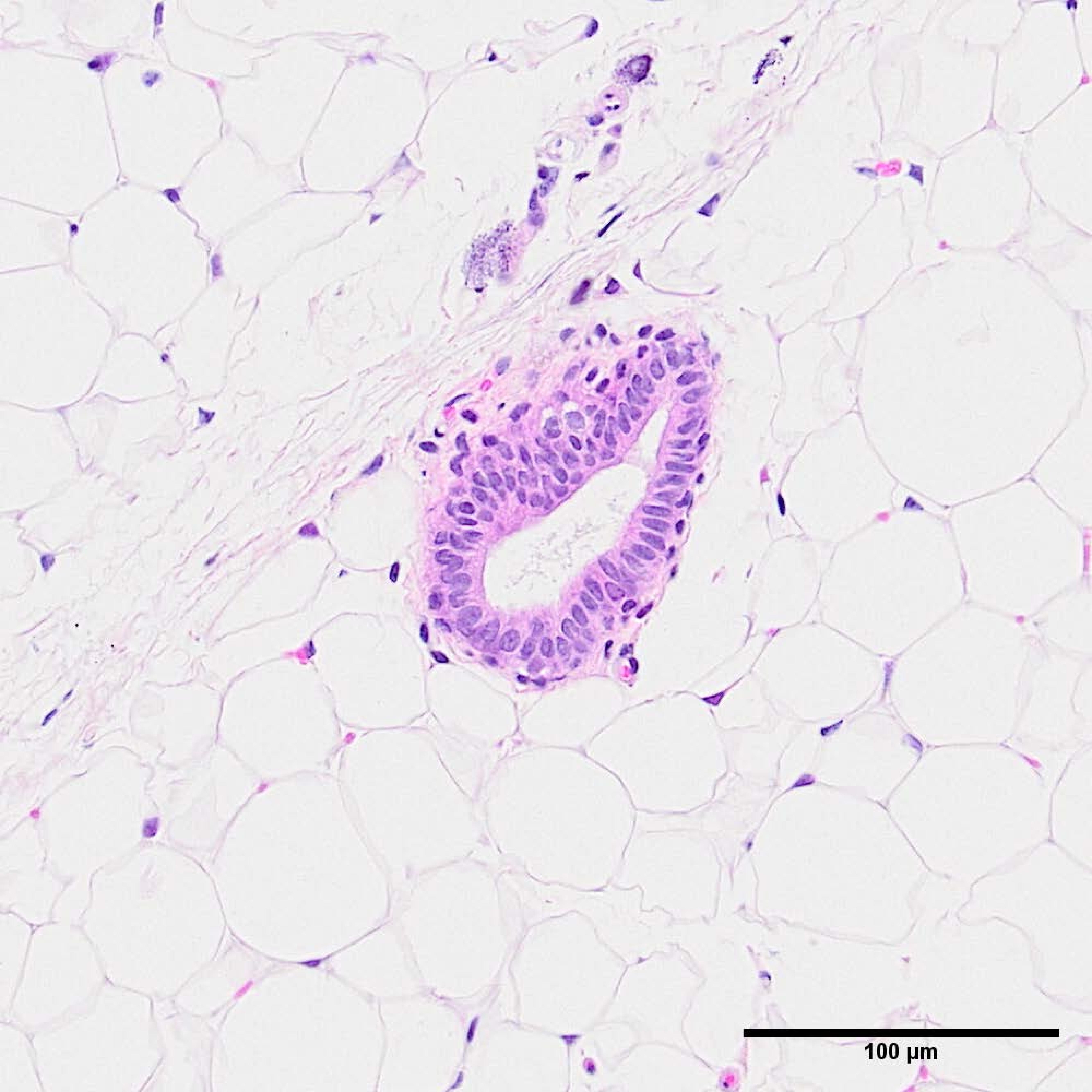
100 µm

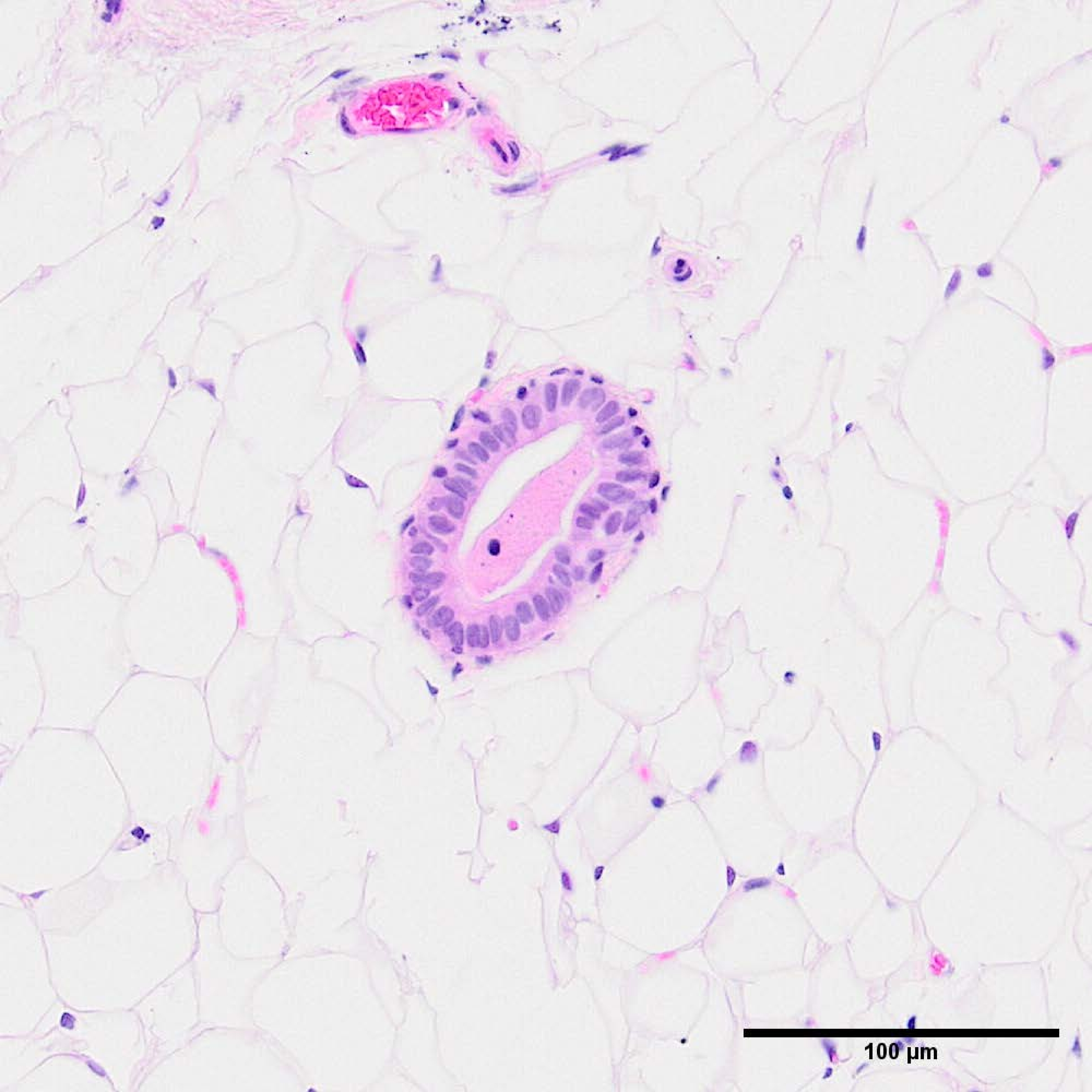
100 µm

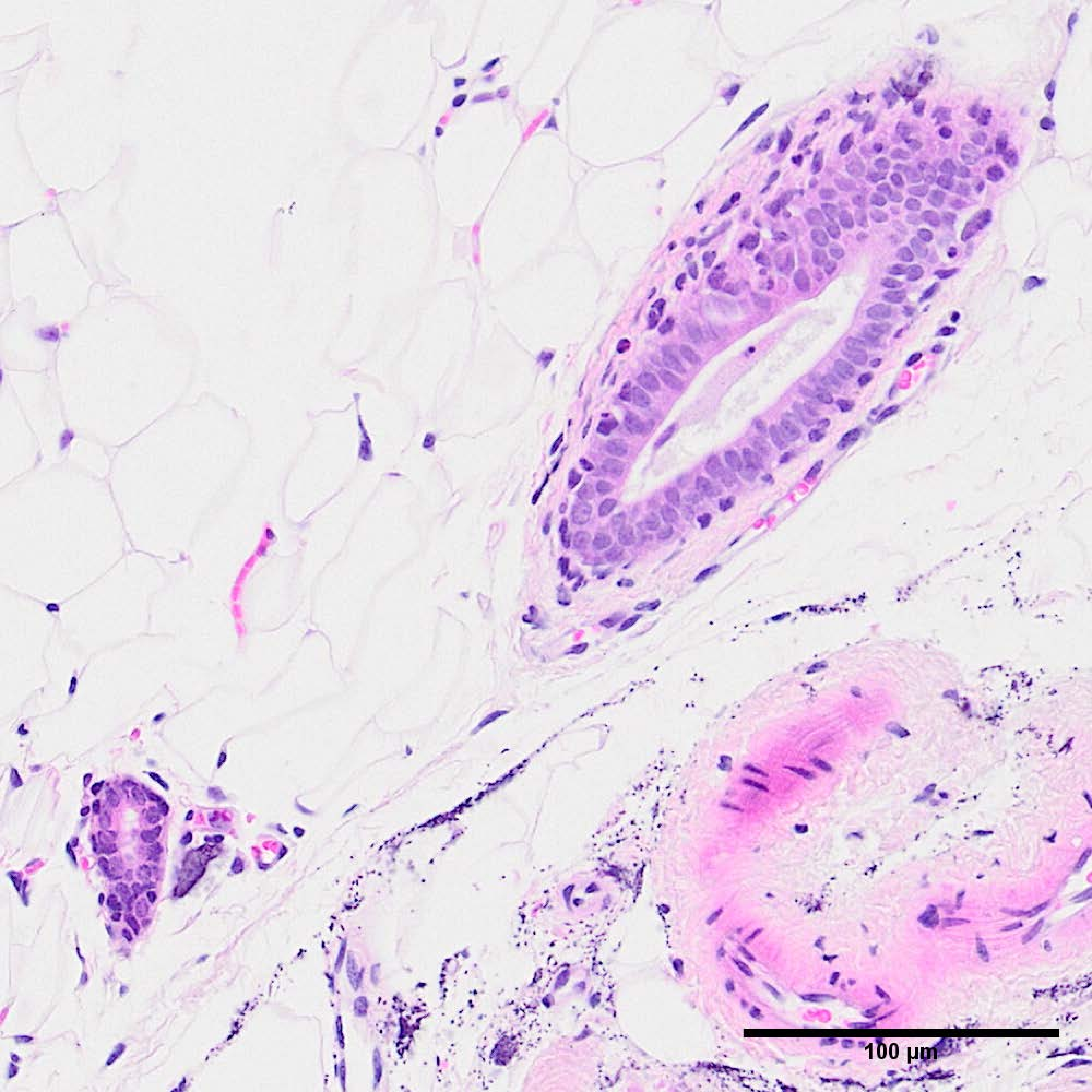
100 µm

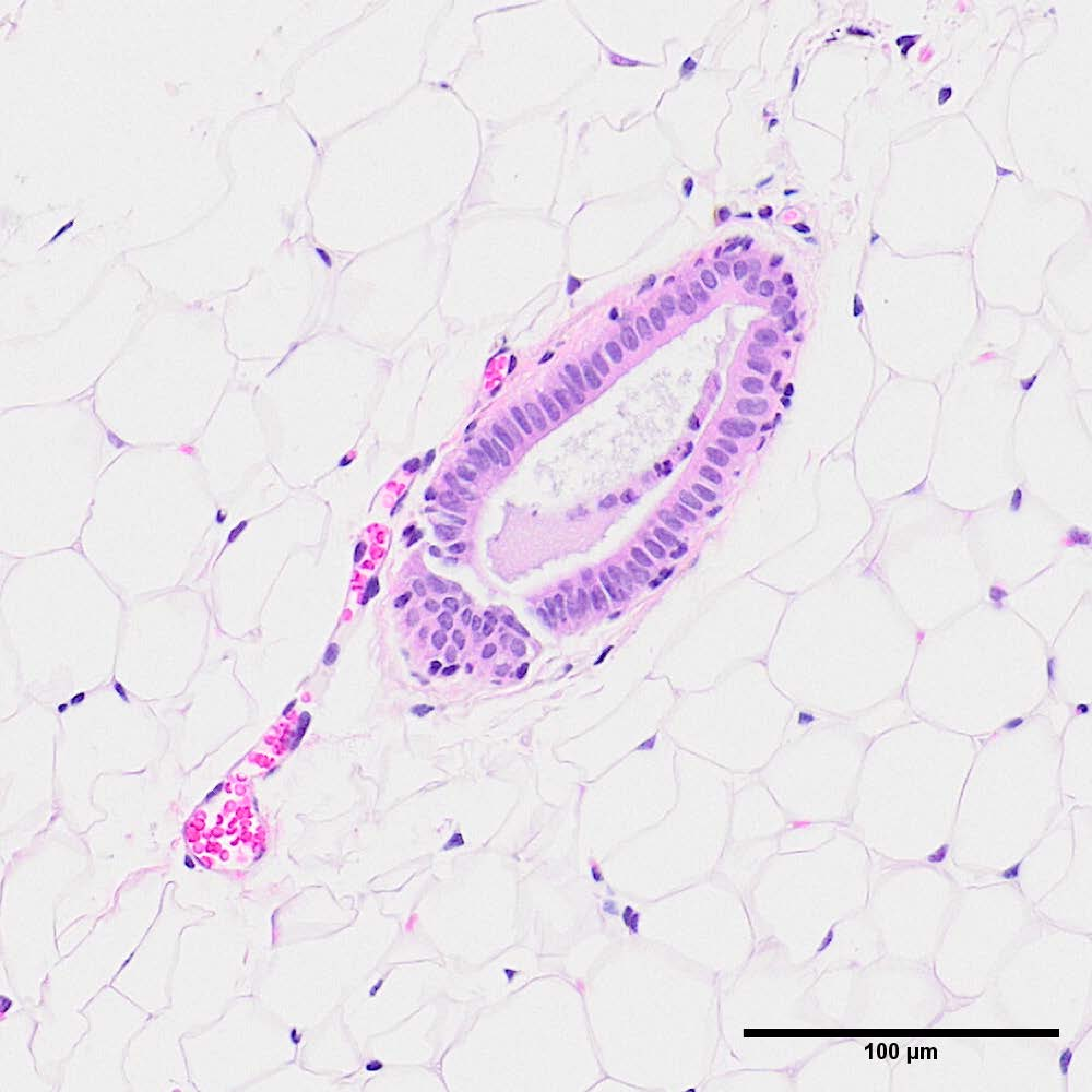
100 µm

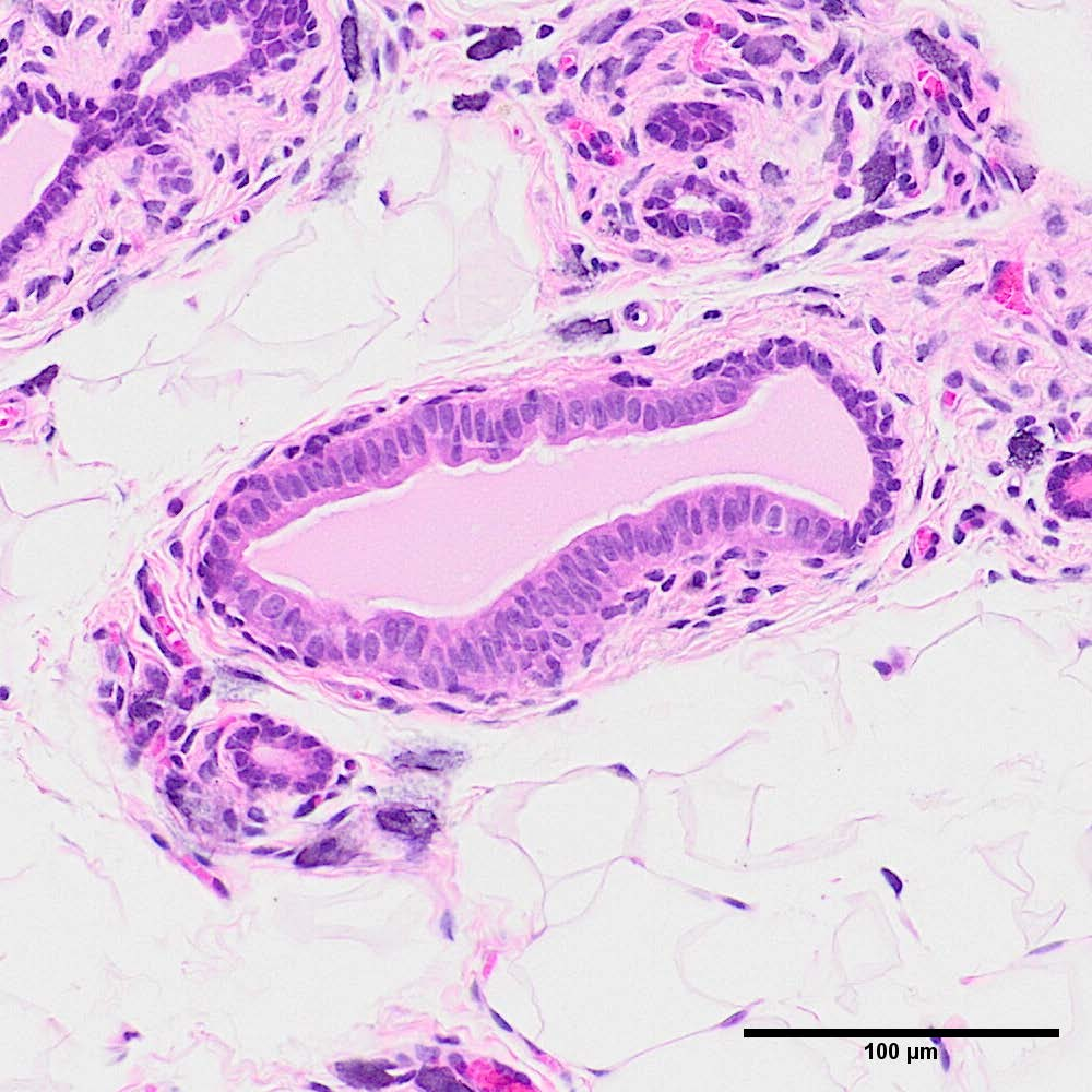
100 µm

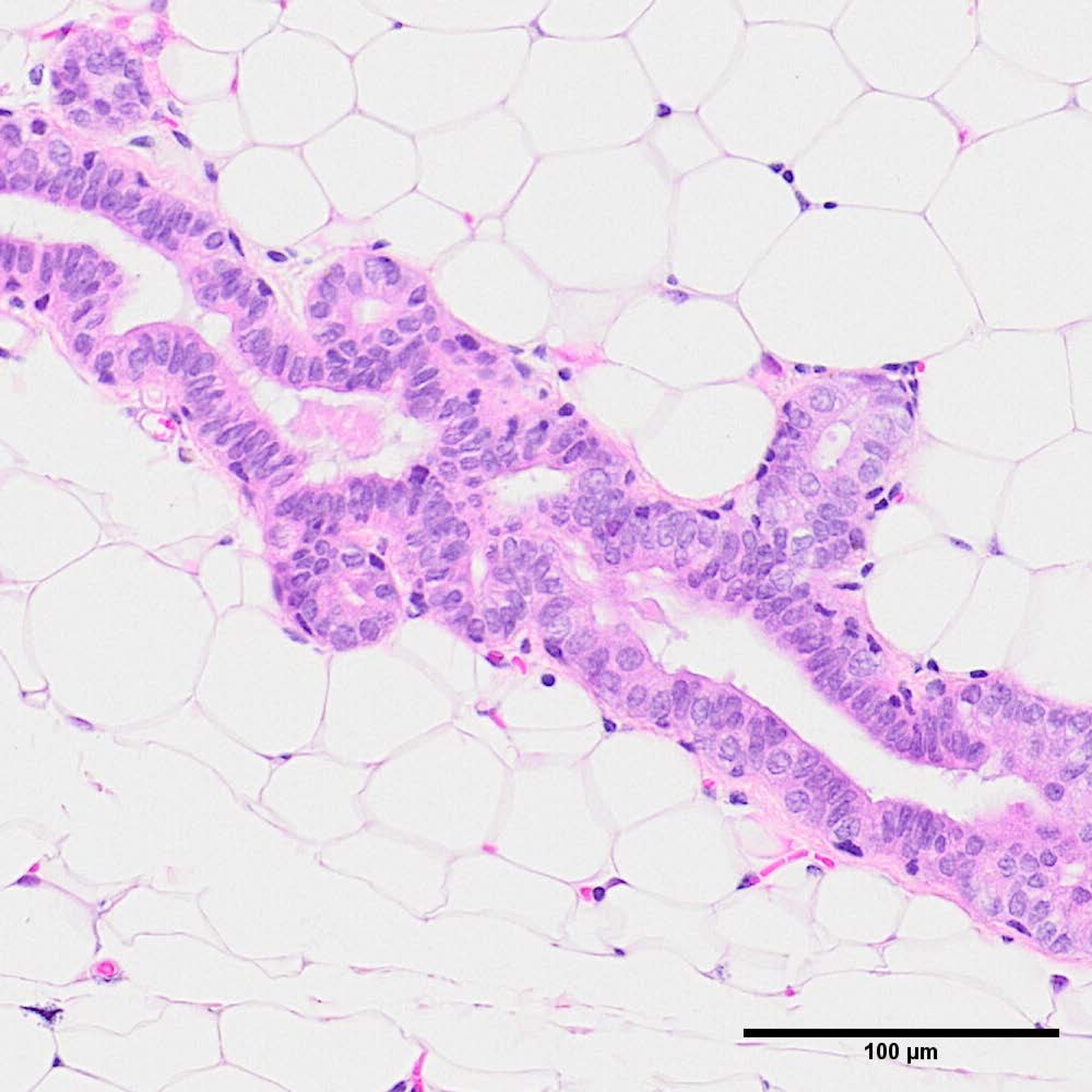
100 µm

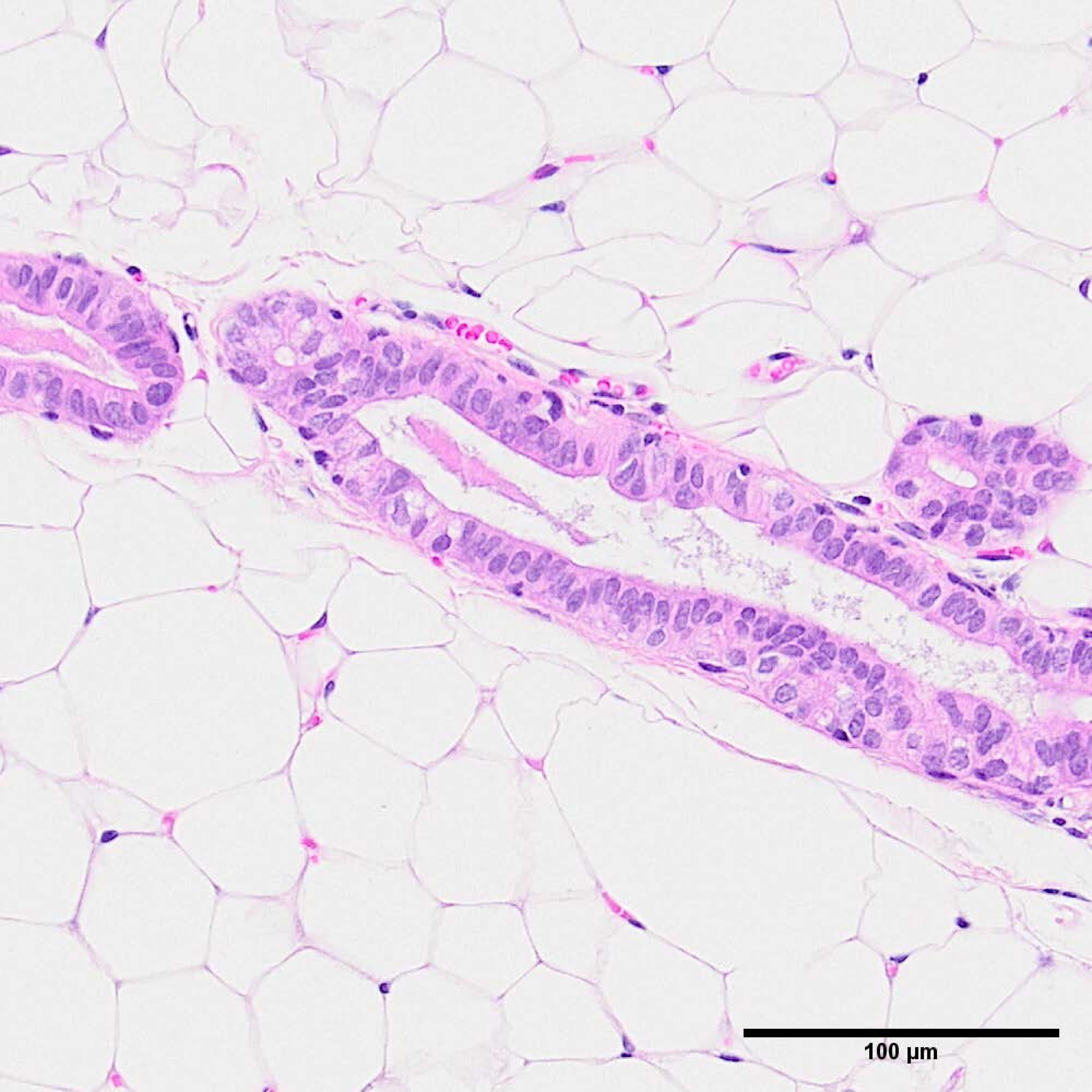
100 µm

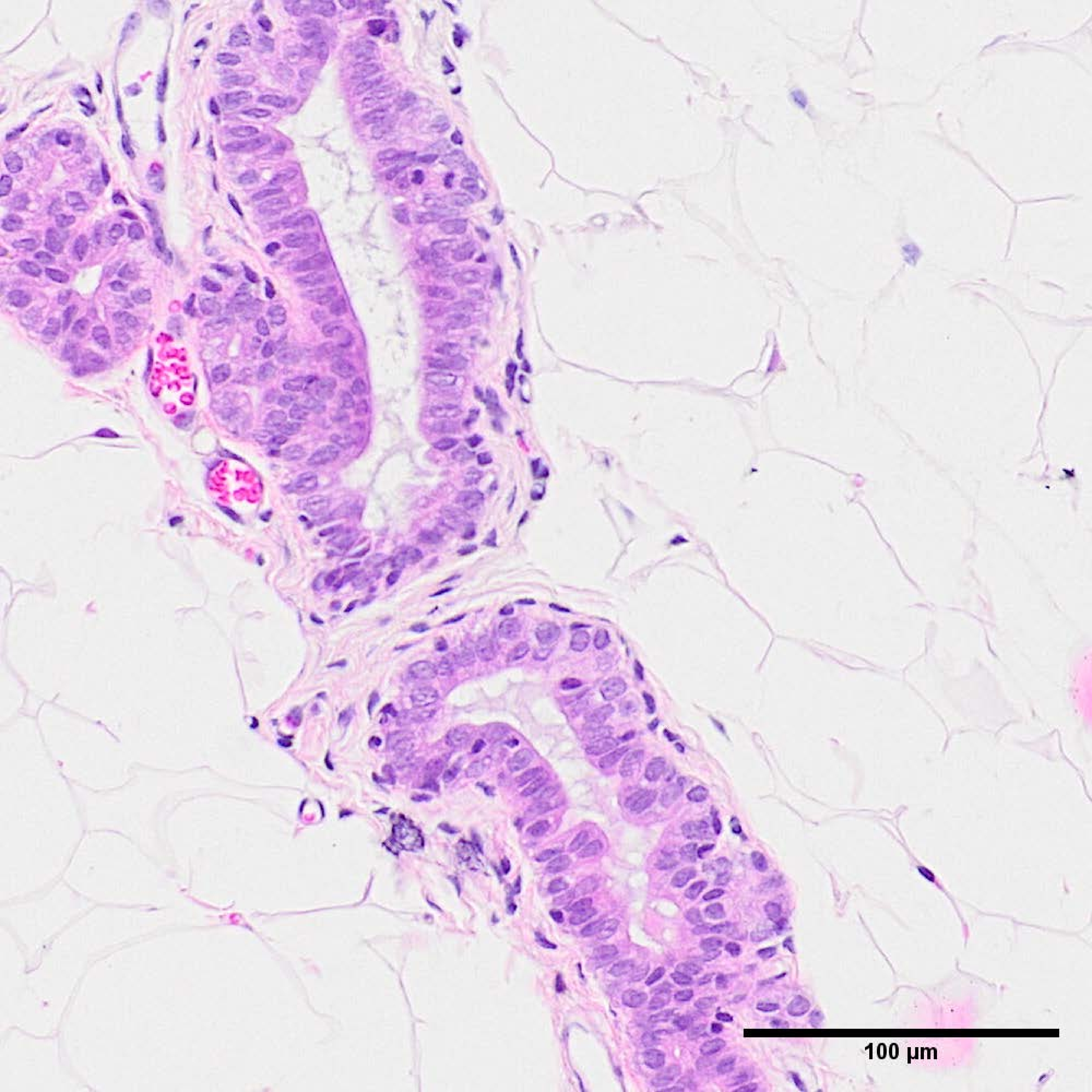
100 µm

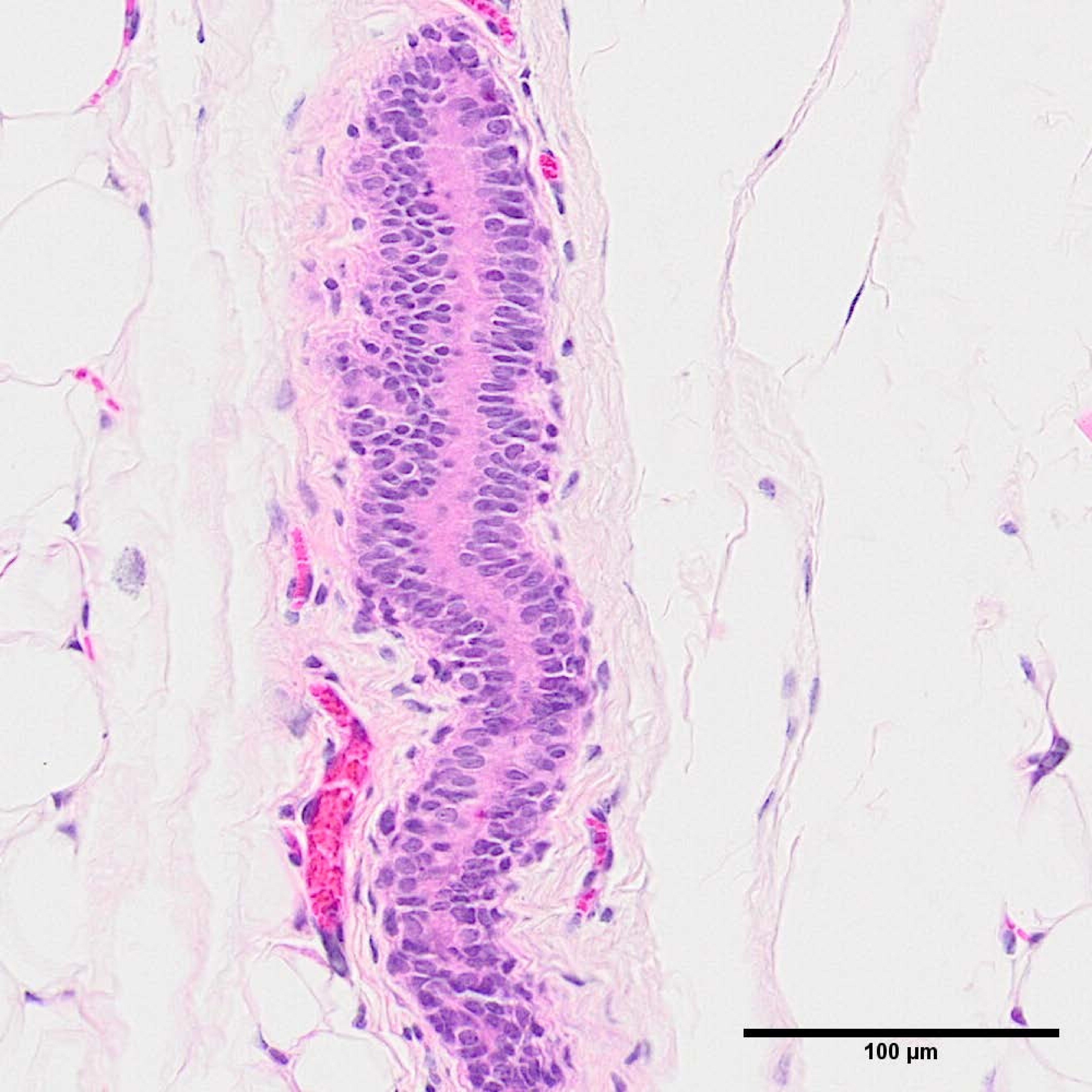
100 µm

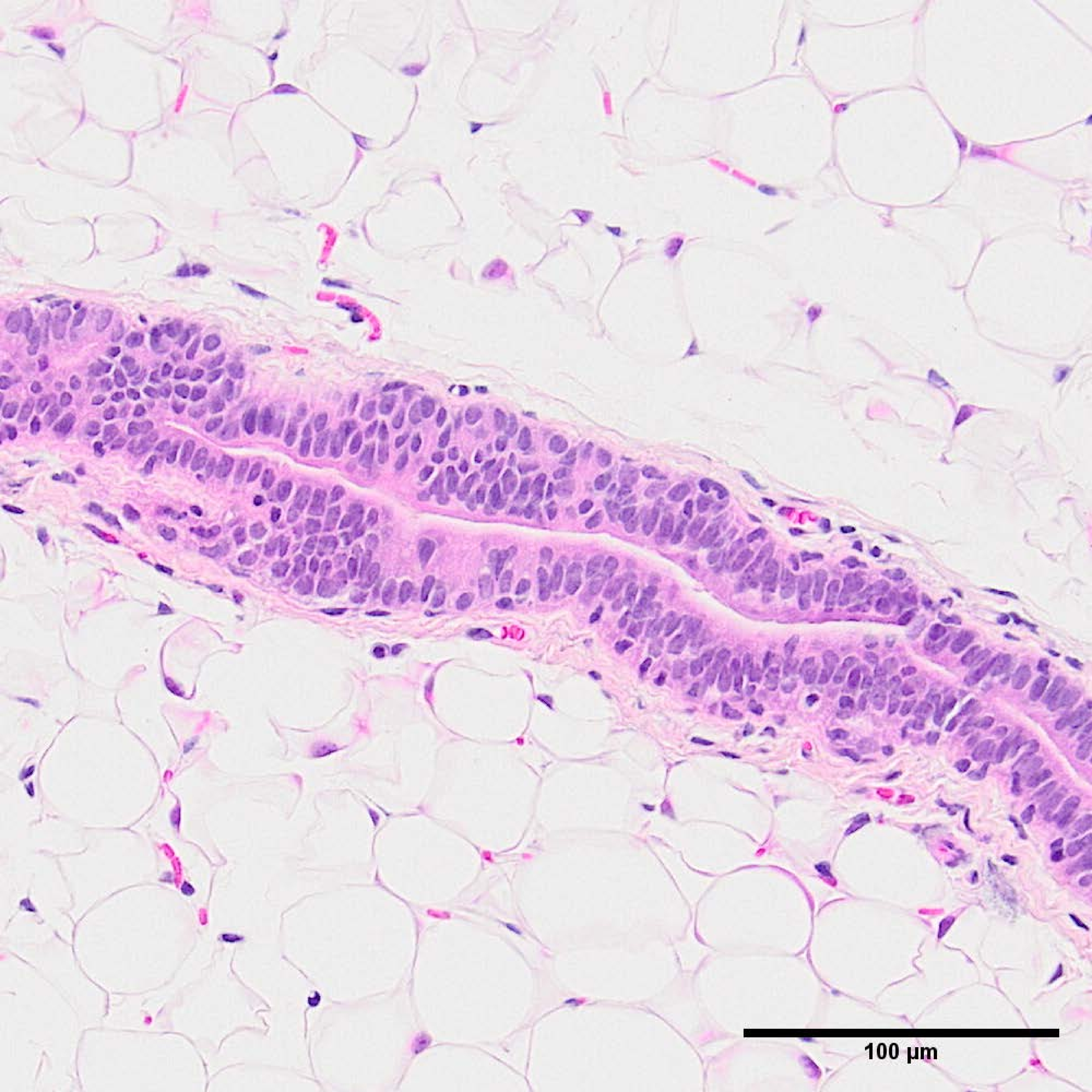
100 µm

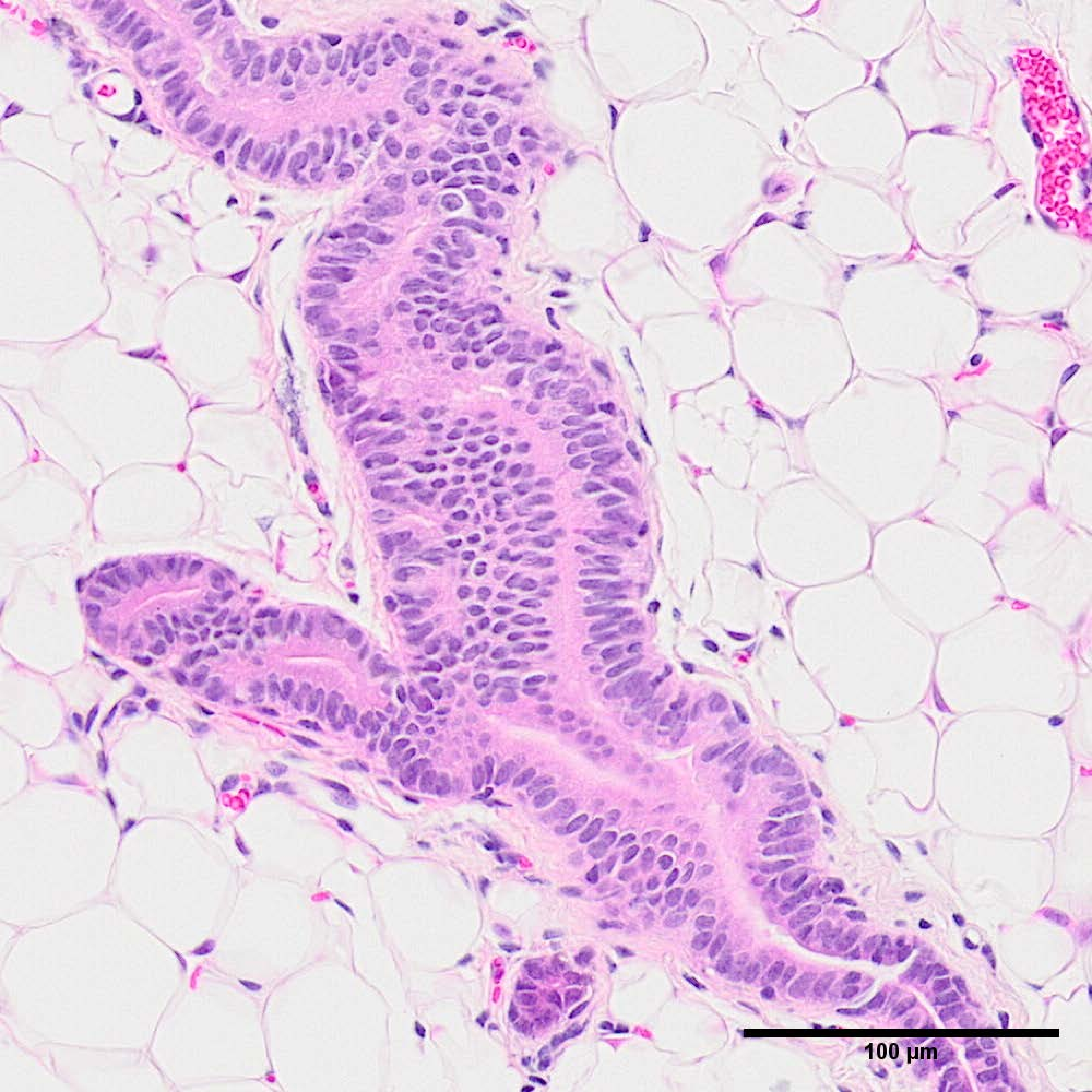
100 µm

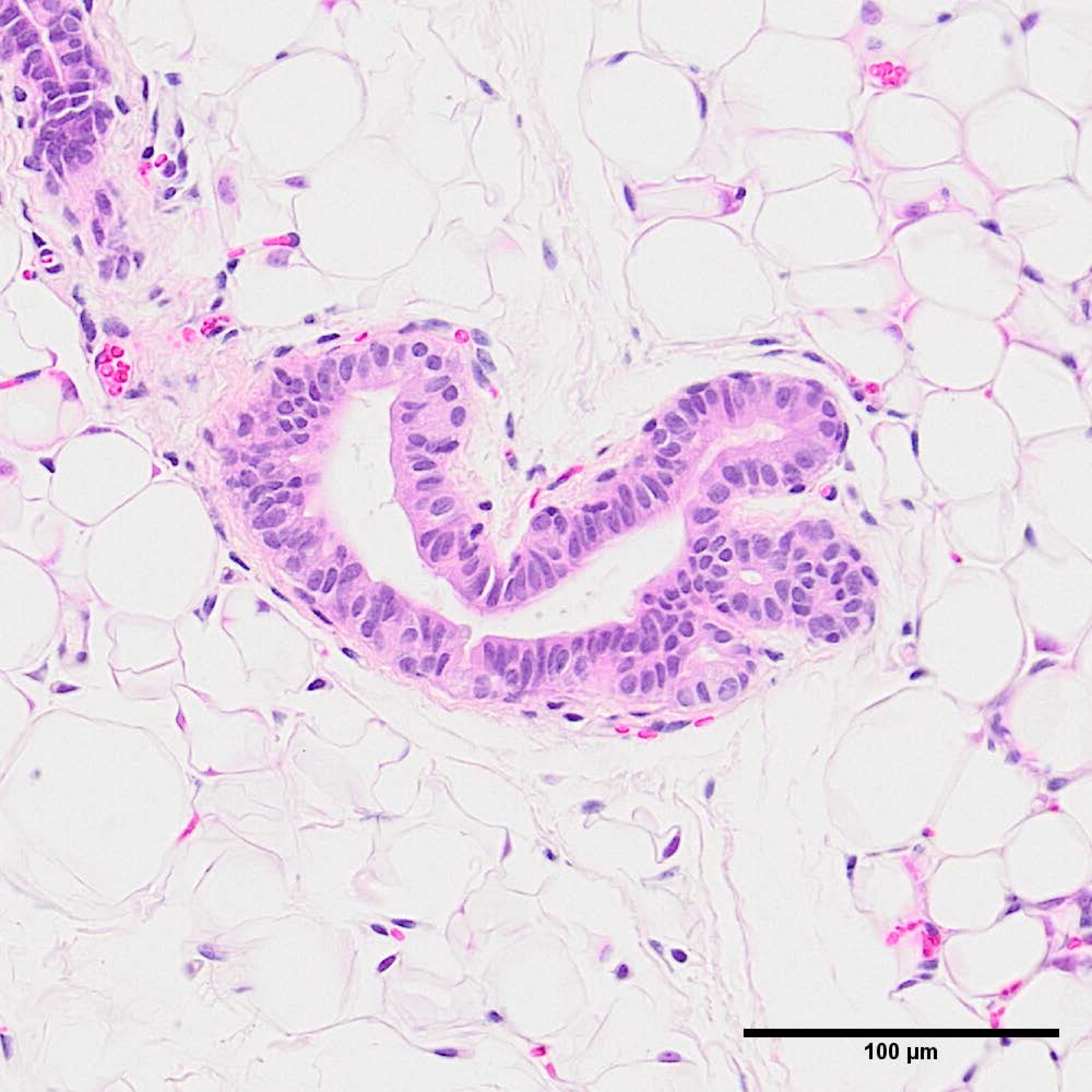
100 µm

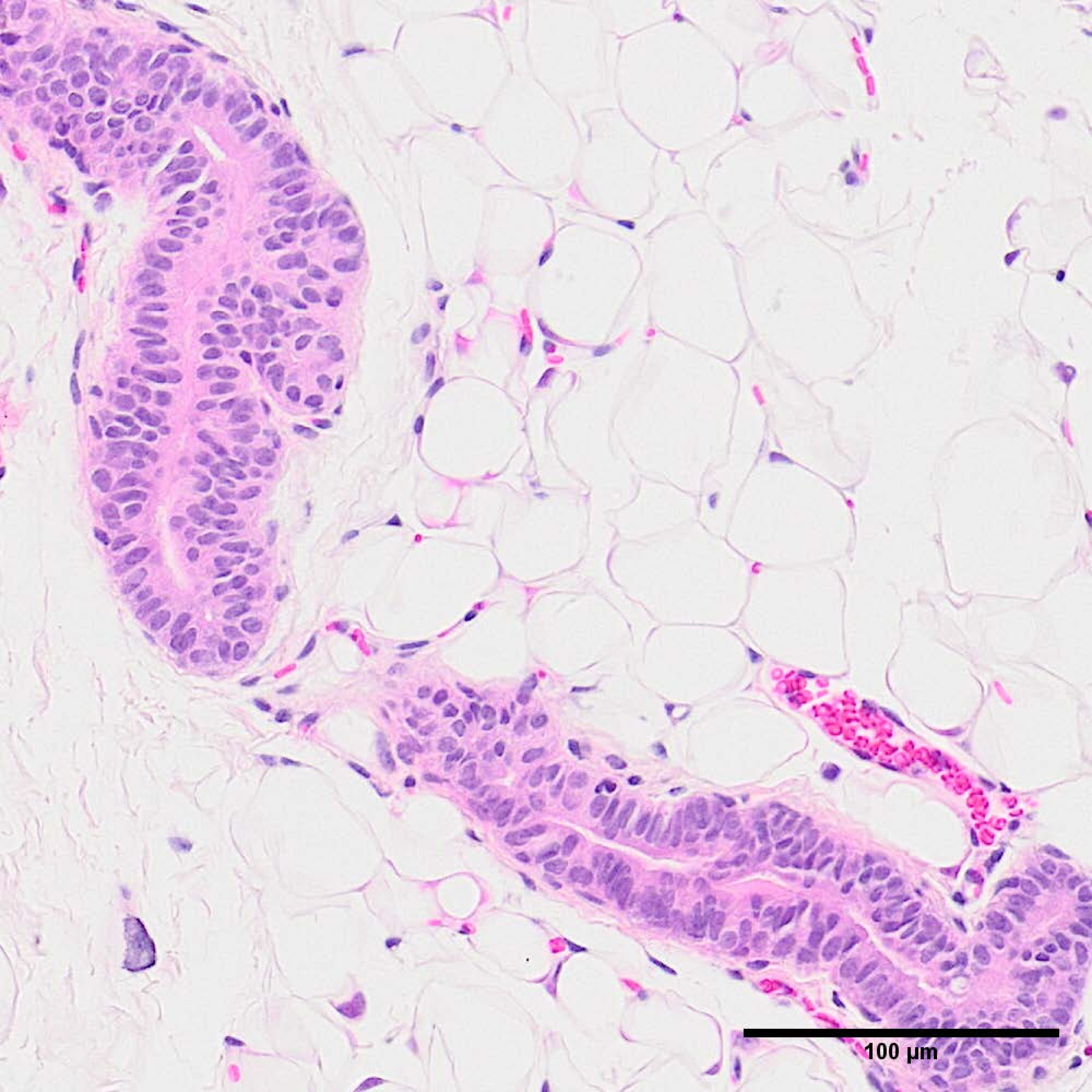
100 µm

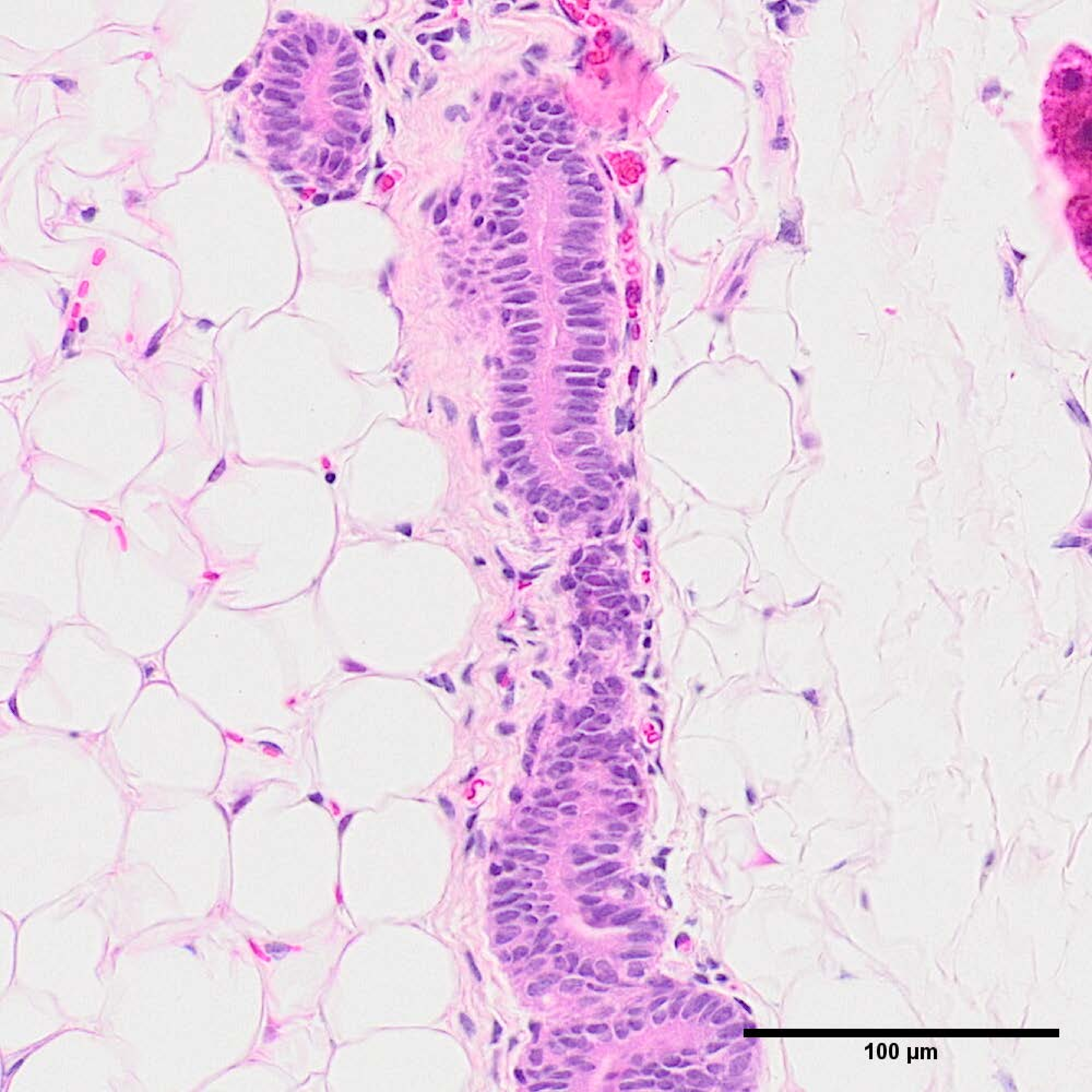
100 µm

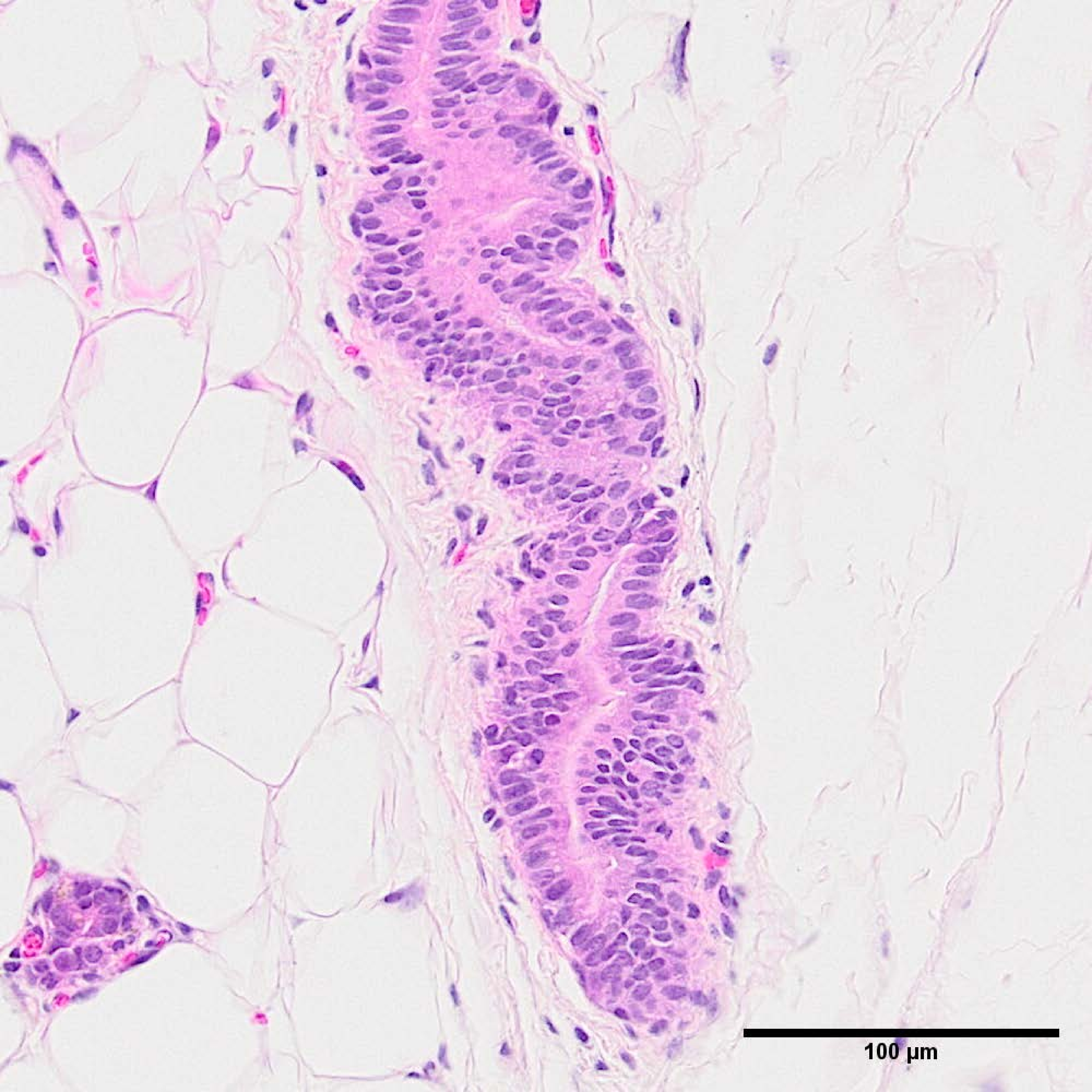
100 µm

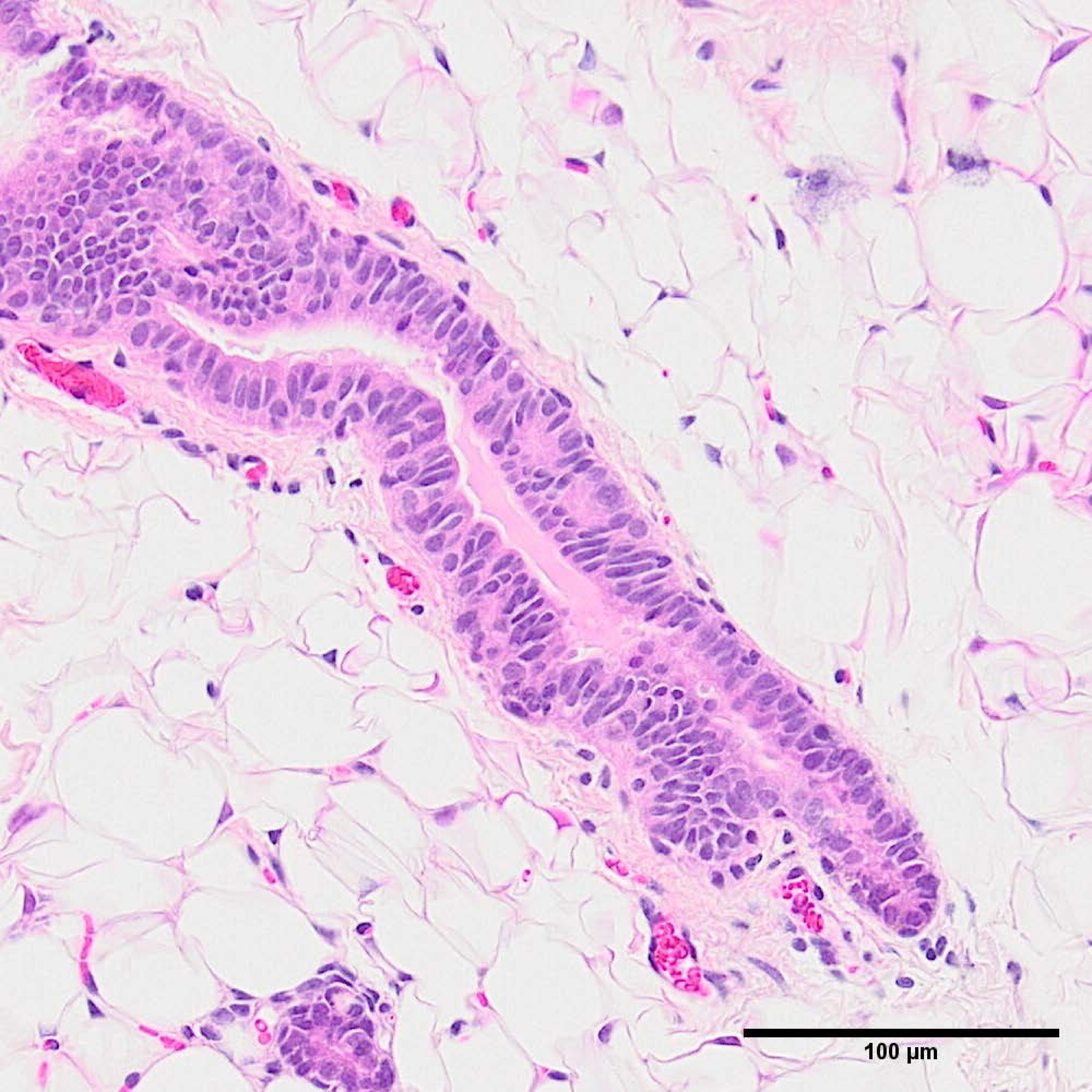
100 µm

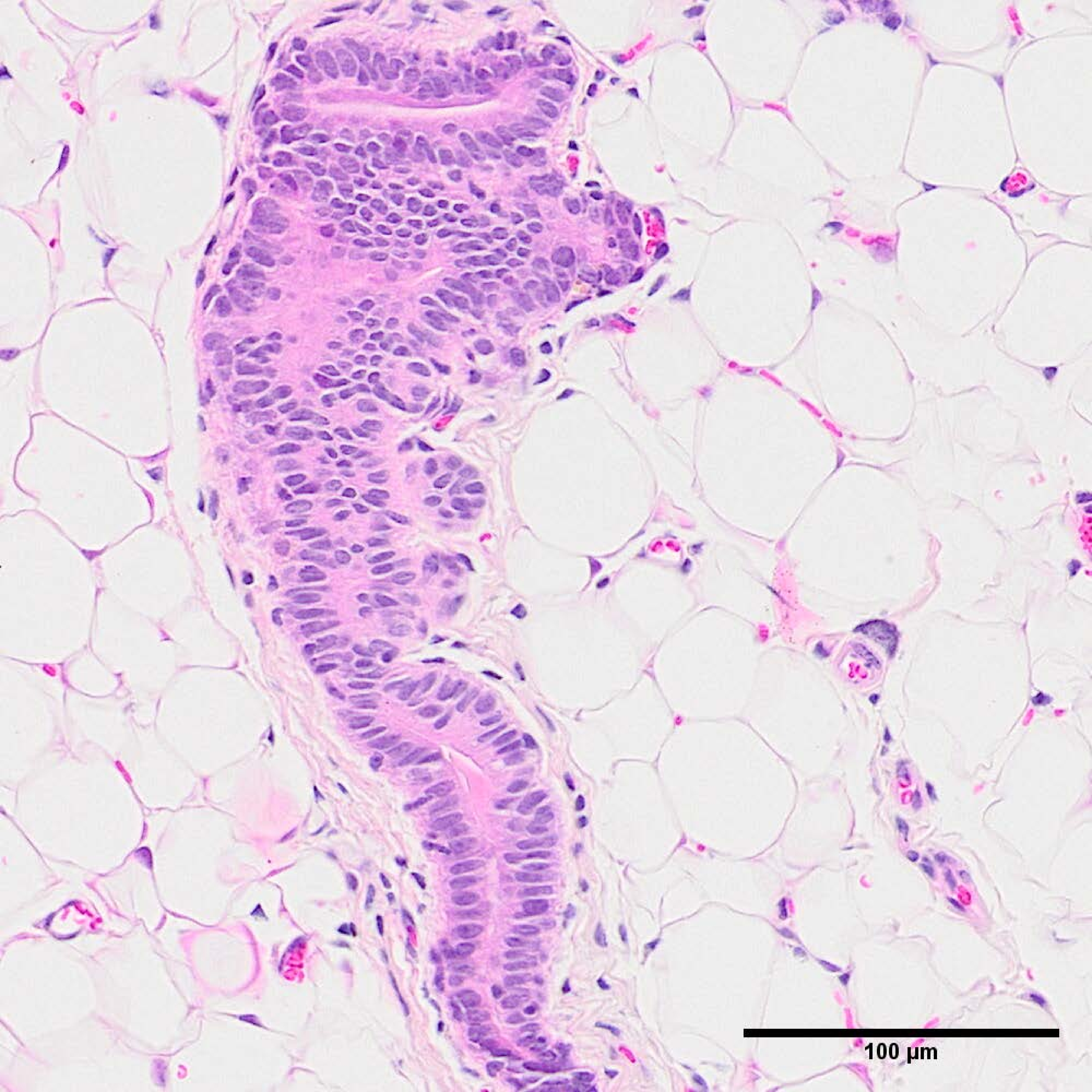
100 µm

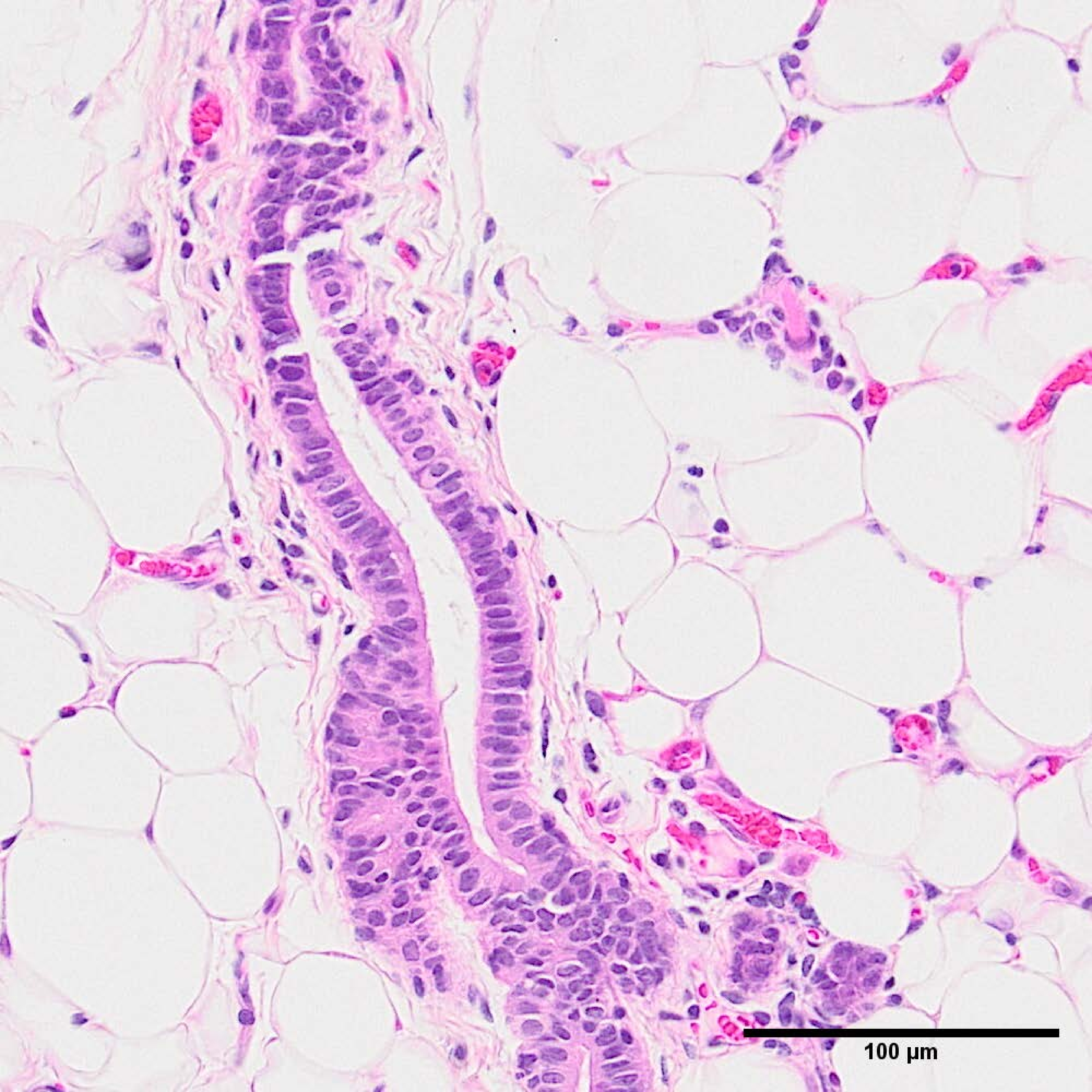
100 µm

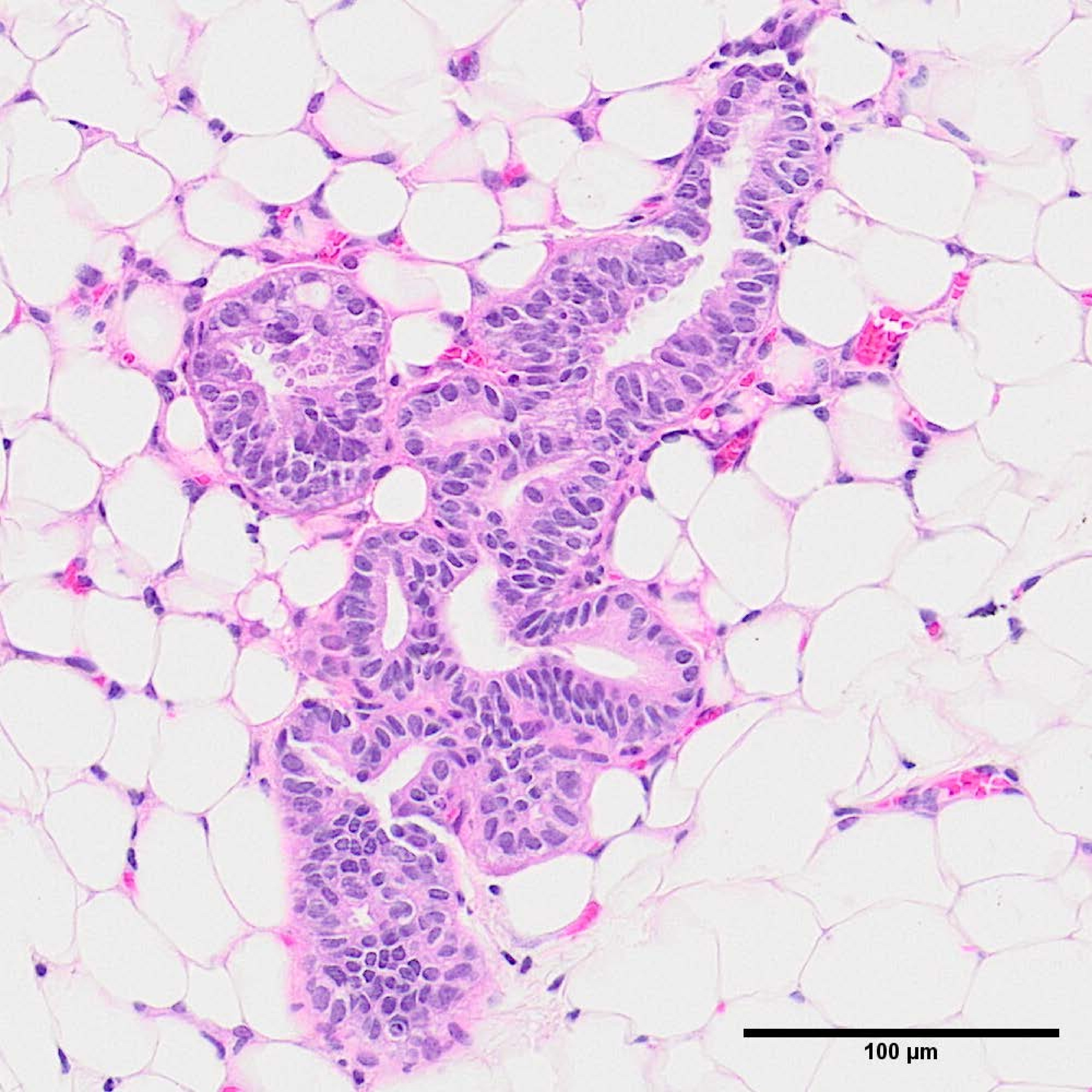
100 µm

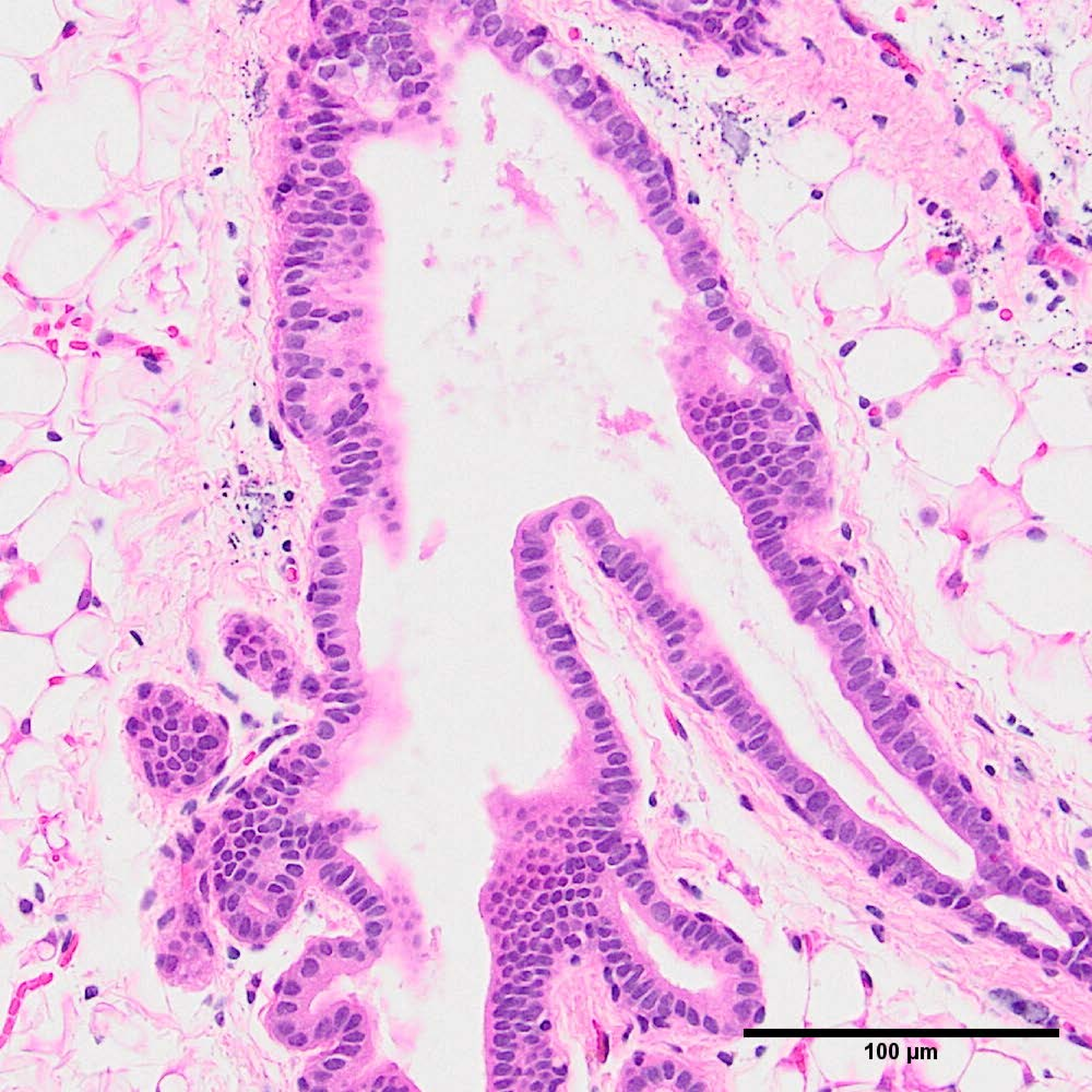
100 µm

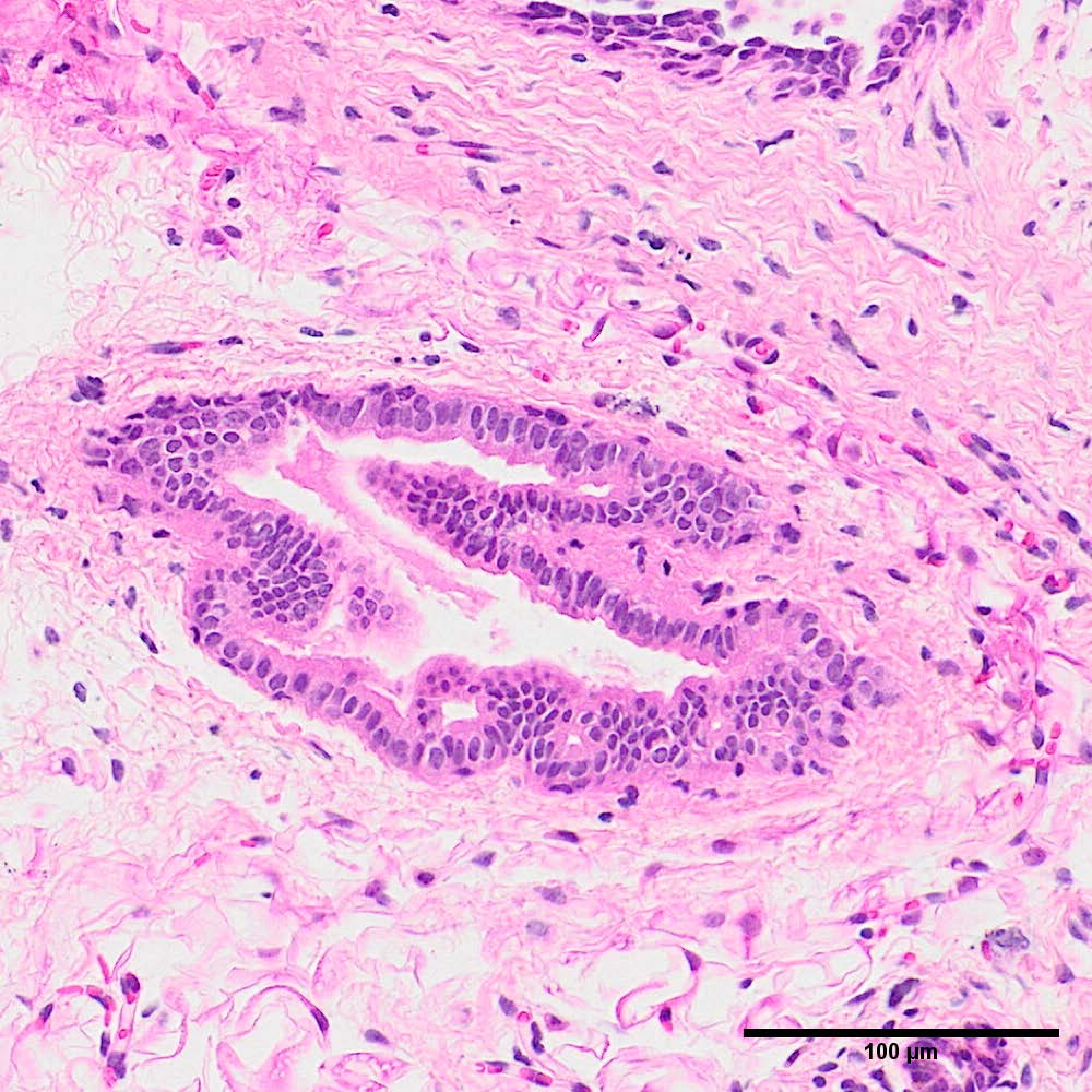
100 µm

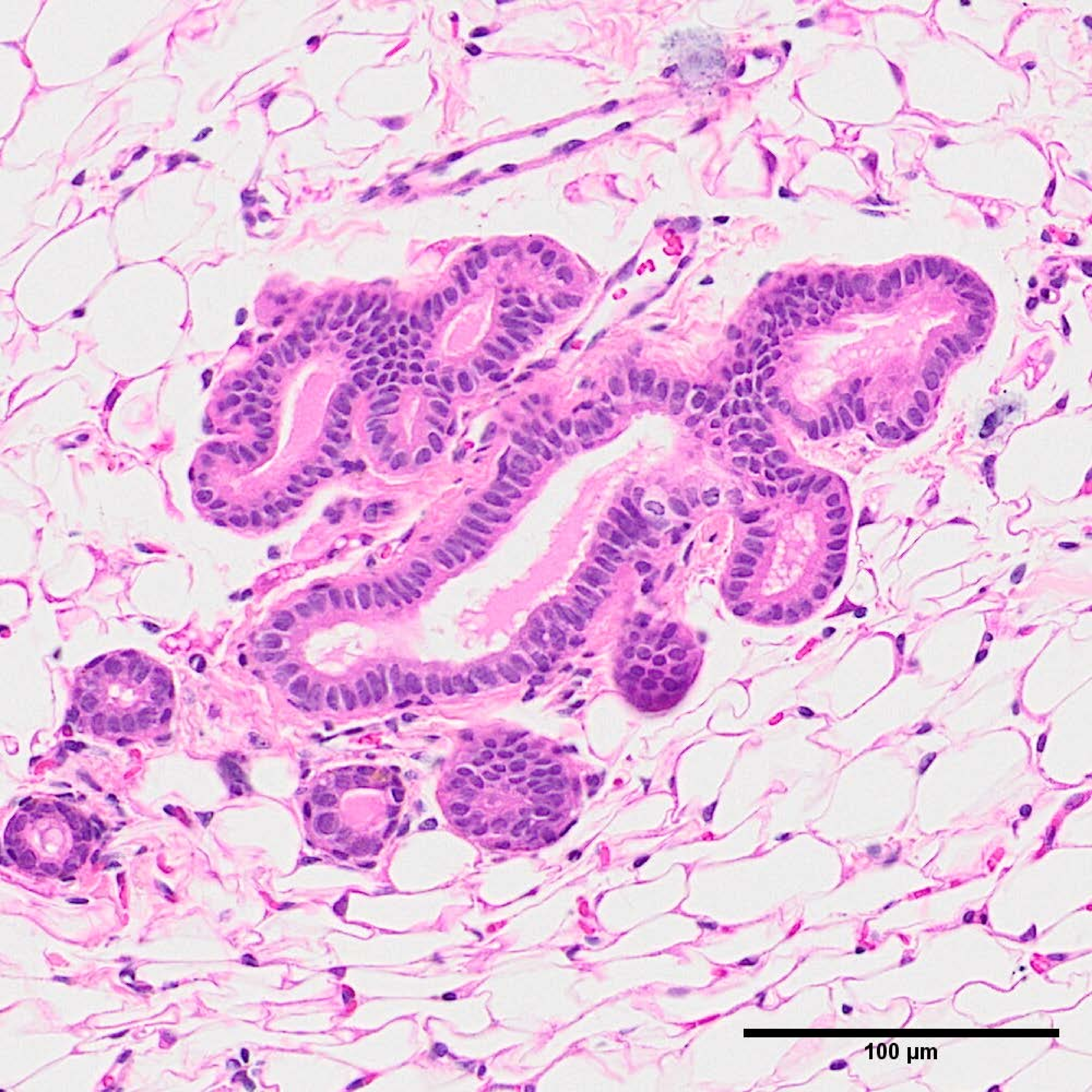
100 µm

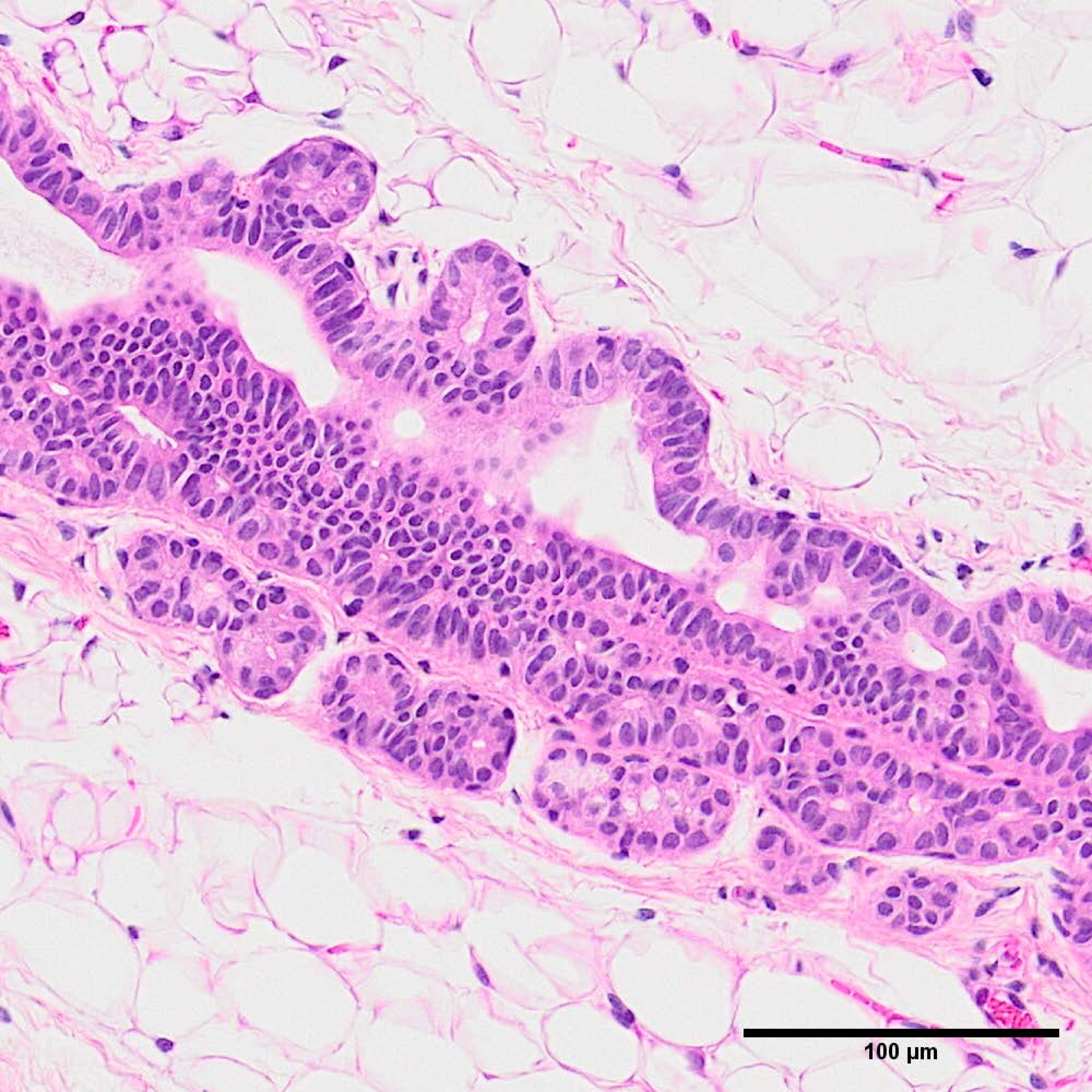
100 µm

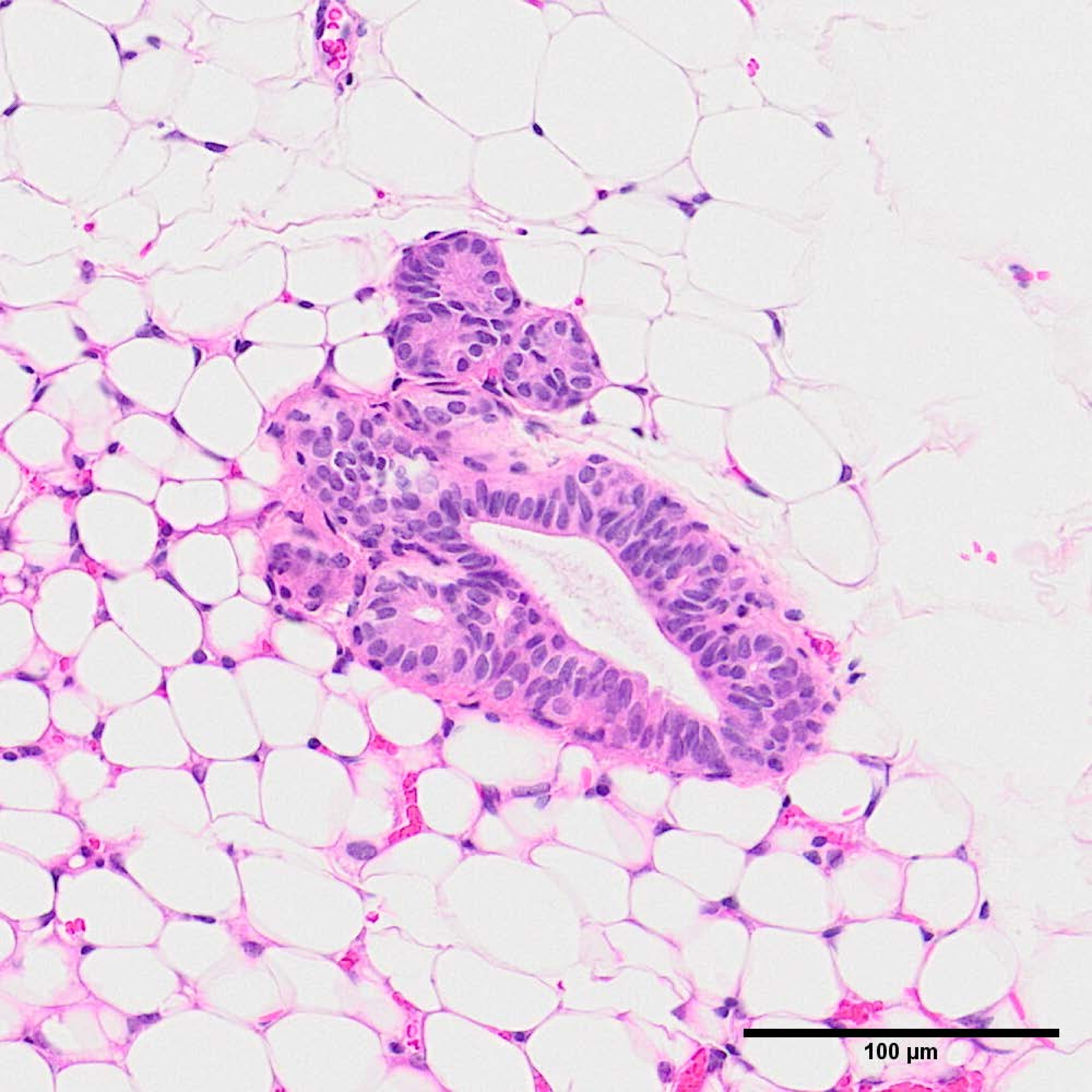
100 µm

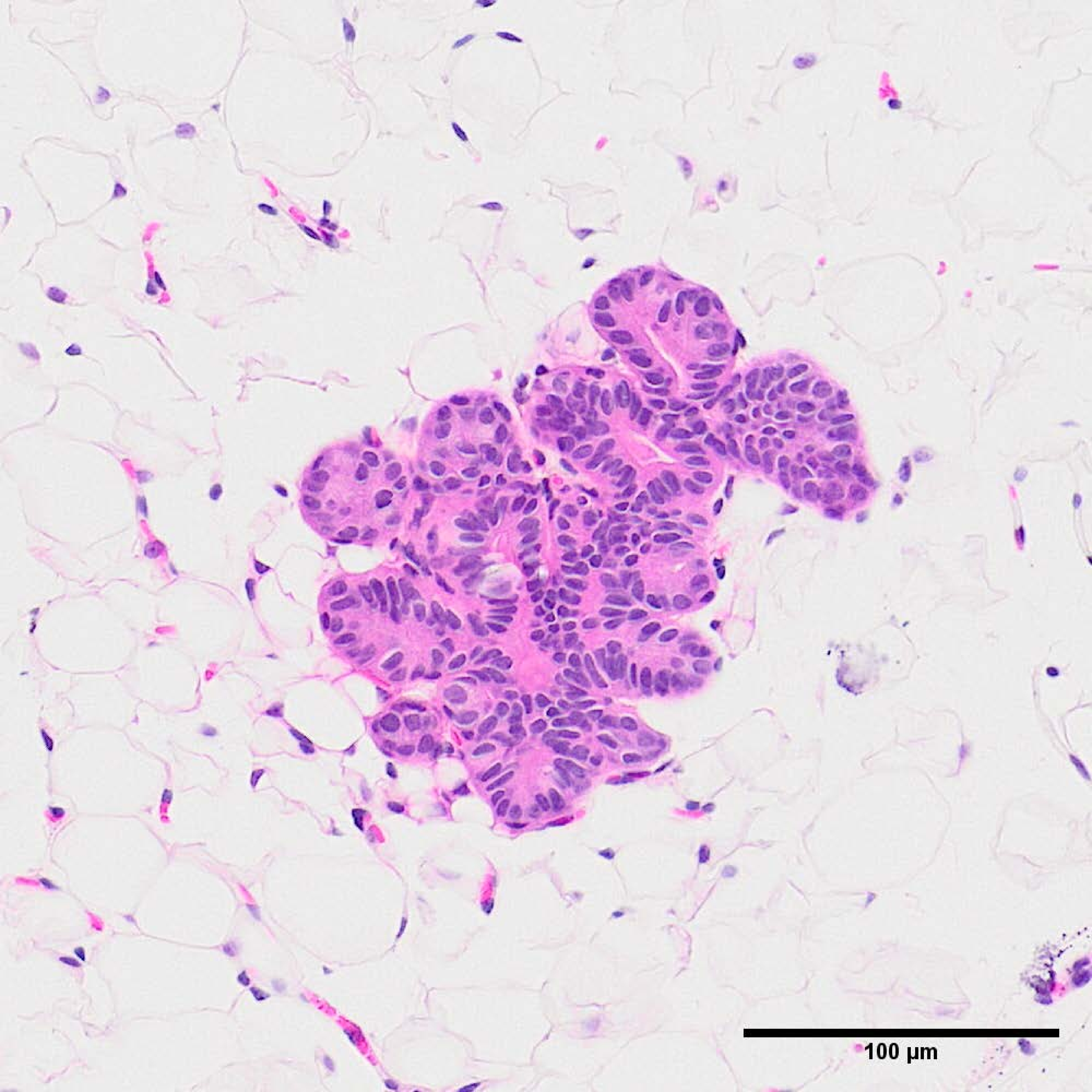
100 µm

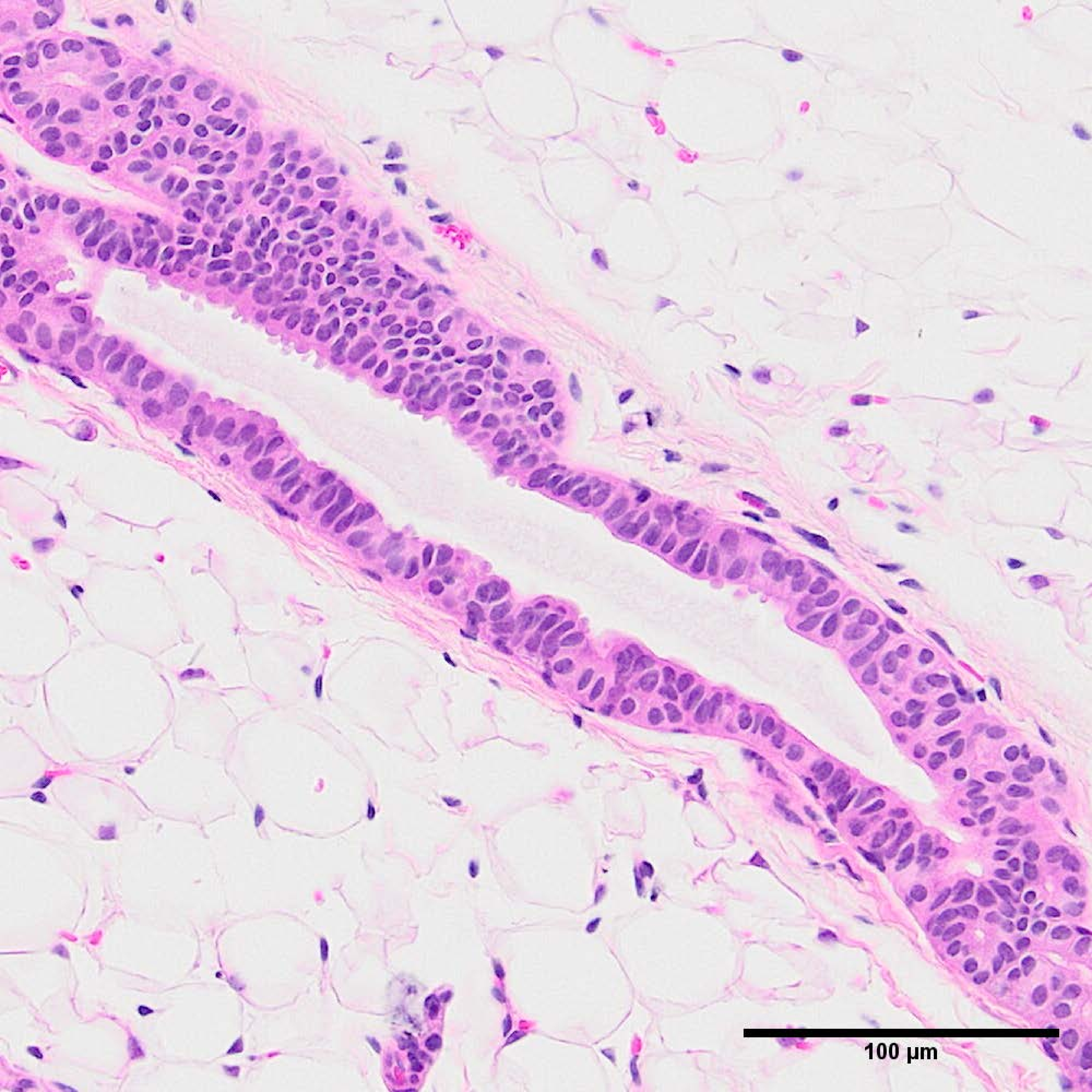
100 µm

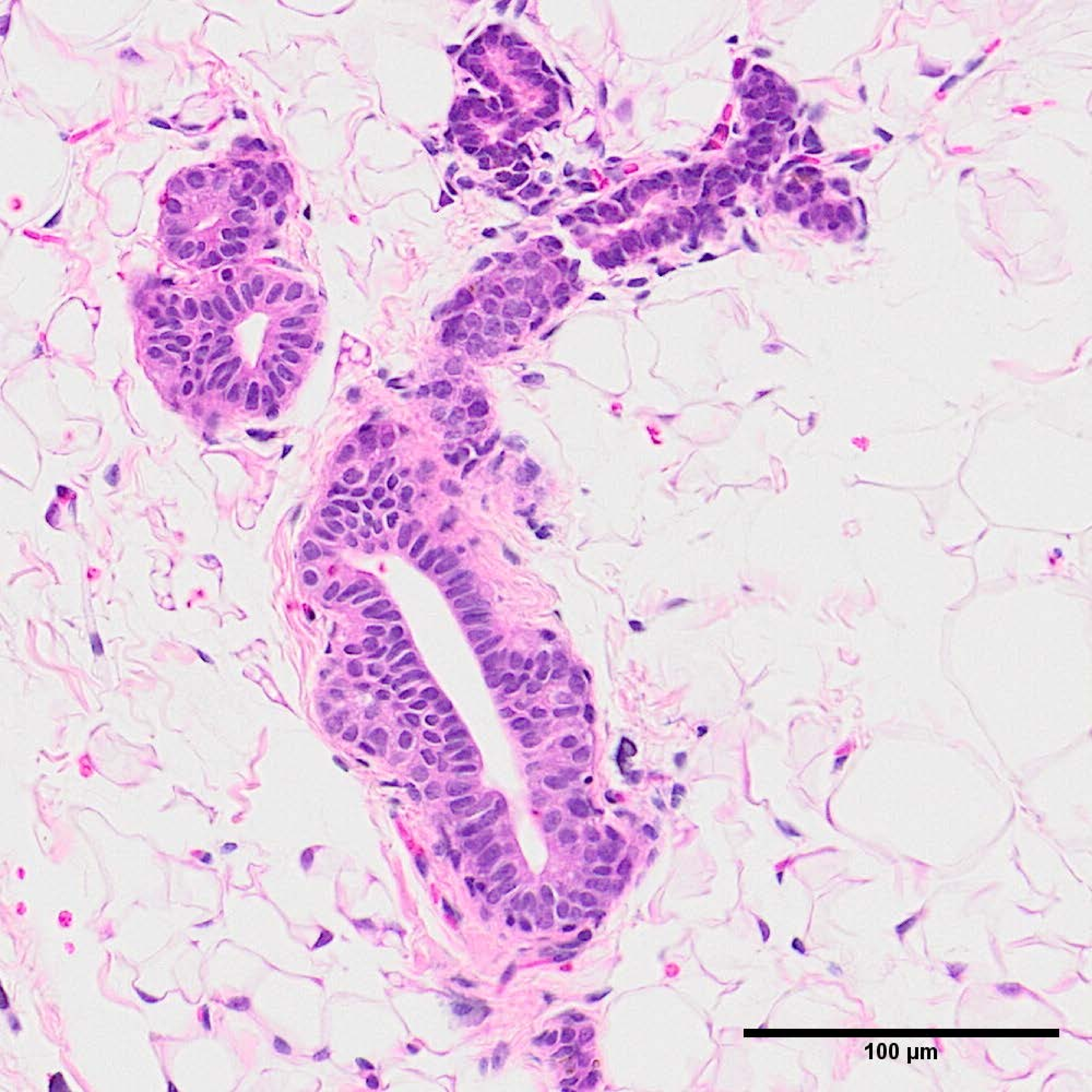
100 µm

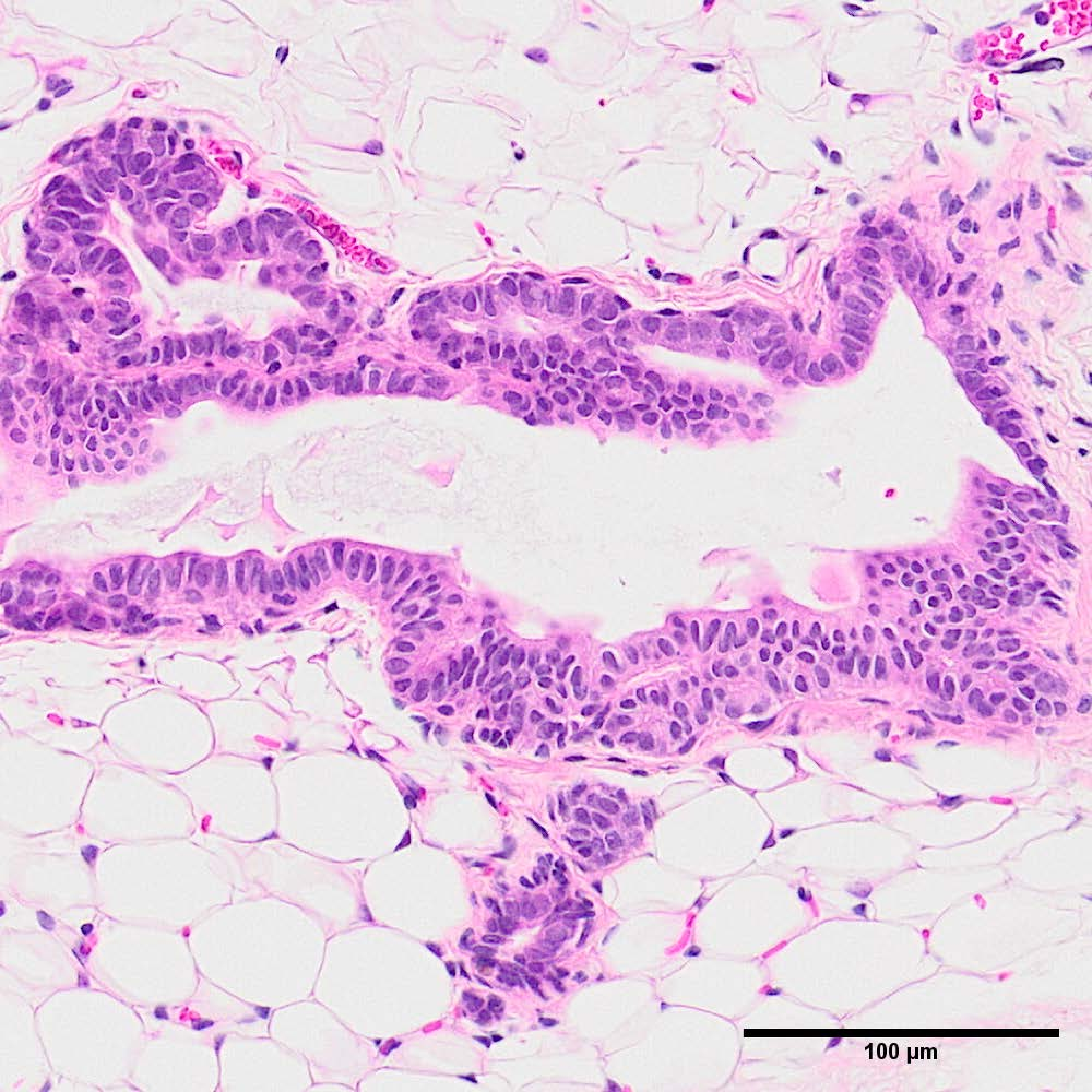
100 µm

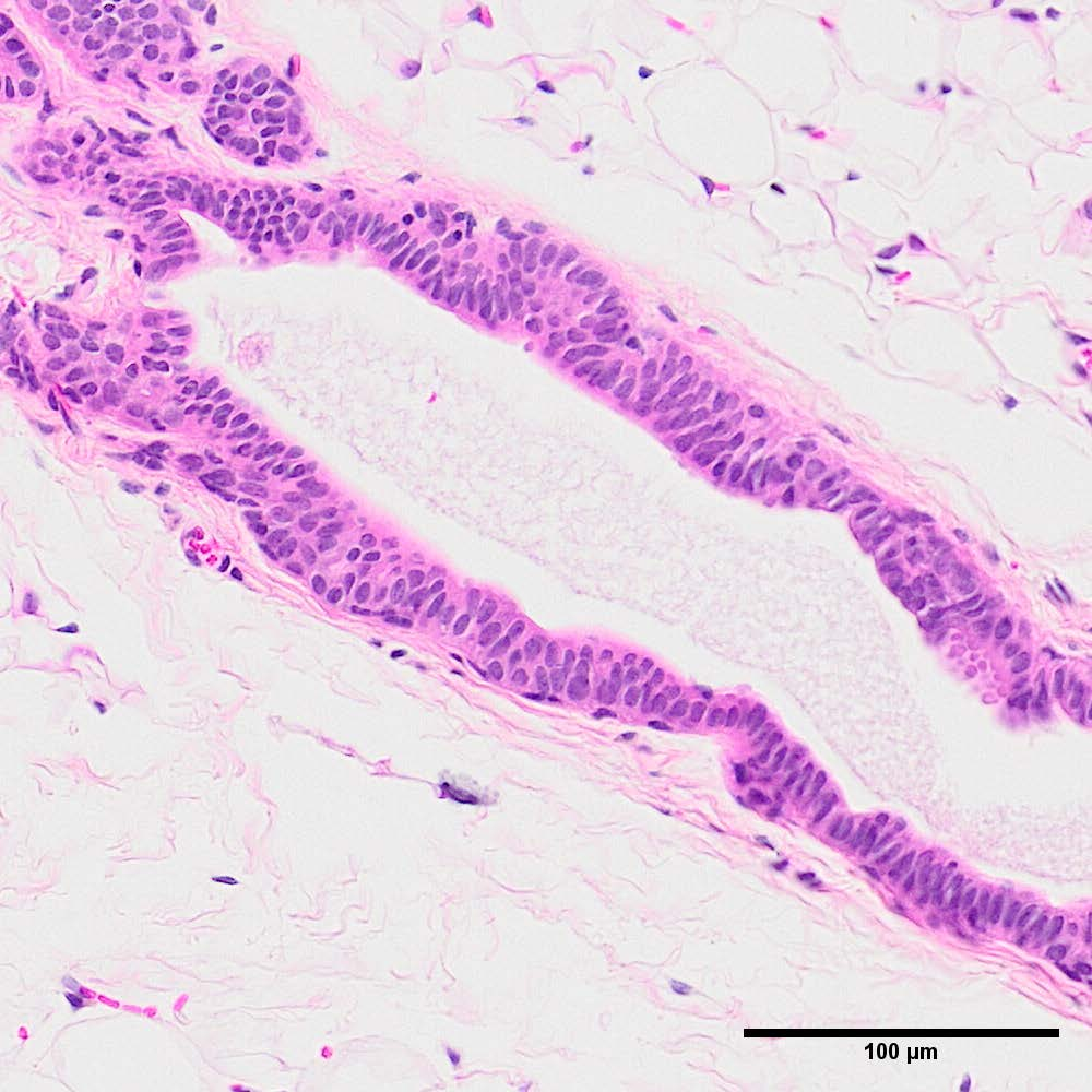
100 µm

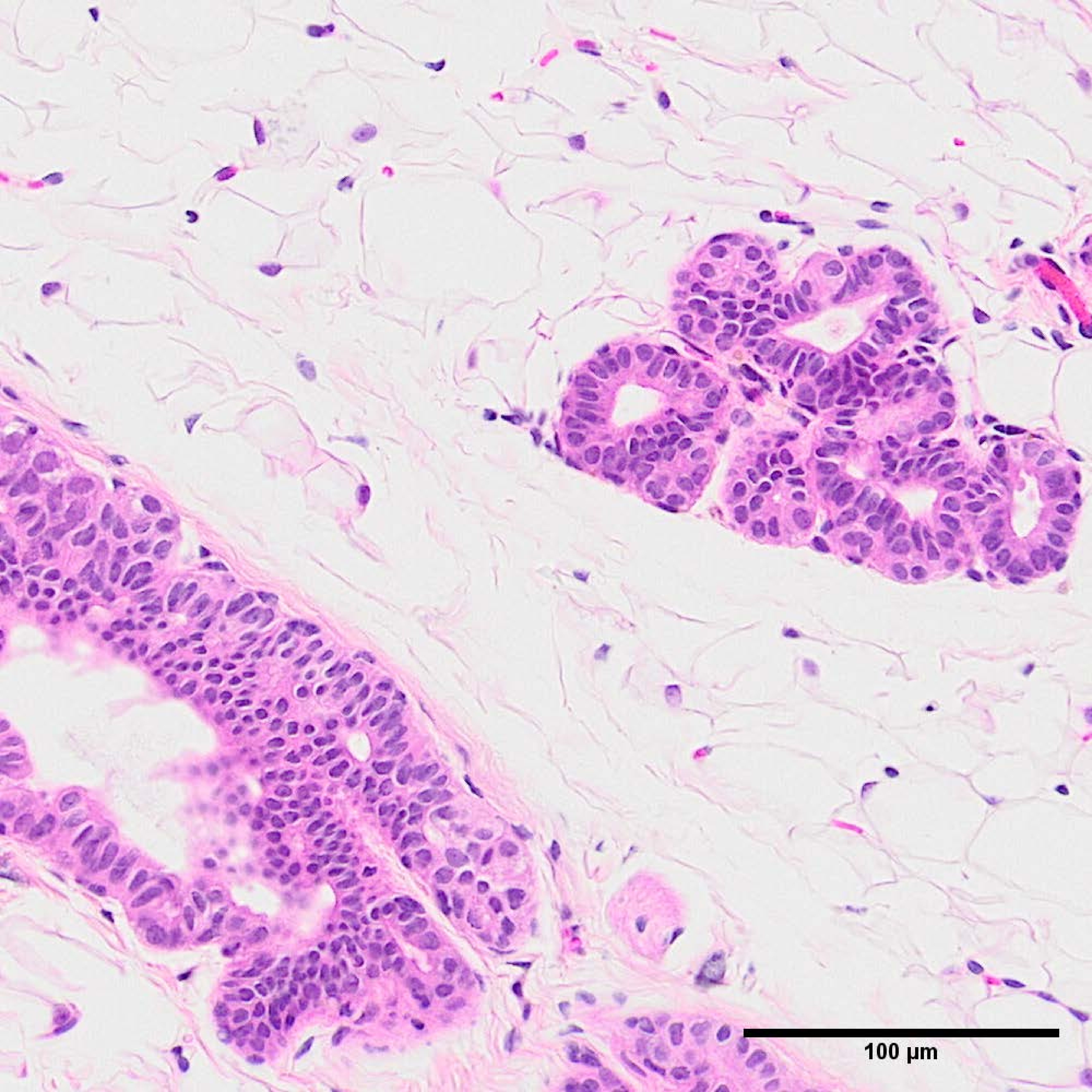
100 µm

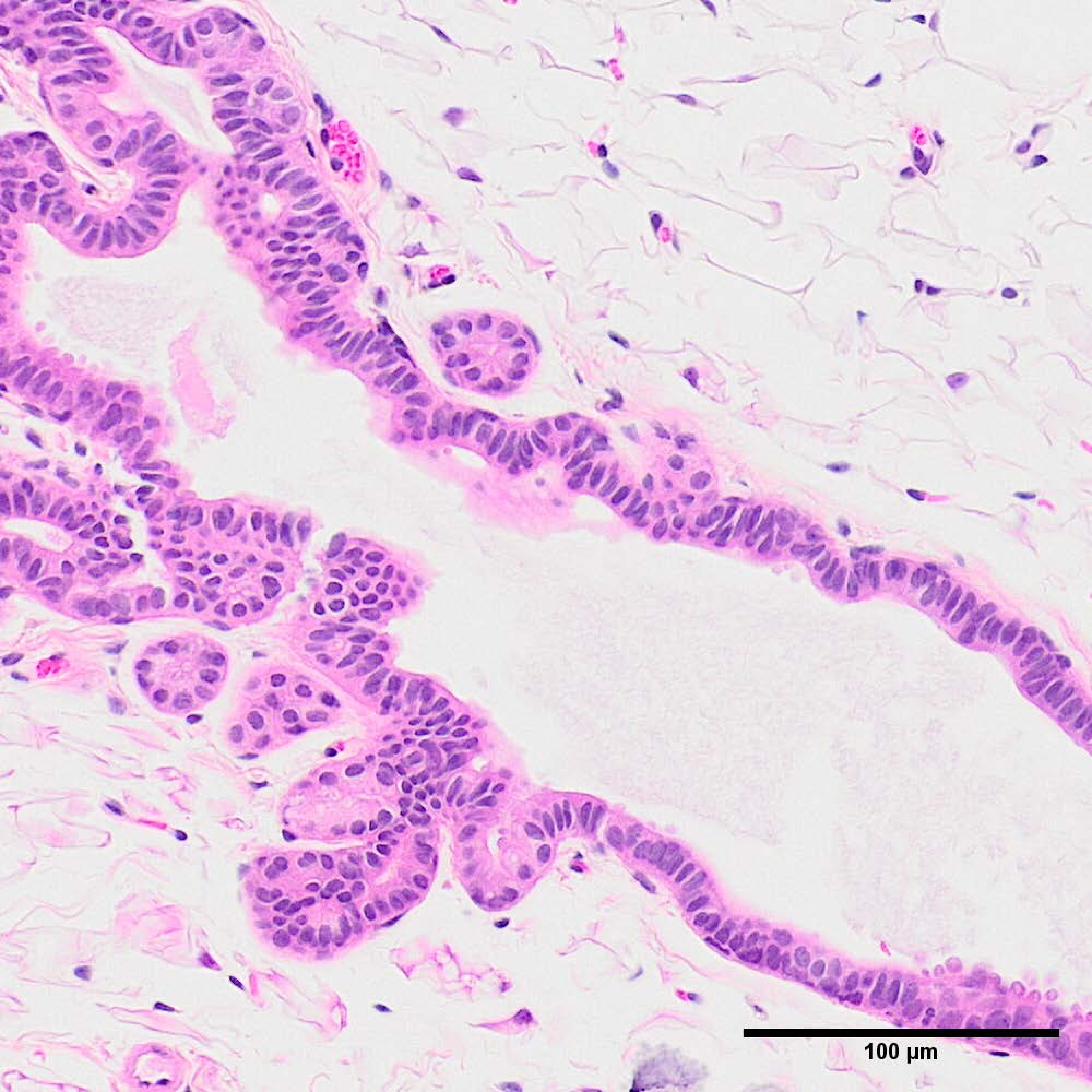
100 µm

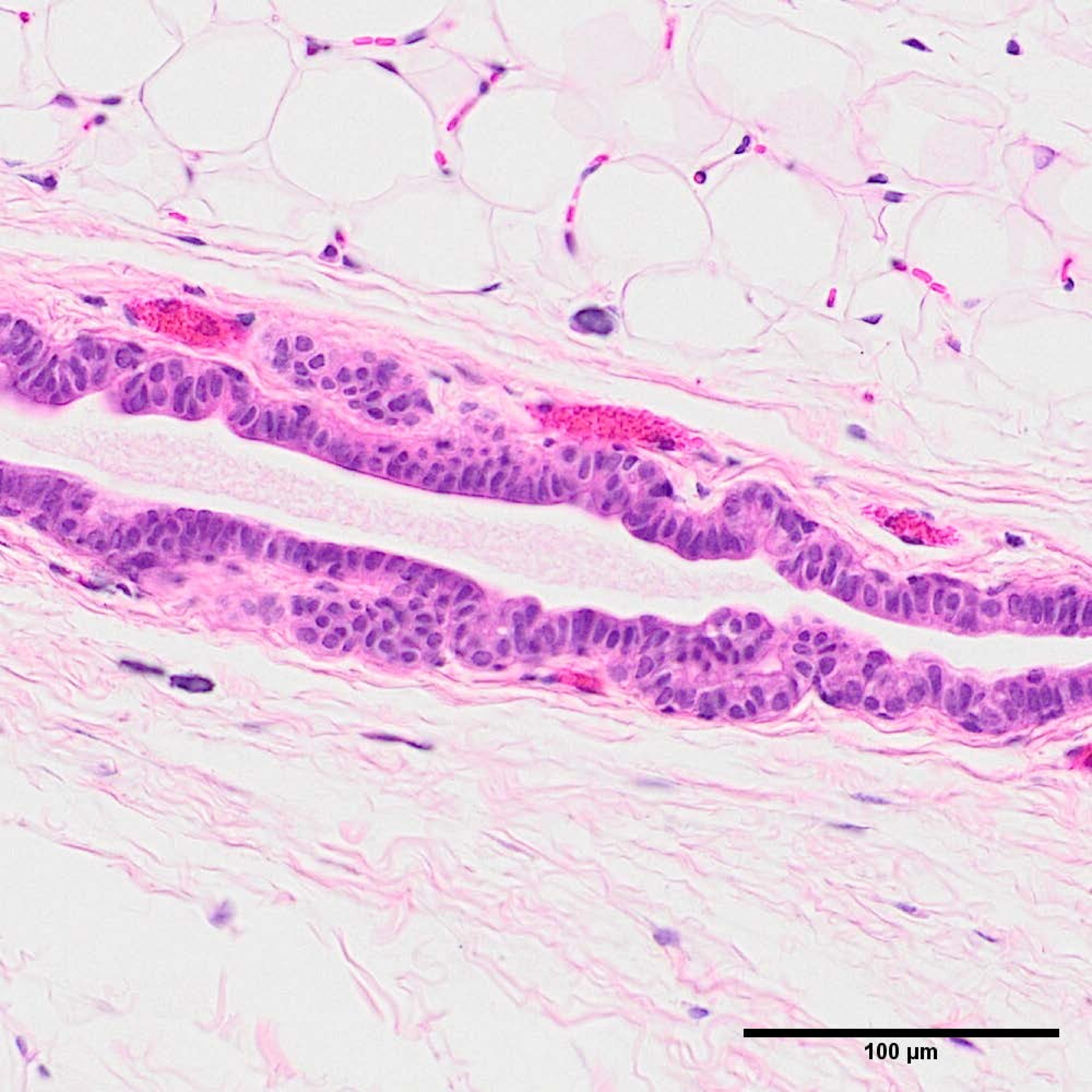
100 µm

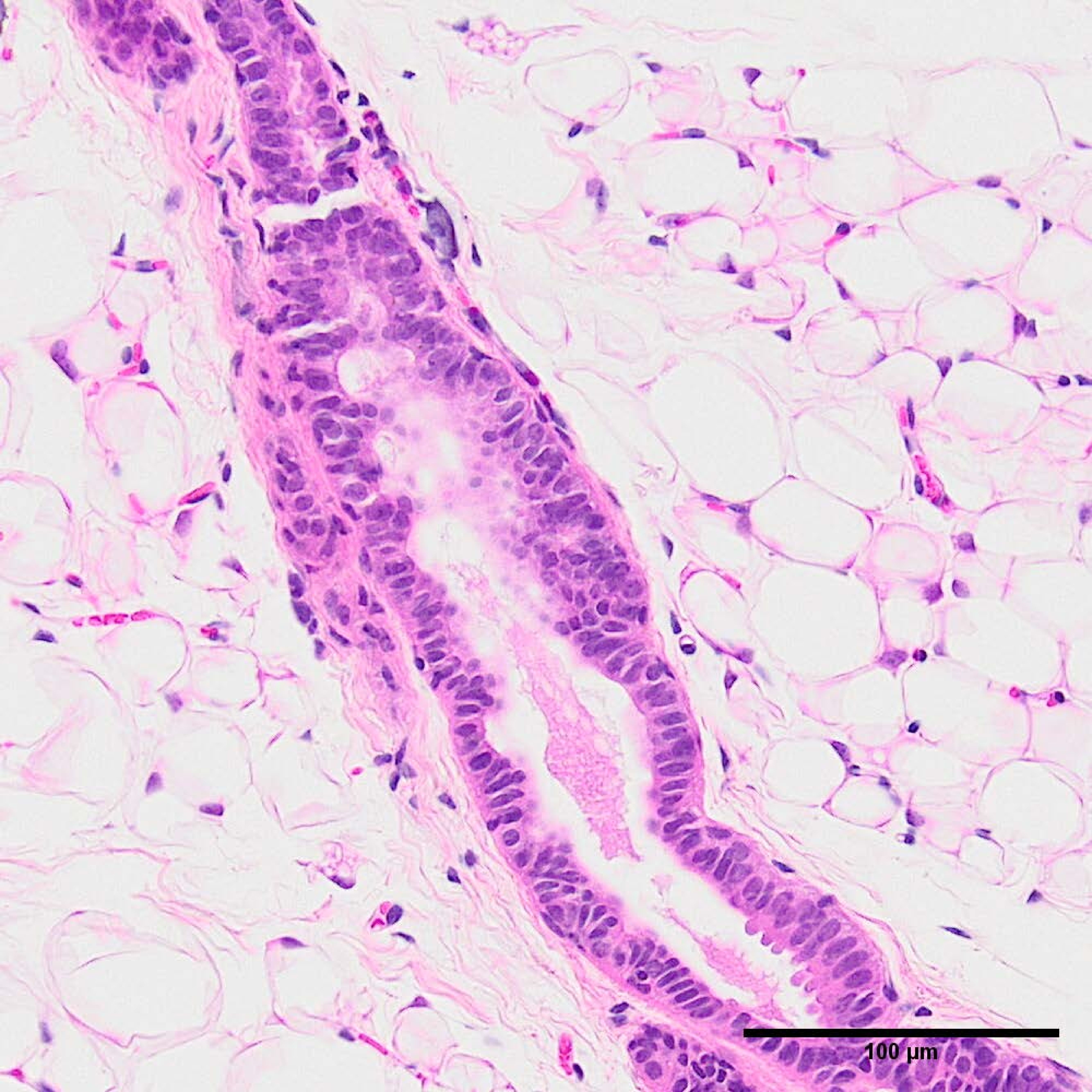
100 µm

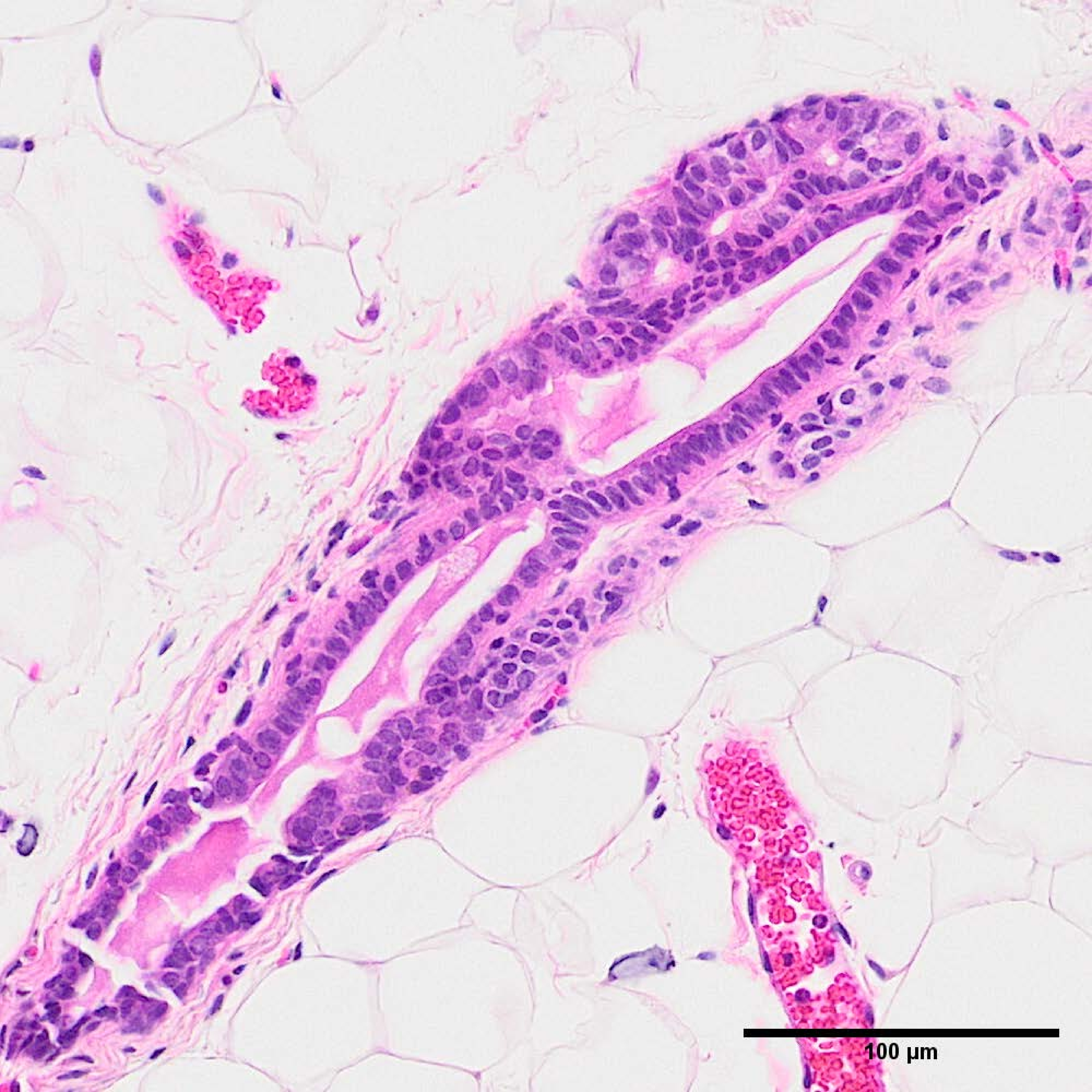
100 µm

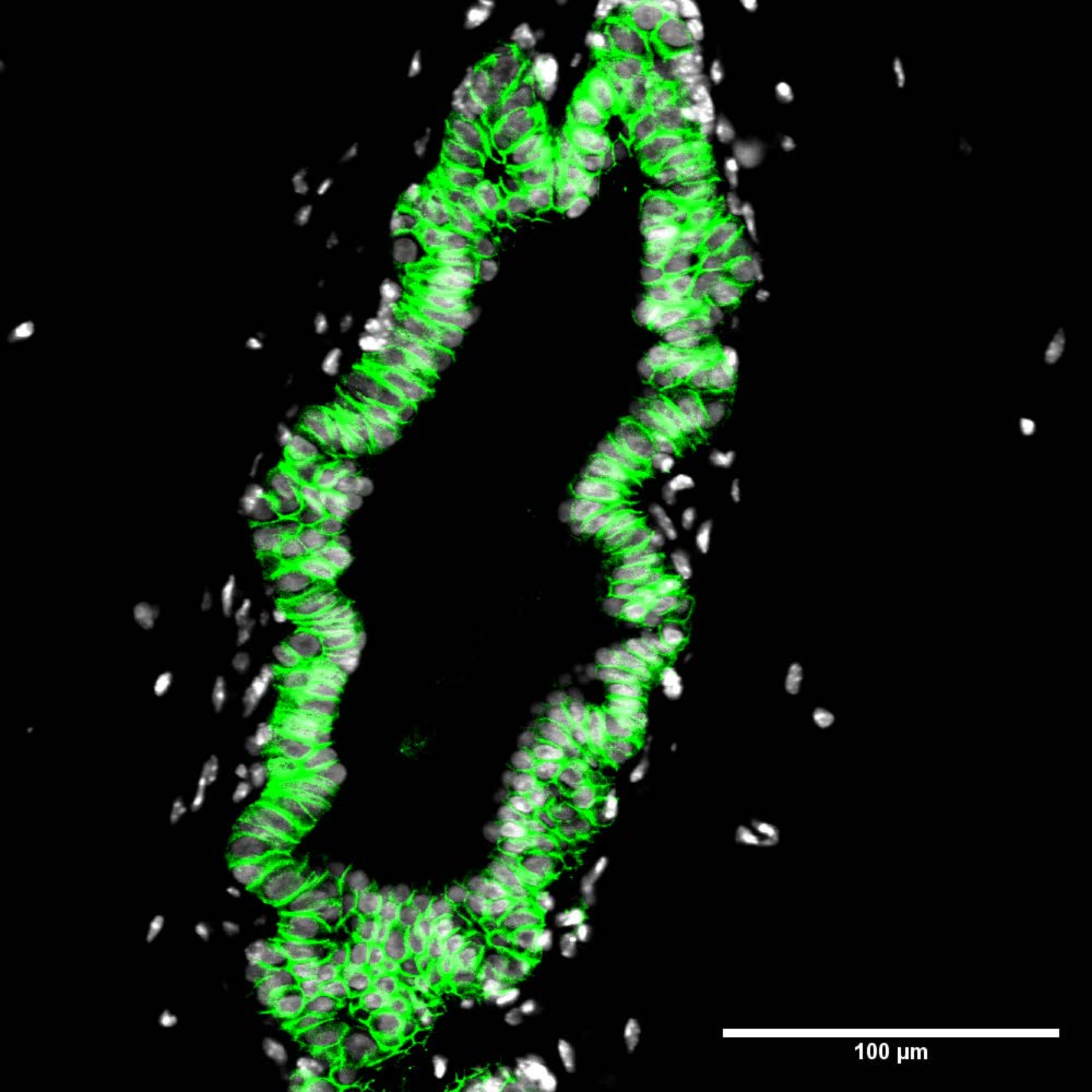
100 µm

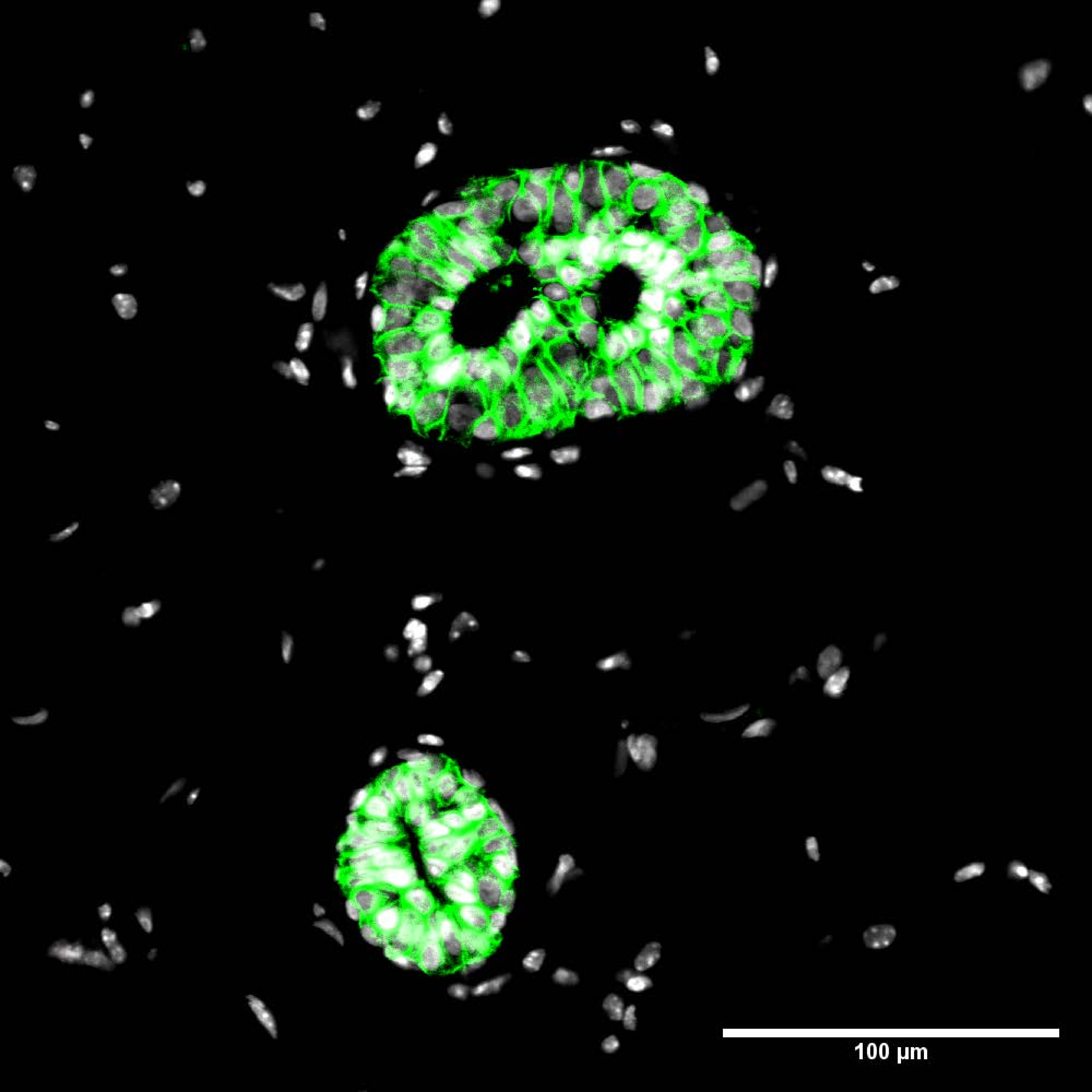
100 µm

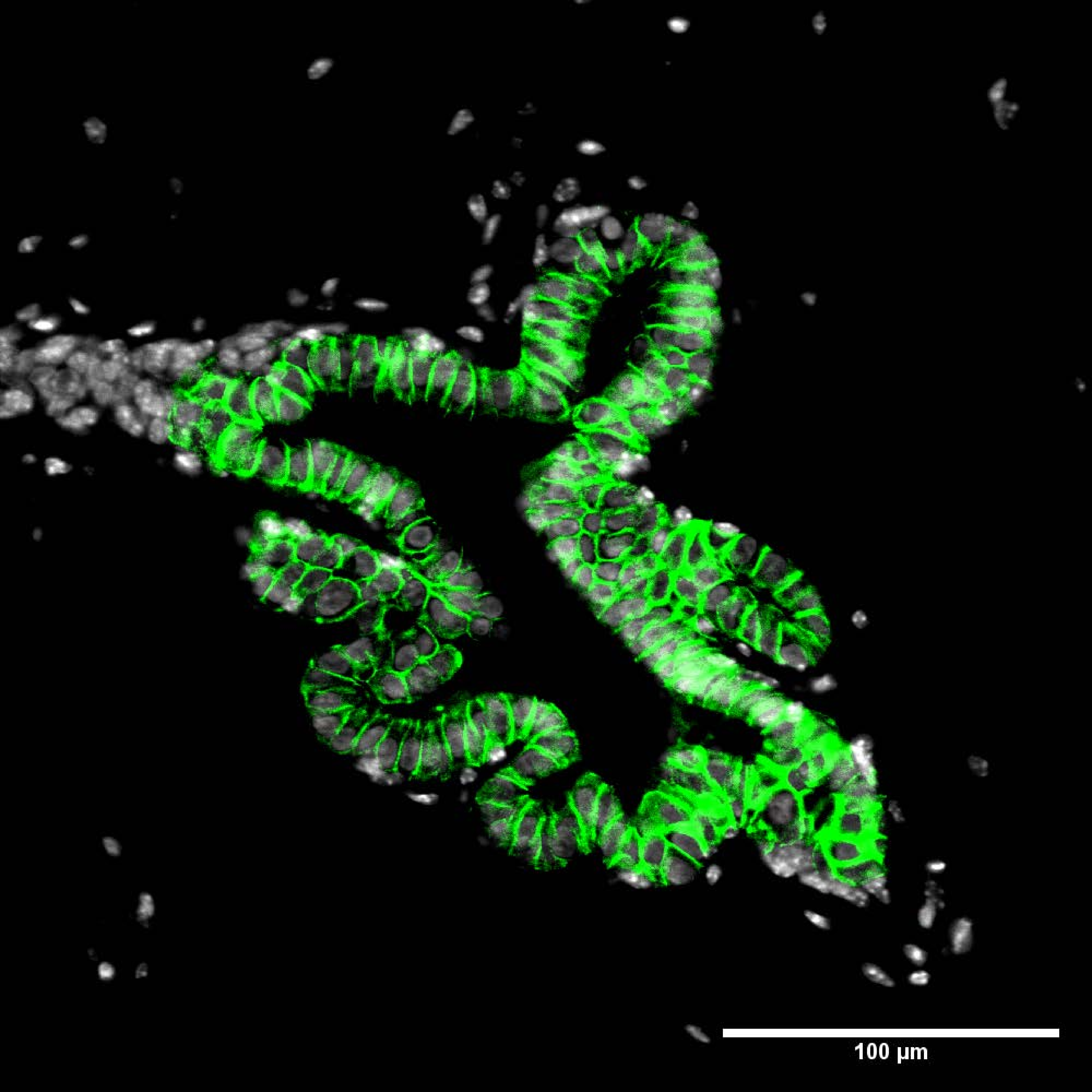
100 µm

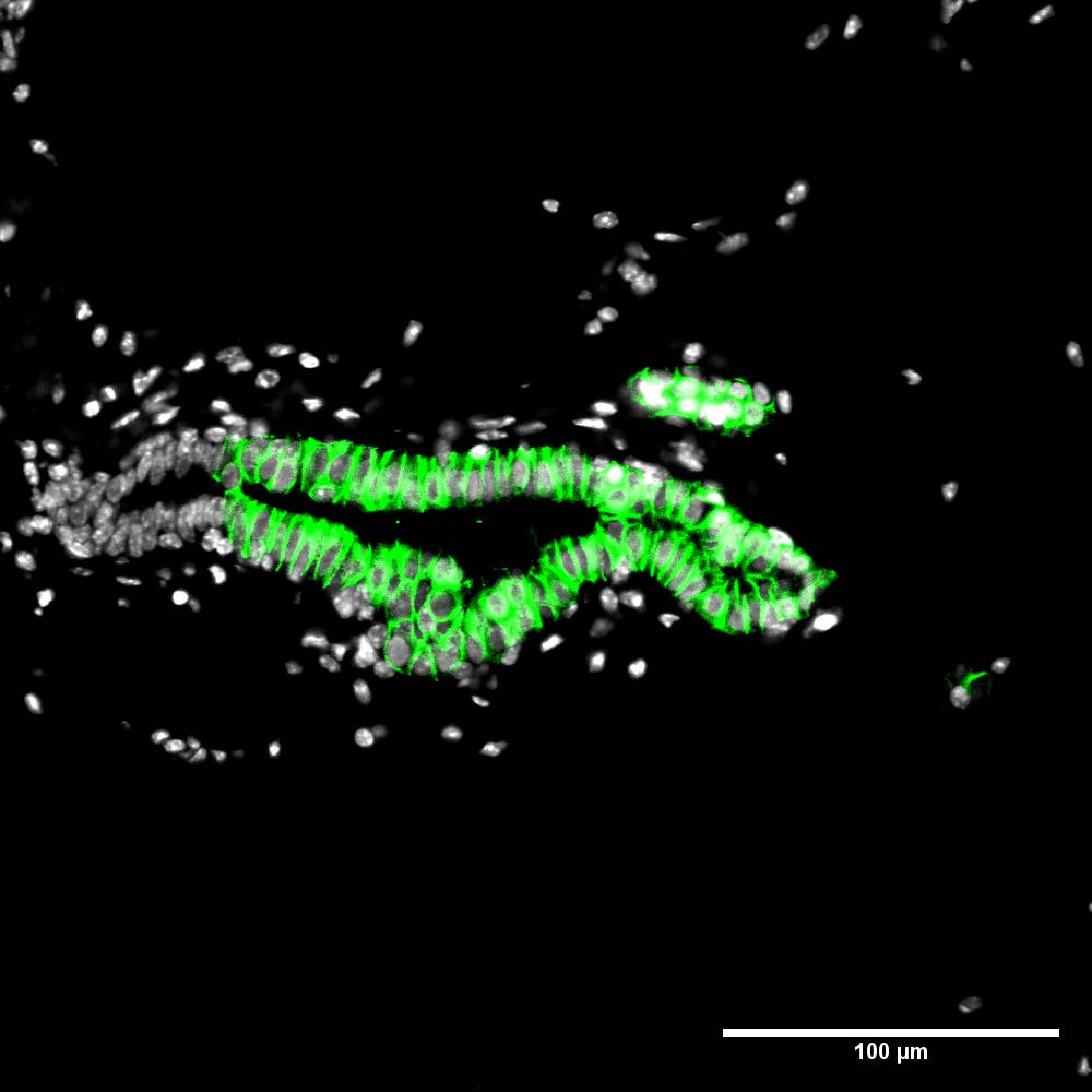
100 µm

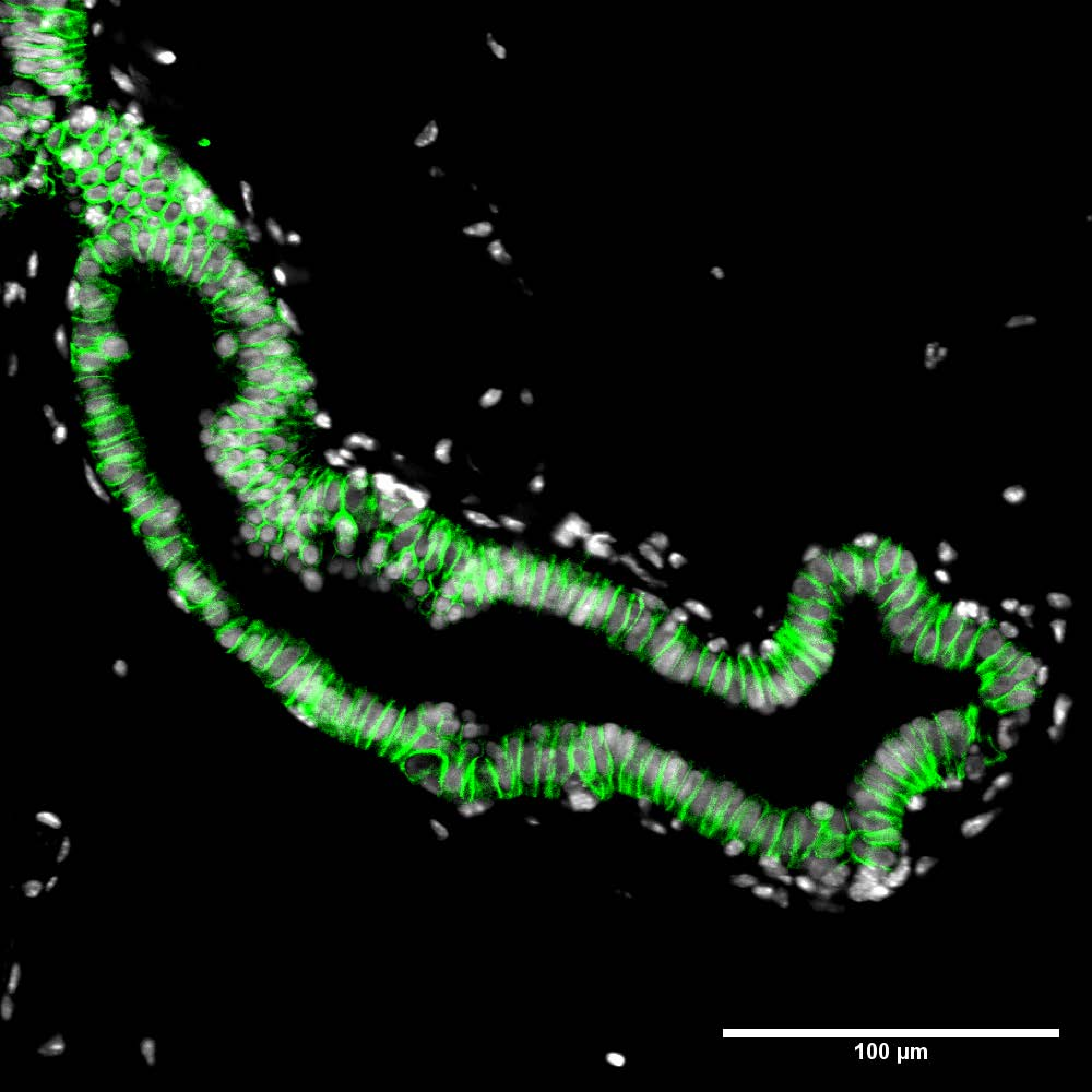
100 µm

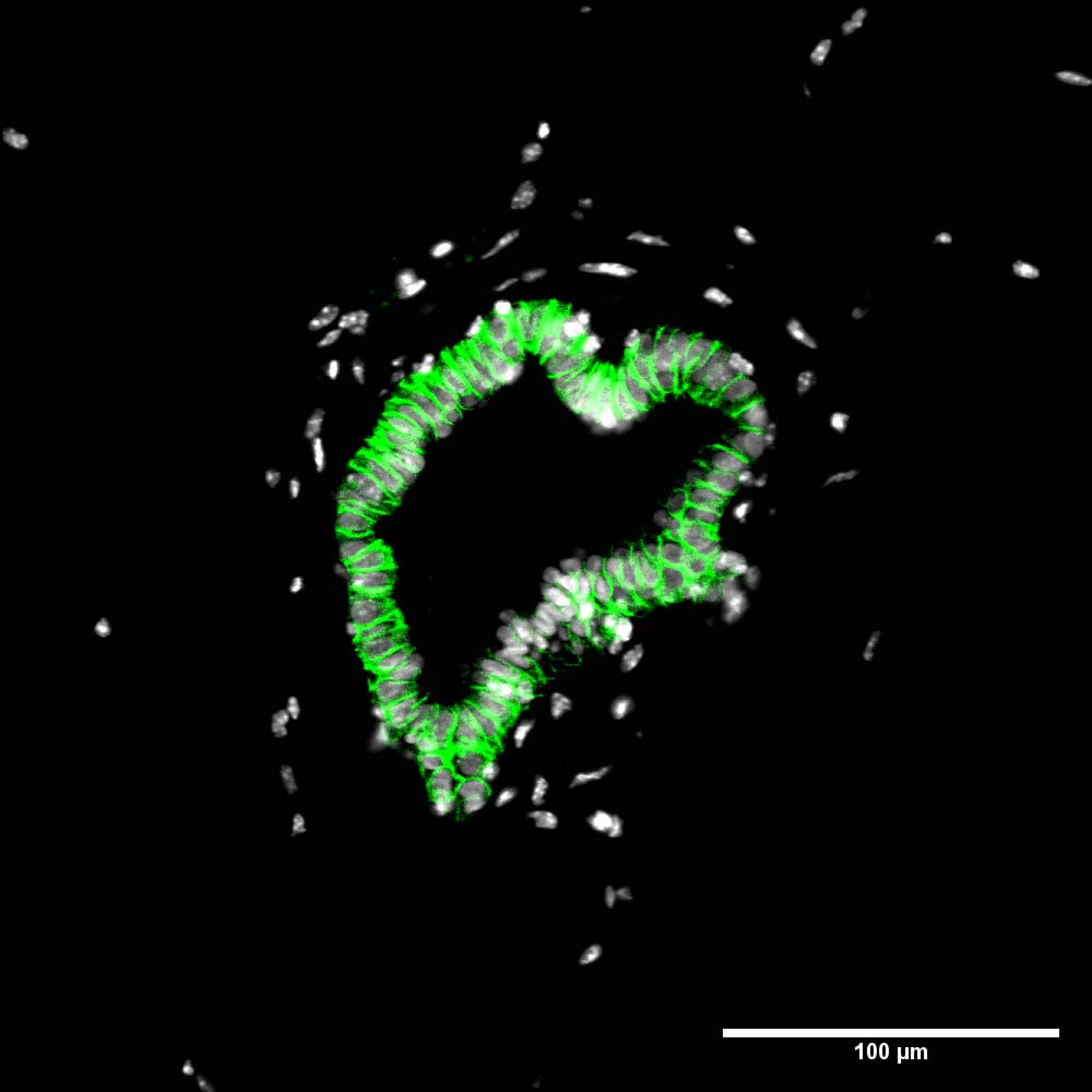
100 µm

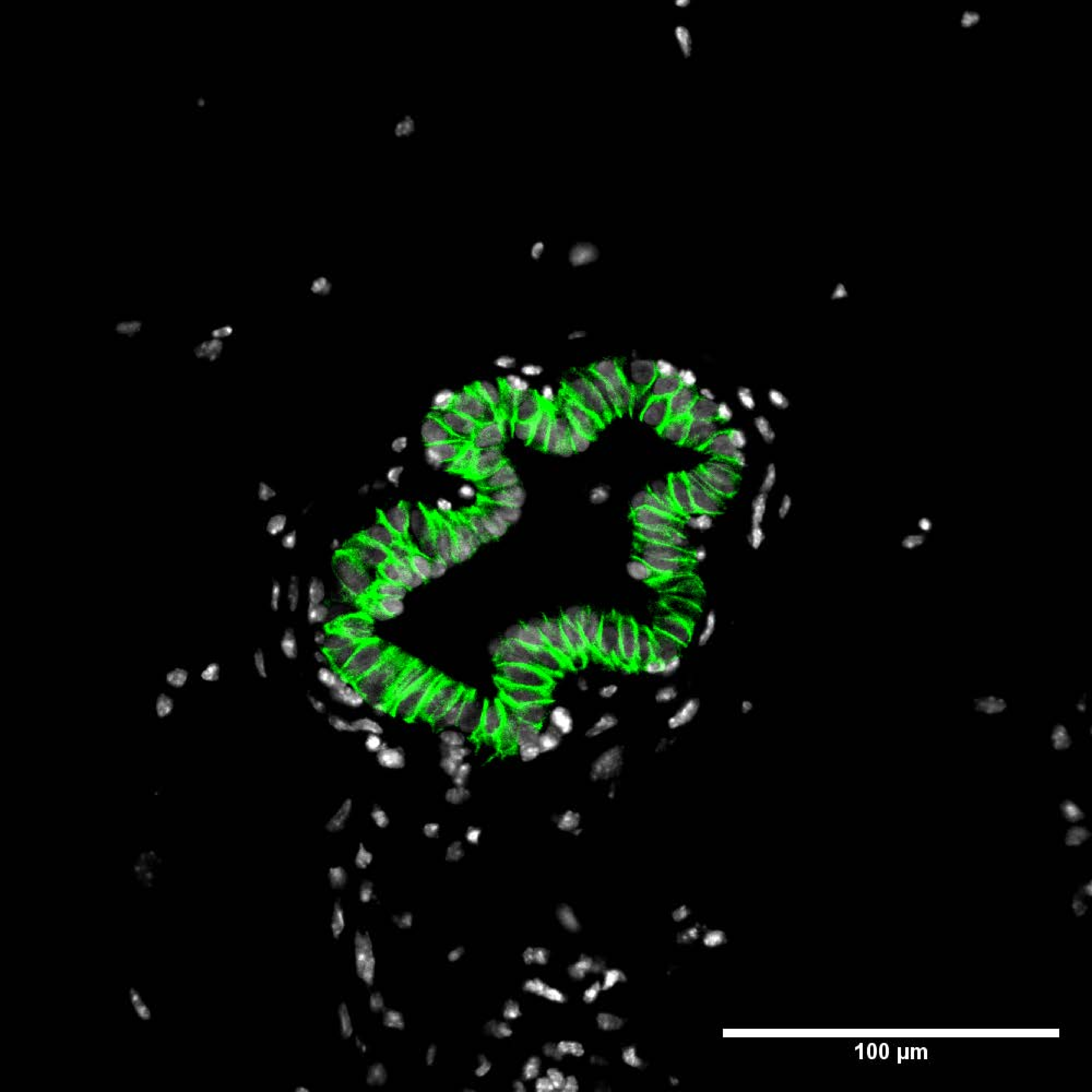
100 µm

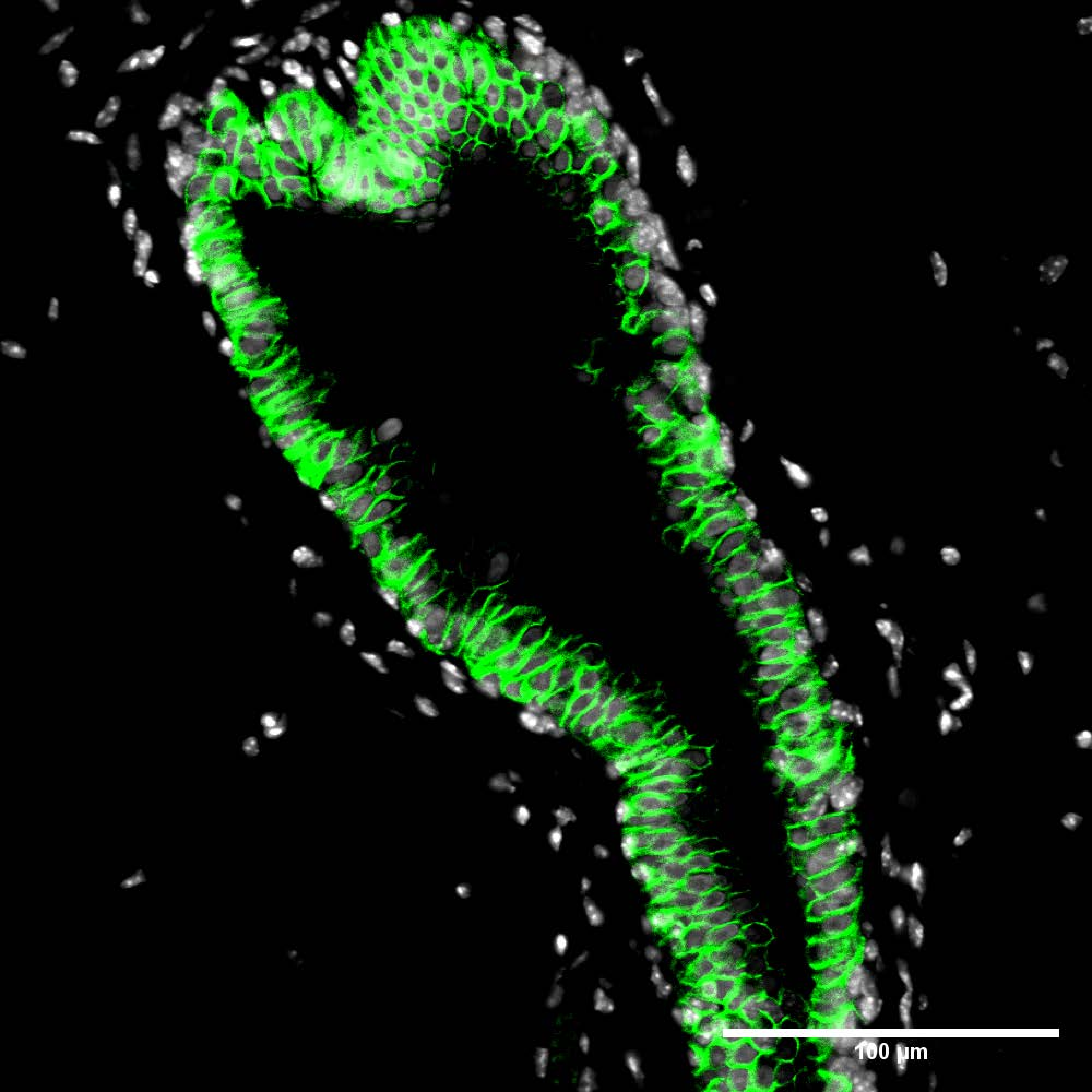
100 µm

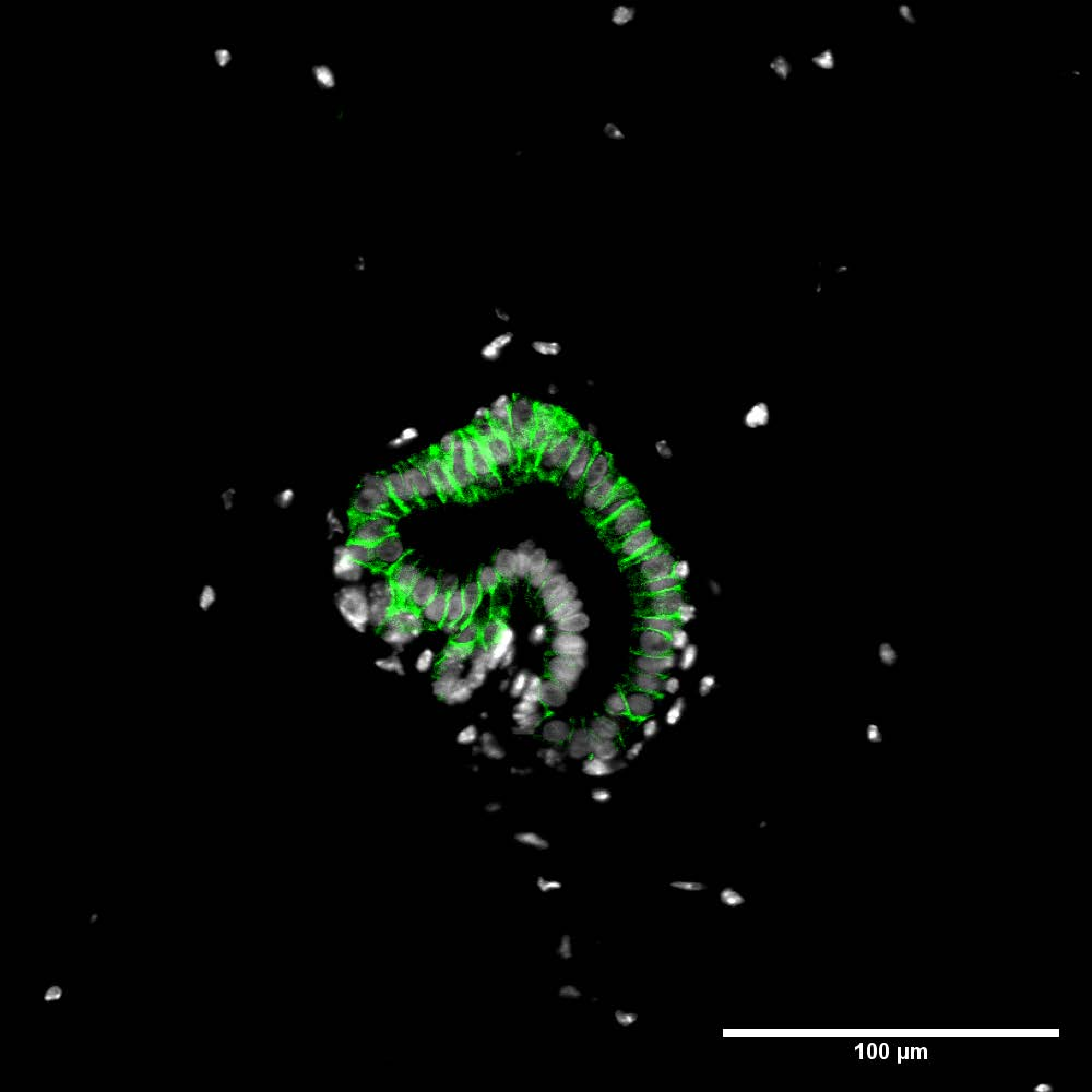
100 µm

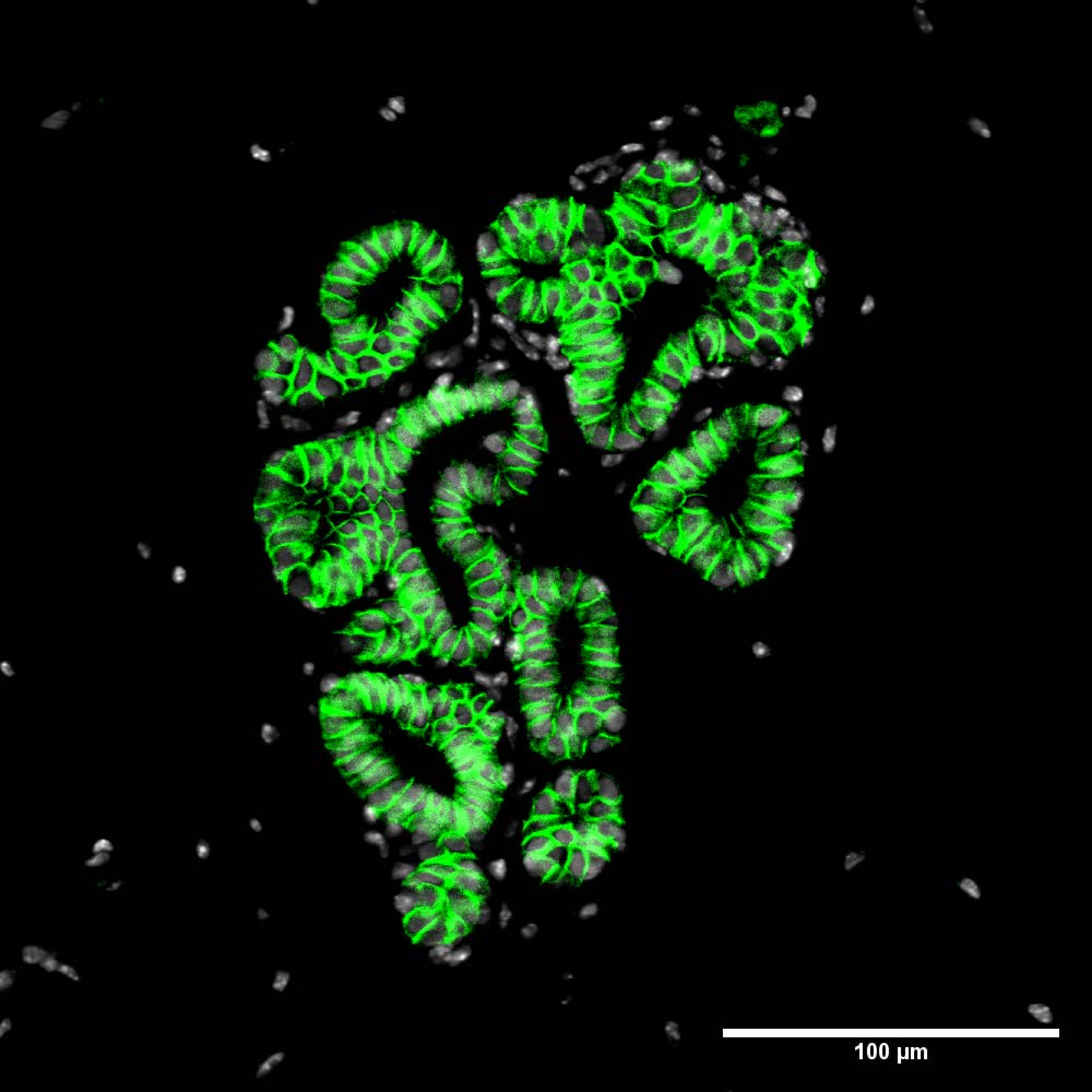
100 µm

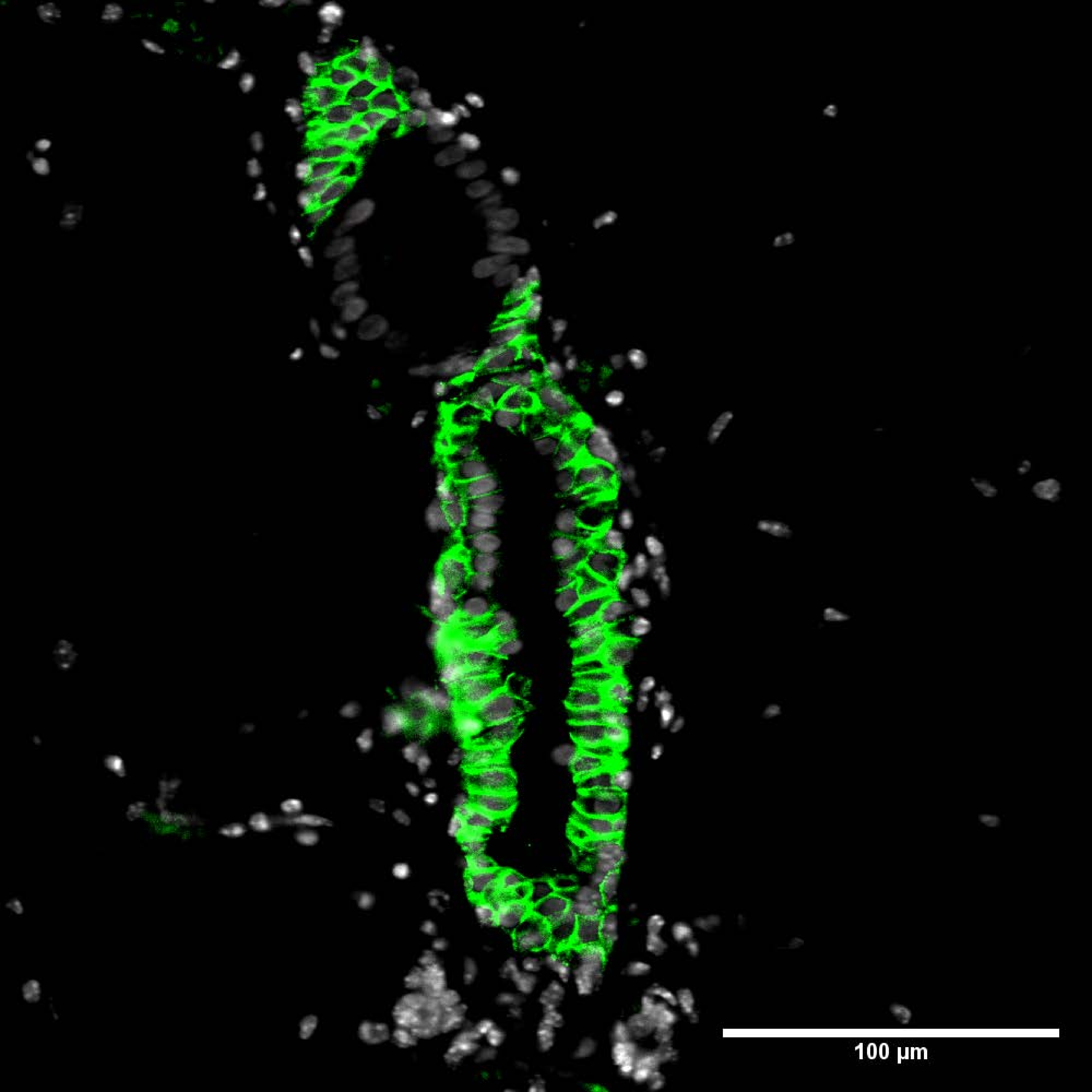
100 µm

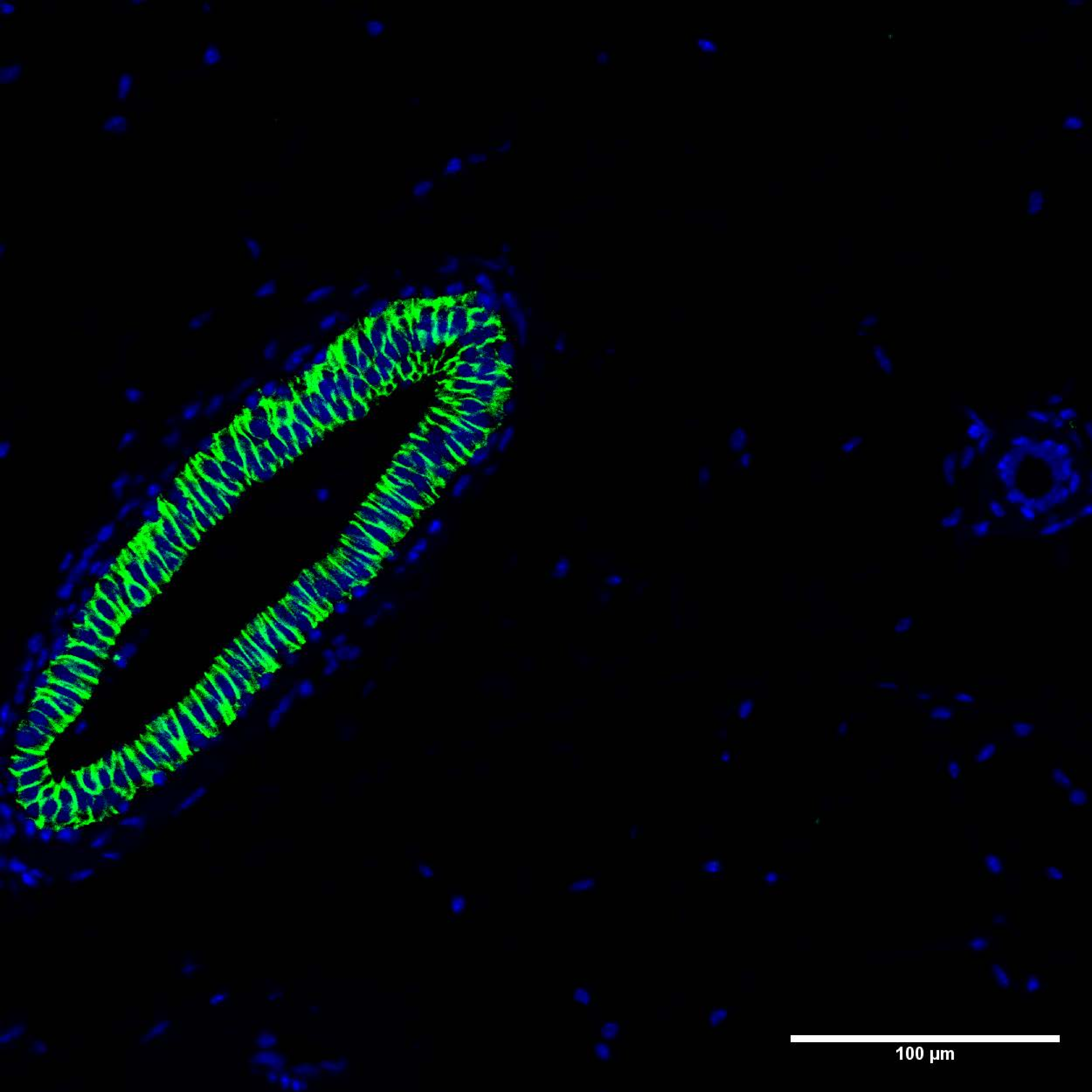
100 µm

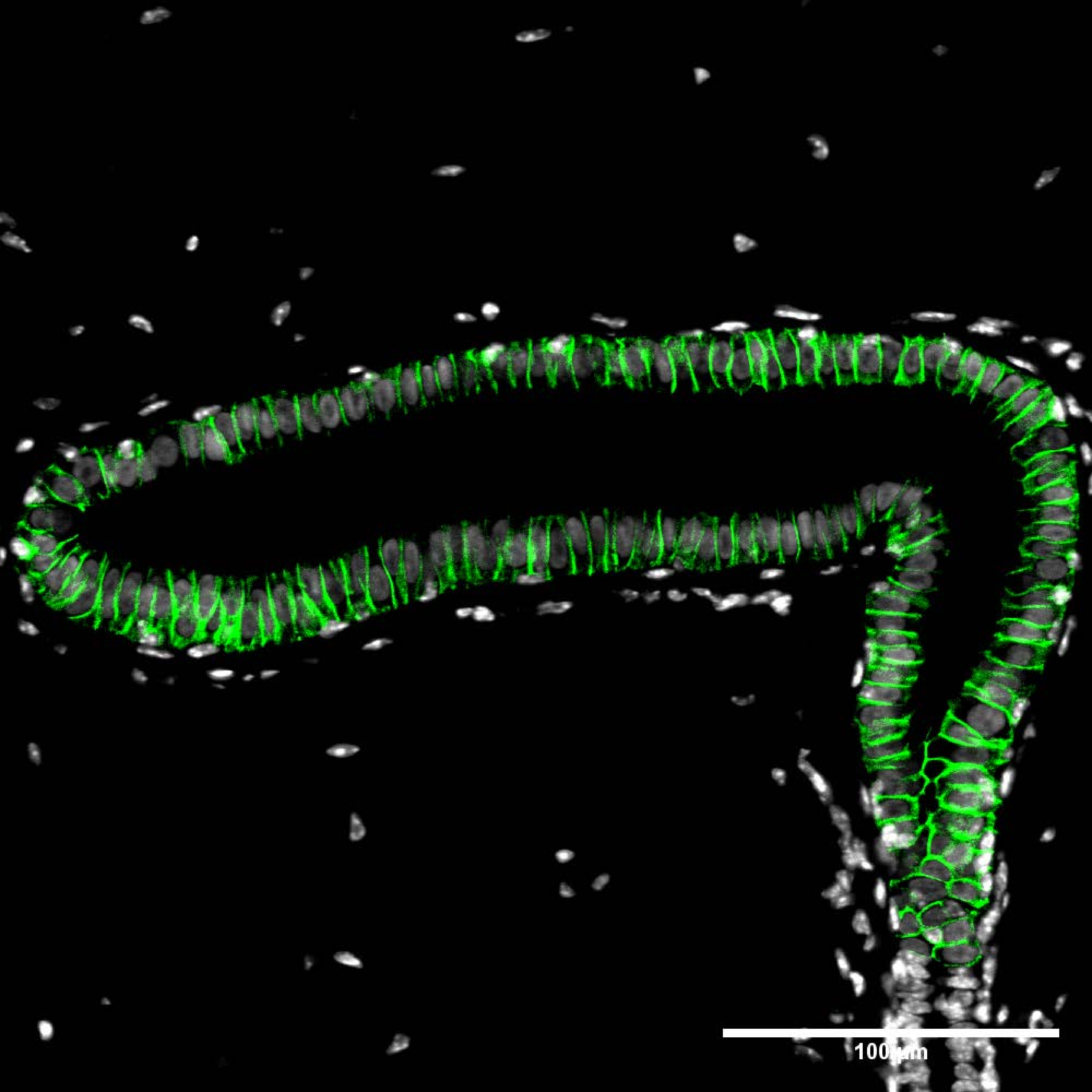
100 µm

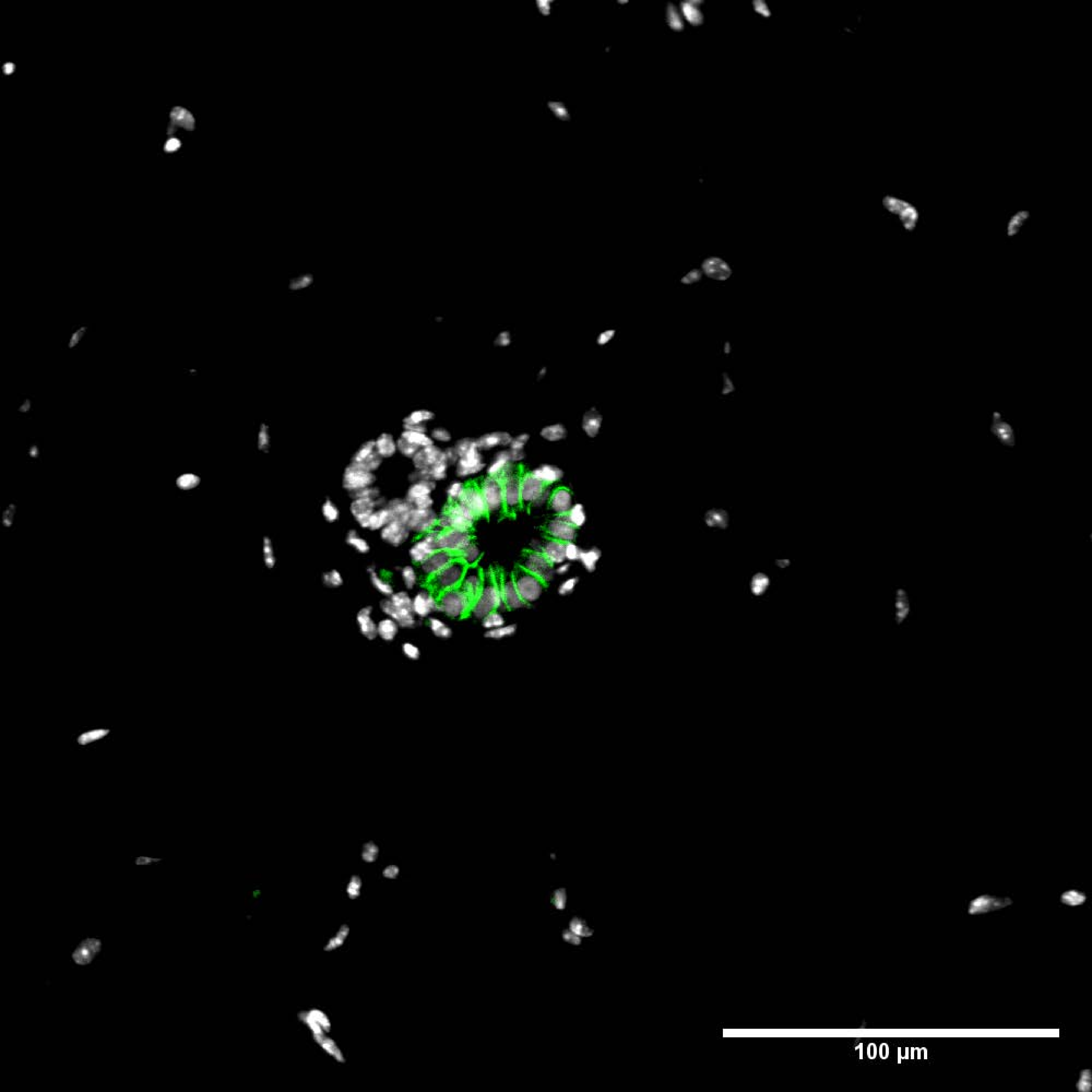
100 µm

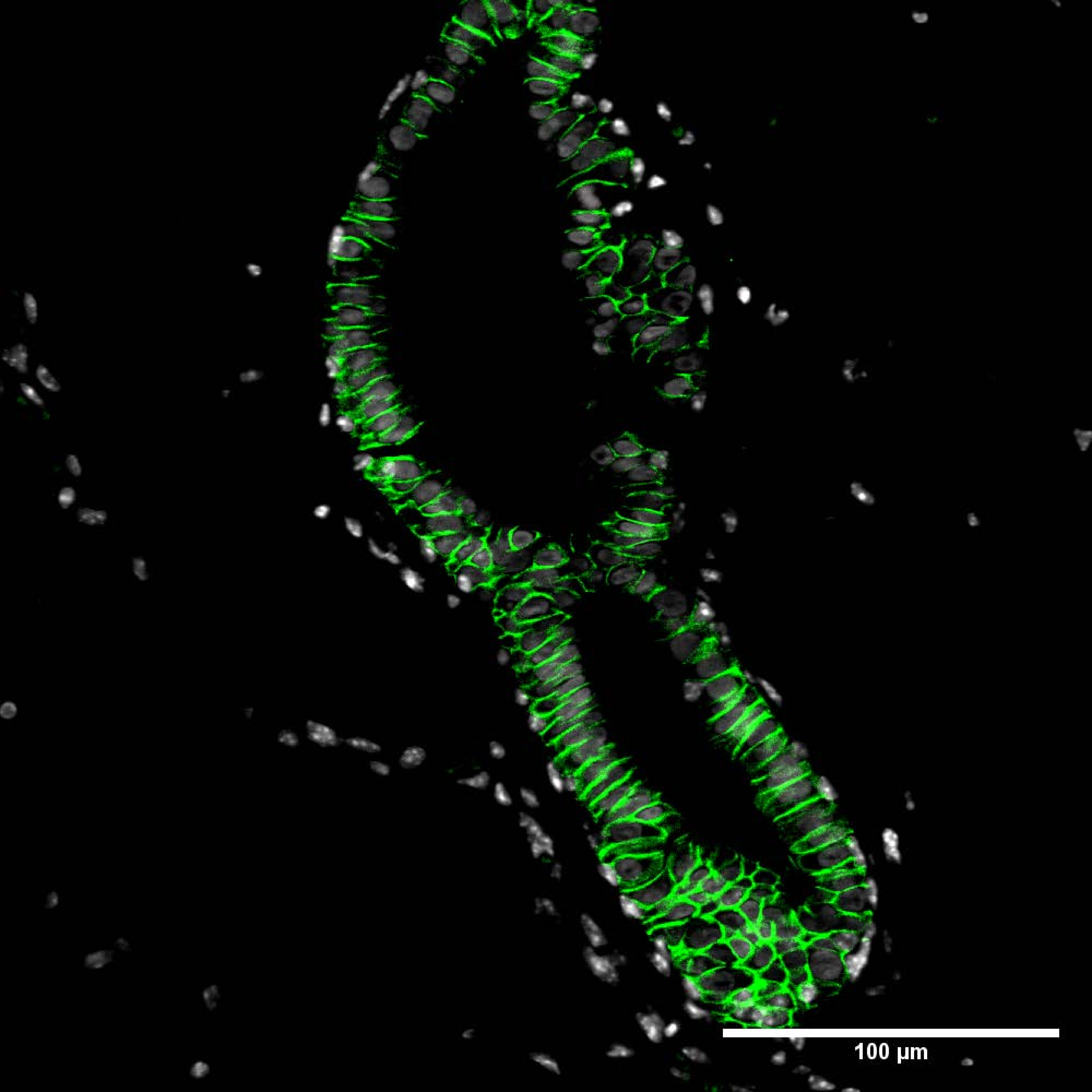
100 µm

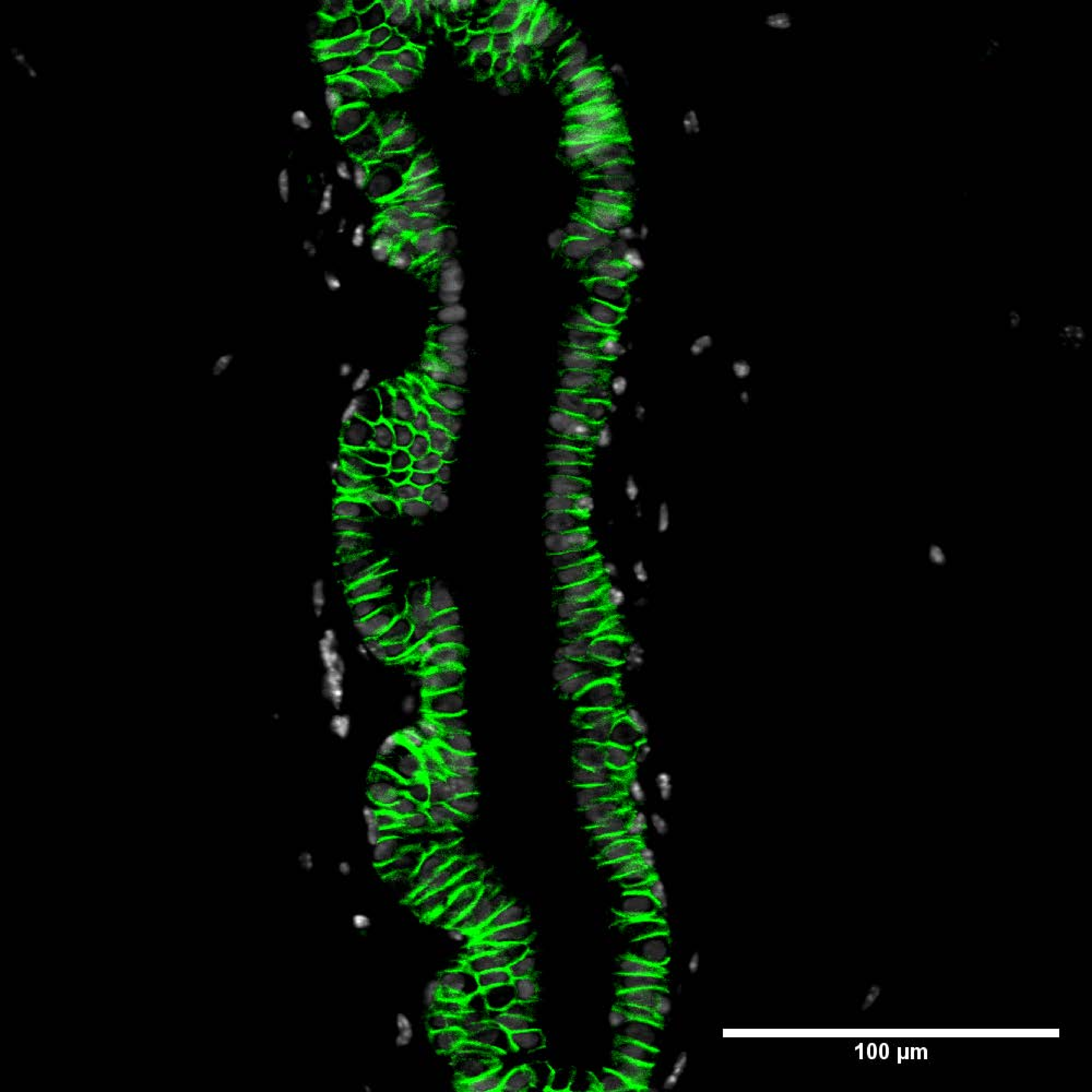
100 µm

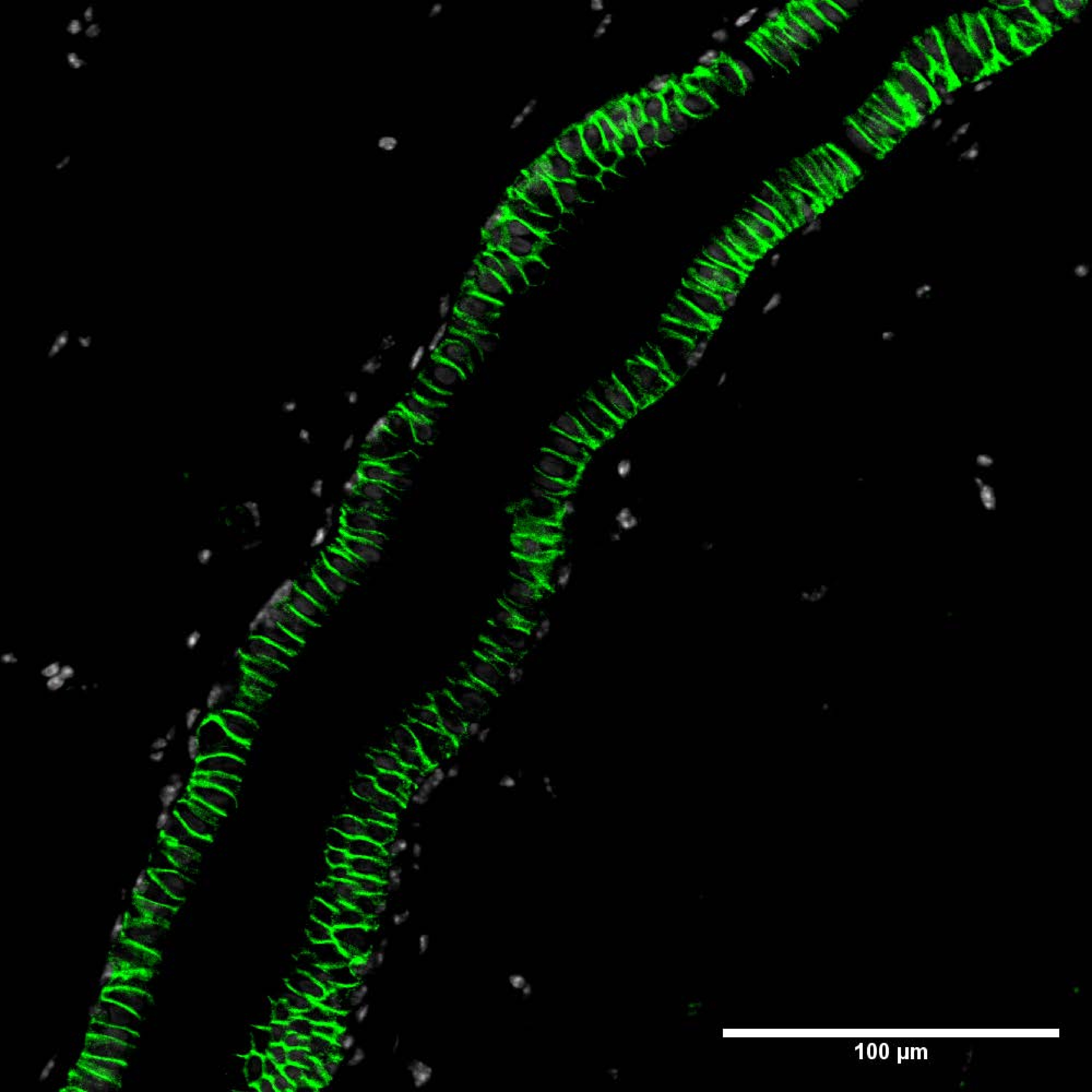
100 µm

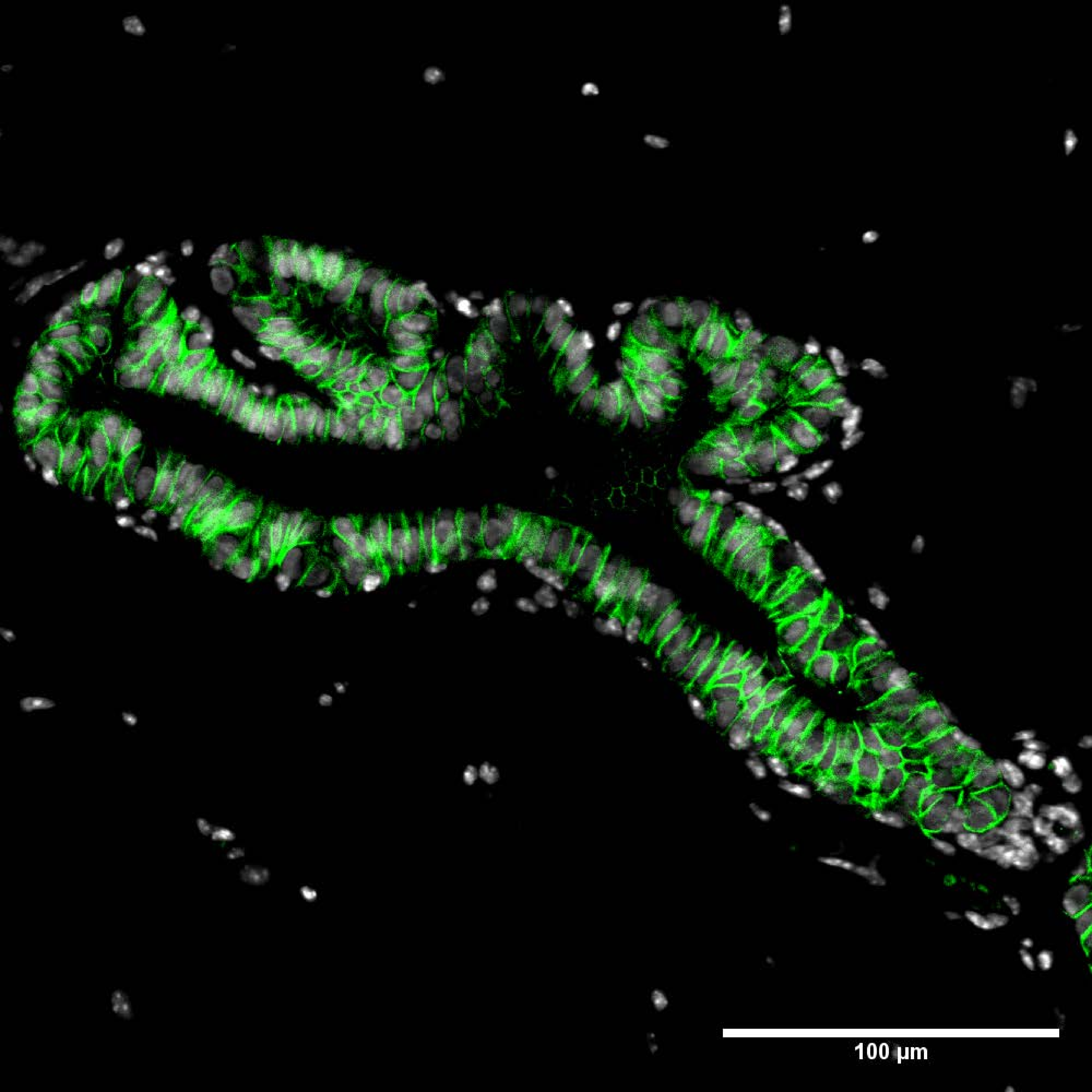
100 µm

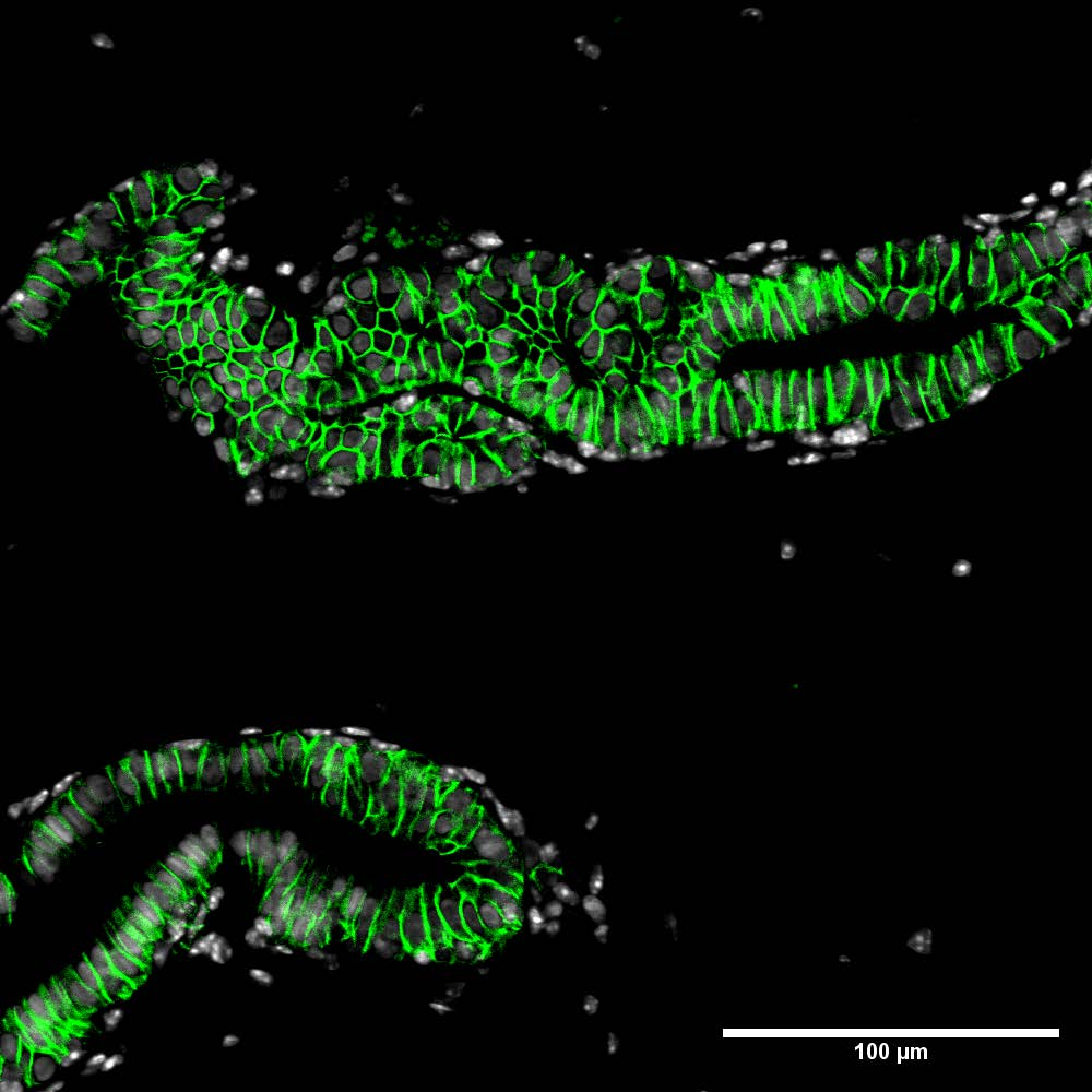
100 µm

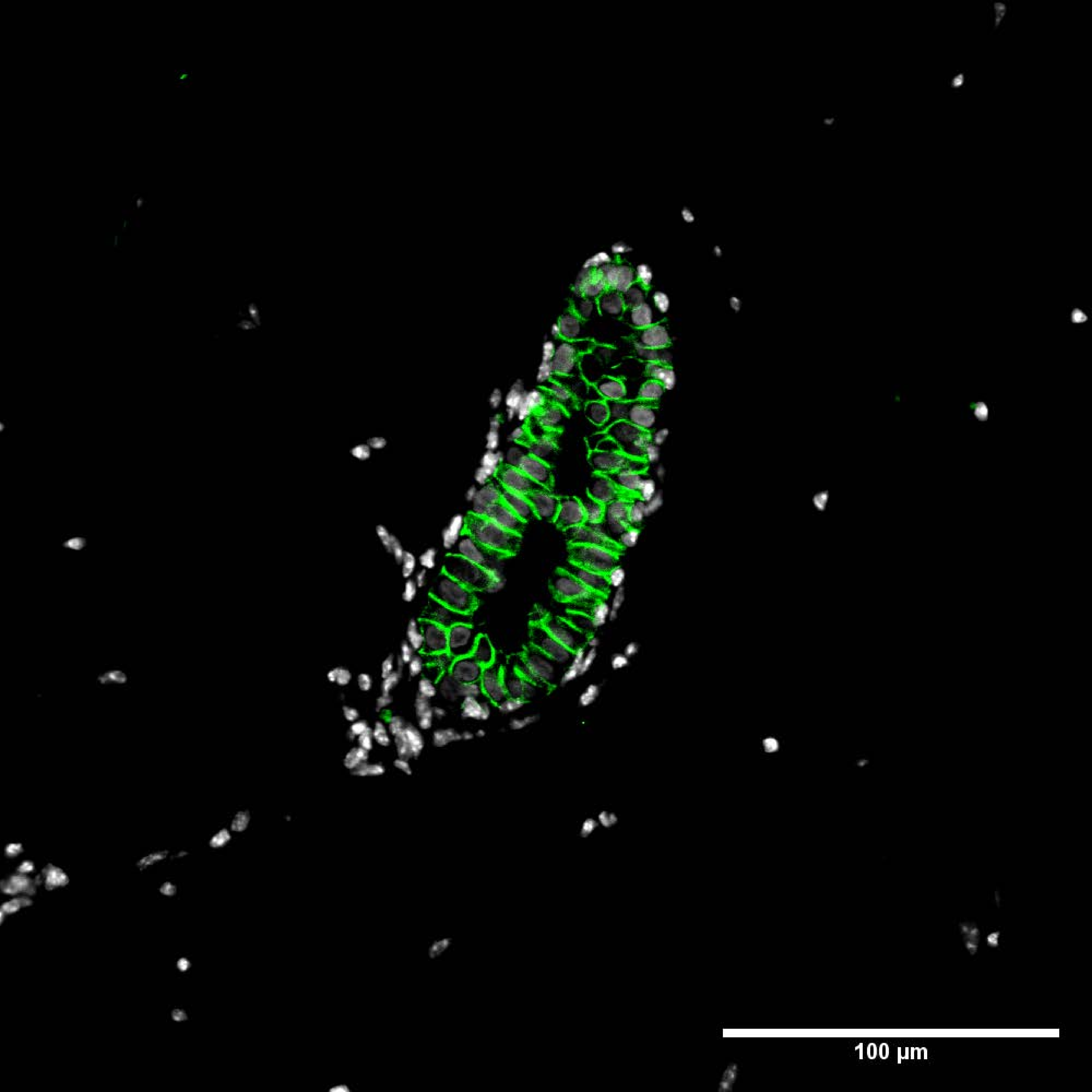
100 µm

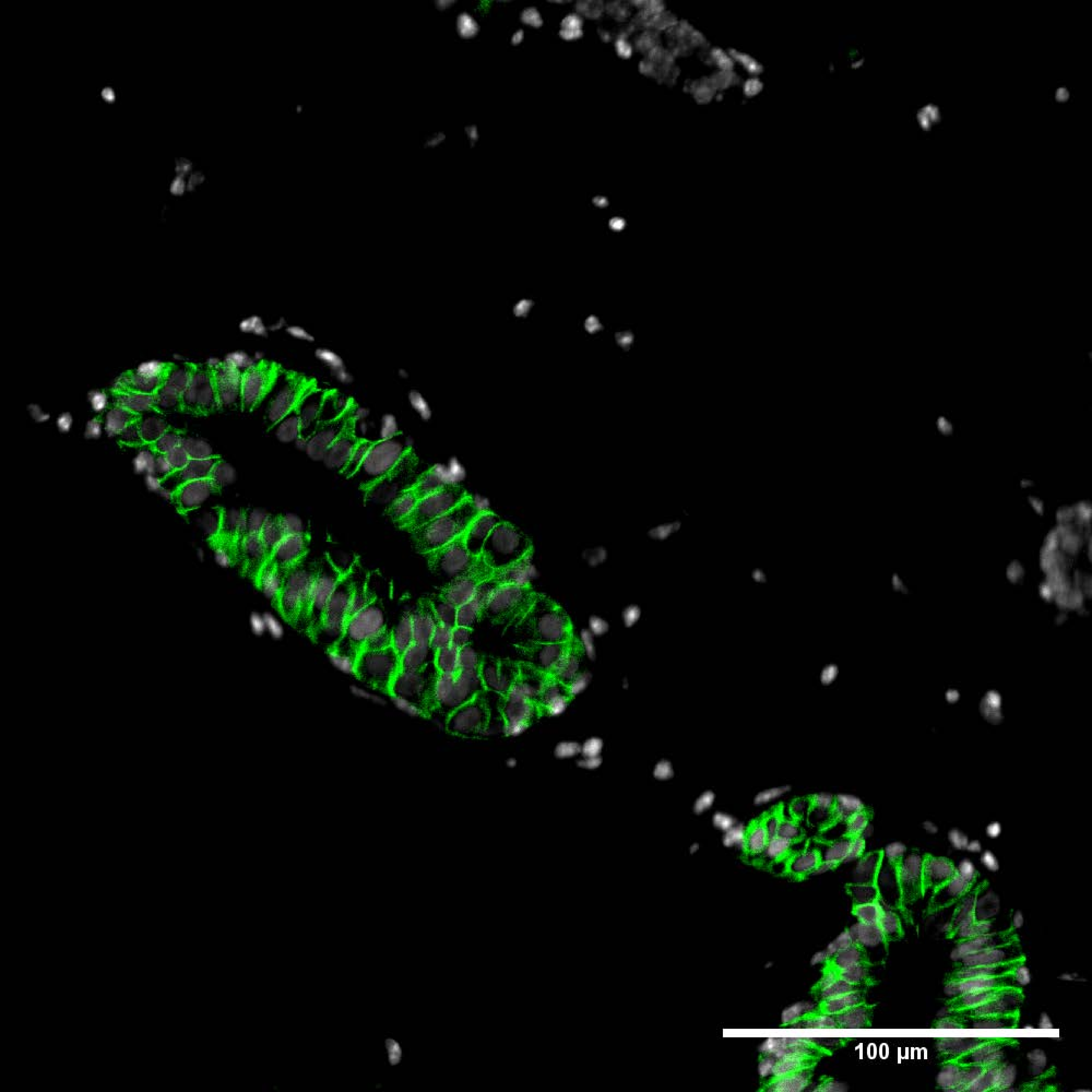
100 µm

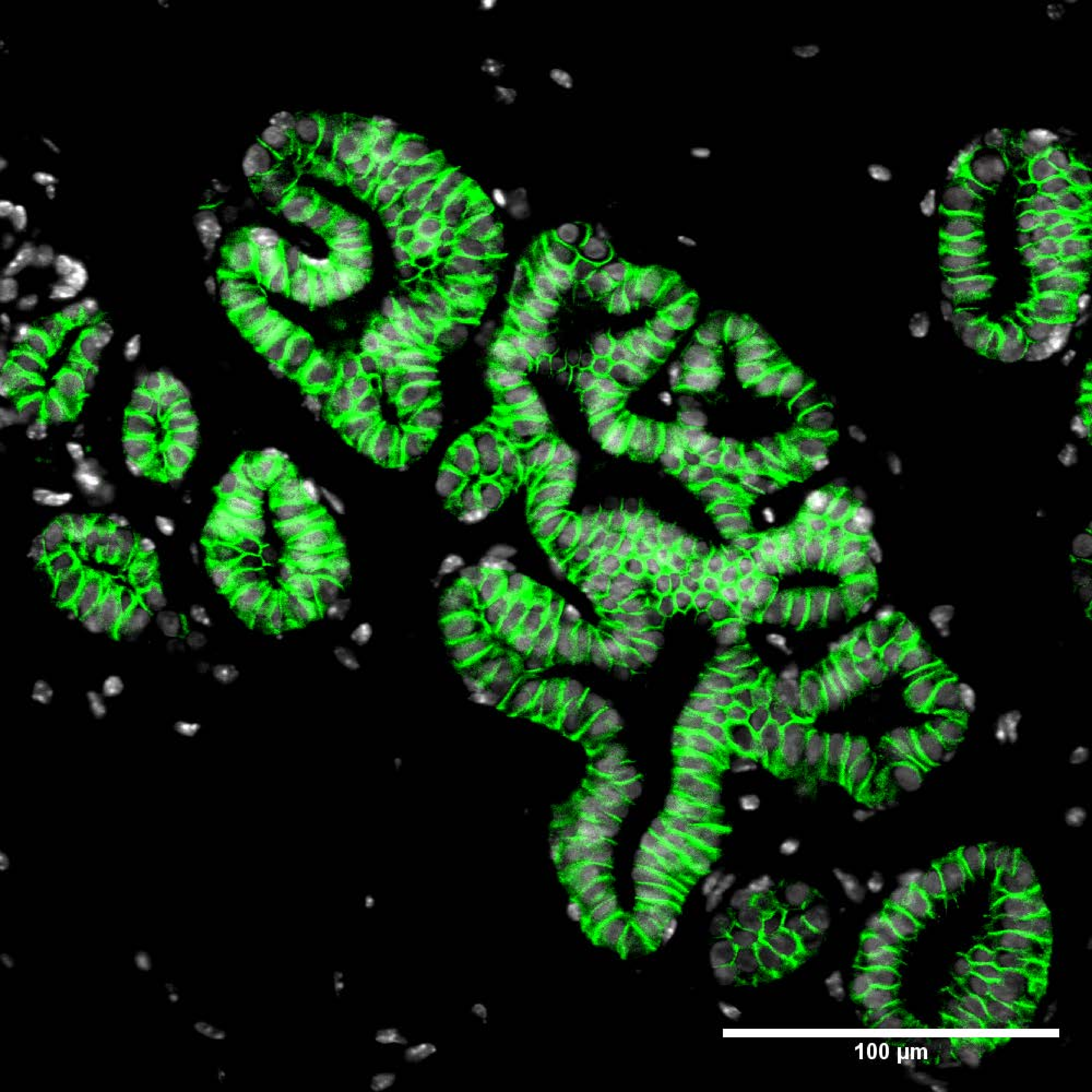
100 µm

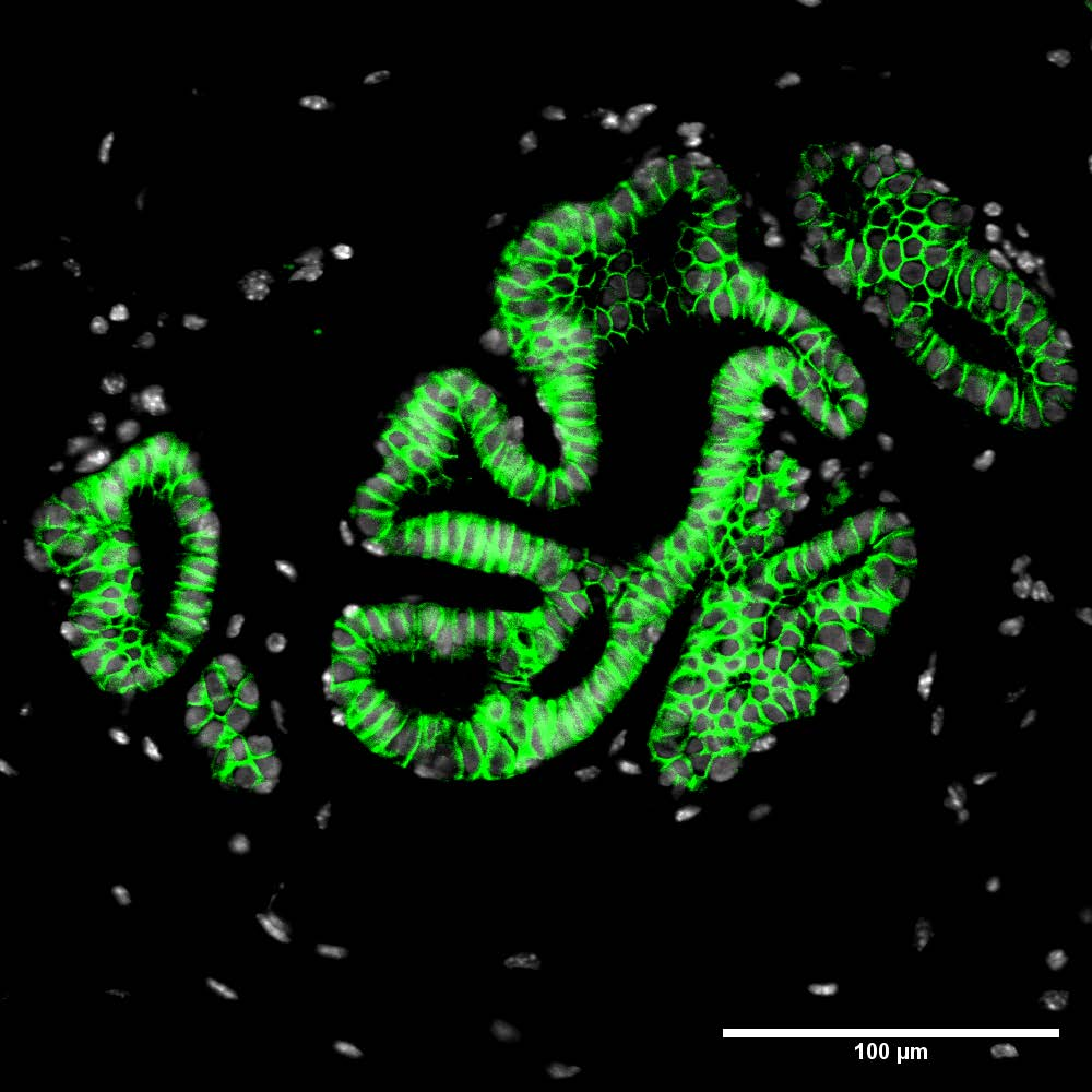
100 µm

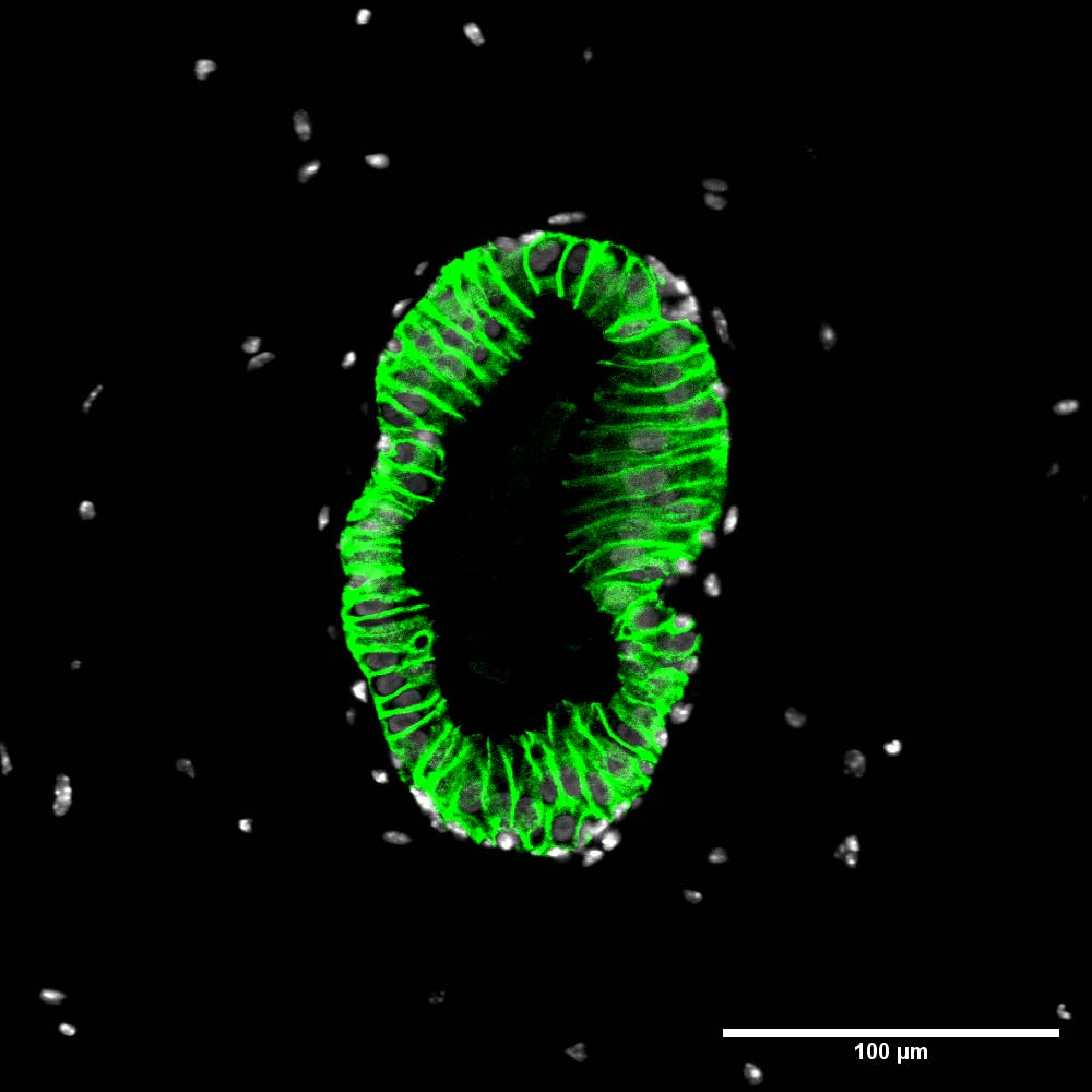
100 µm

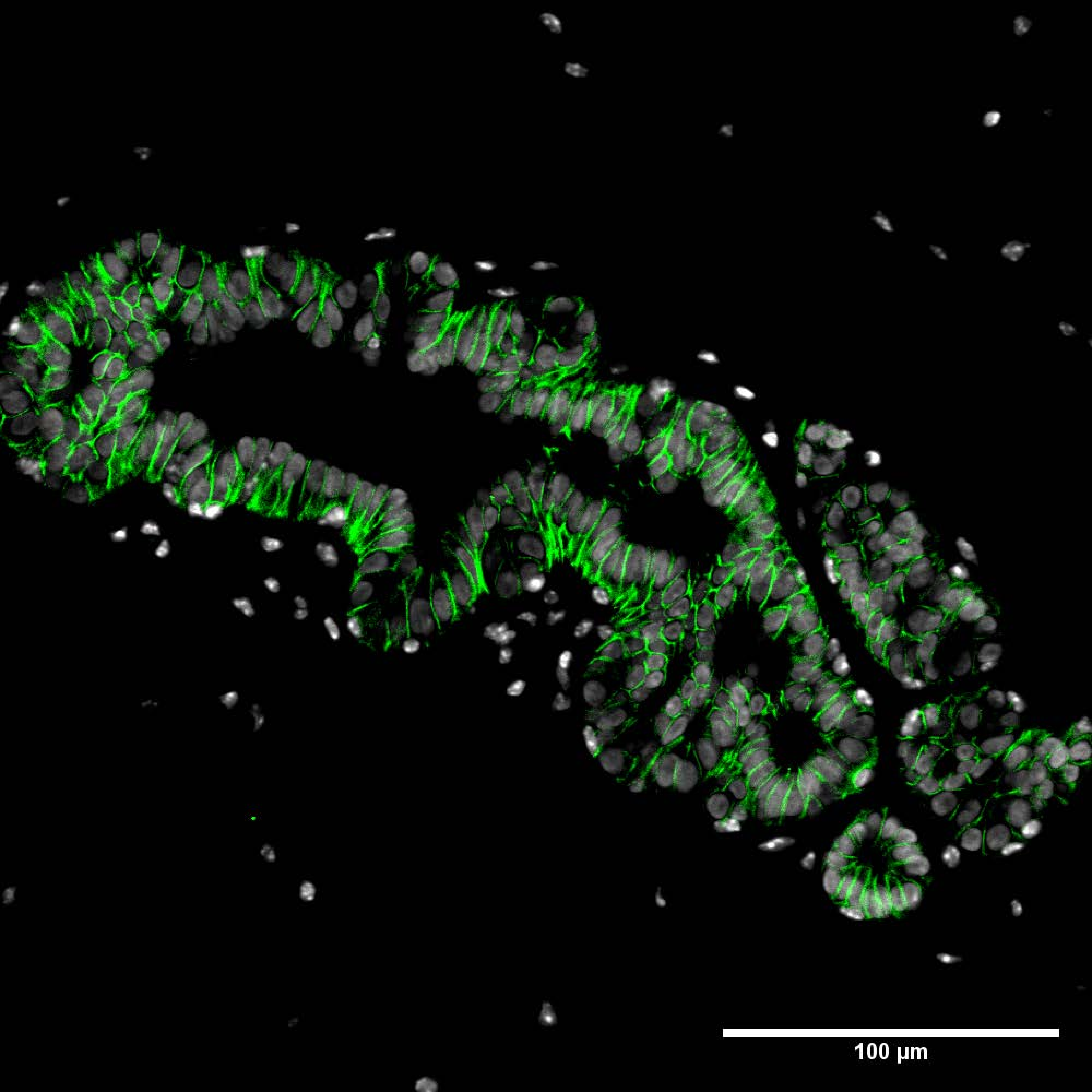
100 µm

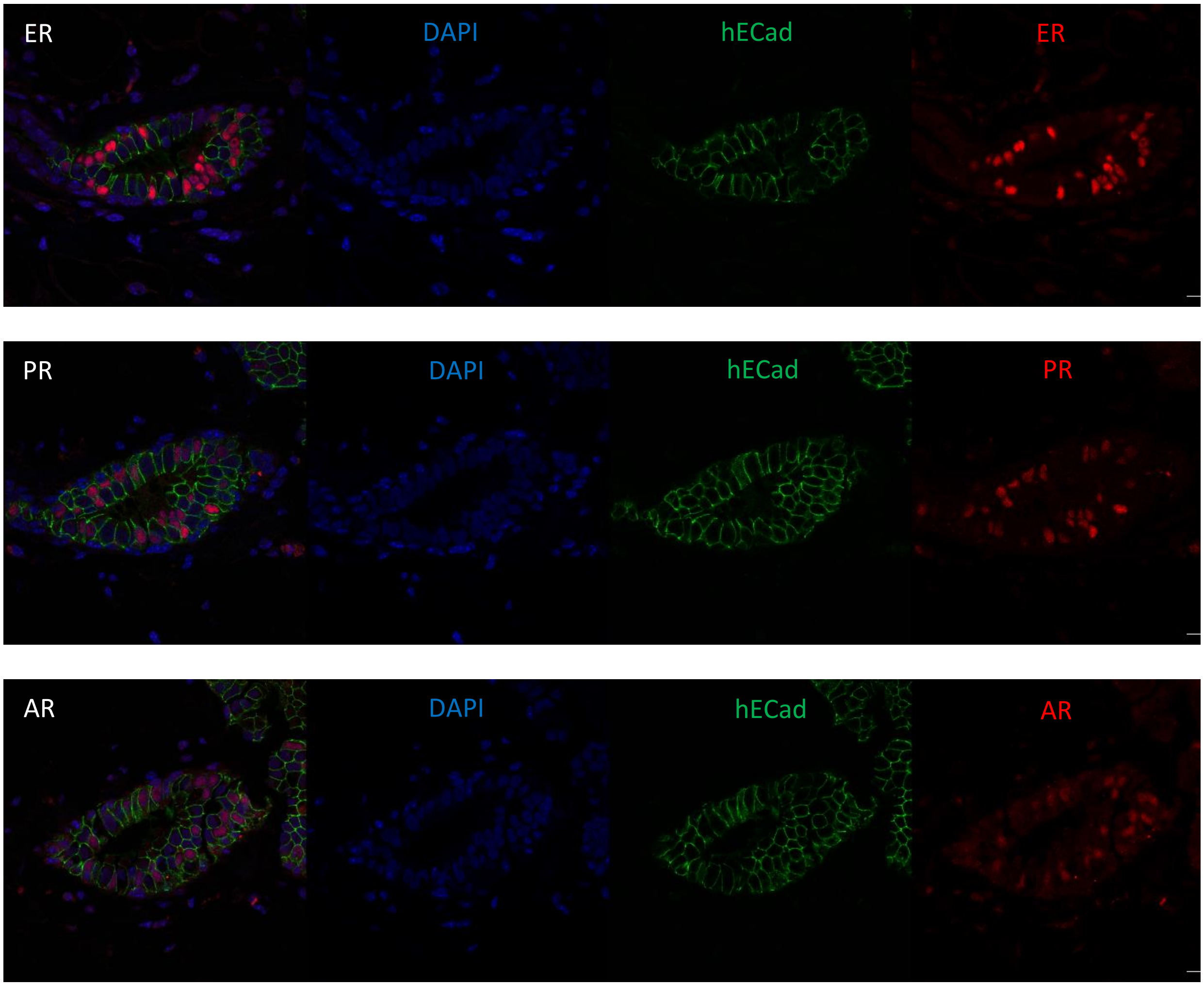

ER
DAPI
hECad
ER
PR
DAPI
hECad
PR
AR
DAPI
hECad
AR
