# Supplementary figures and images for: Contraceptive progestins with androgenic properties stimulate breast epithelial cell proliferation
Source: EMBO Mol Med. 2021 May 27;13(7):e14314. doi: 10.15252/emmm.202114314 (PMC8261488; doi:10.15252/emmm.202114314)

SCR

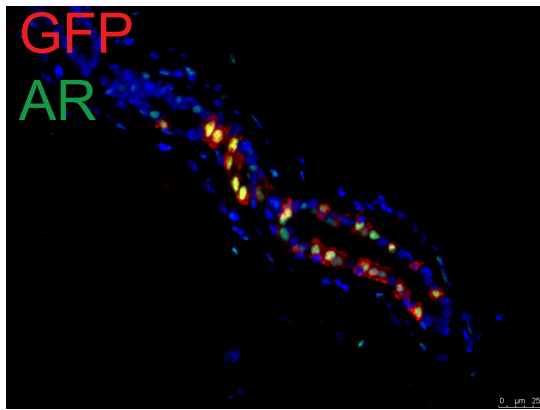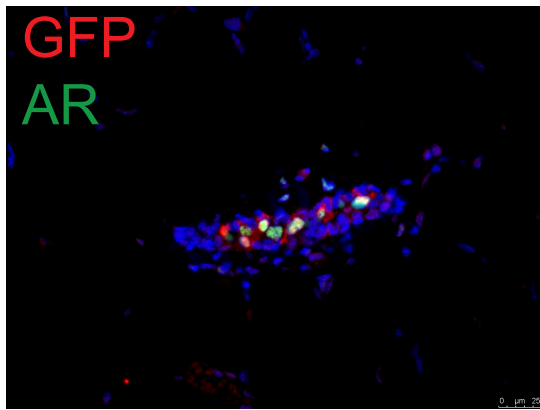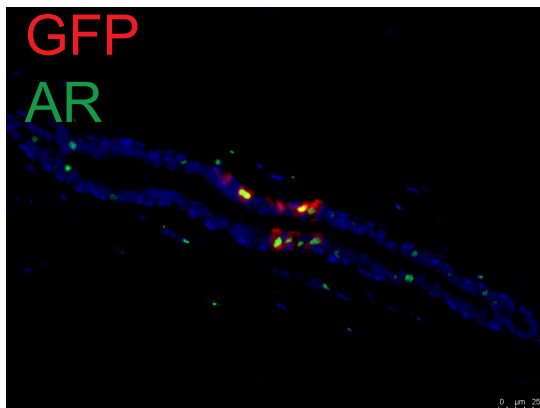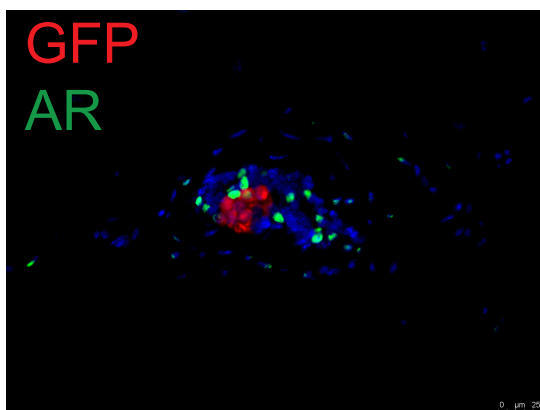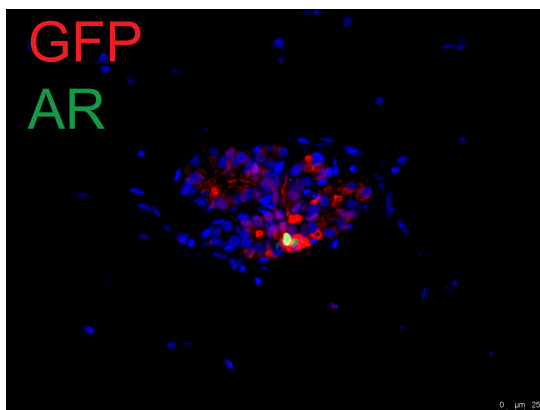

shAR

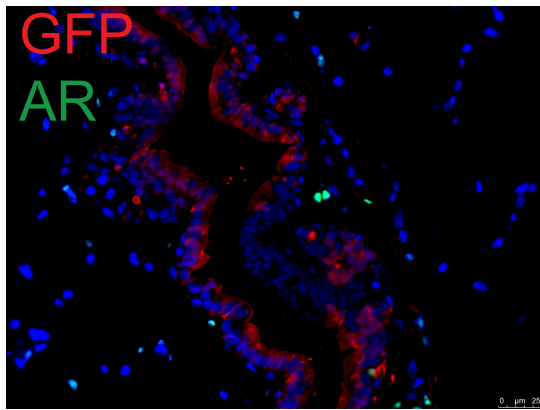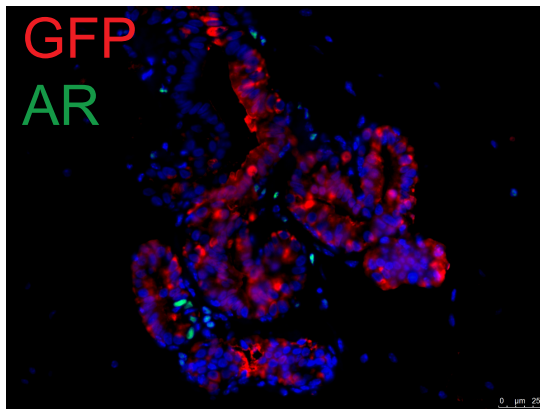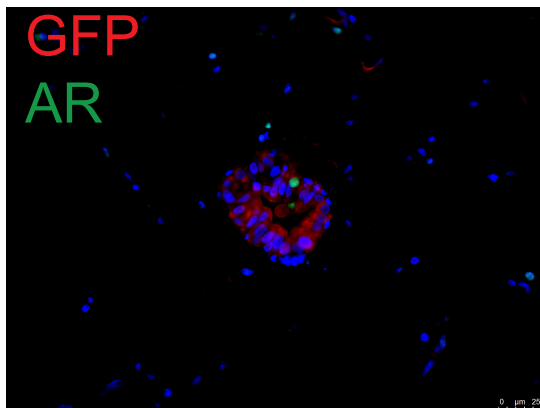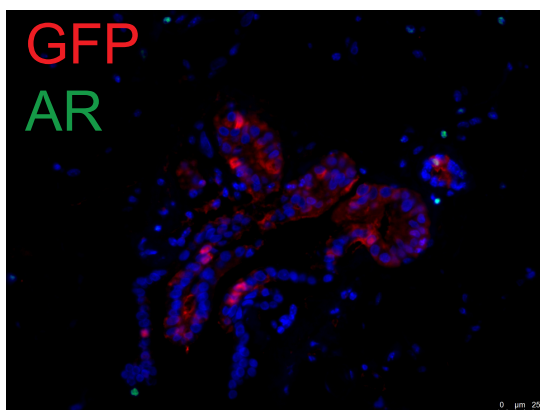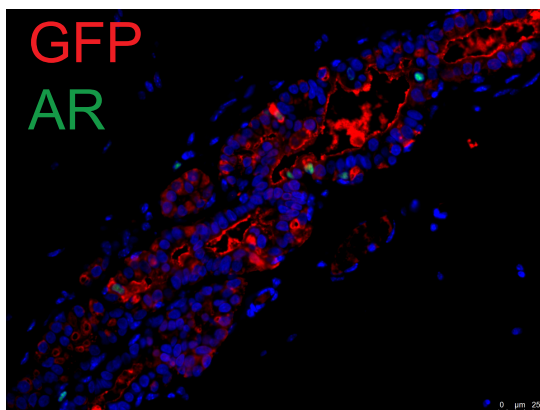

Supplement: Supplementary file 4 — Source Data for Figure 7 [file EMMM-13-e14314-s002.pdf]
